# An Unusually Broad Series of Seven Cyclombandakamines, Bridged Dimeric Naphthylisoquinoline Alkaloids from the Congolese Liana *Ancistrocladus ealaensis*

**DOI:** 10.1038/s41598-019-46336-z

**Published:** 2019-07-08

**Authors:** Dieudonné Tshitenge Tshitenge, Torsten Bruhn, Doris Feineis, Virima Mudogo, Marcel Kaiser, Reto Brun, Gerhard Bringmann

**Affiliations:** 1 Institute of Organic Chemistry, University of Würzburg, Am Hubland, D-97074 Würzburg, Germany; 2 Faculty of Pharmaceutical Sciences, University of Kinshasa, B.P. 212, Kinshasa XI, Democratic Republic of the Congo; 3 Federal Institute for Risk Assessment, Max-Dohrn-Straße 8-10, D-10589 Berlin, Germany; 4 Faculty of Sciences, University of Kinshasa, B.P. 202, Kinshasa XI, Democratic Republic of the Congo; 5 Swiss Tropical and Public Health Institute, Socinstrasse 57, CH-4002 Basel, Switzerland; 6 University of Basel, Petersplatz 1, CH-4003 Basel, Switzerland

**Keywords:** Stereochemistry, Solution-state NMR, Screening, Structure elucidation

## Abstract

A series of seven unusual dimeric naphthylisoquinoline alkaloids was isolated from the leaves of the tropical liana *Ancistrocladus ealaensis* J. Léonard, named cyclombandakamine A (1), 1-*epi*-cyclombandakamine A (2), and cyclombandakamines A_3–7_ (3–7). These alkaloids have a chemically thrilling structural array consisting of a twisted dihydrofuran-cyclohexenone-isochromene system. The 1′″-epimer of 4, cyclombandakamine A_1_ (8), had previously been discovered in an unidentified *Ancistrocladus* species related to *A. ealaensis*. Both lianas produce the potential parent precursor, mbandakamine A (9), but only *A. ealaensis* synthesizes the corresponding cyclized form, along with a broad series of slightly modified analogs. The challenging isolation required, besides multi-dimensional chromatography, the use of a pentafluorophenyl stationary phase. Featuring up to six stereocenters and two types of chiral axes, their structures were elucidated by means of 1D and 2D NMR, HRESIMS, in combination with oxidative chemical degradation experiments as well as chiroptical (electronic circular dichroism spectroscopy) and quantum chemical calculations. Compared to the ‘open-chain’ parent compound 9, these dimers displayed rather moderate antiplasmodial activities.

## Introduction

The plant family Ancistrocladaceae – together with the related Dioncophyllaceae – has been in the focus of our most recent phytochemical investigations^1–4^. This interest is justified by the exclusive occurrence of polyketide-derived naphthylisoquinoline alkaloids in these plants^5–8^. The chemical thrill induced by their broad structural diversity^2,9^, unprecedented biosynthesis, and pronounced biological activities has made them most attractive natural products^10–14^.

After the isolation of mbandakamines A and B in an as yet botanically undescribed Congolese *Ancistrocladus* species^9^, our group has intensified the search for new secondary metabolites in Central African taxa^3^, in particular in those found in the region of Mbandaka (Democratic Republic of the Congo)^15^. In this framework, we have just reported the occurrence of cyclombandakamine A_1_ (**8**)^16^, in the same ‘new’ plant, and postulated that **8** could originate biosynthetically from an ‘open-chain’ *N*-methylated mbandakamine A precursor^16^. Unfortunately, the respective precursor to **8** was not found in that plant nor was the authentic cyclization product of **9**, cyclombandakamine A (**1**) – despite the high content of **9** in the leaves of the probably ‘new’ *Ancistrocladus* species.

In recent investigations on the botanically well-described liana A*ncistrocladus ealaensis* J. Léonard^5^, we have documented the occurrence of ealapasamines A-C^17^, michellamine-type and mbandakamine-like dimers^18^, and of a plethora of further, monomeric naphthylisoquinoline alkaloids^18^. These findings already suggested an outstanding biosynthetic potential of the Central African liana *A. ealaensis* compared to other known *Ancistrocladus* species.

**Figure 1:**
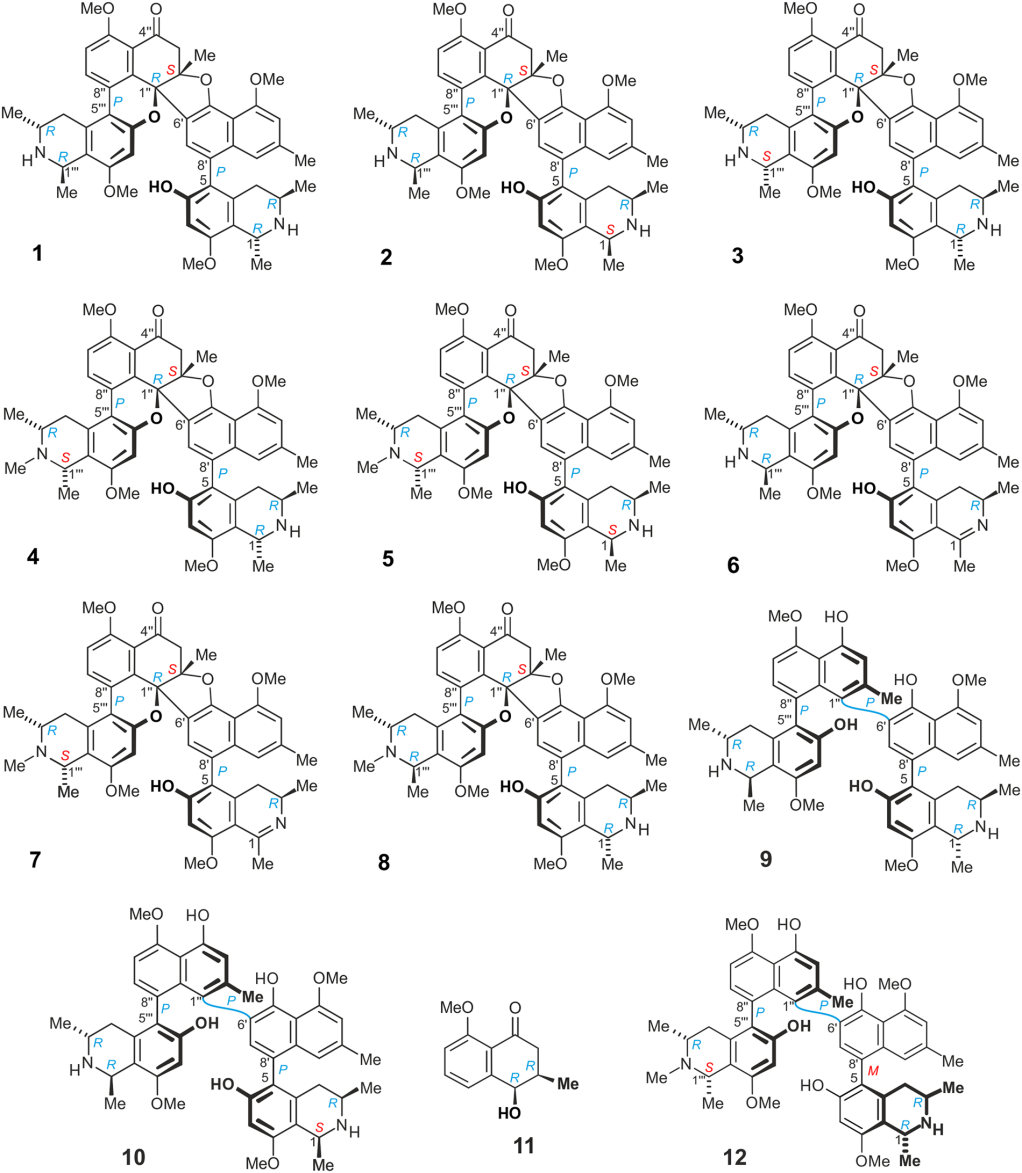
Cyclombandakamine A (1), 1-*epi*-cyclombandakamine A (2), and cyclombandakamines A_3_-A_7_ (3–7) isolated from leaf material of *A. ealaensis*. Also depicted are the yet known cyclombandakamine A_1_ (8), mbandakamines A (9), C (10) and E (12), and *cis*-isoshinanolone (11).

The systematic analysis of leaf extracts of *A. ealaensis*, which had already revealed the presence of three types of unsymmetric dimeric naphthylisoquinoline alkaloids^17,18^, showed that it was not unlikely to find further singularities. These investigations led to the isolation and purification of cyclombandakamine A (**1**), its 1-epimer **2**, and five related dimeric alkaloids, **3–7**, all featuring an unusual condensed cage-like polycyclic backbone (Fig. 1). Their structural elucidation is herein described, as well as their anti-infective profiles.

## Results and Discussion

### Isolation

The spectroscopy-guided analysis of a crude leaf extract of *A. ealaensis* by LC-DAD and LC-MS hinted at the presence of a new type of alkaloid, with unusual UV spectra and MS patterns typical of dimeric naphthylisoquinoline alkaloids. The leaf material was macerated with MeOH, and the extract was further exhaustively partitioned between water and dichloromethane. The organic phases were collected and submitted to reversed-phase chromatography to obtain more slowly eluting subfractions, in which the hard-to-resolve cyclombandakamines were enriched. The isolation by semi-preparative HPLC – on different stationary materials – afforded seven such alkaloids in a pure form.

### Structural elucidation of compounds 1–7

#### Cyclombandakamine A (1)

Its resolution succeeded only on a silica-bonded High-Speed F5 column (having a pentafluorophenyl phase), showing a molecular formula of C_48_H_50_N_2_O_8_ and, thus, an Index of Hydrogenation Deficiency (IHD) of 25 as deduced by HRESIMS. This molecular formula was identical to that of mbandakamine D^18^, but the UV and the ECD spectra, and the NMR data were significantly different, hinting at an unknown unsymmetric dimer with 48 non-equivalent carbon atoms. Most remarkable was the presence of seven – instead of eight – aromatic protons in the ^1^H-NMR spectrum, and of three unusual carbon signals in ^13^C NMR. Its demanding absolute stereostructure was elucidated by means of extensive 1D and 2D NMR experiments, ruthenium-mediated oxidative degradation, ECD spectroscopy, and DFT-based ECD spectra calculations (see also Supporting Information).

**Figure 2:**
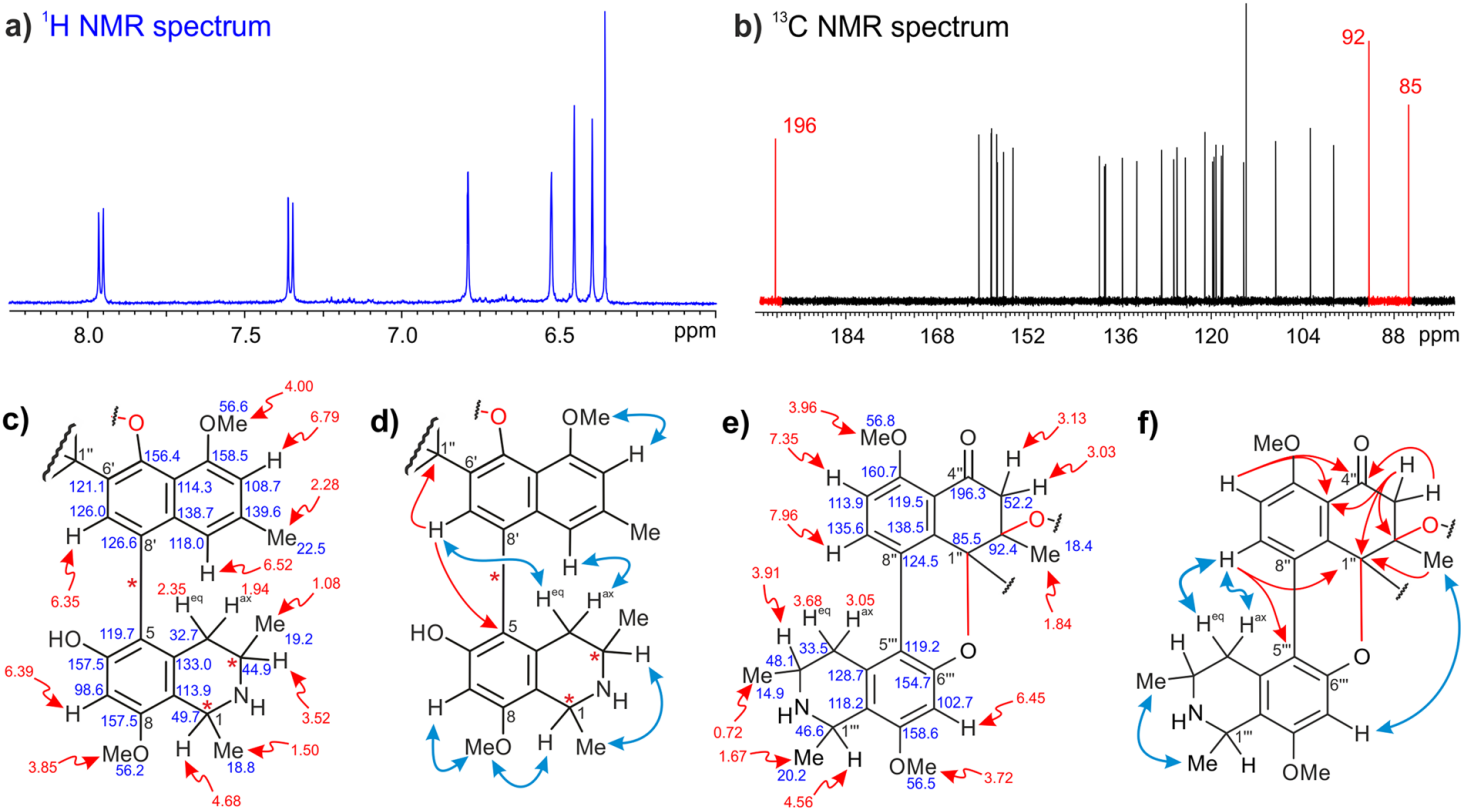
Parts of the (**a**) ^1^H- and (**b**) ^13^C-NMR spectra of cyclombandakamine A (1). Chemical shifts, selected ROESY (double blue arrows) and HMBC (single red arrows) interactions in the southeastern (**c**,**d**) and the northwestern (**e**,**f**) portions of 1.

As in the case of **9**, the ‘southeastern’ portion of the molecule showed four aromatic protons, but also two methoxy functions and seven further signals in the ^1^H NMR spectrum (Fig. 2). Data analysis revealed a tetrahydroisoquinoline subunit, which possessed one aromatic singlet at H-7 (*δ*_H,C_ 6.39, 98.6) interacting in HMBC with two oxygenated carbons, C-6 and C-8. The presence of an *O*-methyl group at C-8 was evidenced by ROESY interactions from H-7 and H-1 (*δ*_H,C_ 4.68, 49.7) to 8-OMe (*δ*_H_ 3.85), leaving the unprotonated C-5 (*δ*_C_ 119.7) as the coupling position. The typical two methyl doublets at C-1 and C-3, the signals of the two diastereotopic protons at C-4 (*δ*_H_ 1.94 and 2.35), and the multiplet at C-3 (*δ*_H,C_ 3.52, 44.9) were also detected as parts of this subunit (Fig. 2c). Its relative *trans*-configuration at C-1 *vs*. C-3 was assigned by the ROESY interaction from the doublet of 1-Me (*δ*_H_ 1.50) to the multiplet of H-3 (Fig. 2d).

Attached to this moiety was a two-fold oxygenated naphthalene unit possessing one pair of *meta*-coupled protons at C-1′ (*δ*_H,C_ 6.52, 118.0) and C-3′ (*δ*_H,C_ 6.79, 108.7), one aromatic proton singlet H-7′ (*δ*_H,C_ 6.35, 126.0), and signals for a methoxy function at C-4′ and a methyl group at C-2′. The presence of a proton singlet at C-7′ suggested that C-6′ and C-8′ were quaternary. This assumption was corroborated by two HMBC interactions with carbon atoms belonging to another spin system. The HMBC cross peak between H-7′ and C-5 indicated a 5,8′-linkage to the tetrahydroisoquinoline unit (Fig. 2c,d). Additionally, H-7′ showed ROEs with 4-H_eq_ (*δ*_H_ 2.35), while H-1′ exhibited cross-peaks in both dimensions with 4-H_ax_ (*δ*_H_ 1.94), confirming the coupling site of the two subunits and, thus, establishing the relative overall configuration in this 5,8′-coupled portion, *viz*. 5*P*,1*R*,3*R* or 5*M*,1*S*,3*S*. The oxidative degradation following a procedure developed earlier^19^, established C-3 and C-3′″ to be *R*-configured, from which the overall absolute configuration in this molecular half was deduced as 5*P*,1*R*,3*R*, like in **9**.

The HMBC correlation from H-7′ to one of the uncommon signals, viz., at a resonance of *δ*_C_ 85.5 – located in the ‘northwestern’ portion – showed the necessity to elucidate its direct chemical environment. In addition to H-7′, a methyl group at *δ*_H,C_ 1.84 and 18.4 (2″-Me), two diastereotopic-proton doublets at *δ*_H_ 3.13 (3″-H_ax_) and 3.03 (3″-H_eq_) exhibited ^3^*J*_C,H_-HMBC cross peaks with the same quaternary carbon at *δ*_C_ 85.5 (C-1″). One binding site of C-1″ remained undefined, but its low-field chemical shift suggested a linkage to an electronegative group, probably a heteroatom. The presence of the two diastereotopic protons at C-3″ (*δ*_C_ 52.2) was unprecedented and suggested its proximity to a stereocenter. The methyl group (singlet) attached to an aliphatic carbon C-2″ (*δ*_C_ 92.4), showed exclusive HMBC interactions to C-1″, C-2″, and C-3″ (Fig. 2e,f). This indicated, on the one hand, the location of the methyl group to be between C-1″ and C-3″ and, on the other hand, revealed the lack of a proton at C-2″, which should be the additional quaternary carbon atom in this spin system. One substituent at C-2″ remained yet unclear at this point, but was well defined at a later stage of the structural analysis.

A carbonyl function was detected at C-4″ (*δ*_C_ 196.3) showing HMBC correlations with the diastereotopic protons 3′-H_eq_ and 3′-H_ax_. An aromatic-proton doublet in the ^1^H spectrum displayed a ^4^*J*_C,H_-HMBC interaction to C-4″, hinting at the connection to a remote molecular fragment. This doublet (*J* = 9.0 Hz) at a resonance of *δ*_H_ 7.35 was *ortho*-coupled to another one, but more deshielded, at *δ*_H_ 7.96. The ROESY interaction between a methoxy group (5″-OMe, *δ*_H,C_ 3.96, 56.8) and H-6″ (*δ*_H,C_ 7.35, 113.9), and the HMBC correlation from H-7″ (*δ*_H,C_ 7.96, 135.6) to C-5″ (*δ*_C_ 160.7) clarified the assignment in the aromatic system as depicted in Fig. 2e,f.

Moreover, ^4^*J*_C,H_-HMBC correlations from H-6″ and 3″-H_eq_ to a quaternary carbon at the resonance of *δ*_C_ 119.5 (C-10″), from H-7″ to C-9″ (*δ*_C_ 138.5), and the ^4^*J*_C,H_-HMBC cross peak observed from H-7″ to C-1″ unambiguously proved the presence of a tetralone substituent instead of a classical naphthalene moiety. In other *Ancistrocladus* species, such acetogenic tetralones – yet free, without an isoquinoline portion – like *cis*-isoshinanolone (**11**)^20^ were known to occur, but had never been found as part of a natural naphthylisoquinoline alkaloid^14^.

This tetralone portion related to *cis*-isoshinanolone (**11**)^21^ showed a pair of *ortho*-coupled aromatic protons at H-6″ and H-7″, leaving C-8″ (*δ*_C_ 124.5) as the connecting point to another spin system. In the HMBC spectrum, an additional cross peak observed from H-7″ to a quaternary carbon at *δ*_C_ 119.2 (C-5′″) suggested the existence of a second linkage to the tetralone. One aromatic singlet at H-7′″ (*δ*_H,C_ 6.45, 102.7) displayed HMBC correlations with C-5′″, C-6′″ (*δ*_C_ 154.7), C-8′″ (*δ*_C_ 124.5), C-9′″ (*δ*_C_ 118.2), and C-1′″ (*δ*_C_ 46.6), reminiscent of a classical 1,3-dimethyl-tetrahydroisoquinoline subunit as in the southeastern part of the dimer (Fig. 2d). The characteristic quartet at C-1′″, the multiplet at C-3′″, and the doublets of doublets of the two diastereotopic protons at C-4′″, typical of a tetrahydroisoquinoline, were observed and assigned accordingly. By ROESY analysis, a relative *trans*-array of the two methyl groups was established in this moiety. The oxidative degradation revealed that all C-3 related positions were *R*-configured, thus leading to the assignment of a 1′″R,3′″R-tetrahydroisoquinoline, 5′″,8″-coupled to a 5″-methoxy-2″-methyl tetralone unit, in this second portion (see Fig. 2e,f).

At this stage of the structural analysis, all signals detected in the ^13^C and ^1^H NMR spectra had been reliably assigned, and all the ten heteroatoms suggested by the molecular formula were positioned. Still, the calculated degree of unsaturation related to C_48_H_50_N_2_O_8_ was not yet reached, but, as yet, amounted to 23 instead of 25. This discrepancy by two units could only be explained by the formation of two additional, heterocyclic rings. As mentioned before, the resonance of the quaternary, and sp^3^-hybridized C-1″ suggested its connection to a heteroatom, and in this case, only an oxygen function attached to C-6′″ could explain its deshielded chemical shift at *δ*_C_ 85.5. This assumption was proven by the observed ROESY interactions from H-7″ to the two diastereotopic protons at C-4′″, proposing a quasi-coplanar orientation of the tetrahydroisoquinoline unit towards the substituted tetralone part, consecutive to the formation of a new pyran ring involving the oxygen function between C-6′″ and C-1″ (see Fig. 2f).

In the tetralone unit, the fourth bond of the quaternary C-2″ had so far remained undefined. Its very low-field shifted resonance at *δ*_C_ 92.4 hinted at a linkage to one of the neighboring oxygen functions, which in this case could only be the one at C-5′, consistent with its chemical shift. The ROESY correlations observed between 4′-OMe and 2″-Me supported the presence of the resulting five-membered dihydrobenzofuran ring attached to the tetralone. By these investigations, the full constitution of the new dimer was established (Fig. 2e,f), displaying two oxygen-bridged heterocyclic rings, both unprecedentedly attached to a tetralone unit.

In contrast to the southeastern portion, the determination of the absolute configuration in the northwestern one turned out to be challenging because of the bridged chiral axis and the two additional stereogenic centers in the cyclohexenone ring, which could not be directly solved by oxidative degradation. However, the steric constraints imposed by the highly condensed ring systems enabled the discrimination between the possible stereoisomers. As mentioned above, the formation of the pyran ring forced the – usually orthogonally oriented – isoquinoline to become nearly coplanar to the tetralone ‘plane’, with an angle of only 28.5°. This unprecedented orientation brought the axial and equatorial diastereotopic protons at C-4′″ in the vicinity of H-7″, as attested in the ROESY spectrum. However, a stronger ROE interaction of H-7″ to 4′″-H_eq_ indicated that the bridged chiral axis between C-5′″ and C-8″ in this molecular half was *P*-configured.

Such a *P*-configured biaryl axis additionally implied that the oxygen bridge at C-1″ must have been formed from ‘above’ the tetralone plane, thus suggesting the absolute configuration at C-1″ to be *R*. In this molecular context, C-1″ would have to be *S* if the axis was *M*-configured, but that would not have been in agreement with the observed ROE interactions between H-7″ and 4′″-H_eq_, those from H-7 and H-7′ to 4′″-H_ax_, and those from 3′″-Me to H-7 and H-7″. These data all strongly supported an *R*-configuration at C-1″.

**Figure 3:**
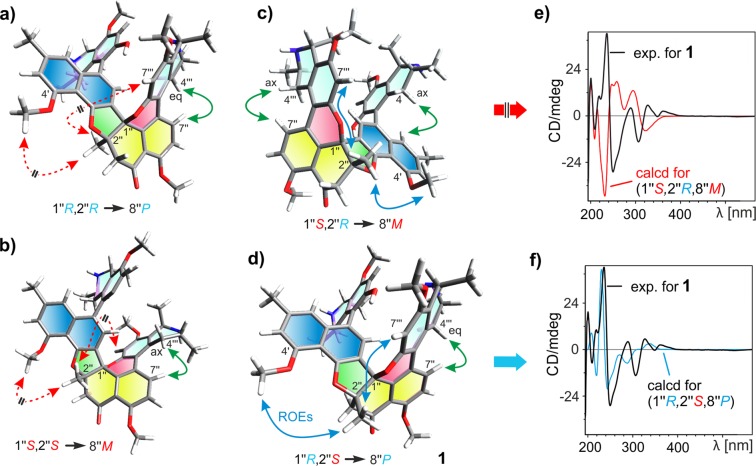
(**a**–**d**) Minimum structures (calculated at the DFT-B3LYP-D3/def2-TZVP level) of the four most plausible stereoisomers of cyclombandakamine A (1), with the given absolute configurations and the expected crucial ROEs for the relative configuration. The ROESY interactions in blue are indicative of the *trans-* or *cis-*orientation in the cyclohexanone while those in red are not expected to be observed. The green double arrows suggest the strongest ROEs between H-7″ and one of the diastereotopic protons at C-4′″, relevant for the axial configuration. (**e**) and (**f**) The experimental ECD spectrum of 1 compared to the curves calculated for the two most plausible *trans*-diastereomers (1″S,2″R and 1″R,2″S) in the tetralone subunit^16^, confirming that the isolated structure of 1 is the one shown in (**d**). The ECD spectra were calculated at the TDCAM-B3LYP/def2-TZVP level^16^.

The analysis of the DFT-calculated minimum structures (Fig. 3a–d) indicated that in the case of an *R-*configured C-2″ (Fig. 3a), the methyl 2″-Me should be under the ‘tetralone plane’, and thus, on the opposite side of the new bridge at C-1″, and would be expected to show long-range ROESY interactions to 4-H_eq_, given the calculated distance of 3.269 Å – but no interaction to 4′-OMe should be possible. Additionally, these investigations predicted a decisive ROE interaction between 2″-Me and H-7′″, which was found to be corroborative for the relative *trans*-configuration in the tetralone, *viz*. 1″R,2″S or 1″S,2″R. With the known configuration at C-1″ (*R*), only a 1″R,2″S array was plausible for the cyclohexenone unit.

Moreover, the analysis of optimized structures featuring the relative *cis*-configuration, *i.e*. 1″R,2″R or 1″S,2″S, indicated that 2″-Me and H-7′″ showed opposite spatial orientations and should not exhibit cross peaks in the ROESY investigations. Thus, the absolute configuration at C-2″ was confirmed to be *S* (Fig. 3d).

Based on the aforementioned assignment, the full stereochemical array of the dimer **1** could only be 5*P*,1*R*,3*R*,1″R,2″S,8″P,1′″R,3′″R as shown in Figs 1 and 3d. Additionally, the structural attributions agreed with the observed ROESY interactions across the two molecular halves, in particular those between H-7 and H-1′″, H-7′ and H-1′″, H-3 and 4′″-H_ax_, and from 3′″-Me to 8-OMe.

Additional proof was provided by time-dependent density functional theory (TDDFT) calculations of the ECD spectra, performed with TDCAM-B3LYP/def2-TZVP (Fig. 3e,f)^16^. The two relevant diastereomers featuring a *cis*- (1″S,2″R,8″M) and a *trans*-configured (1″R,2″S,8″P) cyclohexenone were analyzed as previously described^16^. As anticipated, the experimental and the calculated ECD spectra for the *trans-*diastereomer of **1** showed a good agreement, while the *cis-*diastereomer (1″S,2″R,8″M) displayed an opposite ECD curve, confirming the assignment established above. Due to its structural array reminiscent of the known – and co-occurring – ‘open-chain’ dimer mbandakamine A (**9**), the new compound **1** was named cyclombandakamine A.

#### 1-epi-Cyclombandakamine A (2)

Another compound, showing the same UV spectrum and the same molecular formula by HRESIMS as cyclombandakamine A (**1**), was also purified on a preparative HS F5 HPLC column. The structural data analysis (see Supporting Information) led to a constitution identical to that of **1**. The typical seven aromatic protons, the two unusual doublets corresponding to two diastereotopic protons at C-3″, and the characteristic carbon signals at *δ*_C_ 196.3, 92.4, and 85.4 ppm were clearly visible. The singularity of compound **2** as compared to cyclombandakamine A (**1**) was its relative *cis*-configuration – instead of *trans* as in **1** – between C-1 and C-3 as evidenced by the ROE interaction between H-1 (*δ*_H,C_ 4.52, 52.0) and H-3 (*δ*_H,C_ 3.09, 50.6). Moreover, the deshielded shift of C-3 and the shielded value of H-1 were in agreement with the ROE-based attribution. This alkaloid indicated no further dissimilarities to **1**, thus leading to the elucidation of its structure as being 1-*epi*-cyclombandakamine A (**2**), which could also be addressed as cyclombandakamine C based on the fact that its potential precursor – mbandakamine C (**10**)^18^ – had been found in *A. ealaensis*, too.

#### Cyclombandakamine A_3_ (3)

Semi-preparative resolution on a Chromolith^®^ column enabled the purification of a trace alkaloid, which, astonishingly, showed the same UV spectrum, the same molecular formula by HRESIMS, and the same constitution by NMR as **1** and **2** (see Supporting Information). The southeastern portion was stereochemically identical to the one described for **1**, and, thus, clearly different from that of **2**. In the northwestern portion, ROESY interactions between H-1′″ (*δ*_H,C_ 4.49, 52.9) and H-3′″ (*δ*_H,C_ 3.81, 50.2) evidenced their diaxial orientation, from which a relative *cis*-configuration was attributed. Therefore, the only difference to cyclombandakamine A (**1**) was the absolute configuration at C-1′″, being *S-*configured. This structural difference was also in agreement with a new ROESY cross-peak between 1′″-Me (*δ*_H,C_ 1.63, 20.4) and H-7 (*δ*_H,C_ 6.39, 98.6), while no ROE interactions were monitored between H-7 and H-1′″. The new alkaloid **3** was named cyclombandakamine A_3_ (**3**), but it might also be addressed as 1′″-*epi*-cyclombandakamine A.

#### Cyclombandakamine A_4_ (4)

In the same fraction from which **3** was isolated, the main chromatographic peak eluting more slowly on Chromolith^®^ led to a molecular formula of C_49_H_52_N_2_O_8_ by HRESIMS, suggesting the presence of an additionally methylated analog of **1**. Extensive structural data indicated strong similarities to cyclombandakamine A, evidenced by all the so far typical signals in the NMR spectra (Fig. 2a,b, and Table 1). While no difference was noticed compared to **1** in the southeastern part, the new alkaloid **4** showed an additionally *N*-methylated tetrahydroisoquinoline subunit instead. This was evidenced by a singlet at a resonance of 3.03 ppm, corresponding to a methyl group on the nitrogen (2′″-NMe, *δ*_C_ 49.3). Moreover, the ^1^H- and ^13^C NMR shifts at C-1′″ (*δ*_H,C_ 5.04, 71.3) and C-3′″ (*δ*_H,C_ 4.46, 63.3) were extremely deshielded, hinting at a relative *cis*-configuration in this *N*-methylated subunit, as known from mbandakamine E (**12**)^18^. ROESY interactions from the low-field shifted quartet of H-1′″ to the unusual multiplet H-3′″ confirmed this relative configuration. Its stereochemically assigned elements of chirality – 5 *P*,1 *R*,3 *R*,1″R,2″S,8″P,1′″S,3′″R – were in line with the observed ROESY interactions across the molecular halves, namely between 2′″-*N*Me and H-7 (*δ*_H,C_ 6.39, 98.4), and those from 8-OMe (*δ*_H,C_ 3.88, 56.3) to 2′″-*N*Me. The new alkaloid was named cyclombandakamine A_4_ (**4**).

**Table 1:** ^1^H (600 MHz) and ^13^C (151 MHz) data of the cyclombandakamines 1–7 in methanol-*d*_4_.

Position	1		2	3	4	5		6	7
	*δ*_H_ (Hz)	*δ*_C_, type	*δ*_C_, type	*δ*_C_, type	*δ*_C_, type	*δ*_H_ (Hz)	*δ*_C_, type	*δ*_C_, type	*δ*_C_, type
1	4.68, q	49.7, CH	52.0, CH	49.0, CH	49.1, CH	4.53, q	52.0, CH	175.2, C	175.4, C
3	3.52, m	44.9, CH	50.6, CH	44.8, CH	44.9, CH	3.09, m	50.6, CH	48.8, CH	48.8, CH
4	2.35, dd	32.7, CH^eq^	32.9, CH^eq^	32.8, CH^eq^	32.8, CH^eq^	2.17, dd	33.0, CH^eq^	32.6, CH^eq^	32.6, CH^eq^
	1.94, dd	32.7, CH^ax^	32.9, CH^ax^	32.8, CH^ax^	32.8, CH^ax^	2.05, dd	33.0, CH^ax^	32.6, CH^ax^	32.6, CH^ax^
5		119.7, C	119.9, C	119.8, C	120.0, C		120.0, C	122.2, C	122.2, C
6		157.5, C	156.5, C	157.6, C	157.1, C		157.0, C	167.4, C	167.2, C
7	6.39, s	98.6, CH	98.6, CH	98.6, CH	98.4, CH	6.39, s	99.1, CH	99.3, CH	99.0, CH
8		157.5, C	158.5, C	157.5, C	157.7, C		158.6, C	165.6, C	165.7, C
9		113.9, C	113.9, C	114.2, C	114.3, C		114.2, C	108.2, C	108.5, C
10		133.0, C	135.1, C	133.1, C	133.6, C		133.6, C	142.7, C	143.0, C
1′	6.52, s	118.0, CH	118.1, CH	118.0, CH	117.9, CH	6.55, s	118.1, CH	117.7, CH	117.6, CH
2′		139.6, C	139.6, C	139.6, C	139.8, C		139.8, C	140.1, C	140.1, C
3′	6.79, s	108.7, CH	108.7, CH	108.7, CH	108.8, CH	6.81, s	108.7, CH	108.8, CH	108.9, CH
4′		158.5, C	158.5, C	158.5, C	158.5, C		158.5, C	158.5, C	158.5, C
5′		156.4, C	156.4, C	156.4, C	156.6, C		156.6, C	156.8, C	157.0, C
6′		121.1, C	121.2, C	121.2, C	121.0, C		121.0, C	121.1, C	121.0, C
7′	6.35, s	126.0, CH	126.3, CH	126.1, CH	126.0, CH	6.37, s	126.2, CH	126.0, CH	125.9, CH
8′		126.6, C	126.3, C	126.5, C	126.6, C		126.1, C	124.9, C	124.7, C
9′		138.7, C	139.0, C	138.4, C	138.7, C		139.0, C	138.5, C	138.5, C
10′		114.3, C	114.4, C	114.3, C	114.4, C		114.5, C	114.3, C	114.3, C
1-Me	1.50, d	18.8, Me	20.2, Me	18.8, Me	18.8, Me	1.69, d	20.2, Me	24.9, Me	25.0, Me
3-Me	1.08, d	19.2, Me	18.6, Me	19.3, Me	19.1, Me	1.09, d	14.8, Me	17.8, Me	17.8, Me
2′-Me	2.28, s	22.5, Me	22.6, Me	22.5, Me	22.5, Me	2.30, s	22.6, Me	22.5, Me	22.5, Me
8-OMe	3.85, s	56.2, Me	56.0, Me	56.2, Me	56.3, Me	3.86, s	56.2, Me	56.9, Me	57.0, Me
4′-OMe	4.00, s	56.6, Me	56.6, Me	56.6, Me	56.6, Me	4.00, s	56.6, Me	56.6, Me	56.6, Me
1′″	4.56, q	46.6, CH	46.6, CH	52.9, CH	71.3, CH	5.03, q	71.4, CH	46.6, CH	71.3, CH
3′″	3.91, m	48.1, CH	48.1, CH	50.2, CH	63.3, CH	4.40, m	63.0, CH	48.1, CH	63.3, CH
4′″	3.68, dd	33.5, CH^eq^	33.6, CH^eq^	33.2, CH^eq^	33.5, CH^eq^	4.42, dd	34.9, CH^eq^	33.5, CH^eq^	34.7, CH^eq^
	3.05, dd	33.5, CH^ax^	33.6, CH^ax^	33.2, CH^ax^	33.5, CH^ax^	2.90, dd	34.9, CH^ax^	33.5, CH^ax^	34.7, CH^ax^
5′″		119.2, C	118.9, C	119.3, C	117.7, C		117.4, C	119.1, C	117.6, C
6′″		154.7, C	154.7, C	154.7, C	155.7, C		155.5, C	154.7, C	155.5, C
7′″	6.45, s	102.7, CH	102.6, CH	102.4, CH	102.7, CH	6.46, s	102.7, CH	102.6, CH	102.7, CH
8′″		158.6, C	158.6, C	158.6, C	158.0, C		158.0, C	158.5, C	158.0, C
9′″		118.2, C	118.3, C	118.9, C	119.2, C		119.8, C	118.3, C	119.8, C
10′″		128.7, C	128.7, C	131.0, C	127.9, C		128.1, C	128.8, C	128.2, C
1″		85.5, C	85.4, C	85.3, C	85.4, C		85.4, C	85.3, C	85.4, C
2″		92.4, C	92.4, C	92.4, C	92.5, C		92.4, C	92.5, C	92.5, C
3″	3.13, dd	52.2, CH^eq^	52.2, CH^eq^	52.3, CH^eq^	52.0, CH^eq^	3.13, dd	52.0, CH^eq^	52.2, CH^eq^	51.9, CH^eq^
	3.03, dd	52.2, CH^ax^	52.2, CH^ax^	52.3, CH^ax^	52.0, CH^ax^	3.02, dd	52.0, CH^ax^	52.2, CH^ax^	51.9, CH^ax^
4″		196.3, CO	196.3, CO	196.3, CO	196.2, CO		196.2, CO	196.2, CO	196.1, CO
5″		160.7, C	160.5, C	160.6, C	160.8, C		160.8, C	160.7, C	160.8, C
6″	7.35, d	113.9, CH	113.9, CH	113.8, CH	114.0, CH	7.34, d	114.0, CH	114.0, CH	114.0, CH
7″	7.96, d	135.6, CH	135.5, CH	135.6, CH	135.1, CH	8.32, d	135.1, CH	135.6, CH	135.2, CH
8″		124.5, C	124.5, C	125.2, C	124.6, C		124.6, C	124.5, C	124.7, C
9″		138.5, C	138.3, C	138.7, C	138.8, C		138.8, C	138.3, C	138.8, C
10″		119.5, C	119.6, C	119.5, C	119.4, C		119.5, C	119.5, C	119.4, C
1′″-Me	1.67, d	20.2, Me	20.2, Me	20.4, Me	16.5, Me	1.78, d	16.5, Me	20.3, Me	16.5, Me
3′″-Me	0.72, d	14.9, Me	14.9, Me	14.6, Me	14.8, Me	1.50, d	18.6, Me	14.6, Me	14.8, Me
2″-Me	1.84, s	18.4, Me	18.4, Me	18.4, Me	18.3, Me	1.83, s	18.3, Me	18.3, Me	18.3, Me
8′″-OMe	3.72, s	56.5, Me	56.5, Me	56.4, Me	56.7, Me	3.73, s	56.7, Me	56.5, Me	56.8, Me
5″-OMe	3.96, s	56.8, Me	56.8, Me	56.8, Me	56.8, Me	3.96, s	56.8, Me	56.8, Me	56.8, Me
2′″-NMe					49.3, Me	3.11	49.3, Me		49.5, Me

#### Cyclombandakamine A_5_ (5)

**Figure 4:**
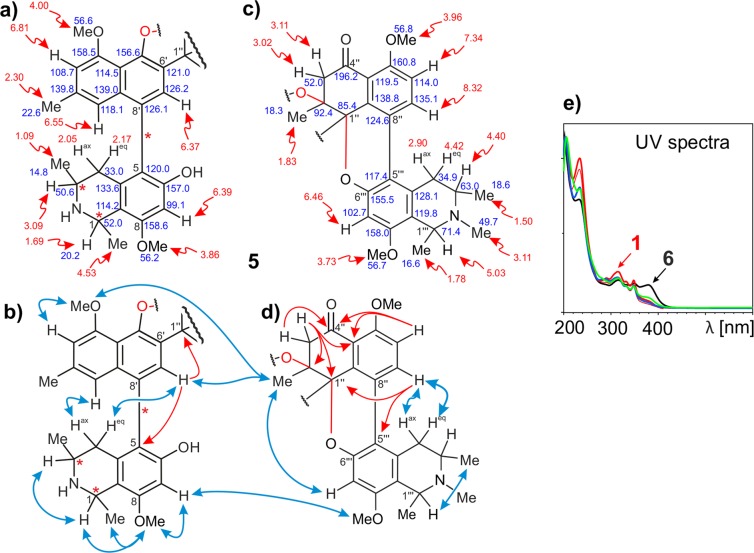
Chemical shifts (in ppm) of (**a**) the southeastern and (**c**) the northwestern halves of 5. Selected ROESY (double blue arrows) and HMBC (single red arrows) interactions in (**b**) the first and in (**d**) the second halves of cyclombandakamine A_5_ (5). (**e**) Typical UV spectra of cyclombandakamine alkaloids.

Along with compound **2**, a fifth alkaloid purified on Symmetry^®^ was obtained featuring the aforementioned characteristics of cyclized mbandakamines. High-resolution ESIMS and full NMR data analysis led to a constitution identical to that of **4** (C_49_H_52_N_2_O_8_). The main difference to **4** was found in the southeastern half, which again turned out to be *cis*-configured. This was evidenced by the ROESY interactions from H-1 (*δ*_H,C_ 4.53, 52.0) to H-3 (*δ*_H,C_ 3.09, 50.6), and their remarkable chemical shifts (see Fig. 4). The observed typical deshielded resonances of C-1′″ (*δ*_H,C_ 5.03, 71.4) and C-3′″ (*δ*_H,C_ 4.40, 63.0) may from now on be considered as characteristic of *cis*-configured and *N*-methylated (*N*-Me) tetrahydroisoquinolines located in the northwestern part of mbandakamines and cyclombandakamines. The new alkaloid **5** was named cyclombandakamine A_5_ (**5**). This dimer was the first unsymmetric naphthylisoquinoline alkaloid featuring one *N*-H and one *N*-Me tetrahydroisoquinoline subunit that were both *cis*-configured (see also Table 1).

#### Cyclombandakamine A_6_ (6)

Eluting on LC after the fraction mainly containing **5**, another better resolvable peak from the series displayed related UV (see Fig. 4e) and ECD spectra. Its molecular formula by HRESIMS corresponded to C_48_H_48_N_2_O_8_, with an IHD of 26. Combined with the presence of only three proton doublets – with *J* ≈ 6.5 Hz – in the ^1^H high-field region and a deshielded ^13^C of signal at *δ*_C_ 175.2 (C-1), its constitution suggested the presence of a dihydroisoquinoline derivative of cyclombandakamine A (**1**). The resonance of the unprotonated C-1 and the methyl singlet attached to it, 1-Me (*δ*_H,C_ 2.71, 24.9), were consistent with this assignment in the southeastern portion. Despite some minor chemical-shift variations, the rest of this molecular half was found to be identical to that of **1**, including the absolute configurations. The new dimeric alkaloid was named cyclombandakamine A_6_ (**6**).

#### Cyclombandakamine A_7_ (7)

Compound **7** required multidimensional-purification liquid chromatography to afford sufficient material for full characterization. From the data, all the common UV and ECD spectra, MS and NMR characteristics of a cyclized mbandakamine were observed at first glimpse. Its molecular formula was deduced to be C_49_H_50_N_2_O_8_, with an IHD of 26. The data analysis indicated its structural similarity to cyclombandakamine A_5_ (**5**), the main difference lying in the southeastern portion. In this molecular part, a dihydroisoquinoline subunit was identified, similar to the one found in **6**. The assignment was proven by the resonance of the sp^2^-hybridized C-1 (*δ*_C_ 175.4) in the ^13^C spectra and the presence of three methyl doublets in the high-field region of the ^1^H spectrum. Thus, the compound displayed a dihydroisoquinoline unit in the southeastern half and a relative *cis*-configured *N*-methyl tetrahydroisoquinoline sub-portion in the northwestern part. It was named cyclombandakamine A_7_ (**7**).

The isolation of such a large series of dimeric alkaloids was of high value and provided valuable insight into this unique class of natural products^16^. Even though the ‘central’ 6′,1″-*C,C*-linkage was no longer a chiral axis in these *O*-bridged dimers, an origin of **1** from **9** seemed most probable, based on our previous biosynthetic assumption on the origin of **8**^16^. The driving force for such an oxidative conversion of **9** to **1** might be the proximity of the present chromophores and the gain of stability by the reduction of the steric load at the central axis, in particular at C-1″. Moreover, the orientations of the HO-5′ and HO-6′″ functions on opposite sides of the plane formed by the C-1″-substituted naphthalene unit in mbandakamine A (**9**) were favorable for the cyclization, which should lead to *trans*-configured 5″-methoxy-2″-methyl-tetralone and, specifically, to the discovered 1″R,2″S,8″P diastereomer only. This also meant that its atropo-diastereomer mbandakamine B, with its 6′*M*,8″P biaryl axes should give the *cis*-configured cyclic diastereomer, *viz*. 1″R,2″R,8″P, exclusively. Interestingly, no such mbandakamine-B-derived cyclombandakamines have so far been found, nor any with a configuration at C-1″/C-2″ different from *R*,*S*.

The bicyclic ring system of *cis*-isoshinanolone (**11**)^7,20–22^, a natural tetralone derivative likewise occurring in Congolese *Ancistrocladus* lianas, is a characteristic structural motive in the cyclombandakamines. In the case of the dimers reported here, only the *cis*-form of this tetralone – with its OH and Me residues on the same side – would be favorable to provide, via a probably enzymatic cyclization, the diastereomer found in the isolated dimeric alkaloids. This formation of the cyclic compounds may be supported by the co-occurrence of **1** and **2** together with their open-chain analogs **9** and **10** in *A. ealaensis*.

The long-known sensitivity of *cis*-configured naphthylisoquinolines, if devoid of an *N*-methyl group (*N*-H), to oxidation^23^ showed that their successful isolation here was an indication of mild workup procedures. Since cyclombandakamines were likely to be oxidation products from ‘open-chain’ mbandakamine-like alkaloids^16^, the discovery of such dimers with intact *N*-H *cis*-relative configurations were a further argument against an imaginable spontaneous, non-enzymatic oxidative formation, but rather hinted at a strictly regulated enzymatic process for their biosynthesis in *A. ealaensis*, evidencing that the compounds were true natural scaffolds and no artificial follow-up products from their analogs.

The similarity of the ECD spectra, despite the different variations in the isoquinoline moieties, further proved the reported^16^ chiroptical dominance of the twisted dihydrofuran-tetralone-pyran chromophore towards the other present elements of chirality. A remarkable structural feature of these dimers is the co-occurrence of bridged and non-bridged biaryl axes, which was quite unique among dimeric naphthylisoquinoline alkaloids. The isolation of this series of novel compounds indicated once more the impressive biosynthetic potential of the tropical liana *A. ealaensis*.

### Biological Evaluations

In contrast to the corresponding open-chain mbandakamines, the cyclombandakamines exhibited *in vitro* activities in the low micromolar range against the chloroquine sensitive (NF54) and resistant (K1) strains of *P. falciparum*, the causative agent of malaria. Cyclombandakamine A (**1**), 1-*epi*-cyclombandakamine A (**2**), cyclombandakamine A_5_ (**5**), and cyclombandakamine A_6_ (**6**) showed IC_50_ values of 0.664 (NF54) and 0.268 µM (K1), 0.300 (NF54) and 0.148 µM (K1), n.d. (NF54) and 0.350 µM (K1), and 0.666 (NF54) and 0.266 µM (K1), respectively (Table 2). These four alkaloids featured the structural variations observed within this series of dimers. Interestingly, they were found to be more active against the chloroquine-resistant strain K1 than against the sensitive strain NF54. The lower activities of **1** and **2** compared to their open-chain analogs suggested that the highly condensed polycyclic system or the reduced number of free phenolic OH groups might be decisive for the reduced anti-infective potential of cyclombandakamines. Against *Trypanosoma brucei rhodesiense*, *T. cruzi*, and *Leishmania donovani*, very low (in the case of **1** and **2** for *T. b. rhodesiense*) or virtually no activities were found for any of the evaluated cyclombandakamines. This finding confirms the distinct structure dependency – and thus high specificity – of the antiplasmodial activities of the dimeric naphthylisoquinoline alkaloids presented in Table 2.

**Table 2:** Biological evaluations of **1**, **2**, **5**, and **6** against *Plasmodium falciparum* (strains: NF54 and K1), *Trypanosoma brucei rhodesiense*, *T. cruzi*, and *Leishmania donovani*.

Compounds	T. brucei rhodesiense	T. cruzi	L. donovani ax. am.	P. falciparum NF54/K1	Selectivity index to NF54/K1
Standard	0.01^[1]^	3.56^[2]^	0.43^[3]^	0.01^[4]^/0.36^[4]^	n.d.
**1**	12.38	63.80	39.55	0.664/**0.268**	67/166
**2**	6.36	58.31	>127.73	0.300/**0.148**	>61/117
**5**	—	—	—	−/0.350	−/52
**6**	50.22	95.79	>127.73	0.666/0.266	138/**346**
**9**	1.8	16.8	>100	0.06/−	277/−
**10**	1.8	41.8	>100	0.05/0.06	337/249

The cytotoxicities of the compounds were determined against rat skeletal myoblasts (L6 cells). [1] Melarsoprol. [2] Benznidazole. [3] Miltefosine. [4] Chloroquine. All IC_50_ values are given in µM. n.d.: not determined. Selectivity index determined for *P. falciparum*.

In conclusion, the cyclized mbandakamines displayed a weaker anti-infective potential as compared to the related open-chain alkaloids. While the role of these compounds for the plants and the details of their biosynthetic formation need to be further explored, their charm lies in their intriguing, unprecedented structures.

## Experimental Section

### General experimental procedures

A Jasco^®^ LC-2000Plus Series System (Gross-Umstadt, Germany) was used for LC-DAD analysis. LC-MS measurements were performed on an Agilent 1100 Series System, equipped with binary high-pressure mixing pumps, with an autosampler, a degasser module, a 1100 series photodiode array (PDA) detector (Agilent Technology, Germany), and an ion-trap mass spectrometer with an electrospray ionization interface (Bruker Daltonics, Bremen, Germany). A Bruker Daltonics microTOF spectrometer focus was used for high-resolution electrospray mass spectrometry (positive mode). NMR analyses were acquired on AMX 400 and DMX 600 Bruker spectrometers with cryoprobes. The offline ECD and ORD spectra were recorded on a Jasco J-715 spectropolarimeter, and evaluated with SpecDis_164^24,25^. A Shimadzu UV-1800 spectrophotometer was used to perform offline UV measurements in triplicate. A Jasco P-1020-polarimeter operating with a sodium light source (λ = 589 nm) was used for the measurement of optical rotations. The mechanical shaker operating at the frequency of 160 RPM (rotations per minutes) was from Bottmingen (Switzerland).

### Plant material

Leaf material of *Ancistrocladus ealaensis*^5,17^ was collected in Mbandaka, at the Botanical Garden of Eala (Democratic Republic of the Congo), in August 2008 by one of us (V.M.) and in August 2015 by Mr. B.K. Lombe (GPS coordinates 00°03.605N, 018°18.886E). The material of 2015 was additionally authenticated by LC-DAD-MS to contain the same metabolites as the one of 2008. Voucher specimens are available at the Herbarium Bringmann at the Institute of Organic Chemistry, University of Würzburg (nos. 43 and 57).

### Extraction and isolation

The air-dried leaf material (800 g) was macerated for 24 h in MeOH, with mechanical shaking (160 RPM), followed by further macerations until exhaustion. The 24-h macerates were mixed, filtered, and evaporated to give a viscous solution. The methanolic extract was dissolved in water to permit precipitation of chlorophyll. The partition of the aqueous solution was cleared from residual undesired material using *n*-hexane, before being exhaustively extracted with CH_2_Cl_2_. The organic layer was evaporated to dryness to obtain fraction A. This dichloromethane phase collected under neutral conditions (pH 7) was likewise dissolved in MeOH and submitted to reversed-phase chromatography, which led to the more slowly eluting subfractions A_83_-A_96_. The elution was performed from 0 to 50% H_2_O in MeCN. Subfractions enriched with cyclombandakamines, but still present as hardly resolvable mixtures, were subjected to a multi-dimensional isolation procedure using semi-preparative HPLC to afford the alkaloids in a pure form.

### Isolation and semi-preparative HPLC conditions

For this purpose, four gradient elution conditions were used:

#### System 1

Isolation was performed on a Symmetry^®^ Prep-C_18_ column (Waters, 300 × 19 mm, 7 μm), using A (H_2_O, 0.05% TFA) and B (MeCN, 0.05% TFA), with a flow rate of 10 mL min^−1^. The gradient details were as described for the isolation of the ealapasamines A-C^17^ to afford the enriched fractions.

#### System 2

Purification was performed on a Discovery^®^ HS F5–10 column (Supelco^®^, 250 × 21.2 mm, 10 μm), using A (H_2_O, 0.05% TFA) and B (MeCN, 0.05% TFA), with a flow rate of 13 mL min^−1^. The stationary phase was pentafluorophenyl-bonded high-speed silica, which offers different separations processes on C_18_, characterized by stronger retention of alkaloids. The gradient used in this case was: 0–20 min: 35–55% of B, 25 min: 60% of B, 33 min: 70% of B, 48 min: 75% of B, 50 min: 100% of B, yielding 12.3 mg of cyclombandakamine A (**1**) and 6.1 mg of 1-*epi*-cyclombandakamine A (**2**), eluting faster than **9** and **10**.

#### System 3

Further purification was achieved using a Chromolith^®^ SemiPrep RP-18e column (100 × 10 mm), and a mobile phase made of A and C (MeOH, 0.05% TFA), at the flow rate of 14.2 mL min^−1^. The gradient used was: 0–6 min: 5% of C, 11–15 min: 30% of C, 15.1–25 min: 33% of C, 26 min: 40% of C, 27 min: 45% of C, 28 min: 100% of C, to yield 5.5 mg of cyclombandakamine A_5_ (**5**), 11.3 mg of cyclombandakamine A_6_ (**6**), 2.5 mg of cyclombandakamine A_7_ (**7**), along with other related compounds, which were not fully characterized.

#### System 4

Further purification was performed on a Chromolith^®^ SemiPrep RP-18e column using the mobile solvent systems A and C (MeOH, 0.05% TFA), at the flow rate of 10 mL min^−1^. The gradient used was: 0–2 min: 5% of C, 13–28 min: 30–50% of C, 29 min: 100% of C, yielding 1.5 mg of cyclombandakamine A_3_ (**3**), 4.6 mg of cyclombandakamine A_4_ (**4**), along with 0.5 mg of cyclombandakamine A_5_ (**5**).

#### Cyclombandakamine A (1)

White amorphous powder; ${[\alpha ]}_{D}^{23}$− 22 (*c* = 0.1, MeOH); UV (MeOH) λ_max_ (log ε) = 201 (1.88), 216 (1.30), 233 (1.52), 257 (0.50), 277 (0.28), 288 (0.28), 295 (0.28), 315 (0.34), 330 (0.24), 340 (0.19), 385 (0.03) nm; ECD (MeOH, *c* 0.3) λ_max_ (log ε in cm^2^ mol^−1^) 200 (+8.48), 207 (‒5.19), 216 (+7.68), 219 (+6.55), 233 (+32.09), 247 (‒21.84), 287 (+3.07), 304 (‒10.12), 325 (+4.15), 347 (‒0.26), 360 (+1.82), 400 (+0.04) nm; ORD (MeOH, *c* 0.3) λ_max_ (log ε in cm^2^ mol^−1^) 201 (+6.27), 211 (‒9.54), 217 (‒4.22), 224 (‒8.70), 239 (+40.49), 269 (‒7.00), 295 (+6.09), 315 (‒9.28), 340 (+0.07), 353 (‒1.91), 386 (+0.65), 430 (+0.22) nm; ^1^H NMR and ^13^C NMR data: see Table 1 (for further details, see SI); HRESIMS *m/z* 783.36451 [M + H]^+^ (calcd for C_48_H_51_N_2_O_8_, 783.36399).

#### 1-epi-Cyclombandakamine A (2)

White amorphous powder; ${[\alpha ]}_{D}^{23}$ − 21 (*c* 0.1, MeOH); UV (MeOH) λ_max_ (log ε) 201 (1.88), 216 (1.32), 233 (1.49), 257 (0.50), 277 (0.28), 288 (0.28), 295 (0.28), 315 (0.34), 330 (0.24), 340 (0.19), 385 (0.03) nm; ECD (MeOH, *c* 0.3) λ_max_ (log ε in cm^2^ mol^−1^) 200 (+8.48), 206 (‒5.20), 216 (+7.68), 218 (+6.54), 233 (+32.10), 247 (‒21.80), 287 (+3.09), 304 (‒11.10), 325 (+4.15), 347 (‒0.26), 360 (+1.82), 400 (+0.01) nm; ORD (MeOH, *c* 0.3) λ_max_ (log ε in cm^2^ mol^−1^) 201 (+6.30), 211 (‒9.50), 218 (‒4.22), 224 (‒8.70), 239 (+39.49), 269 (‒7.05), 295 (+6.09), 315 (‒9.28), 340 (+0.08), 353 (‒1.91), 386 (+0.65), 400 (+0.30) nm; ^13^C NMR data in Table 1 (for further details, see SI); HRESIMS *m/z* 783.36229 [M + H]^+^ (calcd for C_48_H_51_N_2_O_8_, 783.36451). It can also be addressed as cyclombandakamine C.

#### Cyclombandakamine A_3_ (3)

White amorphous powder; ${[\alpha ]}_{D}^{23}$ − 13 (*c* 0.04, MeOH); UV (MeOH) λ_max_ (log ε) 201 (1.88), 216 (1.30), 233 (1.52), 257 (0.50), 277 (0.28), 288 (0.285), 295 (0.28), 315 (0.34), 330 (0.24), 340 (0.19), 390 (0.02) nm; ECD (MeOH, *c* 0.1) λ_max_ (log ε in cm^2^ mol^−1^) 200 (+2.23), 210 (+0.41), 212 (+0.49), 220 (‒0.44), 234 (+4.66), 251 (‒2.99), 286 (+0.29), 304 (‒1.57), 328 (+0.43), 347 (‒0.03), 354 (+0.20), 365 (+0.27), 400 (‒0.01) nm; ORD (MeOH, *c* 0.1) λ_max_ (log ε in cm^2^ mol^−1^) 201 (+3.13), 211 (‒4.94), 217 (‒2.12), 224 (‒4.44), 240 (+19.23), 269 (‒3.45), 294 (+3.05), 314 (‒5.00), 340 (+0.02), 355 (‒0.89), 388 (+0.35), 430 (+0.10) nm; ^13^C NMR data in Table 1 (for further details, see SI); HRESIMS *m/z* 783.36203 [M + H]^+^ (calcd for C_48_H_51_N_2_O_8_, 783.36399).

#### Cyclombandakamine A_4_ (4)

White amorphous powder; ${[\alpha ]}_{D}^{23}$ − 14 (*c* 0.04, MeOH); UV (MeOH) λ_max_ (log ε) 205 (1.11), 217 (0.70), 230 (1.01), 257 (0.24), 262 (0.25), 297 (0.14), 315 (0.15), 322 (0.21), 329 (0.19), 338 (0.25), 344 (0.29) nm; ECD (MeOH, *c* 0.1) λ_max_ (log ε in cm^2^ mol^−1^) 200 (+2.25), 205 (+0.72), 213 (‒2.74), 220 (+2.16), 232 (+7.16), 248 (‒5.94), 287 (+0.05), 304 (‒3.18), 325 (+0.68), 347 (‒0.59), 360 (+0.34), 385 (‒0.12), 400 (‒0.31) nm; ORD (MeOH, *c* 0.1) λ_max_ (log ε in cm^2^ mol^−1^) 202 (‒0.35), 209 (‒1.79), 217 (‒0.13), 224 (‒0.92), 240 (+10.15), 269 (‒1.31), 295 (+1.59), 316 (‒2.57), 341 (‒0.13), 353 (‒0.94), 382 (+0.09), 393 (+0.14), 460 (‒0.23) nm; ^13^C NMR data in Table 1 (for further details, see SI); HRESIMS *m/z* 813.37506 [M + H + O]^+^ (calcd for C_49_H_53_N_2_O_9_, 813.37511). The molecular formula is C_49_H_52_N_2_O_8_.

#### Cyclombandakamine A_5_ (5)

White amorphous powder; ${[\alpha ]}_{D}^{23}$ + 18 (*c* 0.09, MeOH); UV (MeOH) λ_max_ (log ε) 201 (1.88), 216 (1.32), 233 (1.49), 257 (0.50), 277 (0.28), 288 (0.28), 295 (0.28), 315 (0.34), 330 (0.24), 340 (0.19), 385 (0.03) nm; ECD (MeOH, *c* 0.2) λ_max_ (log ε in cm^2^ mol^−1^) 202 (+7.25), 215 (+11.11), 219 (+10.24), 231 (+20.50), 247 (‒19.08), 287 (+0.97), 304 (‒10.16), 325 (+4.11), 333 (+2.13), 347 (‒0.75), 361 (+2.25), 400 (+0.05) nm; ORD (MeOH, *c* 0.2) λ_max_ (log ε in cm^2^ mol^−1^) 198 (‒5.03), 208 (‒3.00), 218 (+2.01), 223 (+1.46), 239 (+31.68), 269 (‒4.94), 295 (+5.11), 315 (‒9.53), 341 (‒0.16), 353 (‒2.86), 373 (+0.22), 387 (+0.56), 437 (‒0.46) nm; ^1^H NMR and ^13^C NMR data in Table 1 (further details see SI); HRESIMS *m/z* 813.37280 [M + H + O]^+^ (calcd for C_49_H_53_N_2_O_9_, 813.37511). The molecular formula was C_49_H_52_N_2_O_8_.

#### Cyclombandakamine A_6_ (6)

Yellowish amorphous powder; ${[\alpha ]}_{D}^{23}$ − 20 (*c* 0.09, MeOH); UV (MeOH) λ_max_ (log ε) 201 (1.12), 224 (0.72), 232 (0.73), 263 (0.24), 296 (0.15), 315 (0.19), 329 (0.16), 340 (0.15), 350 (0.17), 361 (0.14), 380 (0.16), 400 (0.06), 420 (0.01) nm; ECD (MeOH, *c* 0.09) λ_max_ (log ε in cm^2^ mol^−1^) 200 (+5.60), 208 (+2.19), 212 (+2.98), 220 (+1.58), 231 (+5.60), 245 (‒4.76), 280 (‒0.90), 290 (+0.28), 304 (‒1.65), 326 (+1.64), 355 (‒1.49), 389 (+0.09), 400 (‒0.09) nm; ORD (MeOH, *c* 0.09) λ_max_ (log ε in cm^2^ mol^−1^) 200 (‒1.96), 204 (‒0.15), 209 (‒0.89), 218 (+0.73), 225 (‒0.61), 238 (+7.76), 254 (‒0.09), 272 (‒0.35), 283 (‒0.90), 296 (+0.69), 315 (‒1.84), 342 (1.55), 371 (‒0.69), 397 (‒0.09), 443 (‒0.03) nm; ^13^C NMR data in Table 1 (for further details, see SI); HRESIMS *m/z* 781.34886 [M + H]^+^ (calcd for C_48_H_49_N_2_O_8_, 781.348892).

#### Cyclombandakamine A_7_ (**7**)

Yellowish amorphous powder; ${[\alpha ]}_{D}^{23}$ − 13 (*c* 0.04, MeOH); UV (MeOH) λ_max_ (log ε) 201 (1.88), 216 (1.30), 233 (1.52), 257 (0.50), 277 (0.28), 288 (0.285), 295 (0.28), 315 (0.34), 330 (0.24), 340 (0.19), 390 (0.02) nm; ECD (MeOH, *c* 0.1) λ_max_ (log ε in cm^2^ mol^−1^) 200 (+2.23), 210 (+0.41), 212 (+0.49), 220 (‒0.44), 234 (+4.66), 251 (‒2.99), 286 (+0.29), 304 (‒1.57), 328 (+0.43), 347 (‒0.03), 354 (+0.20), 365 (+0.27), 400 (‒0.01) nm; ORD (MeOH, *c* 0.1) λ_max_ (log ε in cm^2^ mol^−1^) 201 (+3.13), 211 (‒4.94), 217 (‒2.12), 224 (‒4.44), 240 (+19.23), 269 (‒3.45), 294 (+3.05), 314 (‒5.00), 340 (+0.02), 355 (‒0.89), 388 (+0.35), 430 (+0.10) nm; ^13^C NMR data in Table 1 (for further details, see SI); HRESIMS *m/z* 811.35652 [M + H + O]^+^ (calcd for C_49_H_51_N_2_O_9_, 811.35891). The molecular formula [M]^+^ is C_49_H_50_N_2_O_8_.

### Oxidative degradation

The ruthenium (VIII)-mediated periodate degradation of the cyclombandakamines **1**–**7**, the Mosher-type derivatization of the resulting amino acids using MeOH/HCl and *R*-α-methoxy-α-trifluoromethylacetyl chloride (*R*-MTPA-Cl, prepared from *S*-MTPA), and the subsequent GC-MSD analysis were carried out as described earlier^19^ on a GCMS-QP2010 Systems **(**Shimadzu, Germany).

### Computational analysis

The DFT structural geometry optimizations were done using the B3LYP-D3/def2-TZVP method, with ORCA^17,18,26,27^. The TDDFT calculations of the ECD spectra were performed as described previously^16^ using ORCA and Gausian09^26–28^. TDDFT was used for the simulation of the UV and ECD spectral data^27^, and the calculated spectra were compared with the experimental ones using SpecDis^24,25^.

### Biological evaluation

The *in vitro* antiprotozoal tests were performed on the NF54 (chloroquine-sensitive) and K1 (chloroquine- and pyrimethamine-resistant) strains of *Plasmodium falciparum*, STIB 900 strain of *Trypanosoma brucei rhodesiense* (trypomastigotes), Tulahuen C4 strain of *Trypanosoma cruzi* (amastigotes), and MHOM-ET-67/L82 strain of *Leishmania donovani* (axenically grown amastigotes) in duplicate. The tested compounds were evaluated together with **9** and **10** for a better comparison of IC_50_ values. The cytotoxicity on mammalian host cells (rat skeletal myoblast L6 cells) were determined according to established protocols^29^.

## Supplementary information

Supplementary Information: An Unusually Broad Series of Seven Cyclombandakamines, Bridged Dimeric Naphthylisoquinoline Alkaloids from the Congolese Liana Ancistrocladus ealaensis
