# Supplementary material for: An Unusually Broad Series of Seven Cyclombandakamines, Bridged Dimeric Naphthylisoquinoline Alkaloids from the Congolese Liana Ancistrocladus ealaensis
Source: Sci Rep. 2019 Jul 8;9:9812. doi: 10.1038/s41598-019-46336-z (PMC6614417; doi:10.1038/s41598-019-46336-z)
Supplement: Supplementary file 1 — Supplementary Information: An Unusually Broad Series of Seven Cyclombandakamines, Bridged Dimeric Naphthylisoquinoline Alkaloids from the Congolese Liana Ancistrocladus ealaensis [file 41598_2019_46336_MOESM1_ESM.pdf]

Supporting Information (SI)

An Unusually Broad Series of Seven Cyclombandakamines, Bridged Dimeric Naphthylisoquinoline Alkaloids from the Congolese Liana *Ancistrocladus ealaensis*

Dieudonné Tshitenge Tshitenge[1,2], Torsten Bruhn[3], Doris Feineis[1], Virima Mudogo[4], Marcel Kaiser[5,6], Reto Brun[5,6], and Gerhard Bringmann[1,*]

[1] Institute of Organic Chemistry, University of Würzburg, Am Hubland, D-97074 Würzburg, Germany

[2] Faculty of Pharmaceutical Sciences, University of Kinshasa, B.P. 212 Kinshasa XI, Democratic Republic of the Congo

[3] Federal Institute for Risk Assessment, Max-Dohrn-Straße 8-10, D-10589 Berlin, Germany.

[4] Faculté des Sciences, Université de Kinshasa, B.P. 202, Kinshasa XI, Democratic Republic of the Congo

[5] Swiss Tropical and Public Health Institute, Socinstrasse 57, CH-4002 Basel, Switzerland

[6] University of Basel, Petersplatz 1, CH-4003 Basel, Switzerland

*To whom correspondence and requests for materials should be addressed to G.B.

Tel: +49 931 318 5323. Fax: +49 931 318 4755.

E-mail: bringman@chemie.uni-wuerzburg.de.

Table of Contents

Table S1. Detailed 2D NMR data of cyclocyclombandakamine A (**1**) in MeOD......................................7

Table S2. Detailed 2D NMR data of 1-*epi*-cyclombandakamine A (**2**) in MeOD....................................9

Table S3. Detailed 2D NMR data of cyclocyclombandakamine A_3 (**3**) in MeOD..................................11

Table S4. Detailed 2D NMR data of cyclombandakamine A_4 (**4**) in MeOD..13

Table S5. Detailed 2D NMR data of cyclombandakamine A_5 (**5**) in MeOD..15

Table S6. Detailed 2D NMR data of cyclombandakamine A_6 (**6**) in MeOD..17

Table S7. Detailed 2D NMR data of cyclombandakamine A_7 (**7**) in MeOD..19

Figures

Figure S8a. Overall ^{1}H NMR spectrum of cyclombandakamine A (**1**) in methanol-d_4..........................21

Figure S8b,c. Parts of the ^{1}H NMR spectrum of cyclombandakamine A (**1**) in methanol-d_4…………..22

Figure S9a. Overall ^{13}C NMR spectrum of cyclombandakamine A (**1**) in methanol-d_4.........................23

Figure S9b,c. Parts of the ^{13}C NMR spectrum of cyclombandakamine A (**1**) in methanol-d_4………….24

Figure S10. DEPT-135 NMR spectrum of cyclombandakamine A (**1**) in methanol-d_4..........................25

Figure S11. HSQC spectrum of cyclombandakamine A (**1**) in methanol-d_4..26

Figure S12. HMBC spectrum of cyclombandakamine A (**1**) in methanol-d_4..27

Figure S13. COSY spectrum of cyclombandakamine A (**1**) in methanol-d_4...28

Figure S14. TOCSY spectrum of cyclombandakamine A (**1**) in methanol-d_4..29

Figure S15a. ROESY spectrum of cyclombandakamine A (**1**) in methanol-d_4......................................30

Figure S15b. Parts of the ROESY spectrum of cyclombandakamine A (**1**) in methanol-d_4...................31

Figure S16. HRESIMS of cyclombandakamine A (**1**) in acetonitrile...32

Figure S17. MALDI analysis: profile of cyclombandakamine A (**1**)..33

Figure S18. Oxidative degradation results of cyclombandakamine A (**1**)..34

Figure S19. ECD spectrum of cyclombandakamine A (**1**) in methanol..35

Figure S20. Offline UV spectrum of cyclombandakamine A (**1**) in methanol..35

Figure S21a. Overall ^{1}H NMR spectrum of 1-*epi*-cyclombandakamine A (**2**) in methanol-d_4..............36

Figure S21b,c. Parts of the ^{1}H NMR spectrum of 1-*epi*-cyclombandakamine A (**2**) in methanol-d_4…..37

Figure S22a. Overall ^{13}C NMR spectrum of 1-*epi*-cyclombandakamine *A* (**2**) in methanol-d_4..............38

Figure S22b,c. Parts of the ^{13}C NMR spectrum of 1-*epi*-cyclombandakamine A (**2**) in methanol-d_4.....39

Figure S23. DEPT NMR spectrum of 1-*epi*-cyclombandakamineA (**2**) in methanol-d_4.........................40

Figure S24. HSQC spectrum of 1-*epi*-cyclombandakamine A (**2**) in methanol-d_4................................41

Figure S25. HMBC spectrum of 1-*epi*-cyclombandakamine A (**2**) in methanol-d_4................................42

Figure S26. COSY spectrum of 1-*epi*-cyclombandakamine A (**2**) in methanol-d_4.................................43

Figure S27a. ROESY spectrum of 1-*epi*-cyclombandakamine A (**2**) in methanol-d_4.............................44

Figure S27b,c. Parts of the ROESY spectrum of 1-*epi*-cyclombandakamine A (**2**) in methanol-d_4.......45

Figure S28. HRESIMS spectrum 1-*epi*-cyclombandakamine A (**2**) in methanol...................................46

Figure S29. Oxidative degradation results of 1-*epi*-cyclombandakamine A (**2**).....................................47

Figure S30. ECD spectrum of 1-*epi*-cyclombandakamine A (**2**) in methanol compared to
the one of cyclombandakamine A_4 **(4)** in methanol-d_4…………………………………...…48

Figure 31. Offline UV spectrum of 1-*epi*-cyclombandakamine A (**2**) in methanol................................48

Figure S32a. Overall ^{1}H NMR spectrum of cyclombandakamine A_3 **(3)** in methanol-d_4.......................49

Figure S32b,c. Parts of the ^{1}H NMR spectrum of cyclombandakamine A_3 **(3)** in methanol-d_4.............50

Figure S33. ^{13}C NMR spectrum of cyclombandakamine A_3 **(3)** in methanol-d_4.....................................51

Figure S34. Overall HSQC spectrum of cyclombandakamine A_3 **(3)** in methanol-d_4............................52

Figure S34b. Aliphatic part of the HSQC spectrum of cyclombandakamine A_3 **(3)** in methanol-d_4.......53

Figure S35. HMBC spectrum of cyclombandakamine A_3 **(3)** in methanol-d_4..54

Figure S36a. COSY spectrum of cyclombandakamine A_3 **(3)** in methanol-d_4.......................................55

Figure S36b. Aliphatic part of the COSY spectrum of cyclombandakamine A_3 **(3)** in methanol-d_4.......56

Figure S37. ROESY spectrum of cyclombandakamine A_3 **(3)** in methanol-d_4.......................................57

Figure S38. HRESIMS spectrum of cyclombandakamine A_3 (**3**)..58

Figure S39. MALDI analysis: profile of cyclombandakamine A_3 (**3**)..59

Figure S40. Oxidative degradation products of cyclombandakamine A_3 (**3**)..60

Figure S41. ECD spectrum of cyclombandakamine A_3 (**3**) in methanol, compared to the one of **4**........61

Figure S42. Offline UV spectrum of cyclombandakamine A_3 (**3**) in methanol……………………......61

Figure S43a. Overall ^{1}H NMR spectrum of cyclombandakamine A_4 (**4**) in methanol-d_4.......................62

Figure S43b,c. Parts of the ^{1}H NMR spectrum of cyclombandakamine A_4 (**4**) in methanol-d_4.............63

Figure S44a. ^{13}C NMR spectrum of cyclombandakamine A_4 **(4)** in methanol-d_4...................................64

Figure S44b,c. ^{13}C NMR spectrum of cyclombandakamine A_4 **(4)** in methanol-d_4................................65

Figure S45. DEPT-135 NMR spectrum of cyclombandakamine A_4 **(4)** in methanol-d_4..........................66

Figure S46. Overall HSQC spectrum of cyclombandakamine A_4 **(4)** in methanol-d_4............................67

Figure S47. HMBC spectrum of cyclombandakamine A_4 **(4)** in methanol-d_4..68

Figure S48a. Overall COSY spectrum of cyclombandakamine A_4 **(4)** in methanol-d_4...........................69

Figure S48b. Part of the COSY spectrum of cyclombandakamine A_4 **(4)** in methanol-d_4......................70

Figure S49a. ROESY spectrum of cyclombandakamine A_4 **(4)** in methanol-d_4.....................................71

Figure S49b. Part of ROESY spectrum of cyclombandakamine A_4 **(4)** in methanol-d_4.........................72

Figure S50. HRESIMS spectrum of cyclombandakamine A_4 **(4)** methanol...73

Figure S51. MALDI analysis: profile of cyclombandakamine A_4 (**4**)...74

Figure S52. Oxidative degradation results of cyclombandakamine A_4 (**4**) (very diluted sample)...........75

Figure S53. ECD spectrum of cyclombandakamine A_4 (**4**) in methanol..76

Figure S54. Offline UV spectrum of cyclombandakamine A_4 (**4**) in methanol......................................76

Figure S55a. Overall ^{1}H NMR spectrum of cyclombandakamine A_5 (**5**) in methanol-d_4.......................77

Figure S55b,c. Parts of the ^{1}H NMR spectrum of cyclombandakamine A_5 (**5**) in methanol-d_4.............78

Figure S56. ^{13}C NMR spectrum of cyclombandakamine A_5 **(5)** in methanol-d_4.....................................79

Figure S57. DEPT-135 NMR spectrum of cyclombandakamine A_5 **(5)** in methanol-d_4..........................80

Figure S58a. Overall HSQC spectrum of cyclombandakamine A_5 **(5)** in methanol-d_4..........................81

Figure S58b. Part of the HSQC spectrum of cyclombandakamine A_5 **(5)** in methanol-d_4......................82

Figure S59a. HMBC spectrum of cyclombandakamine A_5 **(5)** in methanol-d_4......................................83

Figure S59b. HMBC spectrum of cyclombandakamine A_5 **(5)** in methanol-d_4.......................................84

Figure S60. Overall COSY spectrum of cyclombandakamine A_5 **(5)** in methanol-d_4............................85

Figure S61. TOCSY spectrum of cyclombandakamine A_5 **(5)** in methanol-d_4.......................................86

Figure S62a. ROESY spectrum of cyclombandakamine A_5 **(5)** in methanol-d_4......................................87

Figure S62b. Aliphatic part of the ROESY spectrum of **5** in methanol-d_4...88

Figure S63. HRESIMS spectrum of cyclombandakamine A_5 **(5)** methanol..89

Figure S64. MALDI analysis: profile of cyclombandakamine A_5 (**5**)...90

Figure S65. Oxidative degradation results of cyclombandakamine A_5 (**5**) (very diluted sample)...........91

Figure S66. ECD spectrum of cyclombandakamine A_5 (**5**) in methanol..92

Figure S67. Offline UV spectrum of cyclombandakamine A_5 (**5**) in methanol.......................................92

Figure S68a. Overall ^{1}H NMR spectrum of cyclombandakamine A_6 (**6**) in methanol-d_4........................93

Figure S68a,c. Parts of the ^{1}H NMR spectrum of cyclombandakamine A_6 (**6**) in methanol-d_4..............94

Figure S69. ^{13}C NMR spectrum of cyclombandakamine A_6 (**6**) in methanol-d_4.....................................95

Figure S70. DEPT-135 NMR spectrum of cyclombandakamine A_6 (**6**) in methanol-d_4..........................96

Figure S71. Overall HSQC spectrum of cyclombandakamine A_6 (**6**) in methanol-d_4............................97

Figure S72. HMBC spectrum of cyclombandakamine A_6 (**6**) in methanol-d_4..98

Figure S73. Overall COSY spectrum of cyclombandakamine A_6 (**6**) in methanol-d_4…….....................99

Figure S74. ROESY spectrum of cyclombandakamine A_6 (**6**) in methanol-d_4....................................100

Figure S75. Aliphatic part of the ROESY spectrum of cyclombandakamine A_6 (**6**) in methanol-d_4.....101

Figure S76. HRESIMS spectrum of cyclombandakamine A_6 (**6**) methanol...102

Figure S77. MALDI analysis: profile of cyclombandakamine A_6 (**6**)..103

Figure S78. Oxidative degradation results of cyclombandakamine A_6 (**6**) (very diluted sample)…......104

Figure S79. ECD spectrum of cyclombandakamine A_6 (**6**) in methanol...105

Figure S80. Offline UV spectrum of cyclombandakamine A_6 (**6**) in methanol....................................105

Figure S81a. Overall ^{1}H NMR spectrum of cyclombandakamine A_7 (**7**) in methanol-d_4......................106

Figure S81b,c. Parts of the ^{1}H NMR spectrum of cyclombandakamine A_7 (**7**) in methanol-d_4............107

Figure S82. ^{13}C NMR spectrum of cyclombandakamine A_7 (**7**) in methanol-d_4...................................108

Figure S83a. Overall HSQC spectrum of cyclombandakamine A_7 (**7**) in methanol-d_4.........................109

Figure S83b. Part of the HSQC spectrum of cyclombandakamine A_7 (**7**) in methanol-d_4...................110

Figure S84. HMBC spectrum of cyclombandakamine A_7 (**7**) in methanol-d_4......................................111

Figure S85a. ROESY spectrum of cyclombandakamine A_7 (**7**) in methanol-d_4...................................112

Figure S85b. Aliphatic part of ROESY spectrum of cyclombandakamine A_7 (**7**) in methanol-d_4……113

Figure S86. HRESIMS spectrum of cyclombandakamine A_7 (**7**) methanol...114

Figure S87. Oxidative degradation results of cyclombandakamine A_7 (**7**) (very diluted sample).........115

Figure S89. ECD spectrum of cyclombandakamine A_7 (**7**) in methanol...116

Figure S90. Offline UV spectrum of cyclombandakamine A_7 (**7**) in methanol....................................116

Table S1. Detailed NMR data of cyclombandakamine A (**1**) in methanol-d_4 (δ in ppm, J in Hz).

		Cyclombandakamine A (**1**)			
Position	δ_H (J in Hz)	HSQC	HMBC	COSY	ROESY
1	4.68, q (6.7)	49.7, CH	8, 9, 10, 1-Me	1-Me	8-OMe
3	3.52, m	44.9, CH	4, 3-Me	4_{eq},3-Me	1-Me, 4'''$_{ax}$
4	2.35, dd (17.8,4.8)	32.7, CH_{eq}	5, 9, 10	3, 4_{ax}	7', 3-Me
	1.94, dd	32.7, CH_{ax}	3, 9, 10, 3-Me	3, 4eq	1', 3-Me
5		119.7, C			
6		157.5, C			
7	6.39, s	98.6, CH	1, 5, 6, 8, 9		8-OMe,**1'''**,**3'''-Me**,**8'''-OMe**
8		157.5, C			
9		113.9, C			
10		133.0, C			
1'	6.52, s	118.0, CH	3', 10', 8', 9'		4_{ax}, 2'-Me
2'		139.6, C			
3'	6.79, d (0.9)	108.7, CH	1', 2', 4', 10'		2'-Me, 4'-OMe
4'		158.5, C			
5'		156.4, C			
6'		121.1, C			
7'	6.35, s	126.0, CH	5, 5', 9', 10', 1''		4_{eq}, **3''**, 3'''-Me, **7'''**, **4'''$_{ax}$**
8'		126.6, C			
9'		138.7, C			
10'		114.3, C			
1-Me	1.50, d (6.7)	18.8, Me	1, 9	1	3, 8-OMe
3-Me	1.08, d (6.4)	19.2, Me	3, 4	3	4_{eq}, 4_{ax}
2'-Me	2.28, s	22.5, Me	1', 2', 3'		1', 3'
8-OMe	3.85, s	56.2, Me	8		1, 7, 1-Me
4'-OMe	4.00, s	56.6, Me	4'		3', 2''-Me
1''		85.5, C			
2''		92.4, C			
3''	3.13, d (14.9)	52.2, CH_{eq}	1'',2'',**4''**,**10''**,2''-	3''$_{ax}$	2''-Me
	3.03, d (14.5)	52.2, CH_{ax}	1'', 2'', 4'', 2''-	3''$_{eq}$	2''-Me
4''		196.3, C=O			
5''		160.7, C			
6''	7.35, d (9.0)	113.9, CH	4'', 5'', 8'', 10''	7''	5''-OMe
7''	7.96, d (8.9)	135.6, CH	1'', 5'', 9'', 5'''	6''	**4'''$_{eq}$**, 4'''$_{ax}$
8''		124.5, C			
9''		138.5, C			
10''		119.5, C			

Continuation of **Table S1**, see the next page.

Continuation of **Table S1**.

		Cyclombandakamine A (**1**)			
Position	δ_H (*J* in Hz)	HSQC	HMBC	COSY	ROESY
1'''	4.56, q (6.9)	46.6, CH	8''',9''',10''',1'''-	1'''-Me	7, 8'''-OMe
3'''	3.91, m	48.1, CH	4''', 3'''-Me	4'''$_{eq}$,3'''-Me	1'''-Me
4'''	3.68, dd (16.5,	33.5, CH$_{eq}$	5''', 9''', 10'''	3''', 4'''$_{ax}$	7'', 3'''-Me
	3.05, *dd* (16.7, 8.1)	33.5, CH$_{ax}$	3''',9''',10''',3'''-Me	3''', 4'''$_{eq}$	3,7,7'',3'''-Me
5'''		119.2, C			
6'''		154.7, C			
7'''	6.45, *s*	102.7, CH	1''', 5''', 6''', 8''',		**8-OMe**,**2''-Me**,8'''-OMe
8'''		158.6, C			
9'''		118.2, C			
10'''		128.7, C			
1'''-Me	1.67, d (6.6)	20.2, Me	1''', 9'''	1'''	3''', 8'''-OMe
3'''-Me	0.72, d (6.9)	14.9, Me	3''', 4'''	3'''	**7**,**3**,7',4'''$_{eq}$,4'''$_{ax}$,**1**,**8-OMe**
2''-Me	1.85, s	18.4, Me	1'', 2'', 3''		**3''$_{eq}$**, 3''$_{ax}$, 4'-OMe, **7'''**
8'''-OMe	3.72, s	56.5, Me	8'''		7''', 1''', 1'''-Me
5''-OMe	3.96, s	56.8, Me	5''		5''

Table S2. Detailed NMR data of 1-*epi*-cyclombandakamine A (**2**) in methanol-d_4 (δ in ppm, *J* in Hz).

		1-*epi*-Cyclombandakamine A (**2**)			
Position	δ_H (*J* in Hz)	HSQC	HMBC	COSY	ROESY
1	4.52, q (6.5)	**50.0**, CH	8, 9, 10, 1-Me	1-Me	**3**, 8-OMe
3	3.09, m	**50.6**, CH	4, 3-Me	4_{eq},3-Me	**1**
4	2.17, dd(17.4,3.4)	32.9, CH_{eq}	5, 9, 10	3, 4_{ax}	**7'**, 3-Me
	1.94, dd(18.4,11.8)	32.9, CH_{ax}	3, 9, 10, 3-Me	3, 4eq	1', 3-Me
5		119.9, C			
6		156.5, C			
7	6.40, s	98.6, CH	1, 5, 6, 8, 9		8-OMe,**1'''**,**3'''-Me**
8		158.5, C			
9		113.9, C			
10		135.1, C			
1'	6.60, s	118.1, CH	3', 10', 8', 9'		4_{ax}, 2'-Me
2'		139.6, C			
3'	6.80, s	108.7, CH	1', 2', 4', 10'		2'-Me, 4'-OMe
4'		158.5, C			
5'		156.4, C			
6'		121.2, C			
7'	6.38, s	126.3, CH	5, 5', 9', 10', 1''		4_{eq}, 3'''-Me,**4'''$_{ax}$**, **7'''**
8'		126.3, C			
9'		139.0, C			
10'		114.4, C			
1-Me	1.50, d (6.7)	20.2, Me	1, 9	1	8-OMe
3-Me	1.09, d (6.2)	18.6 Me	3, 4	3	4_{eq}, 4_{ax}
2'-Me	2.30, s	22.6, Me	1', 2', 3'		1', 3'
8-OMe	3.84, s	56.0, Me	8		1, 7, 1-Me
4'-OMe	4.00, s	56.6, Me	4'		3', 2''-Me
1''		**84.4**, C			
2''		**92.4**, C			
3''	3.12, d (14.8)	**52.2**, CH_{eq}	1'',2'',**4''**,**10''**,2''-Me	3''$_{ax}$	2''-Me
	3.02, d (14.8)	**52.2**, CH_{ax}	1'', 2'', 4'', 2''-Me	3''$_{eq}$	2''-Me
4''		**196.3**, C=O			
5''		160.7, C			
6''	7.35, d (9.1)	113.9, CH	4'', 5'', 8'', 10''	7''	5''-OMe
7''	7.97, d (8.9)	135.5, CH	1'', 5'', 9'', 5'''	6''	**4'''$_{eq}$**, 4'''$_{ax}$
8''		124.5, C			
9''		138.3, C			
10''		119.6, C			

Continuation of **Table S2**, see the next page.

Continuation of **Table S2**.

	Cyclombandakamine A (**1**)				
Position	δ_H (*J* in Hz)	HSQC	HMBC	COSY	ROESY
1'''	4.62 q (6.6)	46.6, CH	8''',9''',10''',1'''-	1'''-Me	7, 8'''-OMe
3'''	3.94, m	48.1, CH	4''', 3'''-Me	4'''$_{eq}$,3'''-Me	1'''-Me
4'''	3.06, dd (16.5,	33.6, CH_{eq}	5''', 9''', 10'''	3''', 4'''$_{ax}$	7'', 3'''-Me
	3.71, *dd* (16.1, 5.0)	33.6, CH_{ax}	3''',9''',10''',3'''-Me	3''', 4'''eq	3,7,7'',3'''-Me
5'''		118.9, C			
6'''		154.7, C			
7'''	6.47, *s*	102.6, CH	1''', 5''', 6''', 8''',		**8-OMe**,**2''-Me**,8'''-OMe
8'''		158.6, C			
9'''		118.3, C			
10'''		128.7, C			
1'''-Me	1.69, d (6.9)	20.2, Me	1''', 9'''	1'''	3''', 8'''-OMe
3'''-Me	0.89, d (6.5)	14.9, Me	3''', 4'''	3'''	7,7',4'''$_{eq}$,4'''$_{ax}$, 8-OMe
2''-Me	1.83, s	18.4, Me	1'', 2'', 3''		**3''$_{eq}$**, 3''ax, 4'-OMe, 7'''
8'''-OMe	3.73, s	56.5, Me	8'''		7''', 1''', 1'''-Me
5''-OMe	3.96, s	56.8, Me	5''		5''

Table S3. Detailed NMR data of cyclombandakamine A_3 (**3**) in methanol-d_4 (δ in ppm, J in Hz).

		Cyclombandakamine A_3 (**3**)			
Position	δ_H (J in Hz)	HSQC	HMBC	COSY	ROESY
1	4.67, q (7.3)	49.0, CH	8, 9, 10, 1-Me	1-Me	8-OMe
3	3.50, m	44.8, CH	4, 3-Me	4_{eq},3-Me	1-Me,4'''$_{ax}$,7''',8'''-OMe
4	2.33, br dd	32.8, CH_{eq}	5, 9, 10	3, 4_{ax}	7', 3-Me
	1.94, br dd	32.8, CH_{ax}	3, 9, 10, 3-Me	3, 4eq	3-Me
5		119.8, C			
6		157.6, C			
7	6.39, s	98.6, CH	1, 5, 6, 8, 9		8-OMe,**1'''-Me**,3'''-Me
8		157.5, C			
9		114.2, C			
10		133.1, C			
1'	6.53, s	118.0, CH	3', 10', 8', 9'		4_{ax}, 2'-Me
2'		139.6, C			
3'	6.79, s	108.7, CH	1', 2', 4', 10'		2'-Me, 4'-OMe
4'		158.5, C			
5'		156.4, C			
6'		121.2, C			
7'	6.36, s	126.1, CH	5, 5', 9', 10', 1"		4_{eq}, 3'''-Me, **7'''**
8'		126.5, C			
9'		138.4, C			
10'		114.3, C			
1-Me	1.50, d (6.6)	18.8, Me	1, 9	1	8-OMe,**1'''**, 8'''-OMe
3-Me	1.07, d (6.3)	19.3, Me	3, 4	3	4_{eq}, 4_{ax}
2'-Me	2.28, s	22.5, Me	1', 2', 3'		1', 3'
8-OMe	3.86, s	56.2, Me	8		1, 7, 1-Me
4'-OMe	4.00, s	56.6, Me	4'		3', 2"-Me
1"		85.3, C			
2"		92.4, C			
3"	3.12, d (14.6)	52.3, CH_{eq}	1",2",**4''**,**10''**,2"-Me	3"$_{ax}$	2"-Me
	3.03, d (14.6)	52.3, CH_{ax}	1", 2", 4", 2"-Me	3"$_{eq}$	2"-Me
4"		196.3, C=O			
5"		160.6, C			
6"	7.35, d (9.0)	113.8, CH	4", 5", 8", 10"	7"	5"-OMe
7"	7.97, d (9.2)	135.6, CH	1", 5", 9", 5'''	6"	**4'''$_{eq}$**, 4'''$_{ax}$
8"		125.2, C			
9"		138.7, C			
10"		119.5, C			

Continuation of **Table S3**, see the next page.

Continuation of **Table S3**.

		Cyclombandakamine A_3 (**3**)			
Position	δ_H (*J* in Hz)	HSQC	HMBC	COSY	ROESY
1'''	4.49, br q	52.9, CH	8''',9''',10''',1'''-Me	1'''-Me	7, **3'''**,1-Me,8'''-OMe
3'''	3.81, m	50.2, CH	4''', 3'''-Me	4'''$_{eq}$,3'''-Me	**1'''**
4'''	3.64, br dd	33.2, CH_{eq}	5''', 9''', 10'''	3''', 4'''$_{ax}$	7'', 3'''-Me
	2.99, br dd	33.2, CH_{ax}	3''',9''',10''',3'''-Me	3''', 4'''$_{eq}$	3,7,7'',3'''-Me
5'''		119.3, C			
6'''		154.7, C			
7'''	6.43, *s*	102.4, CH	1''', 5''', 6''', 8''', 9'''		**3,8-OMe**,**2''-Me**,8'''-OMe
8'''		158.6, C			
9'''		118.9, C			
10'''		131.0, C			
1'''-Me	1.63, d (6.4)	20.4, Me	1''', 9'''	1'''	8'''-OMe
3'''-Me	0.71, d (6.9)	14.6, Me	3''', 4'''	3'''	**1-Me**,**8-OMe**,7,7',4'''$_{eq}$,4'''$_{ax}$
2''-Me	1.85, s	18.4, Me	1'', 2'', 3''		**3''$_{eq}$**, 3''$_{ax}$, 4'-OMe, **7'''**
8'''-OMe	3.72, s	56.4, Me	8'''		3,7''', 1''', 1'''-Me
5''-OMe	3.96, s	56.8, Me	5''		5''

Table S4. Detailed NMR data of cyclombandakamine A$_4$ (**4**) in methanol-d_4 (δ in ppm, J in Hz).

		Cyclombandakamine A$_4$ (**4**)			
Position	δ_H (J in Hz)	HSQC	HMBC	COSY	ROESY
1	4.70, q (6.8)	49.1, CH	8, 9, 10, 1-Me	1-Me	8-OMe
3	3.50, m	44.9, CH	4, 3-Me	4_{eq}, 3-Me	**1-Me**, $4'''_{ax}$
4	2.34, dd (18.2, 4.9)	32.8, CH_{eq}	5, 9, 10	3, 4_{ax}	7', 3-Me
	1.95, dd (18.2, 11.6)	32.8, CH_{ax}	3, 9, 10, 3-Me	3, 4eq	3-Me
5		120.0, C			
6		157.1, C			
7	6.39, s	98.4, CH	1, 5, 6, 8, 9		8-OMe,$4'''_{ax}$,3'''-Me
8		157.5, C			
9		114.3, C			
10		133.6, C			
1'	6.48, s	117.9, CH	3', 10', 8', 9'		4_{ax}, 2'-Me
2'		139.8, C			
3'	6.80, s	108.8, CH	1', 2', 4', 10'		2'-Me, 4'-OMe
4'		158.5, C			
5'		156.6, C			
6'		121.0, C			
7'	6.34, s	126.0, CH	5, 5', 9', 10', 1"		4_{eq}, 3'''-Me, **7'''**
8'		126.6, C			
9'		138.7, C			
10'		114.3, C			
1-Me	1.53, d (6.8)	18.8, Me	1, 9	1	3, 8-OMe
3-Me	1.09, d (6.4)	19.1, Me	3, 4	3	4_{eq}, 4_{ax}
2'-Me	2.28, s	22.5, Me	1', 2', 3'		1', 3'
8-OMe	3.88, s	56.3, Me	8		1, 7, 1-Me
4'-OMe	4.00, s	56.6, Me	4'		3', 2"-Me
1"		85.4, C			
2"		92.5, C			
3"	3.11, d (14.9)	52.0, CH_{eq}	1",2",**4''**,**10''**,2"-Me	$3''_{ax}$	2"-Me
	3.04, d (14.9)	52.0, CH_{ax}	1", 2", 4", 2"-Me	$3''_{eq}$	2"-Me
4"		196.2, C=O			
5"		160.8, C			
6"	7.35, d (9.2)	114.0, CH	4", 5", 8", 10"	7"	5"-OMe
7"	8.29, d (9.1)	135.1, CH	1", 5", 9", 5'''	6"	**$4'''_{eq}$**, $4'''_{ax}$
8"		124.6, C			
9"		138.8, C			
10"		119.4, C			

Continuation of **Table S4**, see the next page.

Continuation of **Table S4**.

		Cyclombandakamine A_4 (**4**)			
Position	δ_H (*J* in Hz)	HSQC	HMBC	COSY	ROESY
1'''	5.04, q (6.9)	71.3, CH	8''',9''',10''',1'''-Me	1'''-Me	7, **3'''**,8'''-OMe
3'''	4.46, m	63.3, CH	4''', 3'''-Me	$4'''_{eq}$,3'''-Me	1''',2'''-NMe
4'''	4.43, dd (18.1, 6.5)	33.5, CH_{eq}	5''', 9''', 10'''	3''', $4'''_{ax}$	7'', 3'''-Me
	2.91, dd (18.1, 11.2)	33.5, CH_{ax}	3''',9''',10''',3'''-Me	3''', $4'''_{eq}$	3,7,7'',3'''-Me
5'''		117.7, C			
6'''		155.7, C			
7'''	6.47, s	102.7, CH	1''', 5''', 6''', 8''', 9'''		**8-OMe**,**2''-Me**,8'''-OMe
8'''		158.0, C			
9'''		119.2, C			
10'''		127.9, C			
1'''-Me	1.78, d (6.6)	16.5, Me	1''', 9'''	1'''	8'''-OMe
3'''-Me	1.46, d (6.3)	14.8, Me	3''', 4'''	3'''	7,7',$4'''_{eq}$,$4'''_{ax}$,**8-OMe**
2''-Me	1.84, s	18.3, Me	1'', 2'', 3''		**$3''_{eq}$**, $3''_{ax}$, 4'-OMe, 7'''
8'''-OMe	3.73, s	56.7, Me	8'''		7''', 1''', 1'''-Me
5''-OMe	3.97, s	56.8, Me	5''		5''
2'''-NMe	3.03, s	49.3, Me			**7**,3''',8'''-OMe

Table S5. Detailed NMR data of cyclombandakamine A_5 (**5**) in methanol-d_4 (δ in ppm, J in Hz).

		Cyclombandakamine A_5 (**5**)			
Position	δ_H (J in Hz)	HSQC	HMBC	COSY	ROESY
1	4.53, q (6.5)	52.0, CH	8, 9, 10, 1-Me	1-Me	**3**,8-OMe,**2'''-NMe**
3	3.09, m	50.6, CH	4, 3-Me	4_{eq}, 3-Me	**1**, 4'''$_{ax}$,**3'''-Me**
4	2.17, dd (17.4, 3.4)	33.0, CH_{eq}	5, 9, 10	3, 4_{ax}	7', 3-Me
	2.05, dd (17.4, 11.8)	33.0, CH_{ax}	3, 9, 10, 3-Me	3, 4eq	1', 3-Me
5		120.0, C			
6		157.0, C			
7	6.39, s	99.1, CH	1, 5, 6, 8, 9		8-OMe,4'''$_{ax}$,1''',3'''-Me
8		158.6, C			
9		114.2, C			
10		133.6, C			
1'	6.55, s	118.1, CH	3', 10', 8', 9'		4_{ax}, 2'-Me
2'		139.8, C			
3'	6.81, s	108.7, CH	1', 2', 4', 10'		2'-Me, 4'-OMe
4'		158.5, C			
5'		156.6, C			
6'		121.0, C			
7'	6.37, s	126.2, CH	5, 5', 9', 10', 1''		4_{eq}, 3'''-Me, **7'''**
8'		126.1, C			
9'		139.0, C			
10'		114.5, C			
1-Me	1.69, d (6.5)	20.2, Me	1, 9	1	8-OMe,**2'''-NMe**
3-Me	1.09, d (6.5)	14.8, Me	3, 4	3	4_{eq}, 4_{ax}
2'-Me	2.30, s	22.6, Me	1', 2', 3'		1', 3'
8-OMe	3.86, s	56.2, Me	8		1, 7, 1-Me,**2'''-NMe**
4'-OMe	4.00, s	56.6, Me	4'		3', 2''-Me
1''		85.4, C			
2''		92.4, C			
3''	3.11, d (14.8)	52.0, CH_{eq}	1'',2'',**4''**,**10''**,2''-Me	3''$_{ax}$	2''-Me
	3.02, d (14.8)	52.0, CH_{ax}	1'', 2'', 4'', 2''-Me	3''$_{eq}$	2''-Me
4''		196.2, C=O			
5''		160.8, C			
6''	7.34, d (9.2)	114.0, CH	4'', 5'', 8'', 10''	7''	5''-OMe
7''	8.32, d (9.2)	135.1, CH	1'', 5'', 9'', 5'''	6''	**4'''$_{eq}$**, 4'''$_{ax}$,**7'**
8''		124.6, C			
9''		138.8, C			
10''		119.5, C			

Continuation of **Table S5**, see the next page.

Continuation of **Table S5**.

Cyclombandakamine A_5 (**5**)					
Position	δ_H (*J* in Hz)	HSQC	HMBC	COSY	ROESY
1'''	5.03, q (6.5)	71.4, CH	8''',9''',10''',1'''-Me	1'''-Me	7, **3'''**,8'''-OMe
3'''	4.40, m	63.0, CH	4''', 3'''-Me	$4'''_{eq}$,3'''-Me	1''',2'''-NMe
4'''	4.42, dd (18.2, 6.2)	34.9, CH_{eq}	5''', 9''', 10'''	3''', $4'''_{ax}$	7'', 3'''-Me
	2.90, dd (18.2,	34.9, CH_{ax}	3''',9''',10''',3'''-Me	3''', $4'''_{eq}$	3,7,7'',3'''-Me
5'''		117.4, C			
6'''		155.5, C			
7'''	6.46, s	102.7, CH	1''', 5''', 6''', 8''', 9'''		**8-OMe**,**2''-Me**,8'''-OMe
8'''		158.0, C			
9'''		119.8, C			
10'''		128.1, C			
1'''-Me	1.78, d (6.9)	16.5, Me	1''', 9'''	1'''	8'''-OMe
3'''-Me	1.50, d (6.2)	18.6, Me	3''', 4'''	3'''	7,7',$4'''_{eq}$,$4'''_{ax}$,**8-OMe**
2''-Me	1.83, s	18.3, Me	1'', 2'', 3''		**$3''_{eq}$**, $3''_{ax}$, 4'-OMe, 7'''
8'''-OMe	3.73, s	56.7, Me	8'''		7''', 1''', 1'''-Me
5''-OMe	3.96, s	56.8, Me	5''		5''
2'''-NMe	3.11, s	49.3, Me			**1**,**3**,**7**,3''',8'''-OMe

Table S6. Detailed NMR data of cyclombandakamine A_6 (**6**) in methanol-d_4 (δ in ppm, J in Hz).

Cyclombandakamine A_6 (**6**)					
Position	δ_H (J in Hz)	HSQC	HMBC	COSY	ROESY
1		**175.2**, C	8, 9, 10, 1-Me		8-OMe
3	3.63, m	48.8, CH	4, 3-Me	4_{eq},3-Me	1-Me, **3'''-Me**, **4'''$_{ax}$**
4	2.43, dd (17.2, 5.8)	32.6, CH_{eq}	5, 9, 10	3, 4_{ax}	7', 3-Me
	2.07, dd (17.3, 9.9)	32.6, CH_{ax}	3, 9, 10, 3-Me	3, 4eq	1', 3-Me
5		122.2, C			
6		167.4, C			
7	6.50, s	99.3, CH	1, 5, 6, 8, 9		8-OMe,**4'''$_{ax}$,1'''**,**3'''-Me**
8		165.6, C			
9		108.2, C			
10		142.7, C			
1'	6.49, s	117.7, CH	3', 10', 8', 9'		4_{ax},3'-Me,2'-Me
2'		140.1, C			
3'	6.81, s	108.8, CH	1', 2', 4', 10'		1',2'-Me, 4'-OMe
4'		158.5, C			
5'		156.8, C			
6'		121.1, C			
7'	6.39, s	126.0, CH	5, 5', 9', 10', 1''		4_{eq}, **3'''-Me**,**4'''$_{ax}$**
8'		124.9, C			
9'		138.5, C			
10'		114.3, C			
1-Me	**2.71**, **s**	**24.9**, Me	1, 9	1	8-OMe
3-Me	1.08, d (6.7)	17.8, Me	3, 4	3	4_{eq}, 4_{ax}
2'-Me	2.30, s	22.5, Me	1', 2', 3'		1', 3'
8-OMe	3.97, s	56.9, Me	8		7,1-Me,**1'''**,**3'''-Me**,**8'''-OMe**
4'-OMe	4.00, s	56.6, Me	4'		3', **2''-Me**
1''		85.3, C			
2''		92.5, C			
3''	3.11, d (14.1)	52.2, CH_{eq}	1'',2'',**4''**,**10''**,2''-	3''$_{ax}$	2''-Me
	3.02, d (14.6)	52.2, CH_{ax}	1'', 2'', 4'', 2''-	3''$_{eq}$	2''-Me
4''		196.2, C=O			
5''		160.7, C			
6''	7.36, d (9.0)	114.0, CH	4'', 5'', 8'', 10''	7''	5''-OMe, 3'''-Me
7''	7.97, d (9.0)	135.6, CH	1'', 5'', 9'', 5'''	6''	**4'''$_{eq}$**, 4'''$_{ax}$, **7'**
8''		124.5, C			
9''		138.3, C			
10''		119.5, C			

Continuation of **Table S6**, see the next page.

Continuation of **Table S6**.

		Cyclombandakamine A_6 (**6**)			
Position	δ_H (J in Hz)	HSQC	HMBC	COSY	ROESY
1'''	4.60, q (6.7)	46.6, CH	8''',9''',10''',1'''-	1'''-Me	**7**, 8'''-OMe
3'''	3.93, m	48.1, CH	4''', 3'''-Me	4'''$_{eq}$,3'''-Me	**1'''-Me**
4'''	3.69, dd (18.1,	33.5, CH$_{eq}$	5''', 9''', 10'''	3''', 4'''$_{ax}$	7'', 3'''-Me
	3.11, *dd* (18.1, 9.0)	33.5, CH$_{ax}$	3''',9''',10''',3'''-Me	3''', 4'''$_{eq}$	3,7,7'',3'''-Me
5'''		119.1, C			
6'''		154.7, C			
7'''	6.47, *s*	102.6, CH	1''', 5''', 6''', 8''', 9'''		**8-OMe**,**2''-Me**,3'''-Me,8'''-OMe
8'''		158.5, C			
9'''		118.3, C			
10'''		128.8, C			
1'''-Me	1.68, d (6.6)	20.3, Me	1''', 9'''	1'''	**3'''**, 8'''-OMe
3'''-Me	0.80, d (6.8)	14.6, Me	3''', 4'''	3'''	**7**,**7'**,4'''$_{eq}$,4'''$_{ax}$,**8-OMe**
2''-Me	1.85, s	18.3, Me	1'', 2'', 3''		**3''$_{eq}$**, 3''$_{ax}$, 4'-OMe, 7'''
8'''-OMe	3.73, s	56.5, Me	8'''		7''', 1''', 1'''-Me
5''-OMe	3.96, s	56.8, Me	5''		5''

Table S7. Detailed NMR data of cyclombandakamine A_7 (**7**) in methanol-d_4 (δ in ppm, J in Hz).

Cyclombandakamine A_7 (**7**)					
Position	δ_H (J in Hz)	HSQC	HMBC	COSY	ROESY
1		**175.4**, C	8, 9, 10, 1-Me		8-OMe
3	3.64, m	48.8, CH	4, 3-Me	4_{eq},3-Me	1-Me, **3'''-Me**, **4'''**$_{ax}$
4	2.43, dd (16.9, 5.6)	32.6, CH_{eq}	5, 9, 10	3, 4_{ax}	7', 3-Me
	2.06, dd (16.9, 10.1)	32.6, CH_{ax}	3, 9, 10, 3-Me	3, 4eq	1', 3-Me
5		122.2, C			
6		167.2, C			
7	6.52, s	99.0, CH	1, 5, 6, 8, 9		8-OMe,**4'''**$_{ax}$,**1'''-Me**,**3'''-Me**
8		165.7, C			
9		108.5, C			
10		143.0, C			
1'	6.45, s	117.6, CH	3', 10', 8', 9'		4_{ax},3'-Me,2'-Me
2'		140.1, C			
3'	6.81, s	108.9, CH	1', 2', 4', 10'		1',2'-Me, 4'-OMe
4'		158.5, C			
5'		157.0, C			
6'		121.0, C			
7'	6.37, s	125.9, CH	5, 5', 9', 10', 1''		4_{eq}, 3'''-Me,**4'''**$_{ax}$
8'		124.7, C			
9'		138.5, C			
10'		114.3, C			
1-Me	**2.73**, **d** (**1.2**)	**25.0**, Me	1, 9	1	8-OMe
3-Me	1.09, d (6.7)	17.8, Me	3, 4	3	4_{eq}, 4_{ax}
2'-Me	2.30, s	22.5, Me	1', 2', 3'		1', 3'
8-OMe	4.01, s	57.0, Me	8		7,1-Me,**1'''**,**3'''-Me**,**8'''-OMe**
4'-OMe	4.00, s	56.6, Me	4'		3', **2''-Me**
1''		85.4, C			
2''		92.5, C			
3''	3.09, d (14.9)	51.9, CH_{eq}	1'',2'',**4''**,**10''**,2''-	3''$_{ax}$	2''-Me
	3.02, d (14.9)	51.9, CH_{ax}	1'', 2'', 4'', 2''-	3''$_{eq}$	2''-Me
4''		196.1, C=O			
5''		160.8 C			
6''	7.36, d (9.1)	114.0, CH	4'', 5'', 8'', 10''	7''	5''-OMe, 3'''-Me
7''	8.31, d (9.1)	135.2, CH	1'', 5'', 9'', 5'''	6''	**4'''**$_{eq}$, 4'''$_{ax}$, **7'**
8''		124.7, C			
9''		138.8, C			
10''		119.4, C			

Continuation of **Table S7**, see the next page.

Continuation of **Table S7**.

			Cyclombandakamine A_7 (**7**)		
Position	δ_H (*J* in Hz)	HSQC	HMBC	COSY	ROESY
1'''	5.01, q (6.9)	71.3, CH	8''',9''',10''',1'''-	1'''-Me	7, **3''**',8'''-OMe
3'''	4.39, m	63.3, CH	4''', 3'''-Me	4'''$_{eq}$,3'''-Me	1''',2'''-NMe
4'''	4.42, br dd	34.7, CH_{eq}	5''', 9''', 10'''	3''', 4'''$_{ax}$	7'', 3'''-Me
	2.96, br dd	34.7, CH_{ax}	3''',9''',10''',3'''-Me	3''', 4'''$_{eq}$	3,7,7'',3'''-Me
5'''		117.6, C			
6'''		155.5, C			
7'''	6.46, s	102.7, CH	1''', 5''', 6''', 8''',		**8-OMe**,**2''-Me**,8'''-OMe
8'''		158.0, C			
9'''		119.8, C			
10'''		128.2, C			
1'''-Me	1.78, d (6.7)	16.5, Me	1''', 9'''	1'''	8'''-OMe
3'''-Me	1.48, d (6.3)	14.8, Me	3''', 4'''	3'''	7,7',4'''$_{eq}$,4'''$_{ax}$,**8-OMe**
2''-Me	1.83, s	18.3, Me	1'', 2'', 3''		**3''$_{eq}$**, 3''$_{ax}$, 4'-OMe, 7'''
8'''-OMe	3.73, s	56.8, Me	8'''		7''', 1''', 1'''-Me
5''-OMe	3.97, s	56.8, Me	5''		5''
2'''-NMe	3.04, s	49.5, Me			**3,7**,3''',8'''-OMe

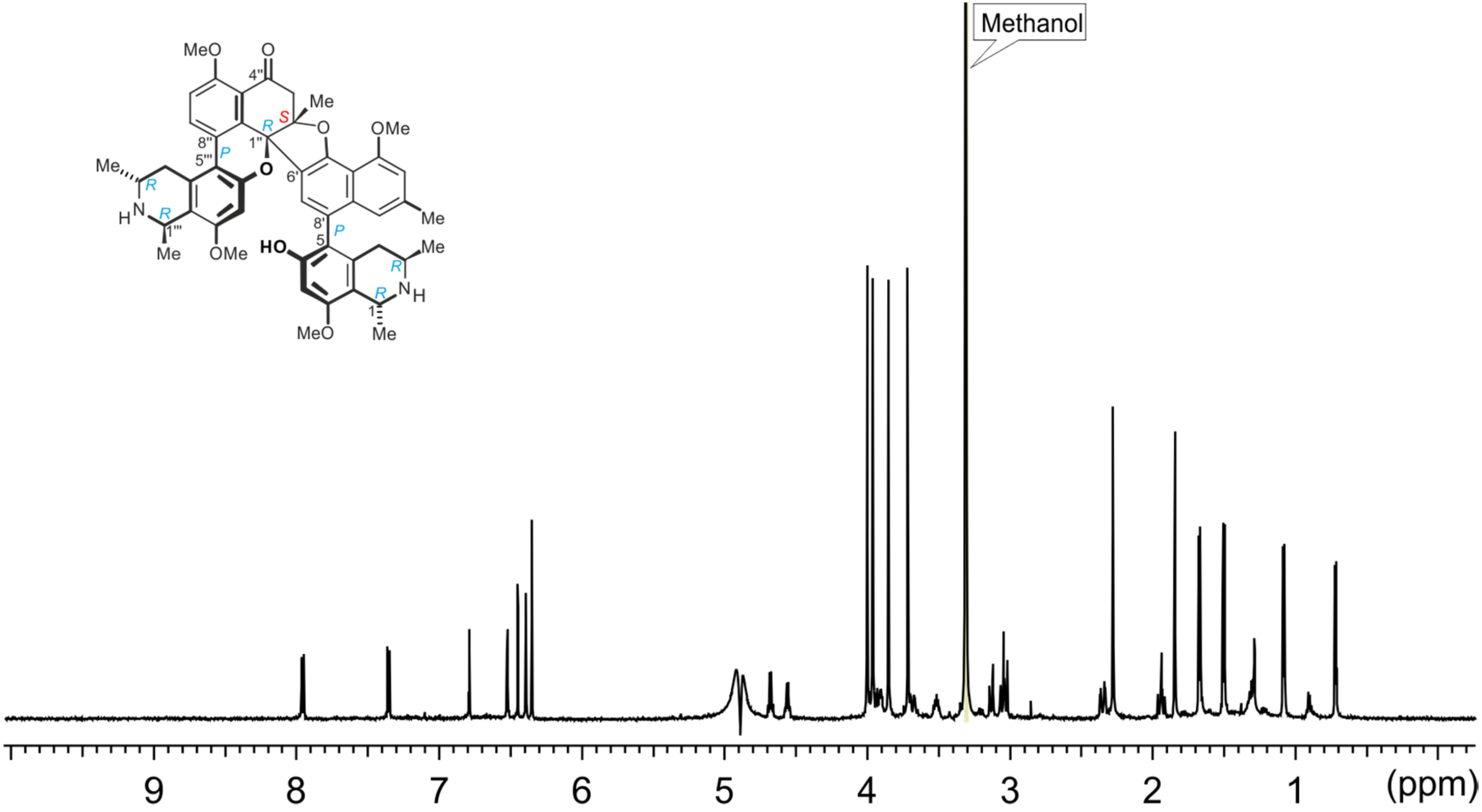

Figure S8a. Overall ^{1}H NMR spectrum of cyclombandakamine A (**1**) in methanol-d_4.

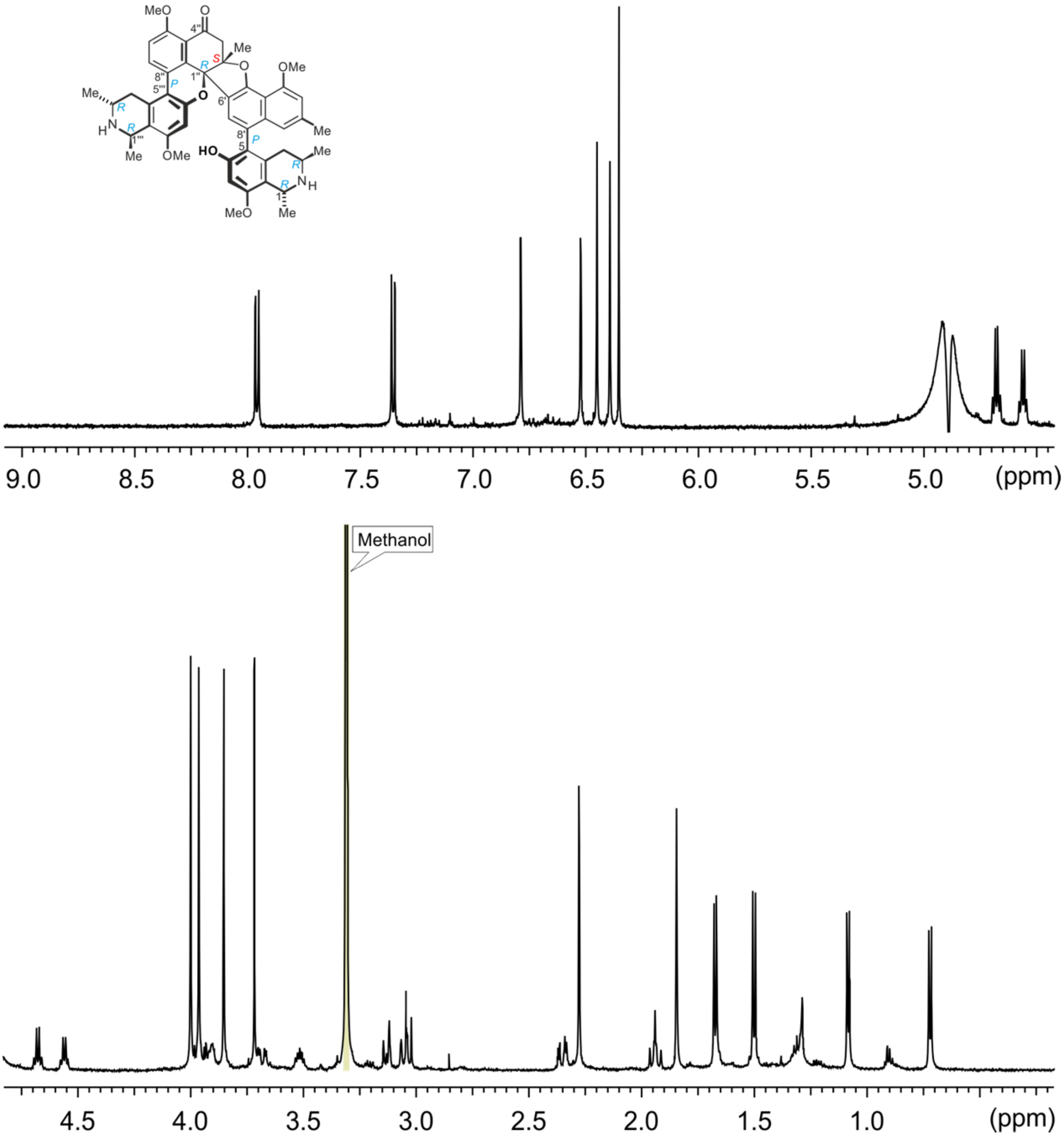

Figure S8b,c. Parts of the ^{1}H NMR spectrum of cyclombandakamine A (**1**) in methanol-d_4.

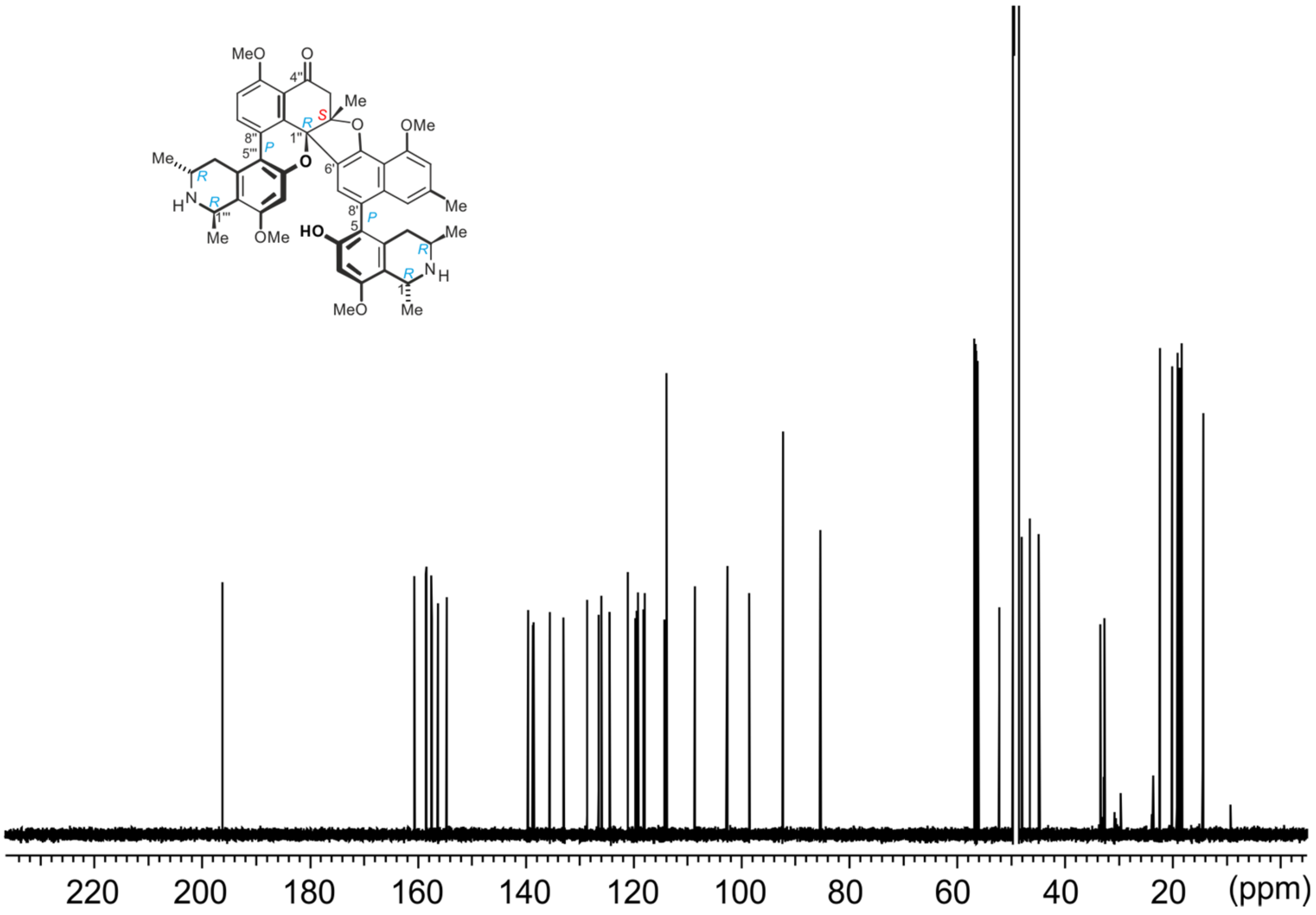

Figure S9a. Overall ^{13}C NMR spectrum of cyclombandakamine A (**1**) in methanol-d_4.

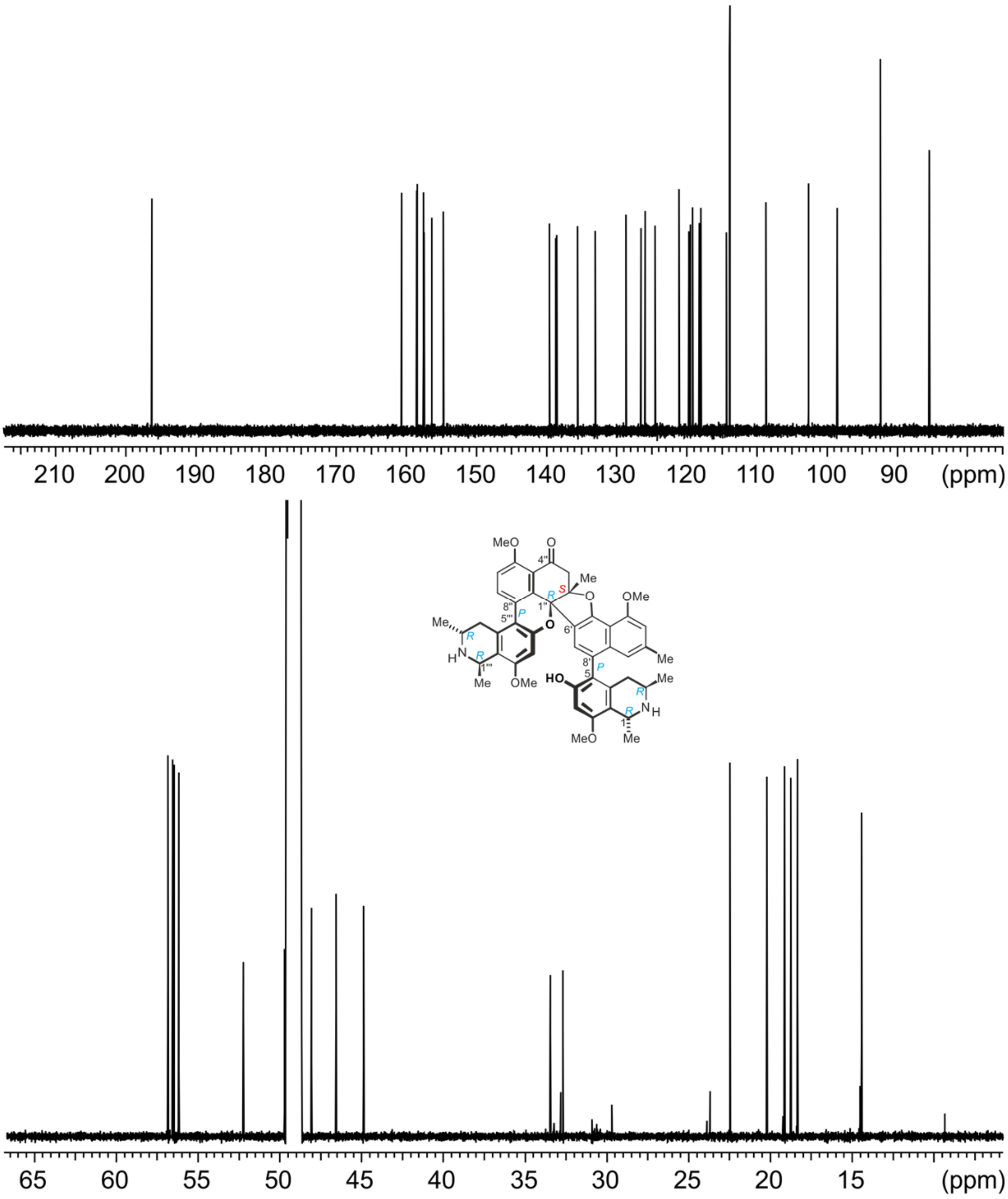

Figure S9b,c. Parts of the ^{13}C NMR spectrum of cyclombandakamine A (**1**) in methanol-d_4.

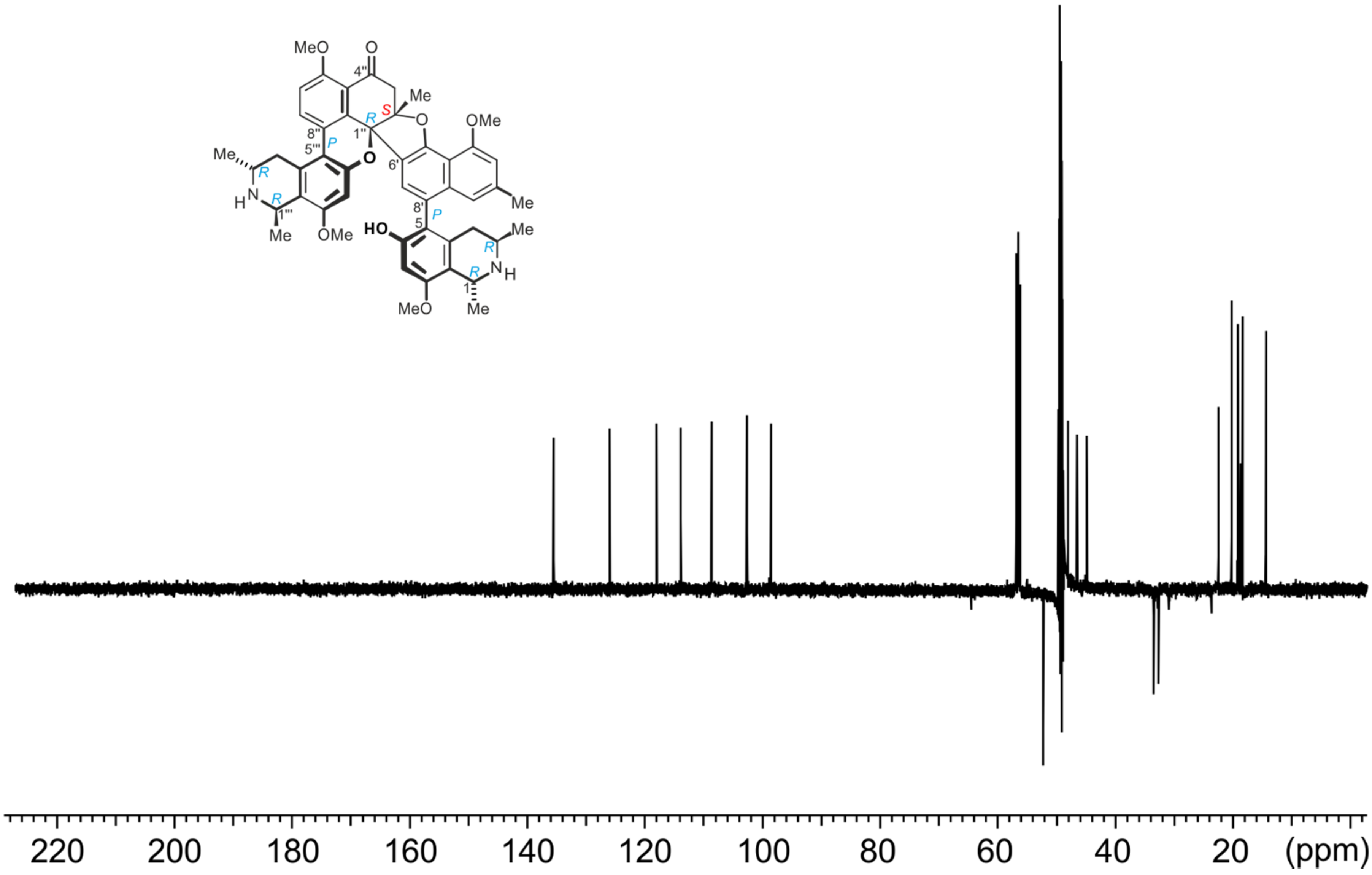

Figure S10. DEPT-135 NMR spectrum of cyclombandakamine A (**1**) in methanol-d_4.

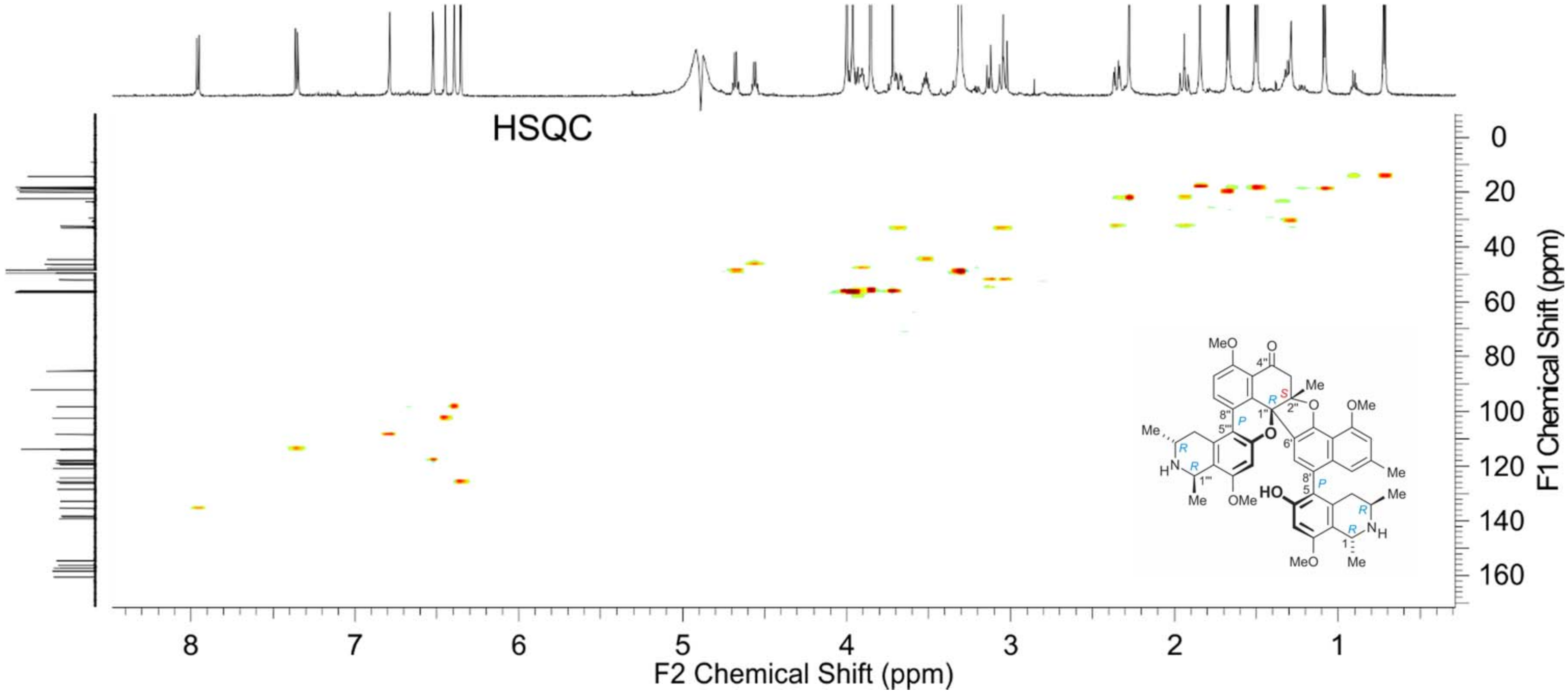

Figure S11. HSQC spectrum of cyclombandakamine A (**1**) in methanol-d_4.

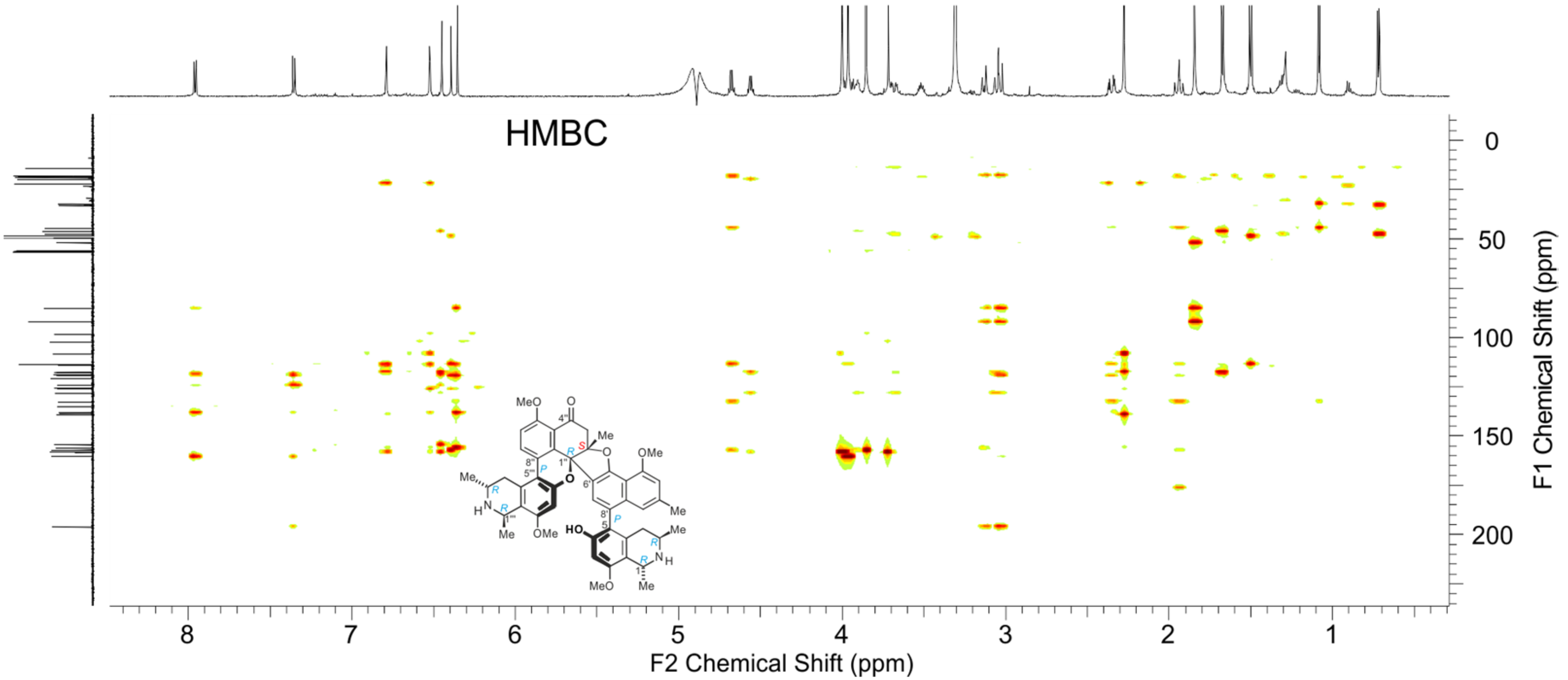

Figure S12. HMBC spectrum of cyclombandakamine A (**1**) in methanol-d_4.

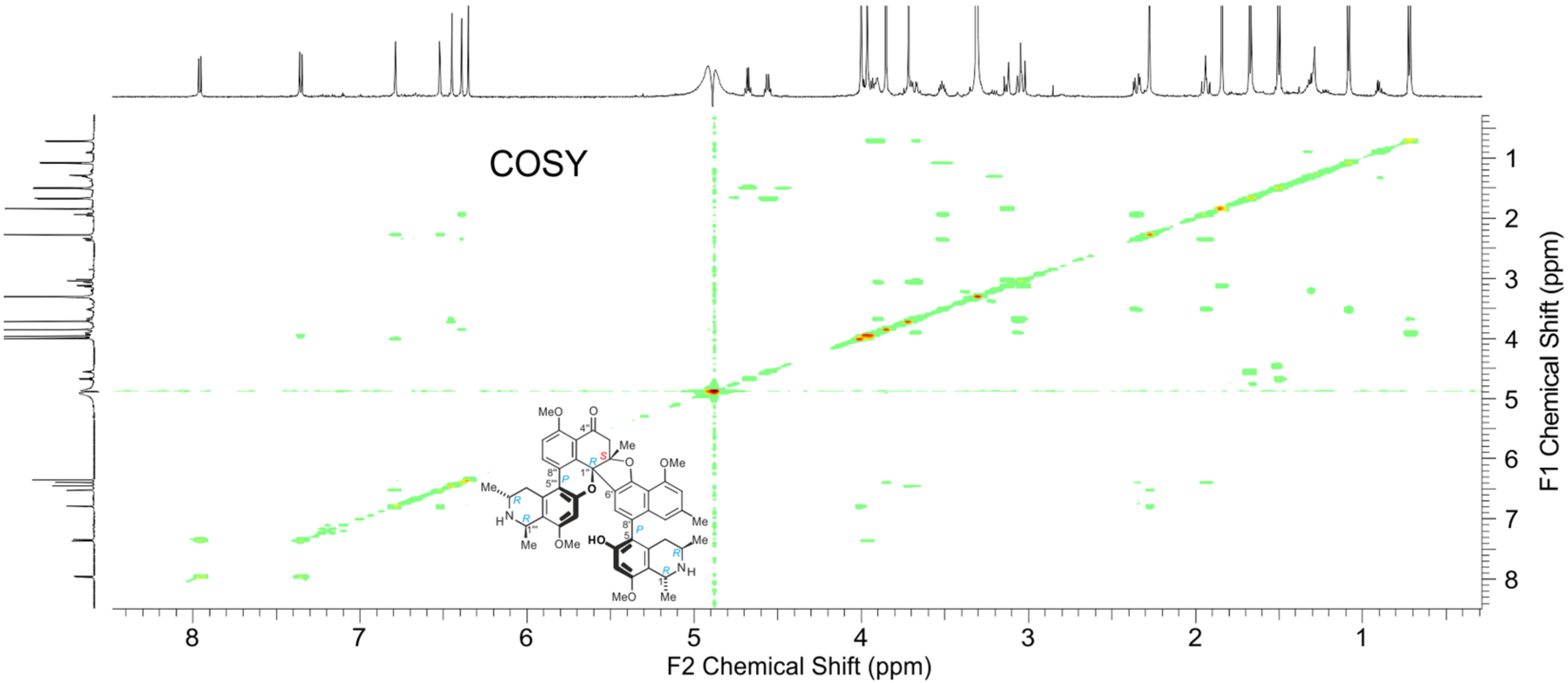

Figure S13. COSY spectrum of cyclombandakamine A (**1**) in methanol-d_4.

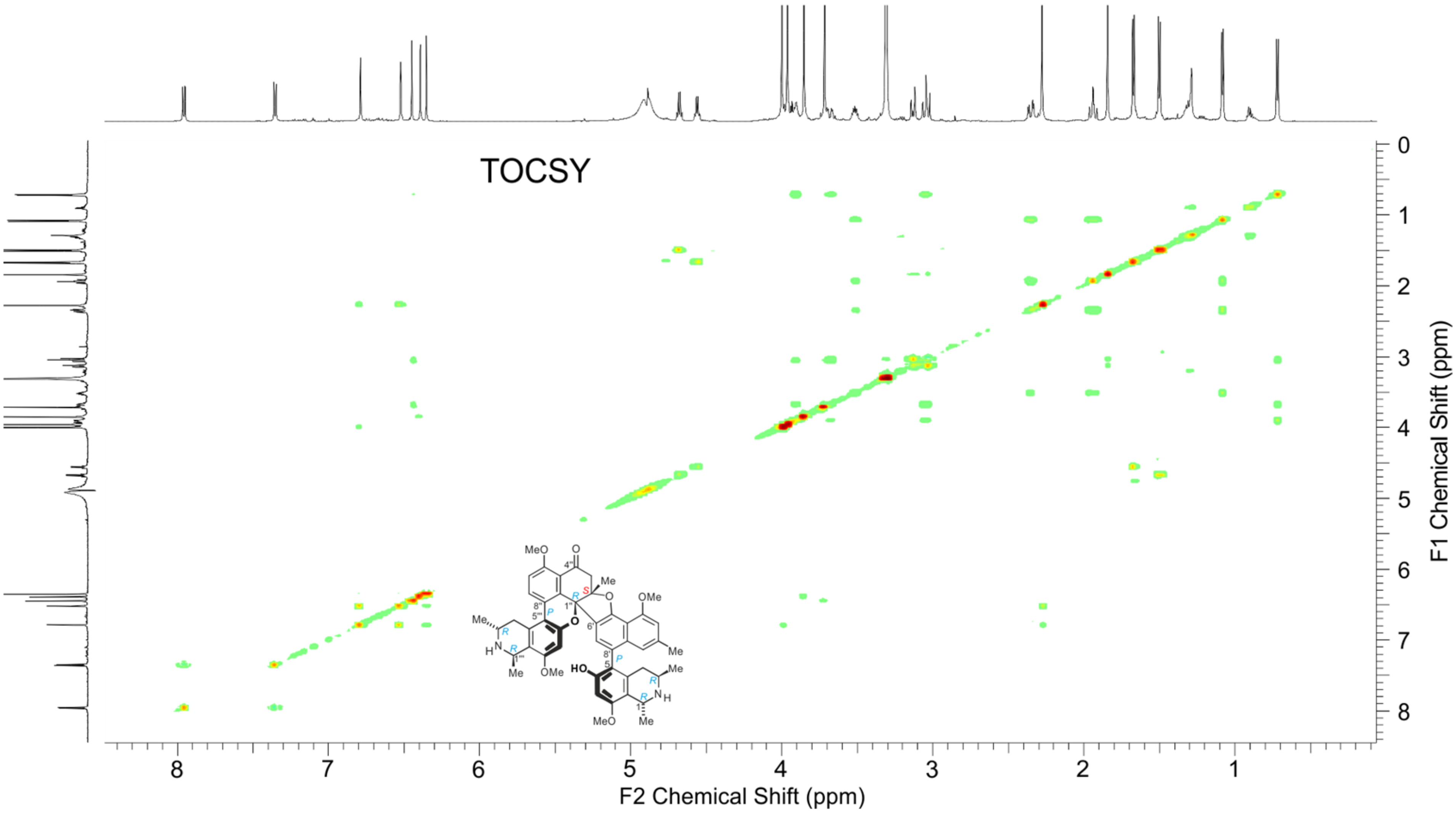

Figure S14. TOCSY spectrum of cyclombandakamine A (**1**) in methanol-d_4.

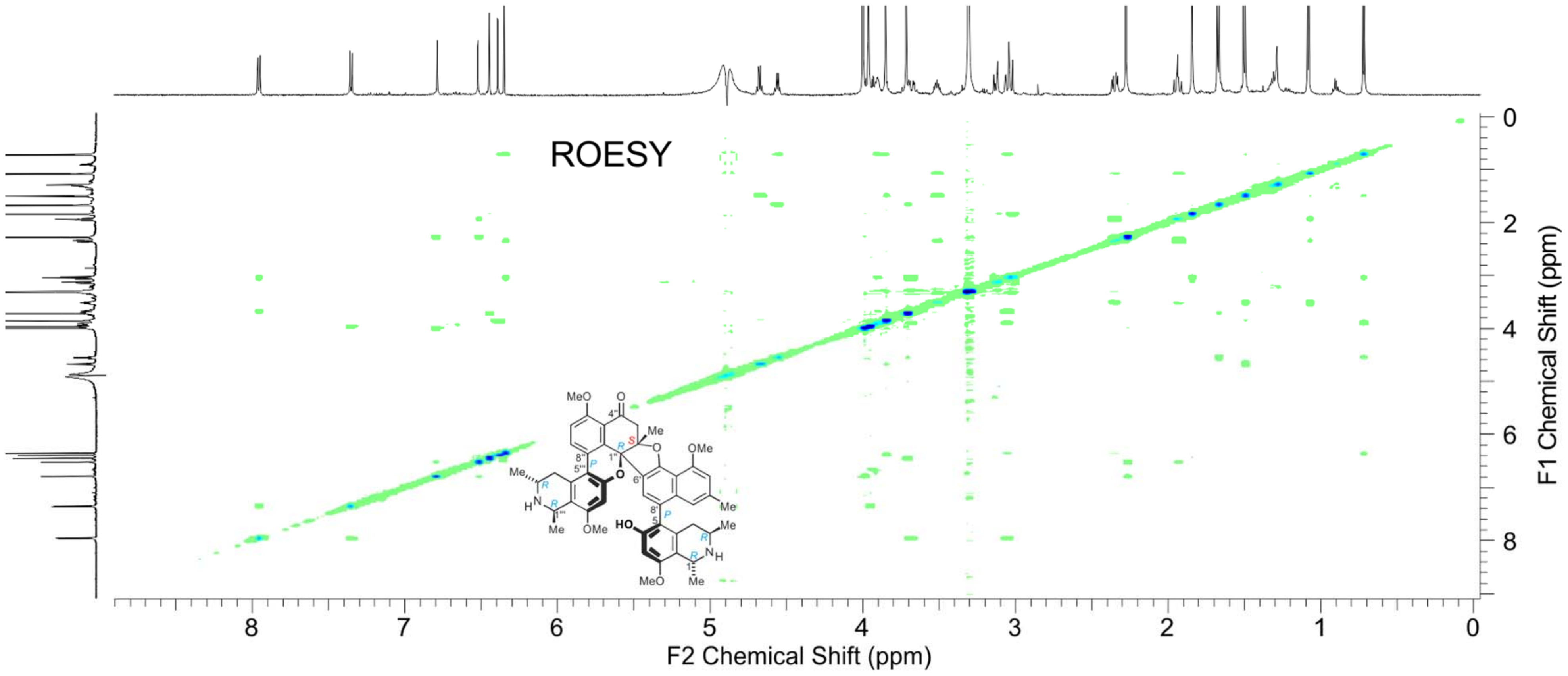

Figure S15a. ROESY spectrum of cyclombandakamine A (**1**) in methanol-d_4.

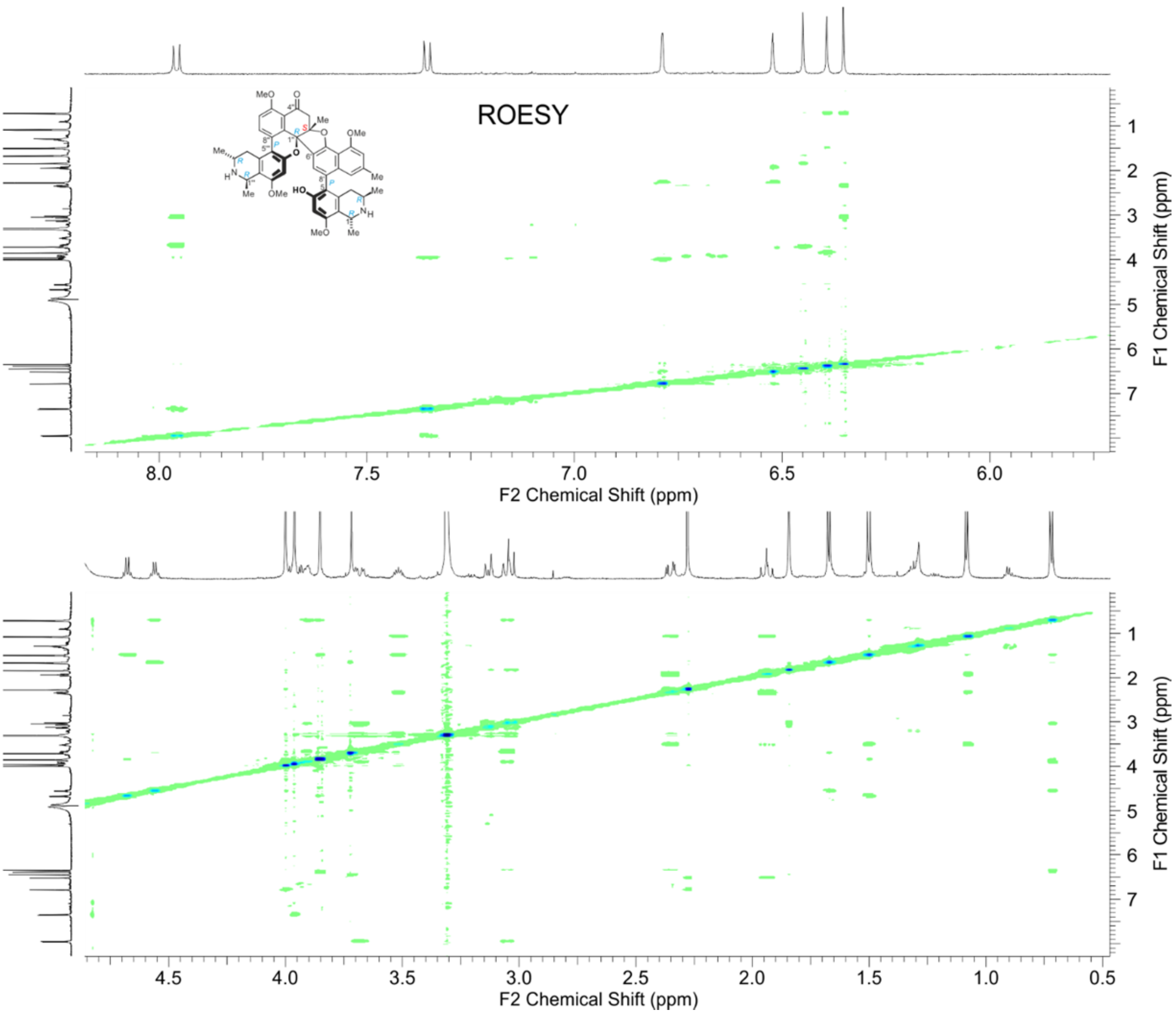

Figure S15b. Parts of the ROESY spectrum of cyclombandakamine A (**1**) in methanol-d_4.

Bruker Daltonics DataAnalysis 3.3

Mass Spectrum Molecular Formula Report

Analysis Info

Analysis Name	D:\Data\Spektren2016\2016_0359_BRI.d	Acquisition Date	15.02.2016 10:11:07
Method	esi_tune_pos_wide.m	Operator	Administrator
Comment	Tshitenge Dieudonne<br>AELV-B-59-T21-P7<br>2pmol/µl in Methanol	Instrument	micrOTOF 88

Acquisition Parameter

Source Type	ESI	Ion Polarity	Positive	Set Corrector Fill	48 V
Scan Range	n/a	Capillary Exit	180.0 V	Set Pulsar Pull	804 V
Scan Begin	50 m/z	Hexapole RF	280.0 V	Set Pulsar Push	807 V
Scan End	3500 m/z	Skimmer 1	50.0 V	Set Reflector	1700 V
		Hexapole 1	23.0 V	Set Flight Tube	8600 V
				Set Detector TOF	2240 V

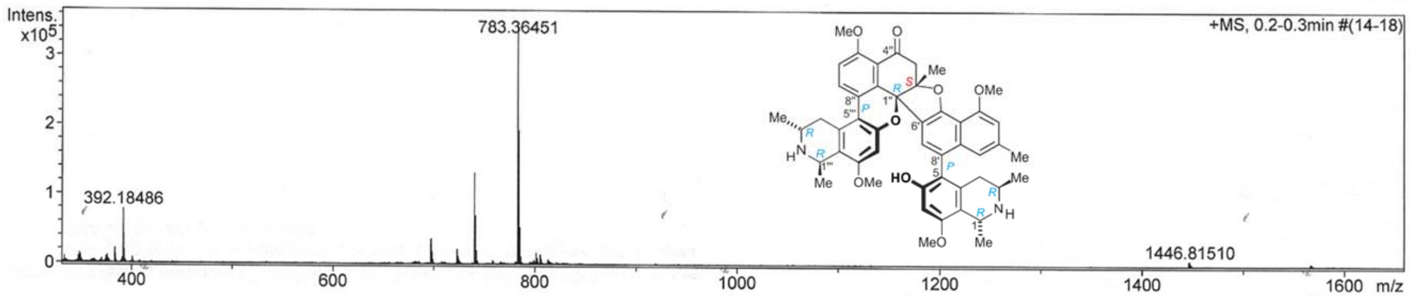

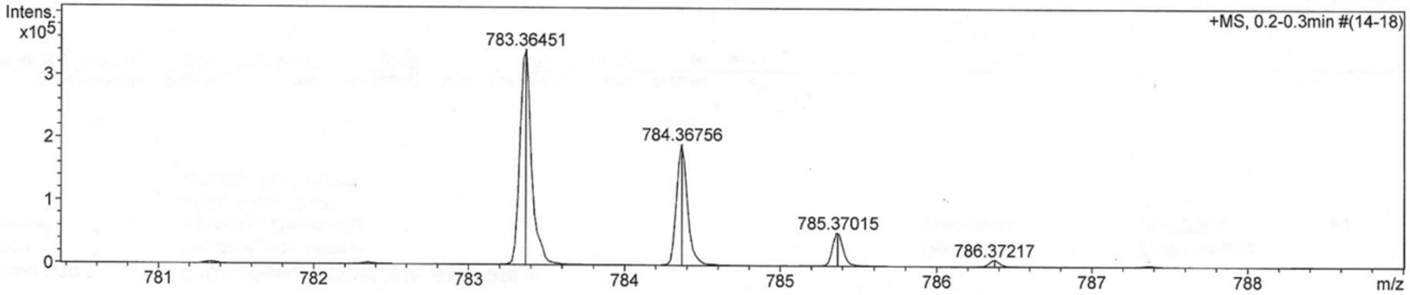

Sum Formula	Sigma	m/z	Err [ppm]	Mean Err [ppm]	rdb	N Rule	e⁻
C 48 H 51 N 2 O 8	0.01	783.36399	-0.66	-0.41	24.50	ok	even

Figure S16. HRESIMS of cyclombandakamine A (**1**) in acetonitrile.

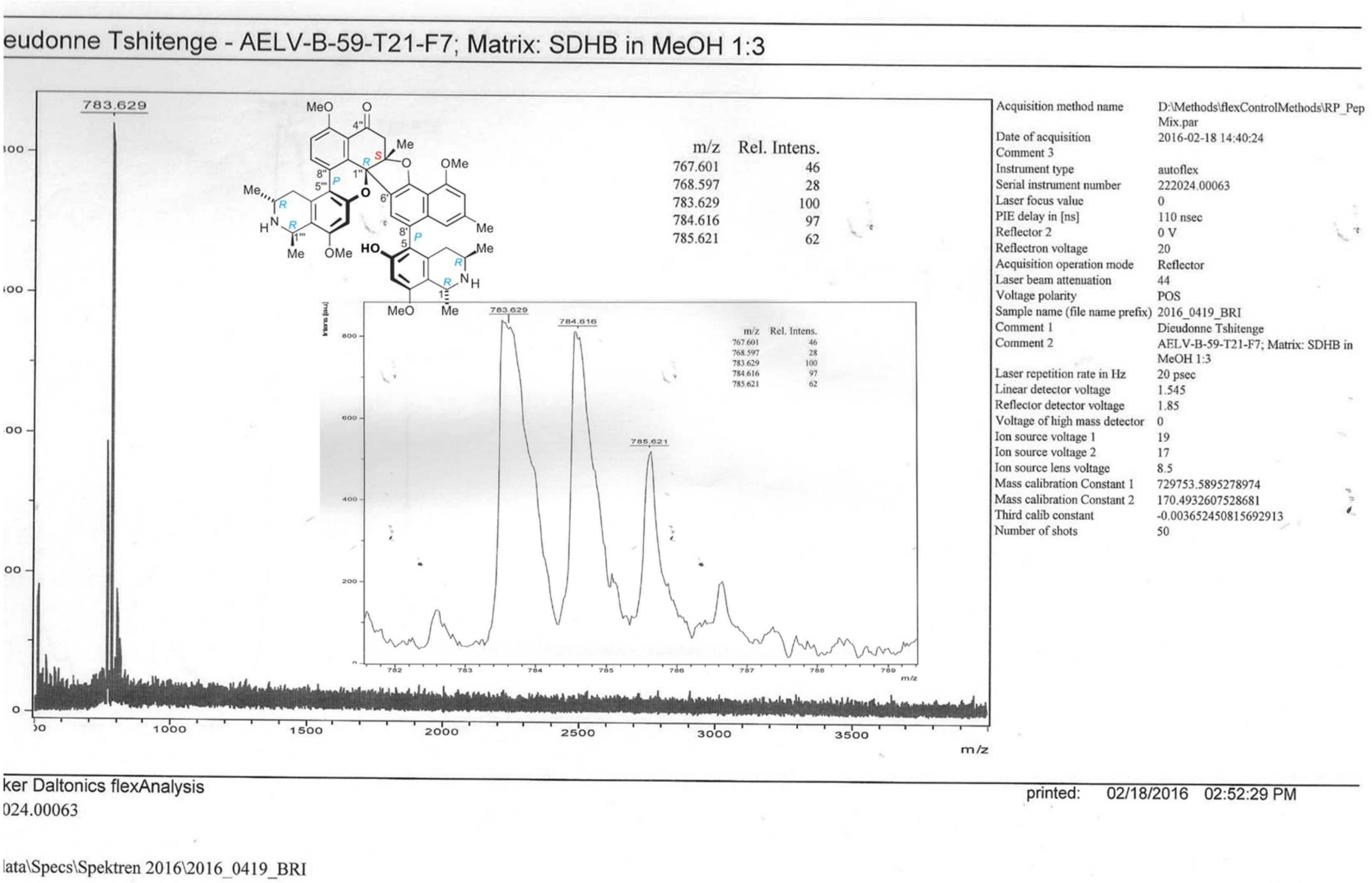

Figure S17. MALDI analysis: profile of cyclombandakamine A (**1**).

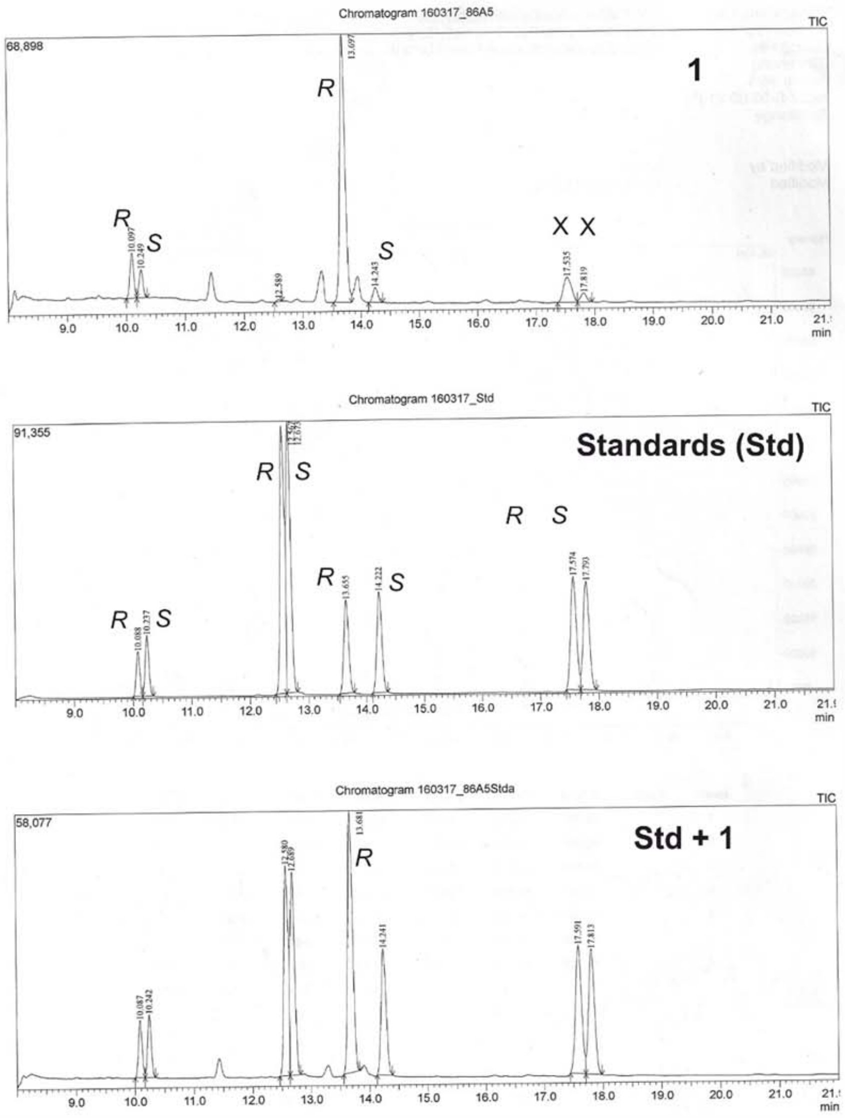

Modified by : Admin
Modified : 17.03.2016 11:14:25

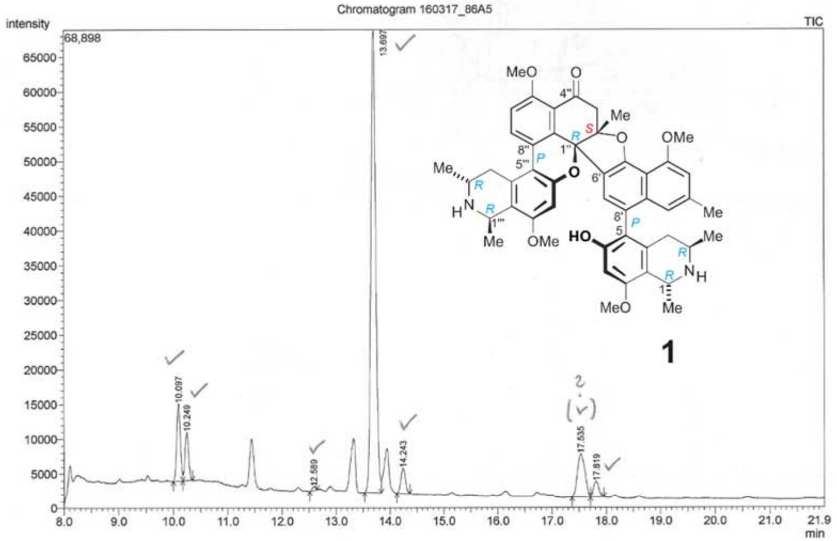

Peak#	Name	R.Time	I.Time	F.Time	Area	Area%	Height	Height%
1		10.097	10.000	10.175	50702	8.50	11192	11.49
2		10.249	10.175	10.350	32017	5.37	7023	7.21
3		12.589	12.515	12.645	2452	0.41	578	0.59
4		13.697	13.530	13.840	414227	69.45	66312	68.10
5		14.243	14.130	14.370	21762	3.65	3735	3.84
6		17.535	17.370	17.710	59631	10.00	6255	6.42
7		17.819	17.710	17.955	15686	2.63	2286	2.35
					596477	100.00	97381	100.00

R:S 19:1

Figure S18. Oxidative degradation results of cyclombandakamine A (**1**).

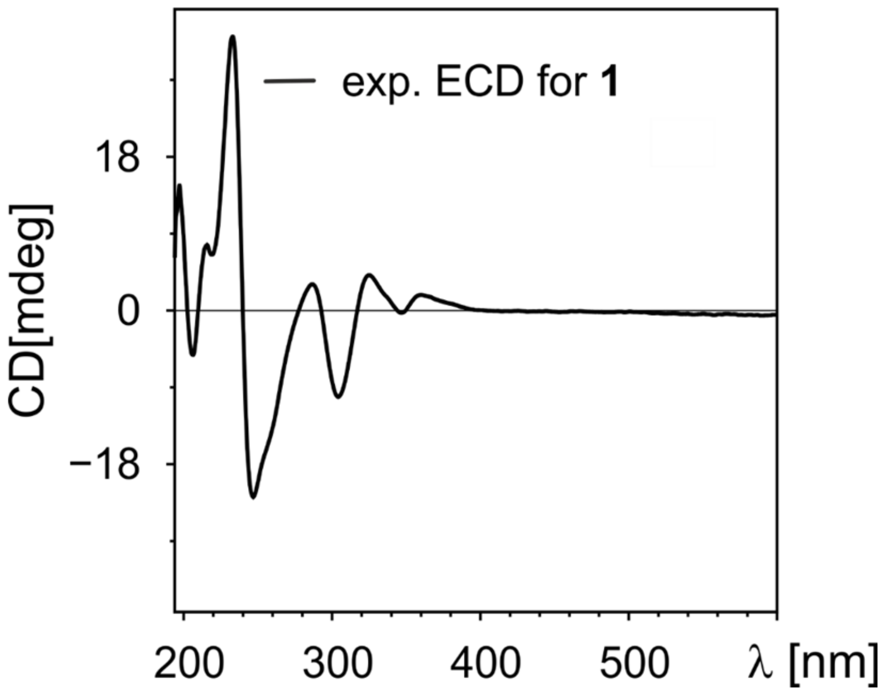

Figure S19. ECD spectrum of cyclombandakamine A (**1**) in methanol.

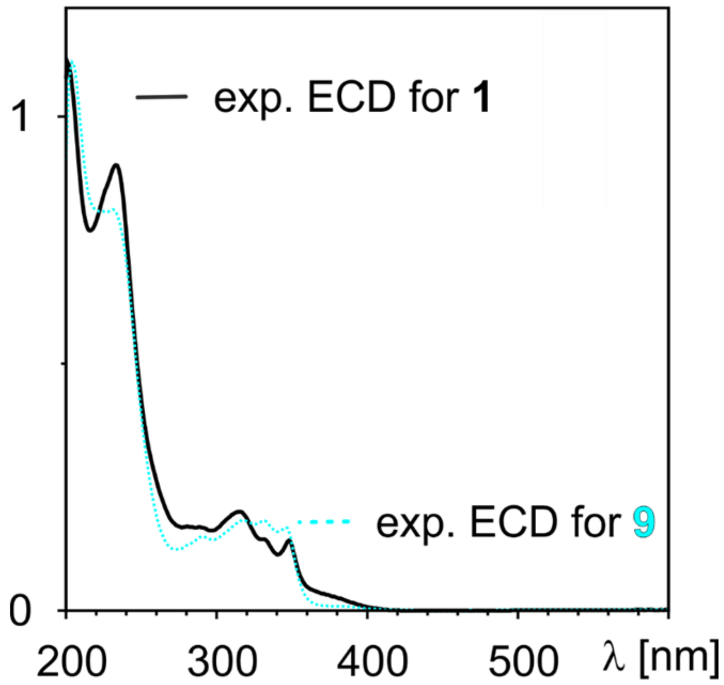

Figure S20. Offline UV spectrum of cyclombandakamine A (**1**) in methanol.

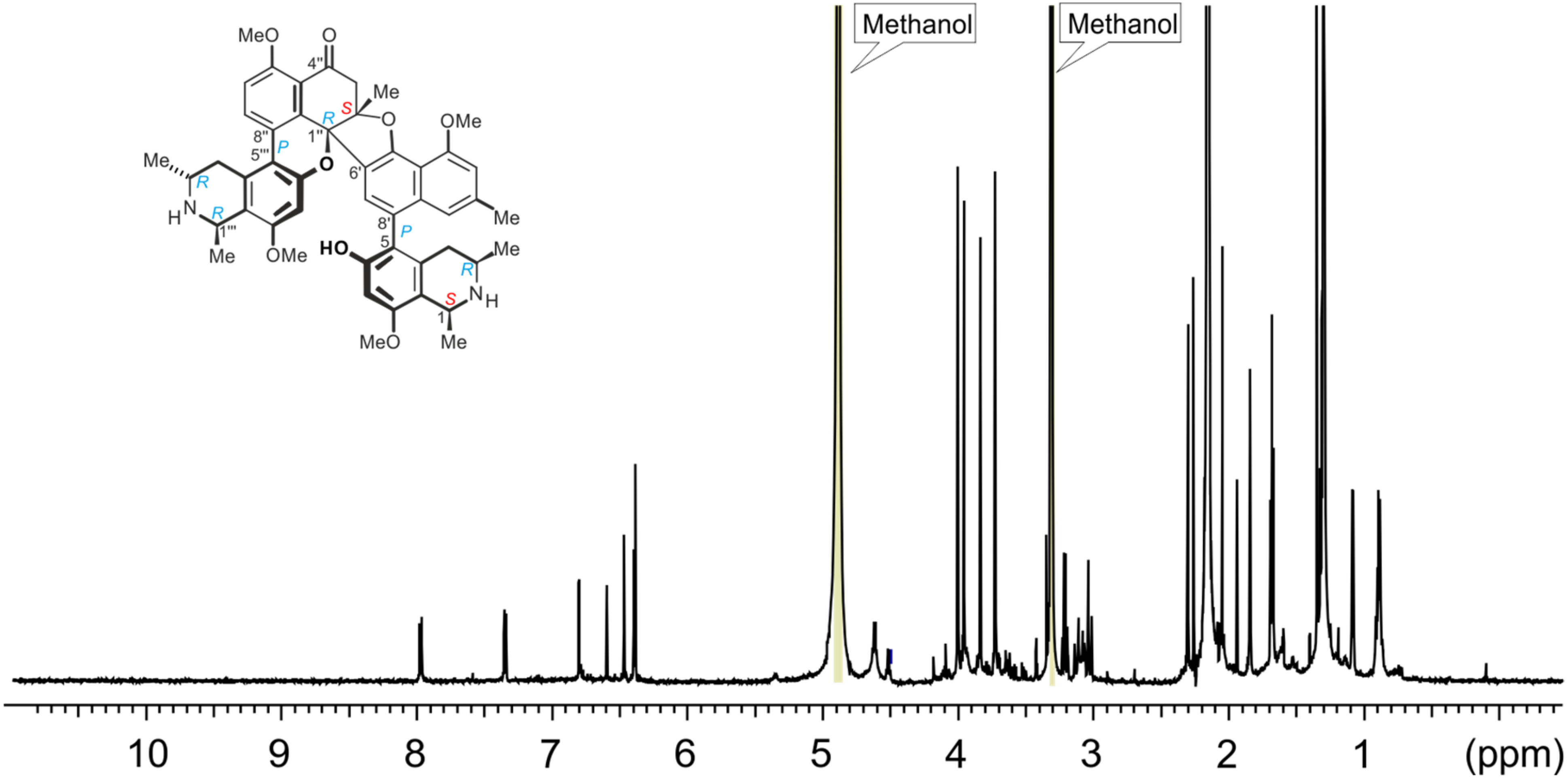

Figure S21a. Overall ^{1}H NMR spectrum of 1-*epi*-cyclombandakamine A (**2**) in methanol-d_4.

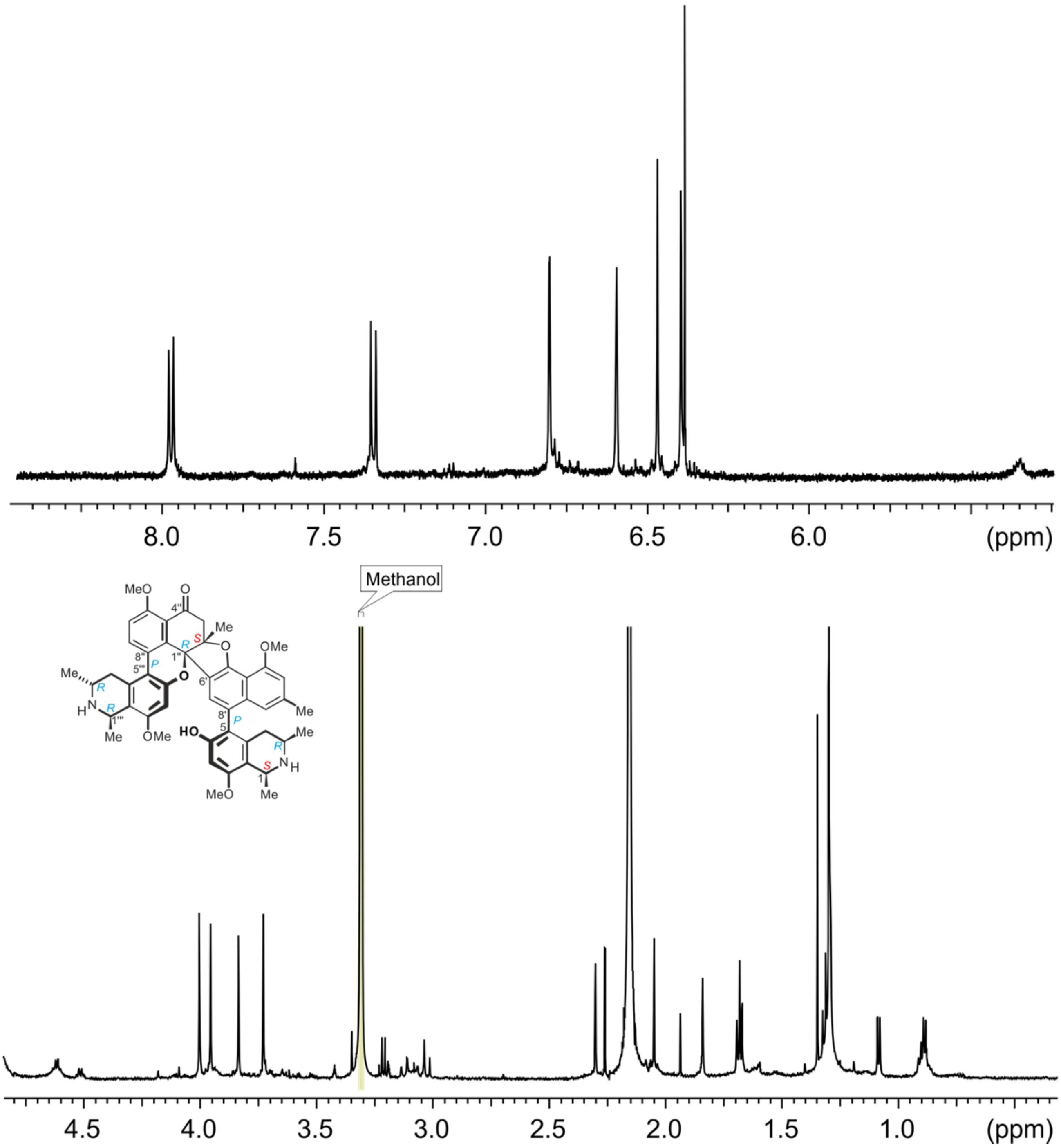

Figure S21b,c. Parts of the ^{1}H NMR spectrum of 1-*epi*-cyclombandakamine A (**2**) in methanol-d_4.

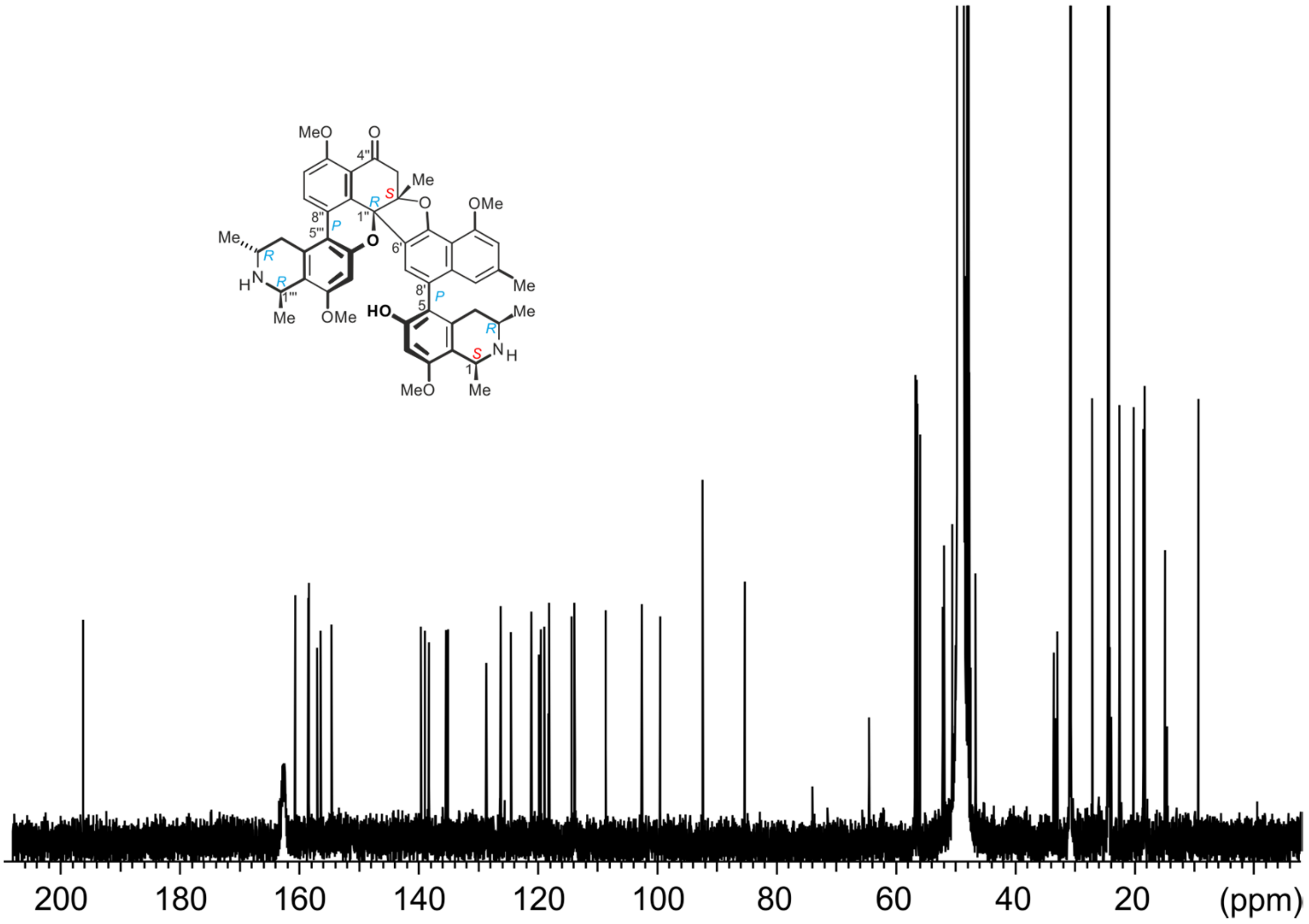

Figure S22a. Overall ^{13}C NMR spectrum of 1-*epi*-cyclombandakamine *A* (**2**) in methanol-d_4.

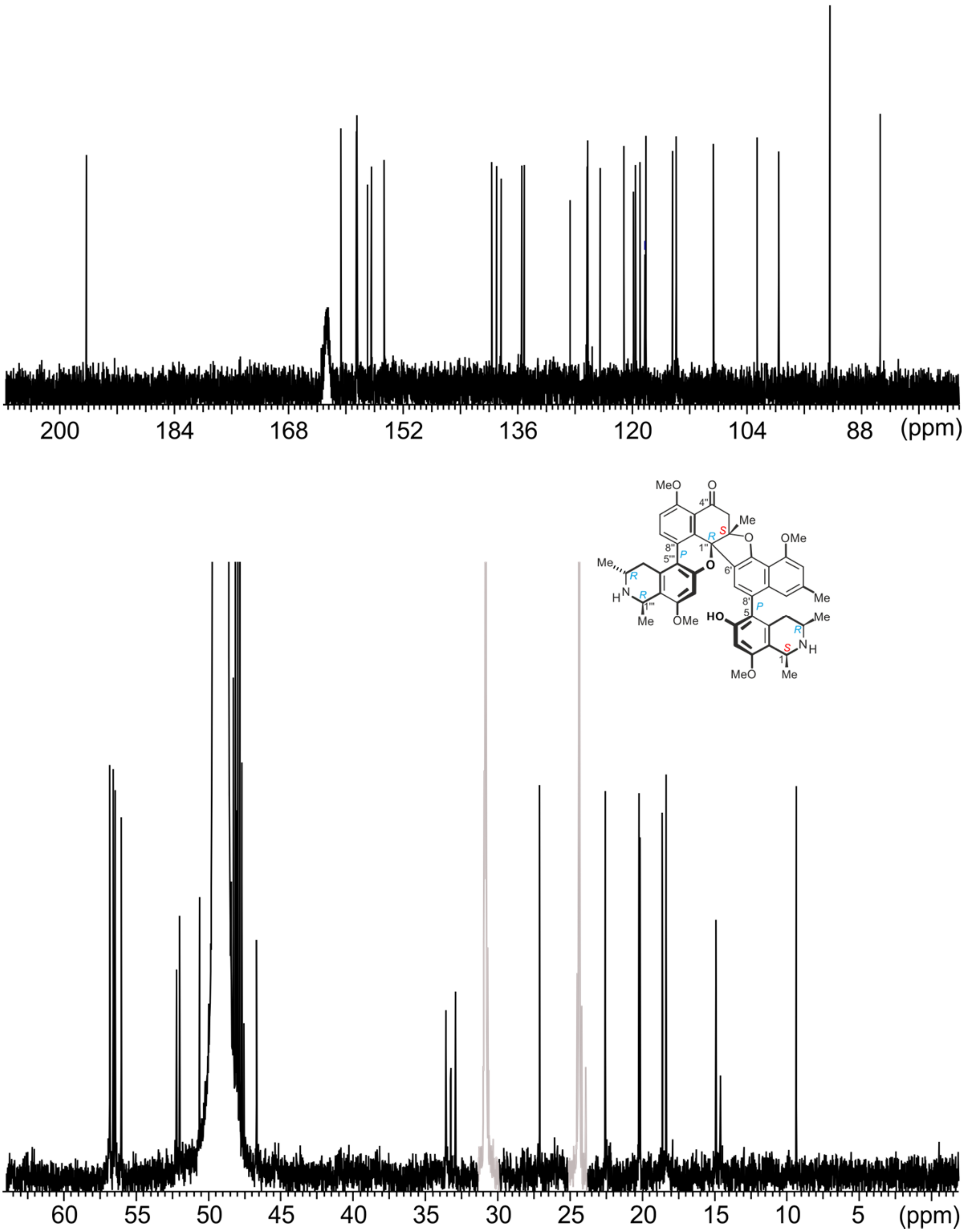

Figure S22b,c. Parts of the ^{13}C NMR spectrum of 1-*epi*-cyclombandakamine A (**2**) in methanol-d_4.

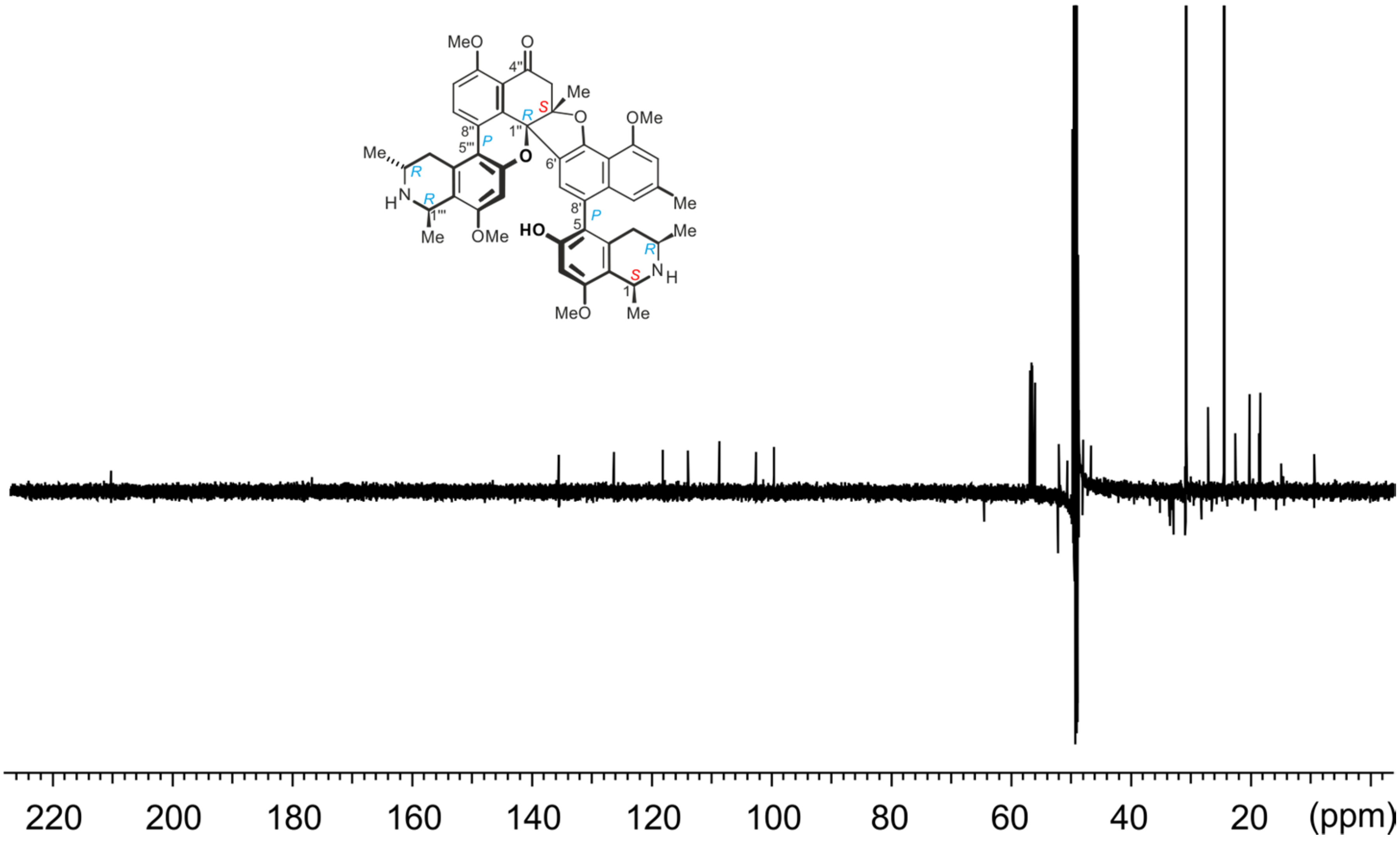

Figure S23. DEPT NMR spectrum of 1-*epi*-cyclombandakamineA (**2**) in methanol-d_4.

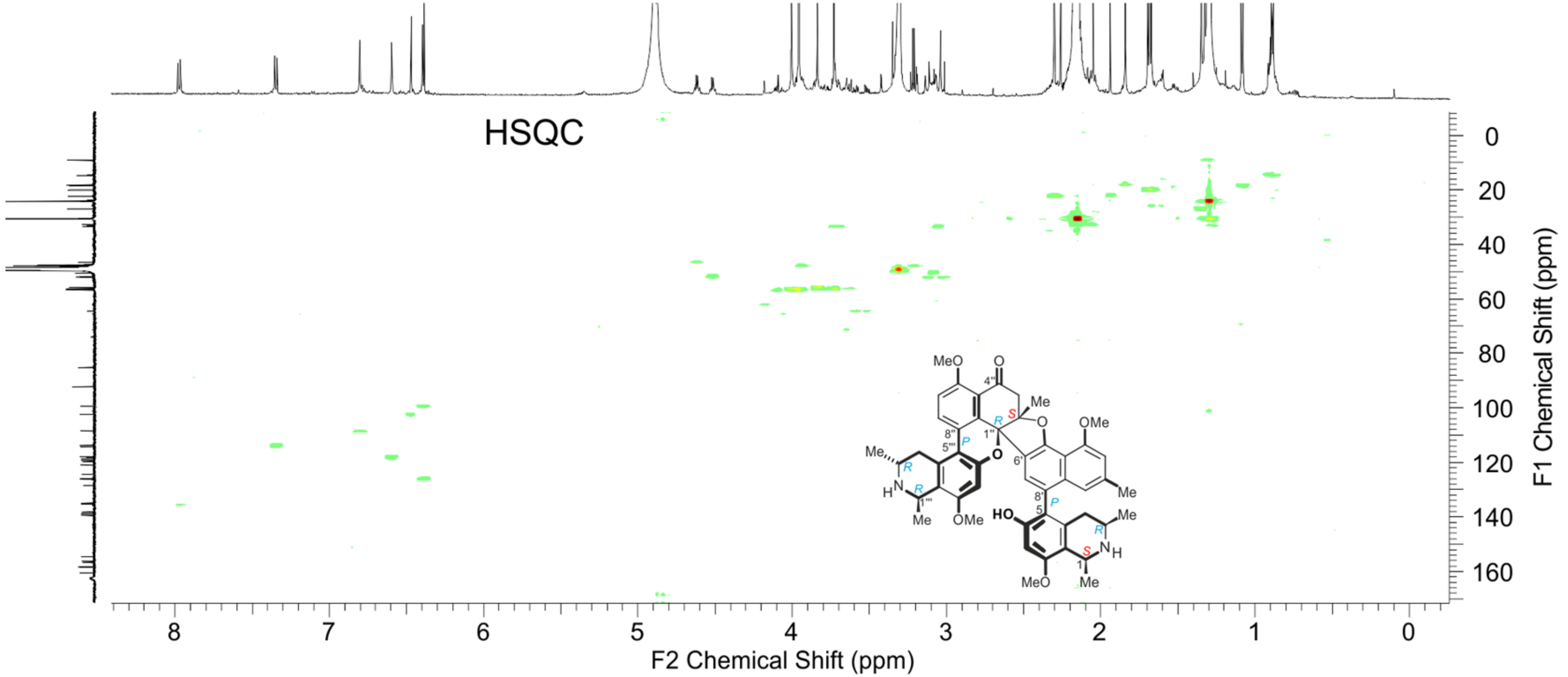

Figure S24. HSQC spectrum of 1-*epi*-cyclombandakamine A (**2**) in methanol-d_4.

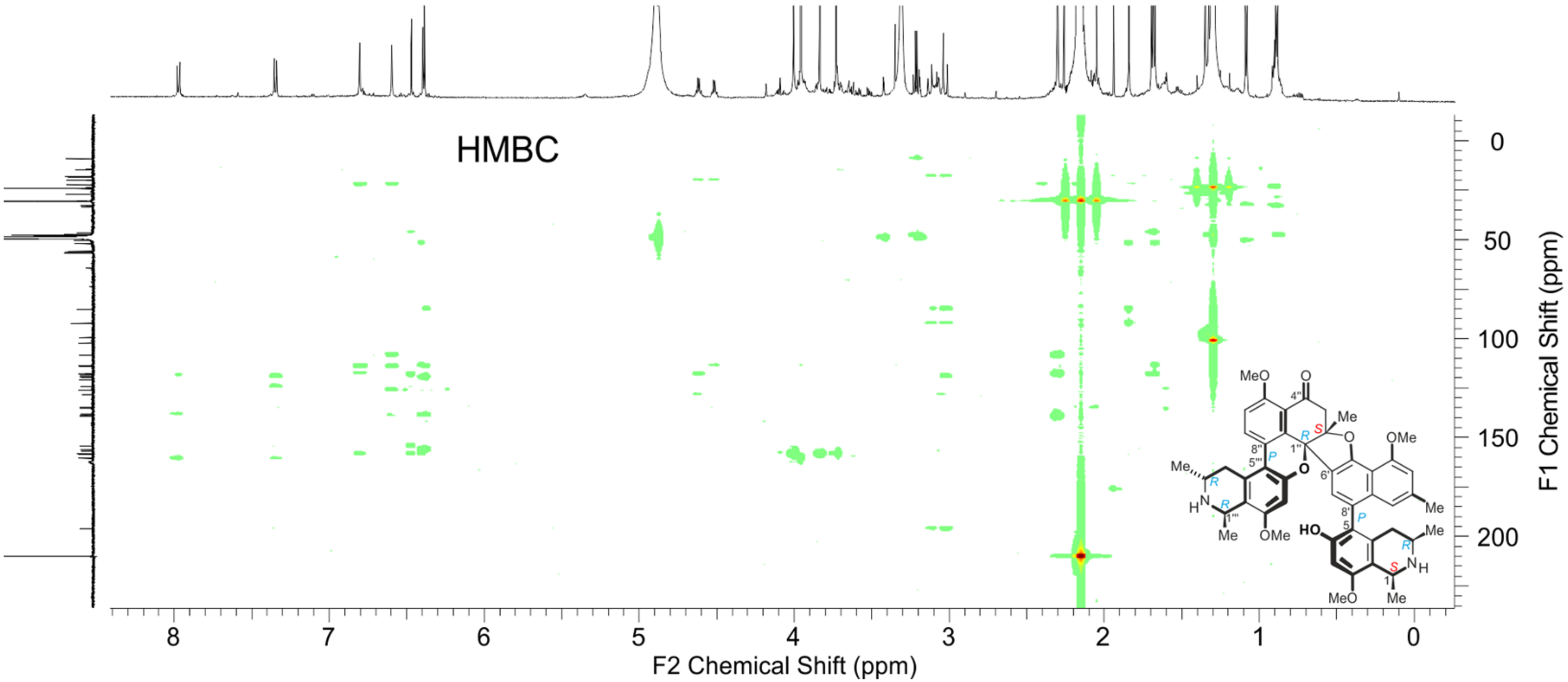

Figure S25. HMBC spectrum of 1-*epi*-cyclombandakamine A (**2**) in methanol-d_4.

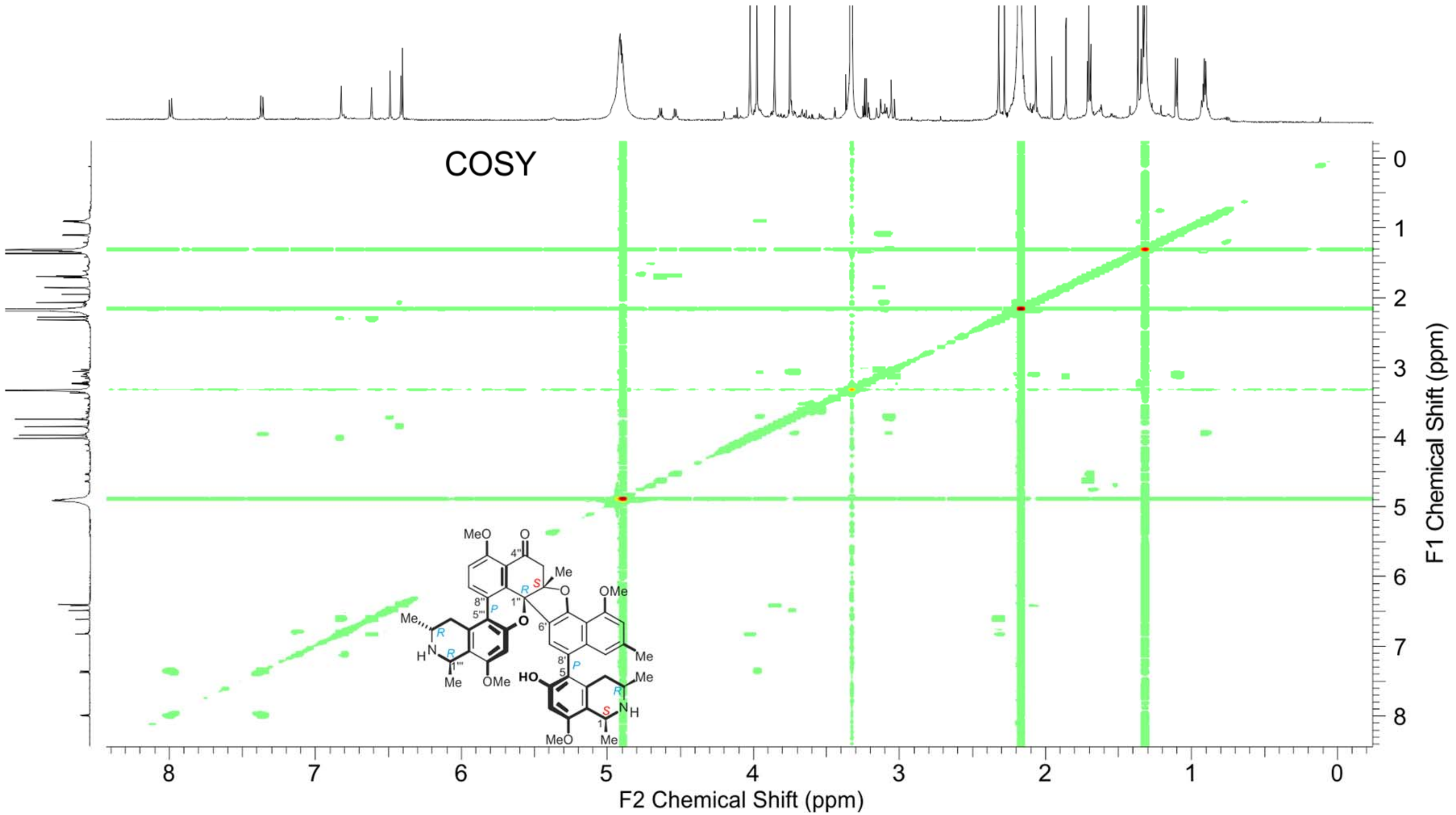

Figure S26. COSY spectrum of 1-*epi*-cyclombandakamine A (**2**) in methanol-d_4.

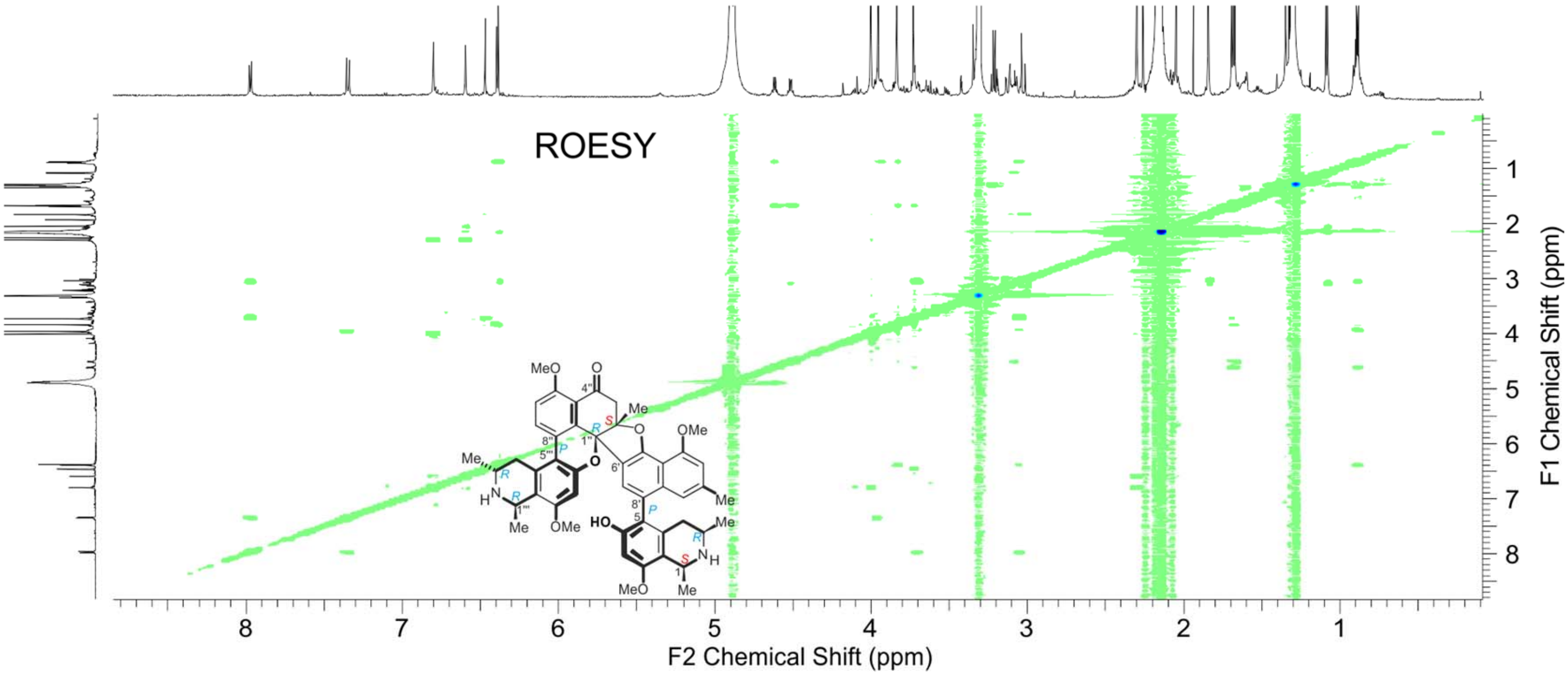

Figure S27a. ROESY spectrum of 1-*epi*-cyclombandakamine A (**2**) in methanol-d_4.

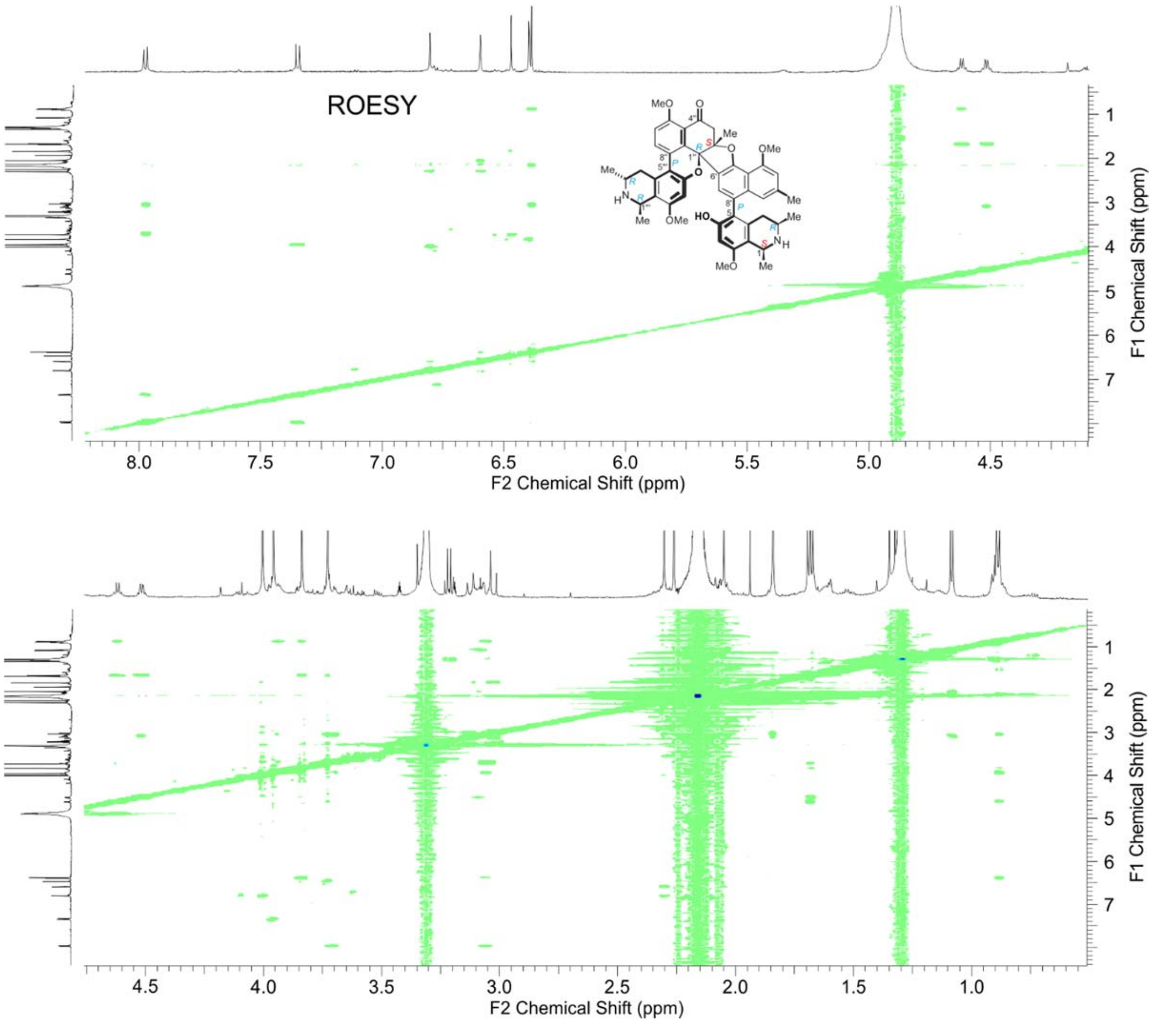

Figure S27b,c. Parts of the ROESY spectrum of 1-*epi*-cyclombandakamine A (**2**) in methanol-d_4.

Analysis Info

Analysis Name	D:\Data\Spektren2016\2016_0415_BRI_2.d	Acquisition Date	19.02.2016 12:45:03
Method	esi_tune_pos_wide.m	Operator	Administrator
Comment	Dieudonne Tshitenge<br>AELV-B-T58-13-PX1<br>unverdünnt (MeOH)	Instrument	micrOTOF 88

Acquisition Parameter

Source Type	ESI	Ion Polarity	Positive	Set Corrector Fill	48 V
Scan Range	n/a	Capillary Exit	280.0 V	Set Pulsar Pull	804 V
Scan Begin	50 m/z	Hexapole RF	380.0 V	Set Pulsar Push	807 V
Scan End	3500 m/z	Skimmer 1	100.0 V	Set Reflector	1700 V
		Hexapole 1	23.0 V	Set Flight Tube	8600 V
				Set Detector TOF	2240 V

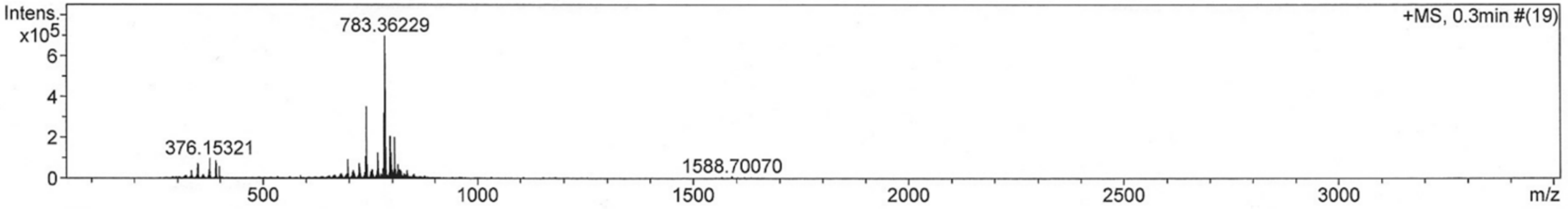

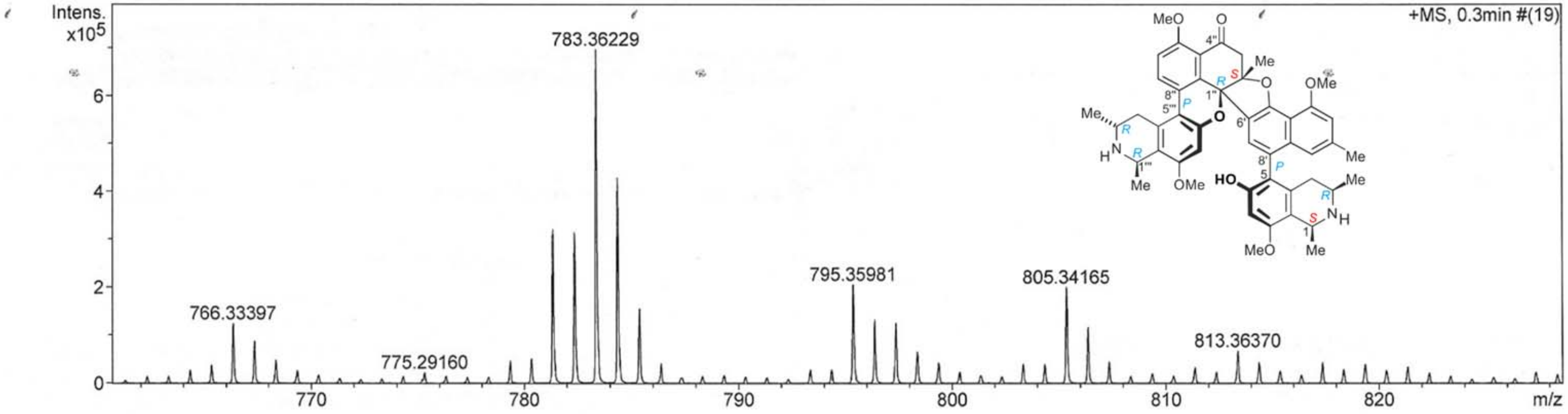

Sum Formula	Sigma	m/z	Err [ppm]	Mean Err [ppm]	rdb	N Rule	e⁻
C 48 H 51 N 2 O 8	0.05	783.36399	2.18	1.48	24.50	ok	even

Figure S28. HRESIMS spectrum 1-*epi*-cyclombandakamine A (**2**) in methanol.

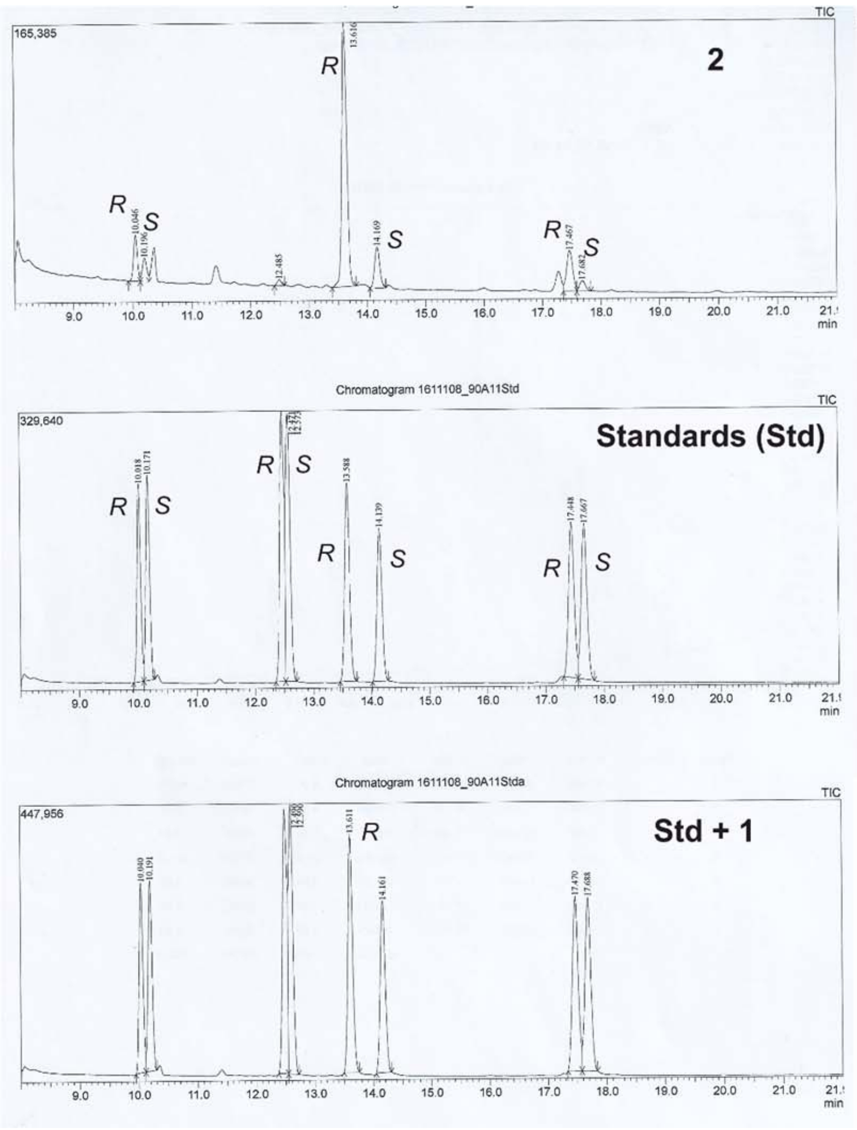

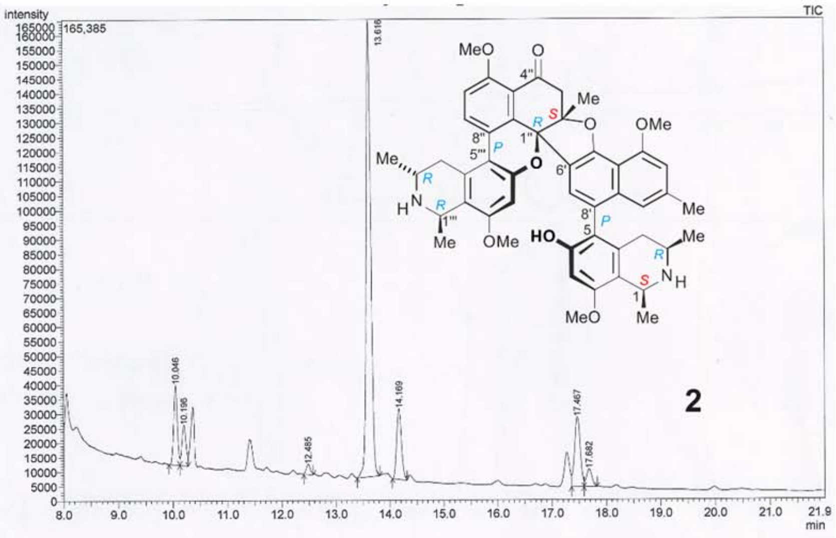

Peak#	Name	R.Time	I.Time	F.Time	Area	Area%	Height	Height%
1		10.046	9.925	10.125	126312	8.30	27630	10.74
2		10.196	10.125	10.275	73234	4.81	13951	5.42
3		12.485	12.405	12.580	18916	1.24	3605	1.40
4		13.616	13.395	13.810	934983	61.42	157236	61.12
5		14.169	14.045	14.315	150521	9.89	24496	9.52
6		17.467	17.360	17.590	175074	11.50	24081	9.36
7		17.682	17.590	17.830	43323	2.85	6248	2.43
					1522363	100.00	257247	100.00

Figure S29. Oxidative degradation results of 1-*epi*-cyclombandakamine A (**2**).

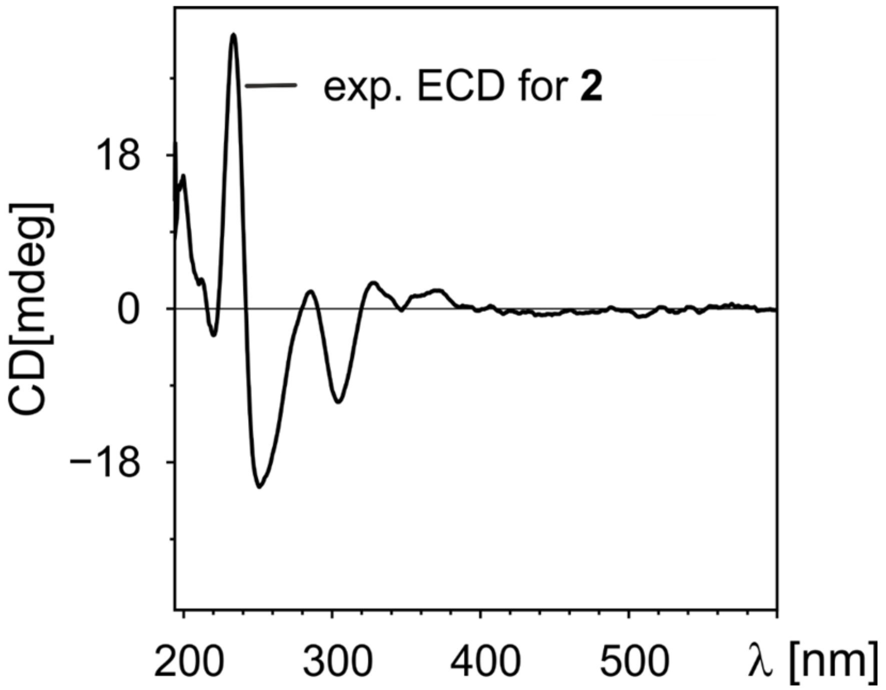

Figure S30. ECD spectrum of 1-*epi*-cyclombandakamine A (**2**) in methanol, compared to the one of **4**.

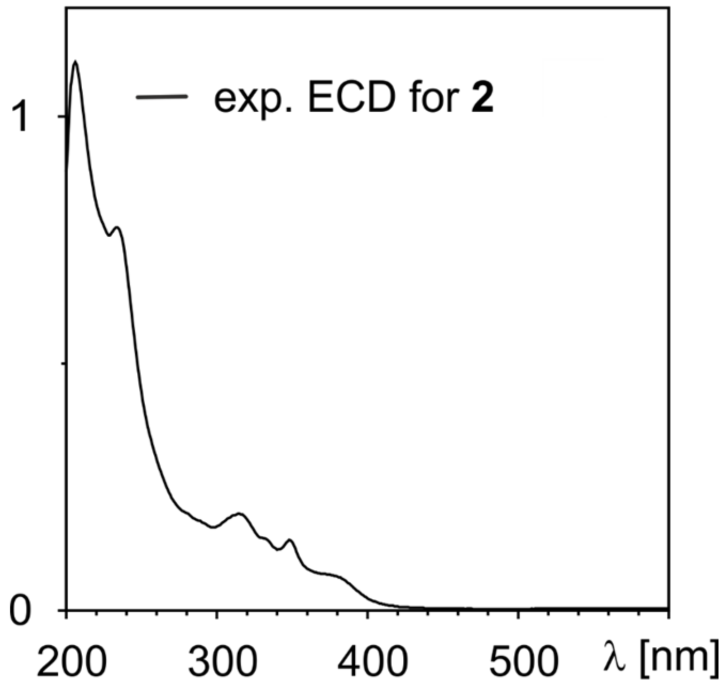

Figure 31. Offline UV spectrum of 1-*epi*-cyclombandakamine A (**2**) in methanol.

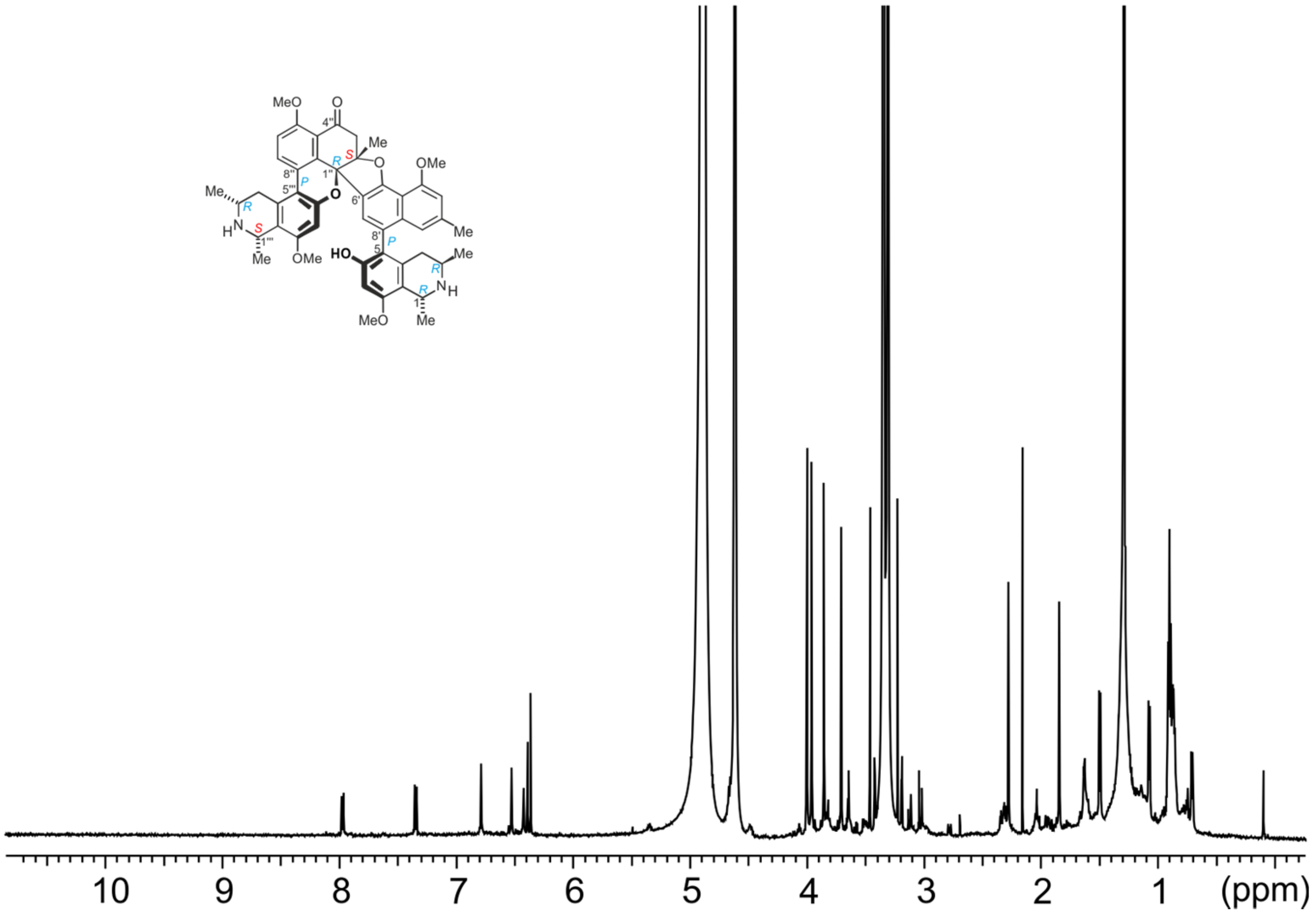

Figure S32a. Overall ^{1}H NMR spectrum of cyclombandakamine A_3 **(3)** in methanol-d_4.

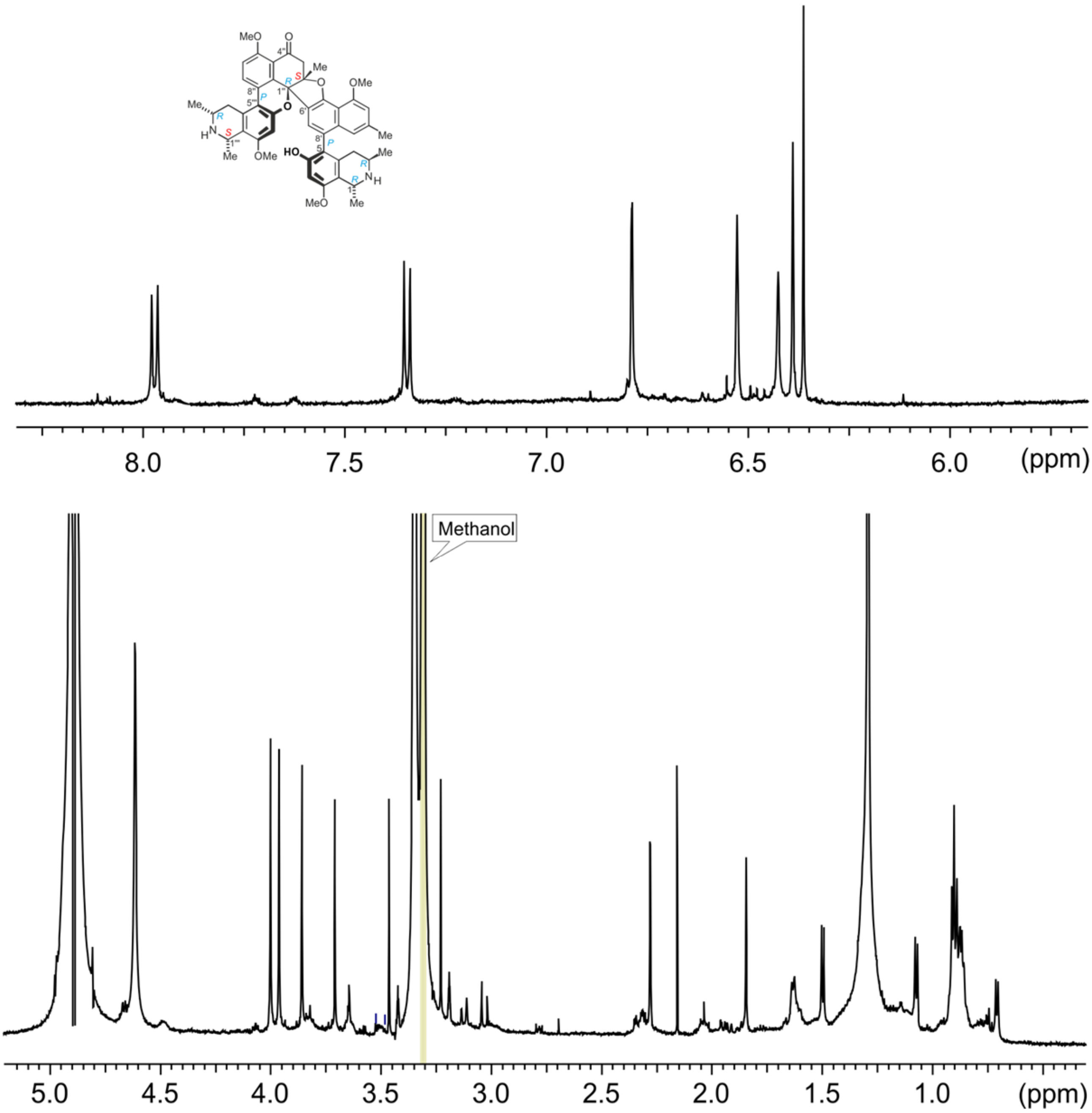

Figure S32b,c. Parts of the ^{1}H NMR spectrum of cyclombandakamine A_3 **(3)** in methanol-d_4.

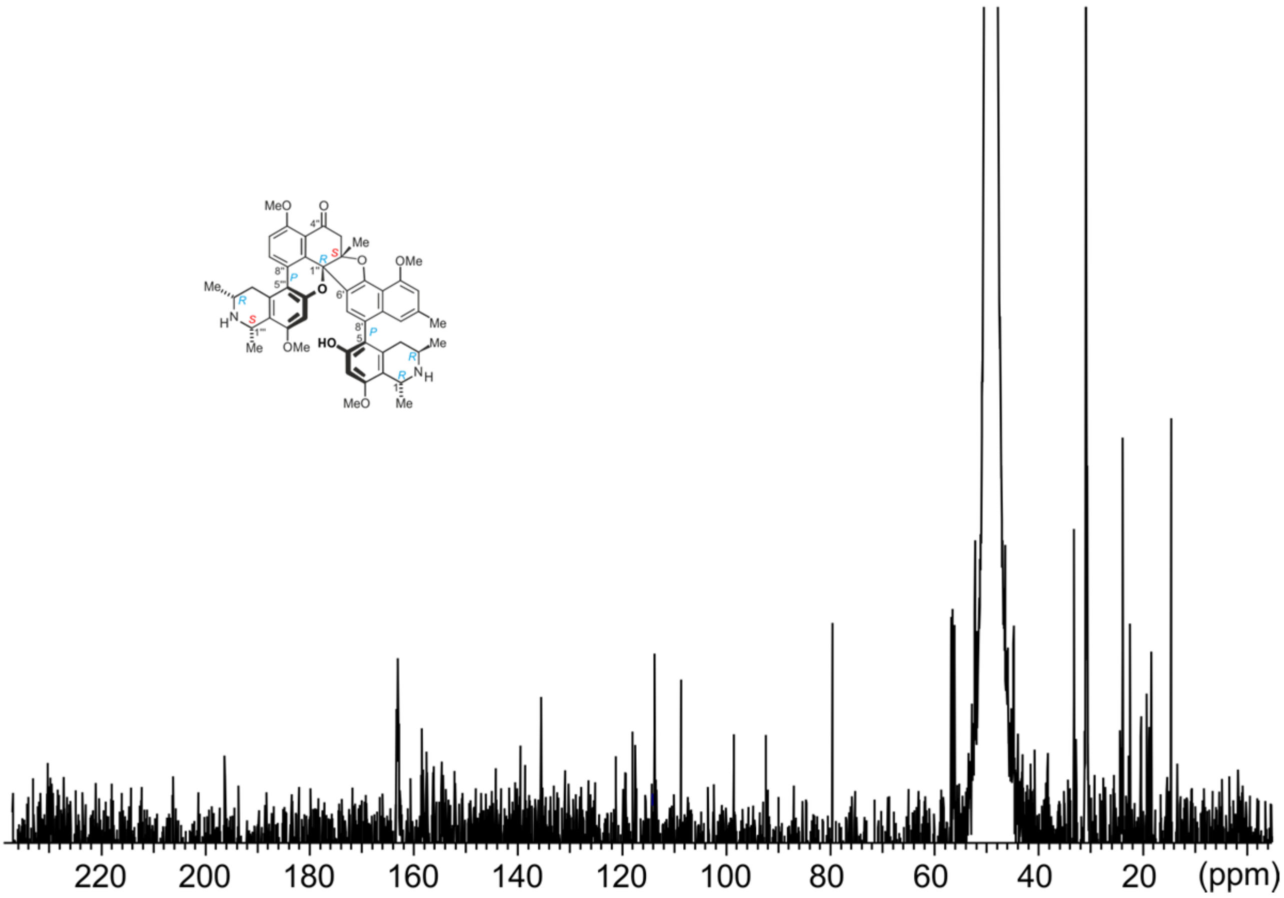

Figure S33. ^{13}C NMR spectrum of cyclombandakamine A$_3$ **(3)** in methanol-d_4.

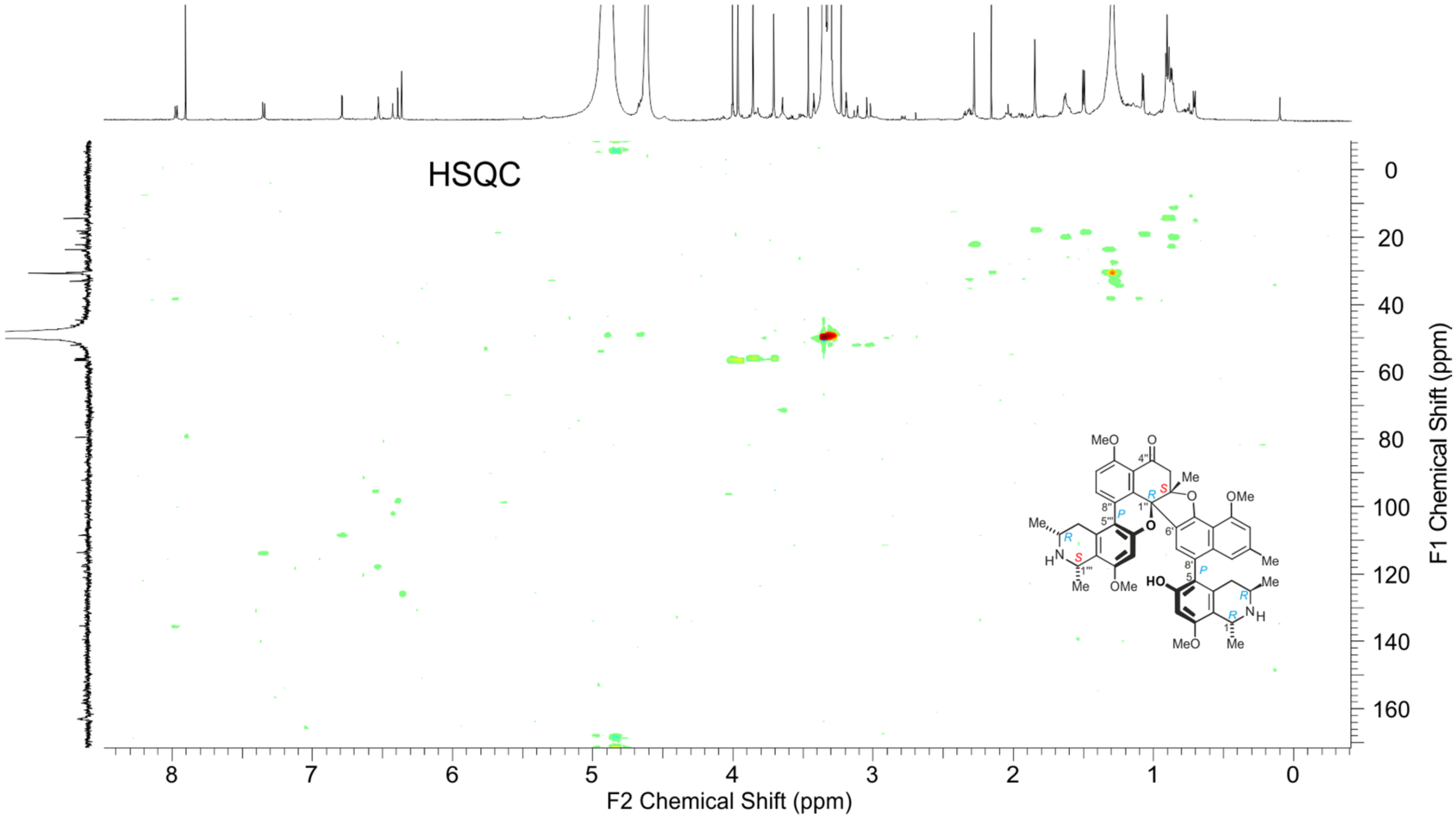

Figure S34. Overall HSQC spectrum of cyclombandakamine A_3 **(3)** in methanol-d_4.

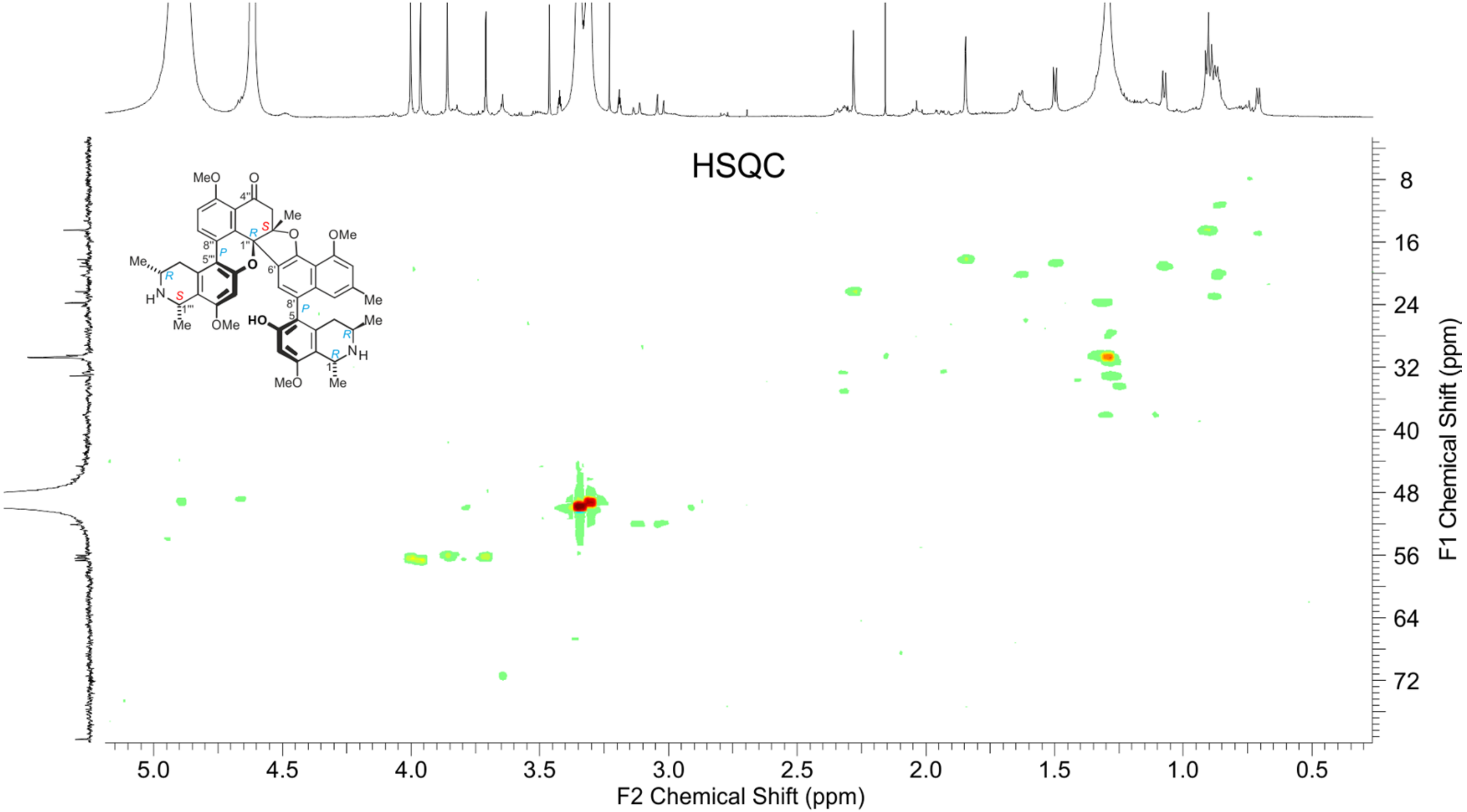

Figure S34b. Aliphatic part of the HSQC spectrum of cyclombandakamine A_3 **(3)** in methanol-d_4.

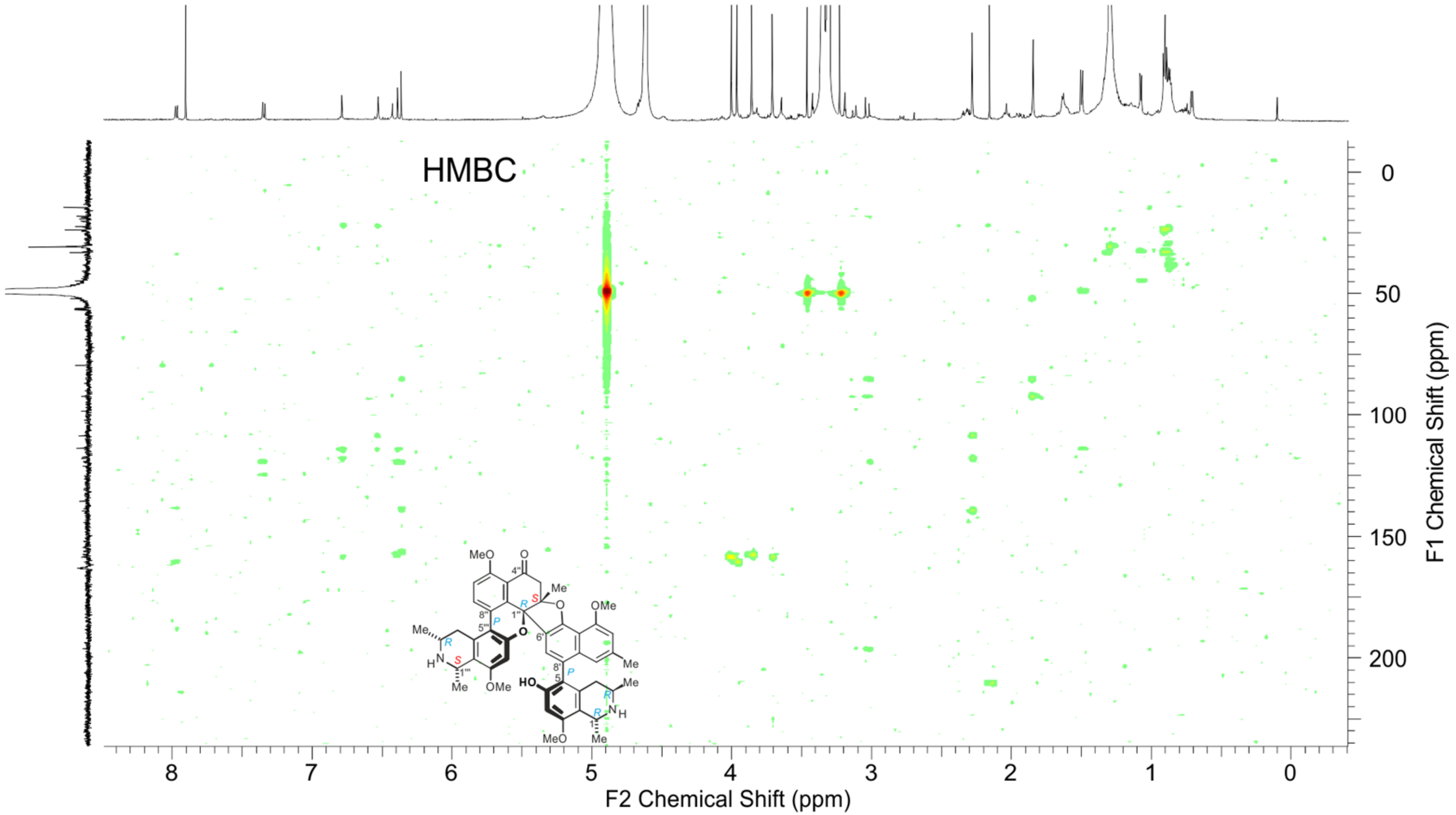

Figure S35. HMBC spectrum of cyclombandakamine A_3 **(3)** in methanol-d_4.

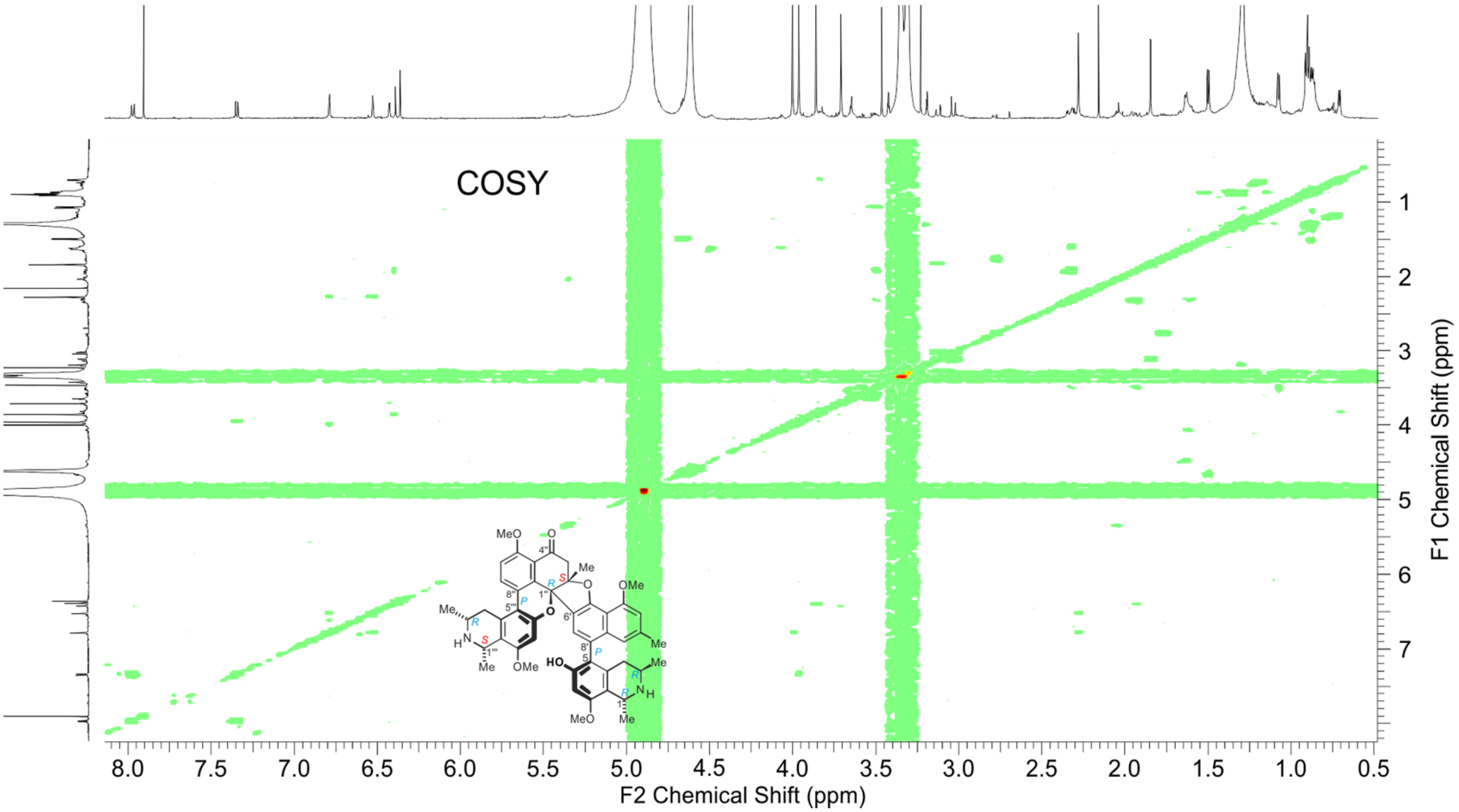

Figure S36a. COSY spectrum of cyclombandakamine A_3 **(3)** in methanol-d_4.

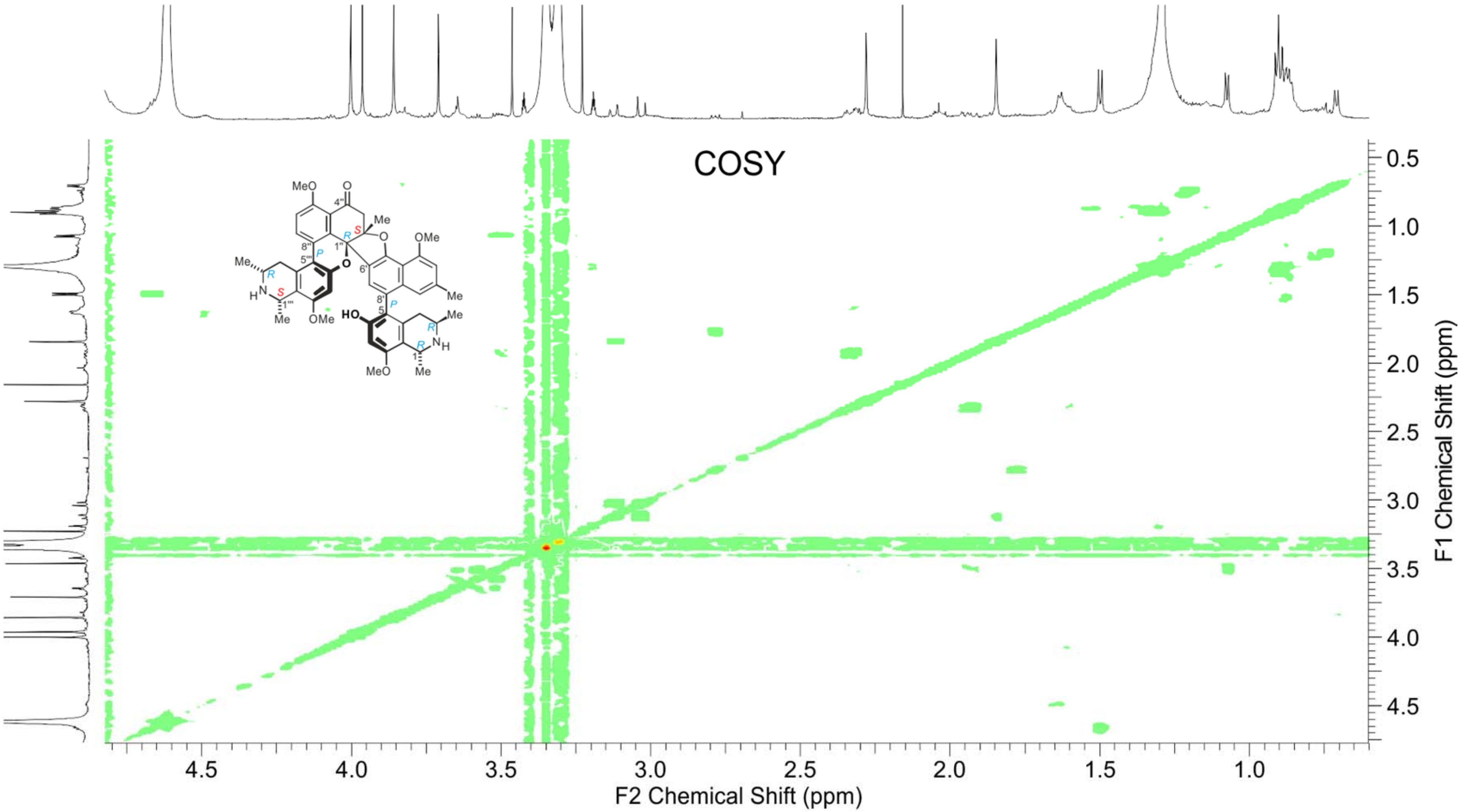

Figure S36b. Aliphatic part of the COSY spectrum of cyclombandakamine A_3 **(3)** in methanol-d_4.

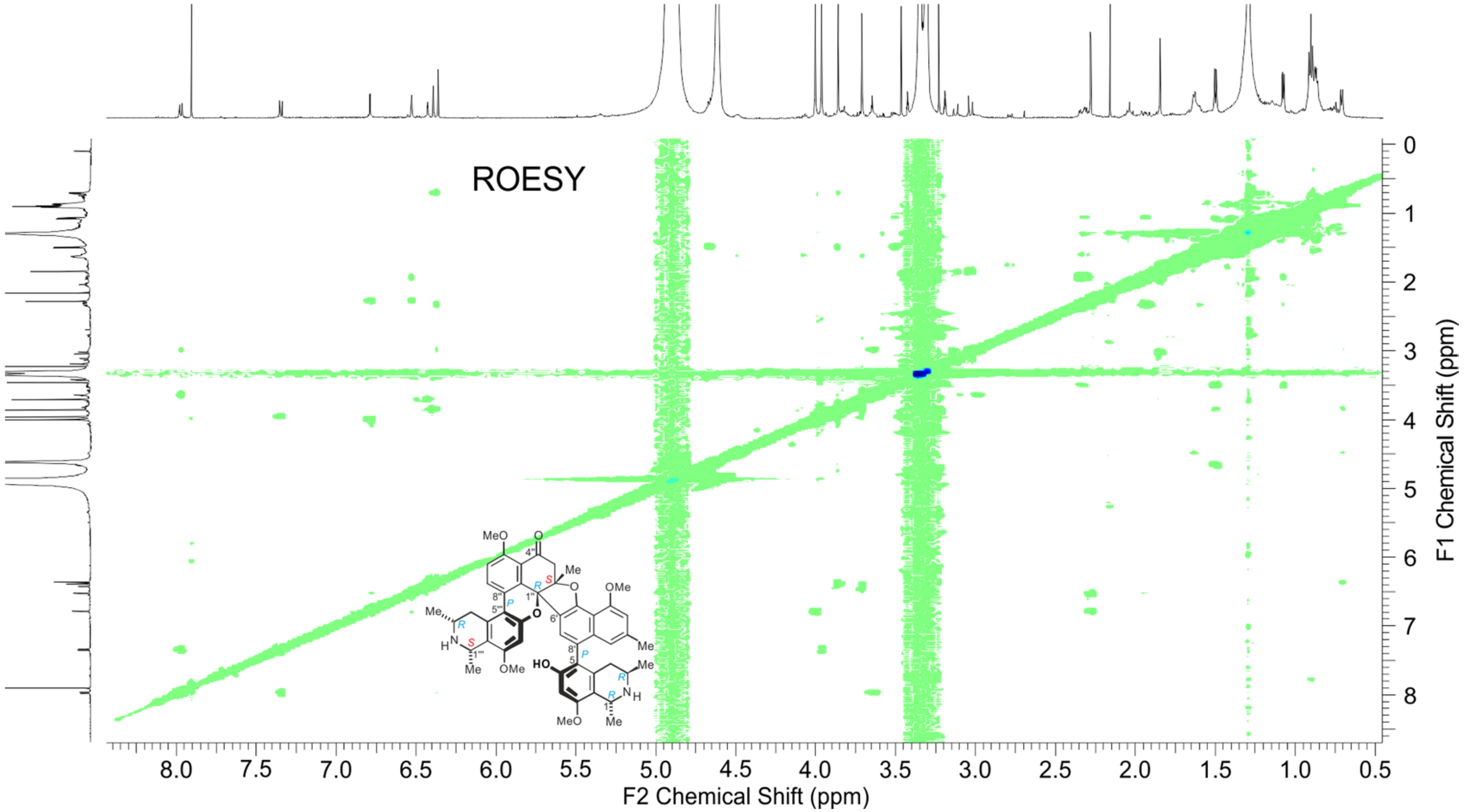

Figure S37. ROESY spectrum of cyclombandakamine A$_3$ **(3)** in methanol-d_4.

Bruker Daltonics DataAnalysis 3.3

Mass Spectrum Molecular Formula Report

Analysis Info

Analysis Name	D:\Data\Spektren2016\2016_0414_BRI_2.d	Acquisition Date	19.02.2016 11:27:17
Method	esi_tune_pos_wide.m	Operator	Administrator
Comment	Dieudonne Tshitenge<br>AELV-B-T58-12-P1<br>undiluted (MeOH)	Instrument	micrOTOF 88

Acquisition Parameter

Source Type	ESI	Ion Polarity	Positive	Set Corrector Fill	48 V
Scan Range	n/a	Capillary Exit	280.0 V	Set Pulsar Pull	804 V
Scan Begin	50 m/z	Hexapole RF	380.0 V	Set Pulsar Push	807 V
Scan End	3500 m/z	Skimmer 1	100.0 V	Set Reflector	1700 V
		Hexapole 1	23.0 V	Set Flight Tube	8600 V
				Set Detector TOF	2240 V

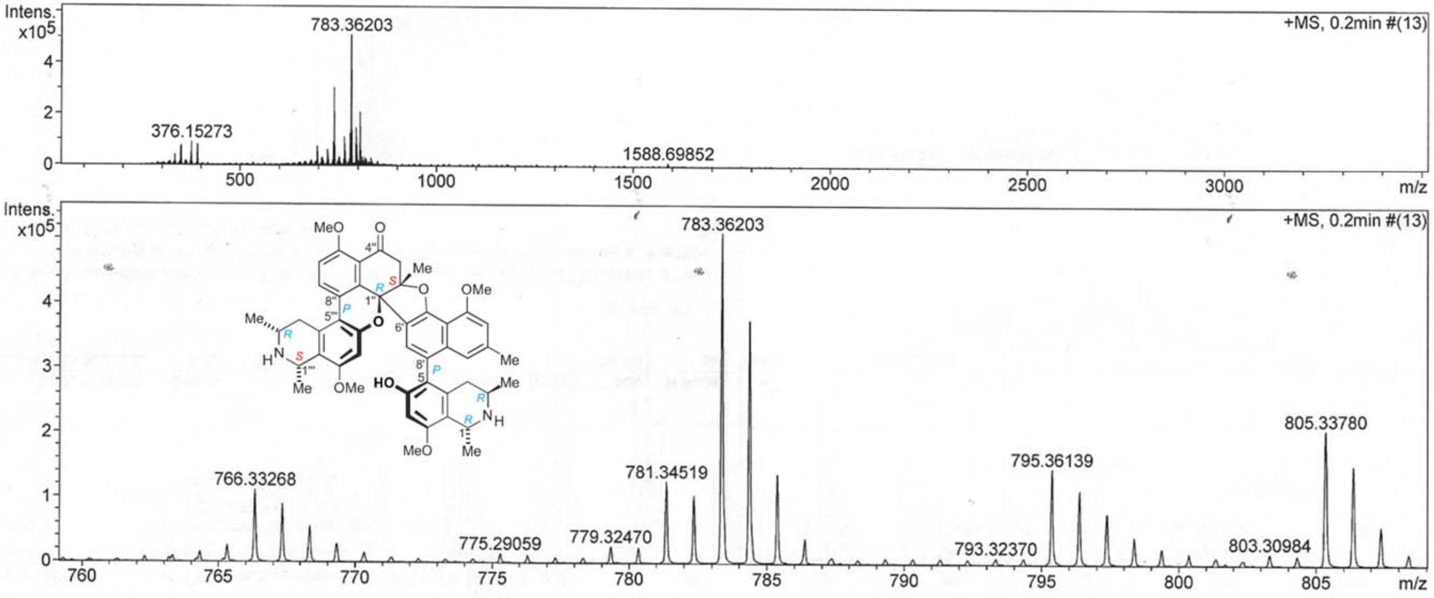

Sum Formula	Sigma	m/z	Err [ppm]	Mean Err [ppm]	rdb	N Rule	e⁻
C 48 H 51 N 2 O 8	0.12	783.36399	2.50	1.17	24.50	ok	even

Figure S38. HRESIMS spectrum of cyclombandakamine A_3 (**3**).

ieudonne Tshitenge - AELV-B-T58-12-P1; Matrix: SDHB in MeOH 1:3

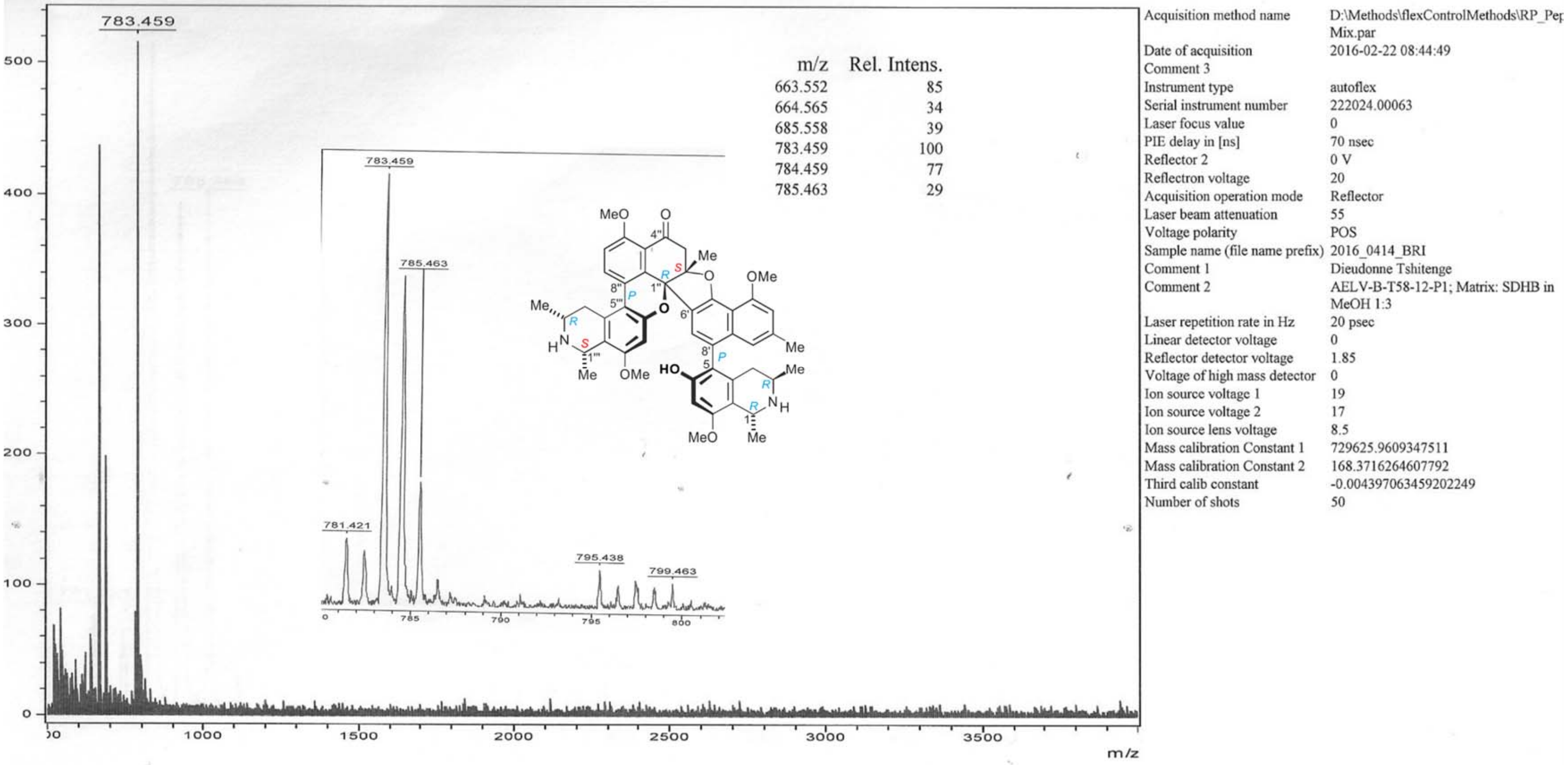

Acquisition method name	D:\Methods\flexControlMethods\RP_Pep Mix.par
Date of acquisition	2016-02-22 08:44:49
Comment 3	
Instrument type	autoflex
Serial instrument number	222024.00063
Laser focus value	0
PIE delay in [ns]	70 nsec
Reflector 2	0 V
Reflectron voltage	20
Acquisition operation mode	Reflector
Laser beam attenuation	55
Voltage polarity	POS
Sample name (file name prefix)	2016_0414_BRI
Comment 1	Dieudonne Tshitenge
Comment 2	AELV-B-T58-12-P1; Matrix: SDHB in MeOH 1:3
Laser repetition rate in Hz	20 psec
Linear detector voltage	0
Reflector detector voltage	1.85
Voltage of high mass detector	0
Ion source voltage 1	19
Ion source voltage 2	17
Ion source lens voltage	8.5
Mass calibration Constant 1	729625.9609347511
Mass calibration Constant 2	168.3716264607792
Third calib constant	-0.004397063459202249
Number of shots	50

ıker Daltonics flexAnalysis printed: 02/22/2016 08:49:27 AM

Figure S39. MALDI analysis: profile of cyclombandakamine A_3 (**3**).

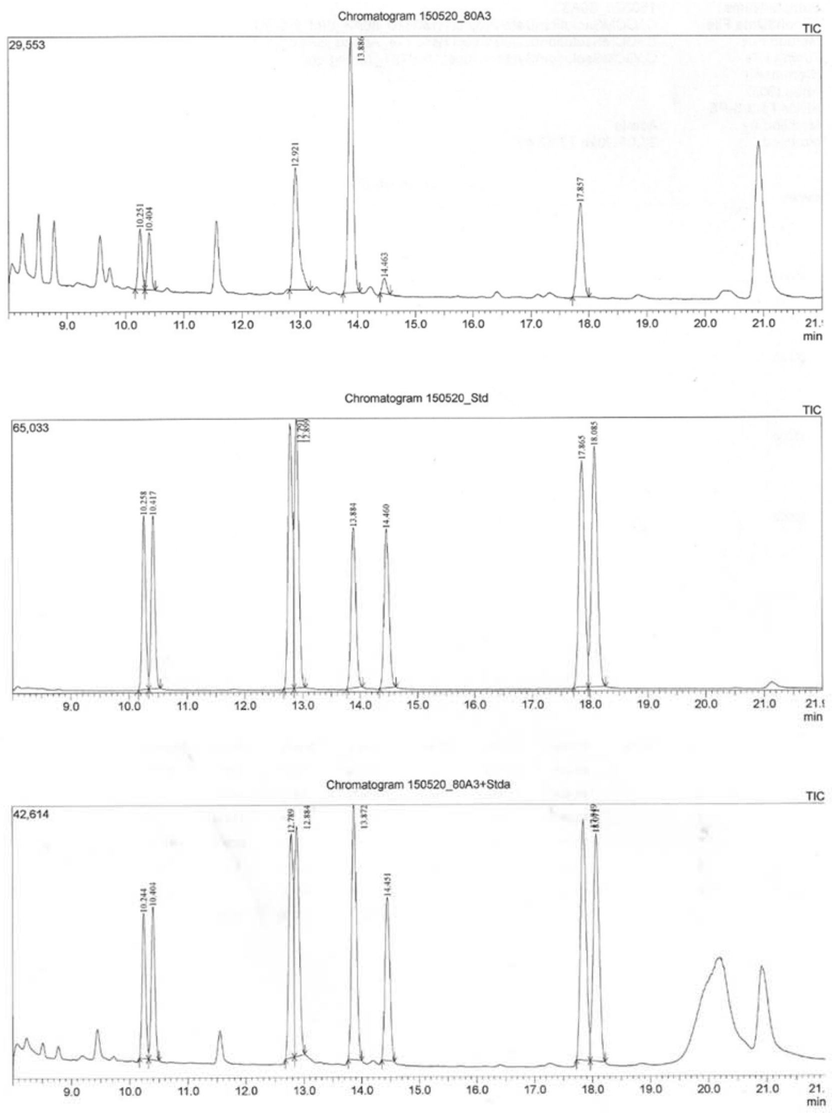

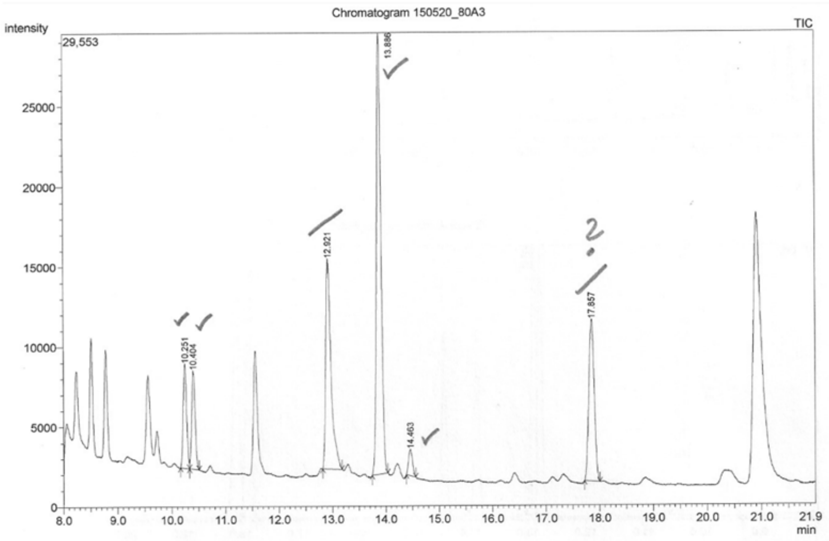

Peak#	Name	R.Time	I.Time	F.Time	Area	Area%	Height	Height%
1		10.251	10.165	10.330	28249	7.30	6527	10.01
2		10.404	10.330	10.510	27072	7.00	6160	9.45
3		12.921	12.820	13.180	90636	23.43	13139	20.15
4		13.886	13.745	14.035	161681	41.80	27566	42.28
5		14.463	14.385	14.560	8512	2.20	1684	2.58
6		17.857	17.720	18.005	70625	18.26	10116	15.52
					386775	100.00	65192	100.00

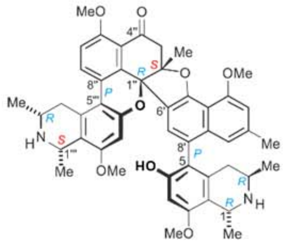

Figure S40. Oxidative degradation products of cyclombandakamine A_3 (**3**).

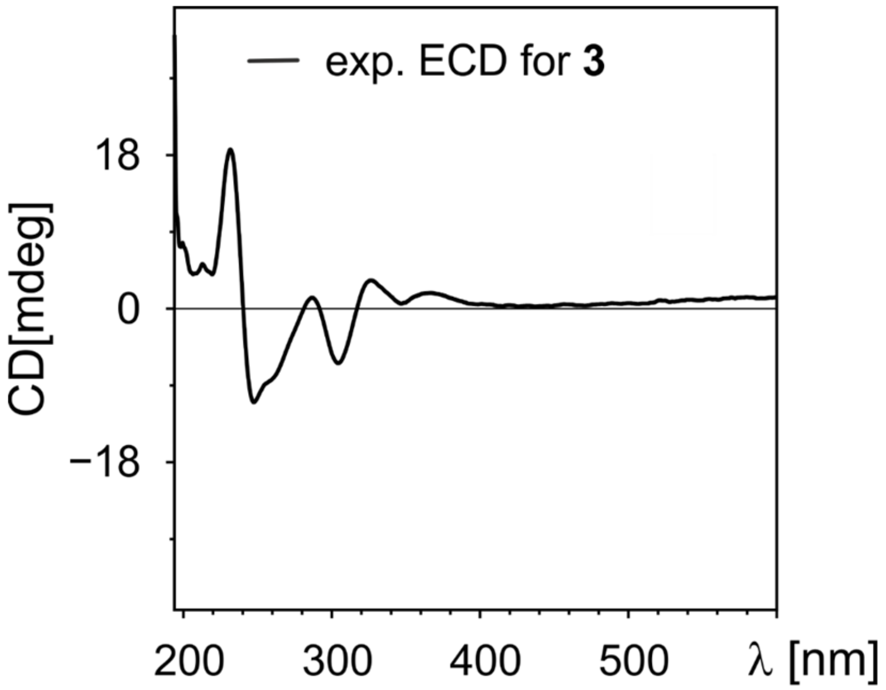

Figure S41. ECD spectrum of cyclombandakamine A_3 (**3**) in methanol, compared to the one of **4**.

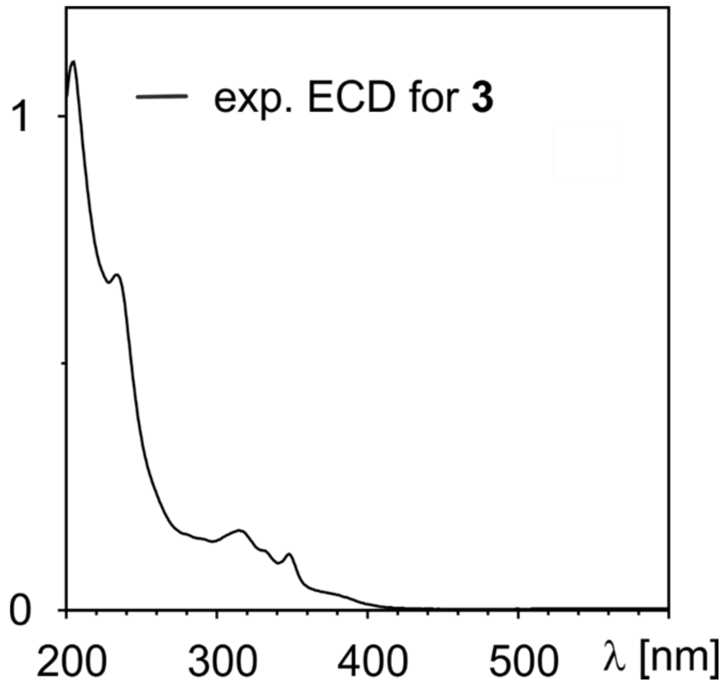

Figure S42. Offline UV spectrum of cyclombandakamine A_3 (**3**) in methanol.

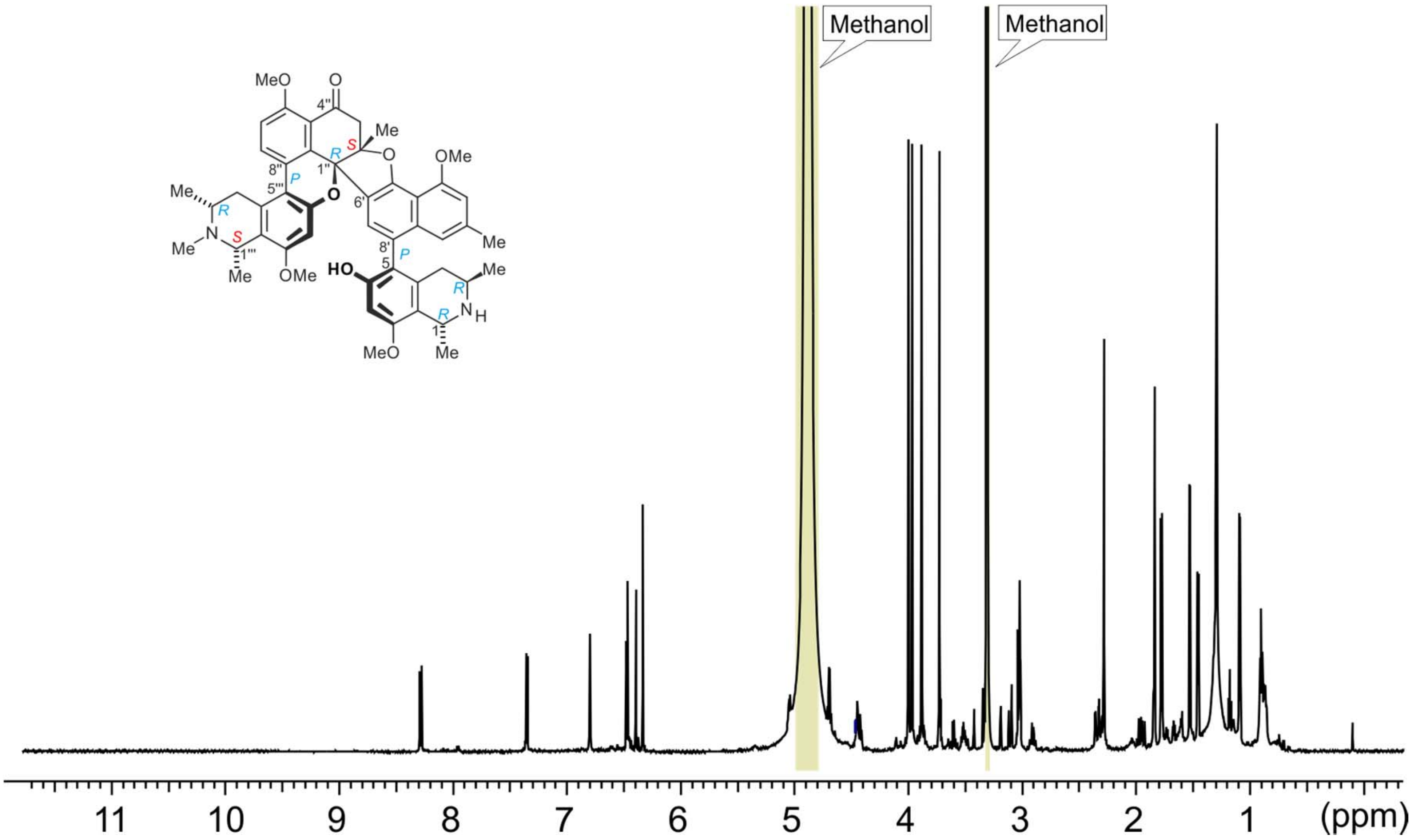

Figure S43a. Overall ^{1}H NMR spectrum of cyclombandakamine A_4 (**4**) in methanol-d_4.

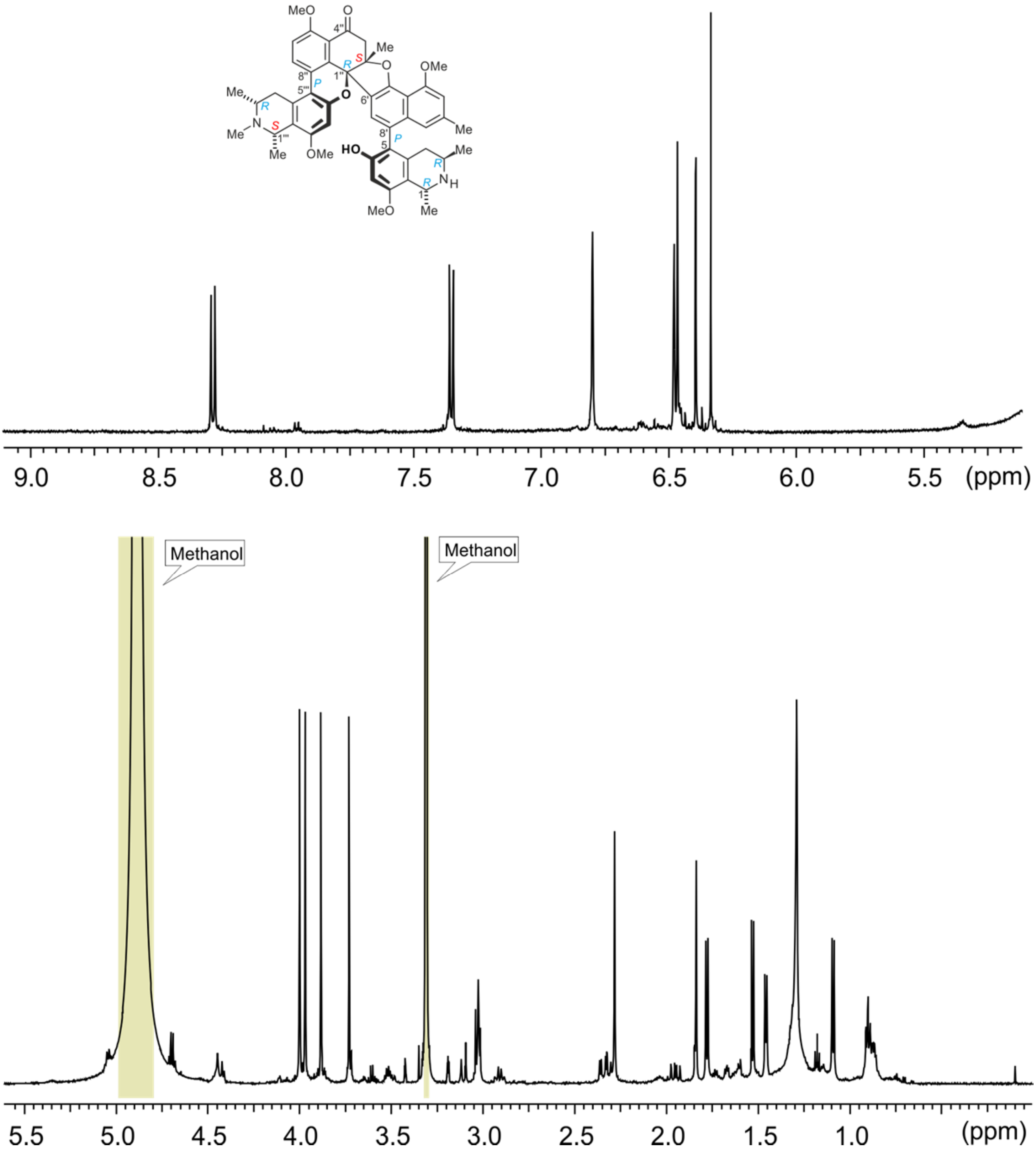

Figure S43b,c. Parts of the ^{1}H NMR spectrum of cyclombandakamine A_4 (**4**) in methanol-d_4.

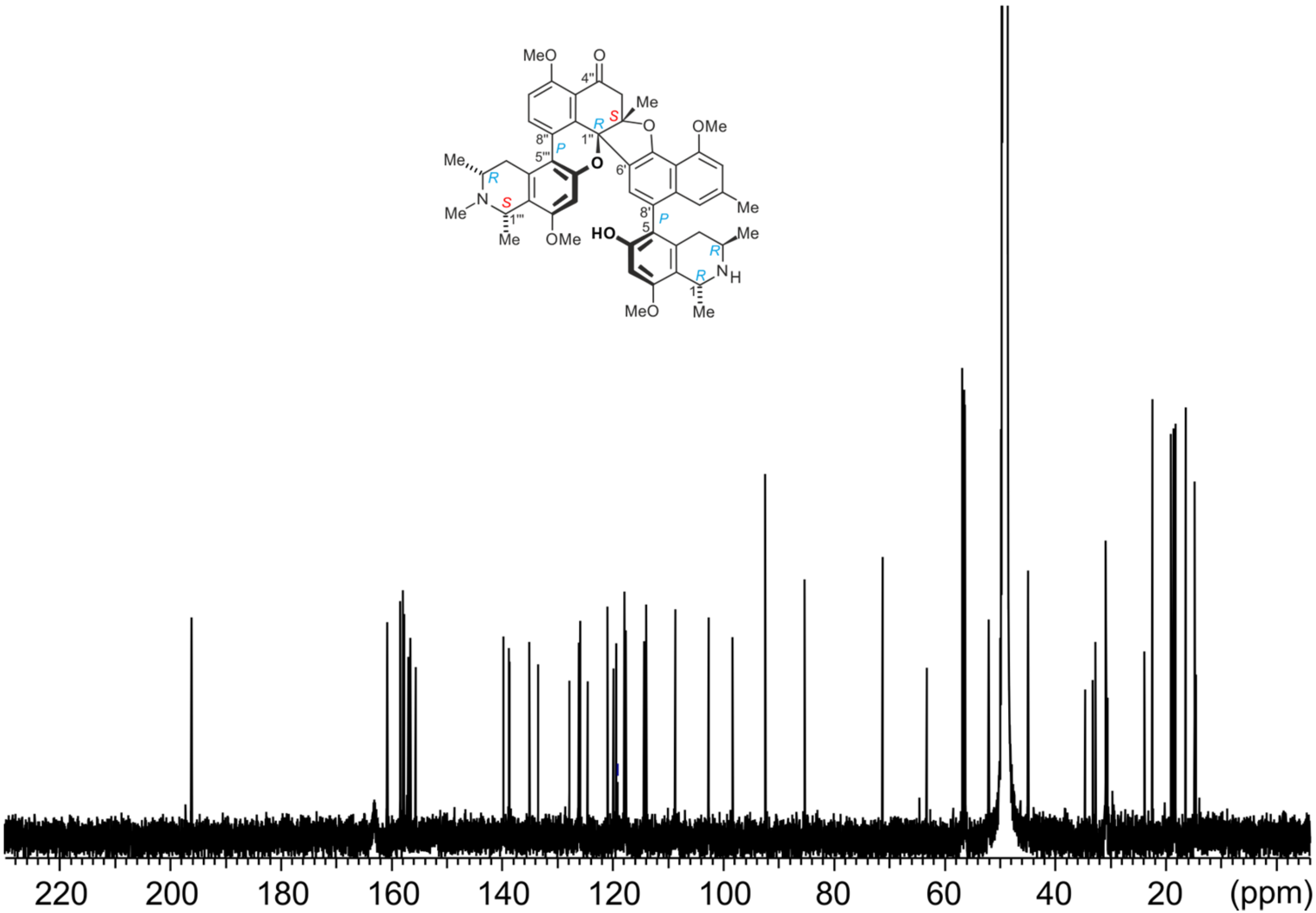

Figure S44a. ^{13}C NMR spectrum of cyclombandakamine A$_4$ **(4)** in methanol-d_4.

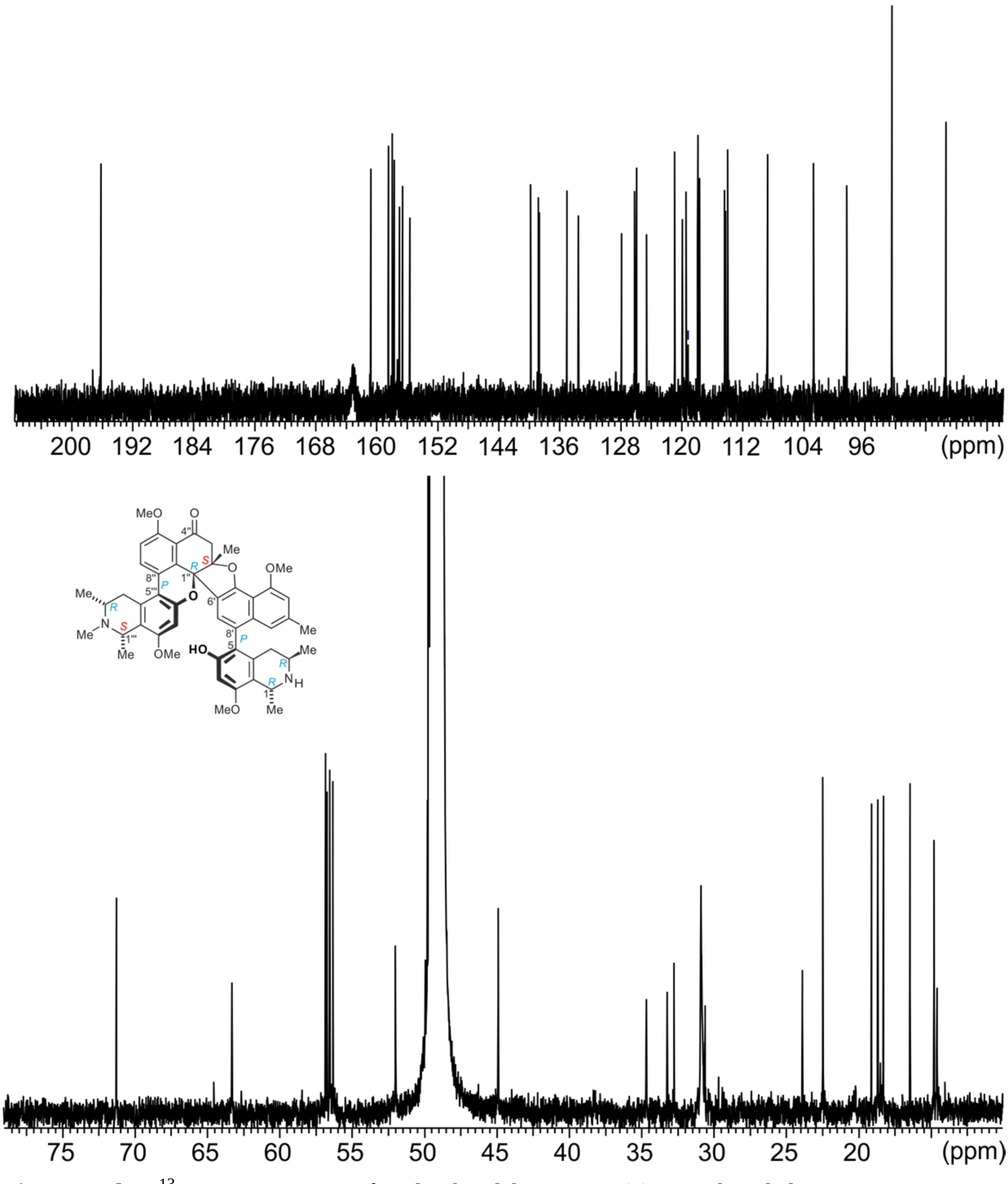

Figure S44b,c. ^{13}C NMR spectrum of cyclombandakamine A$_4$ **(4)** in methanol-d_4.

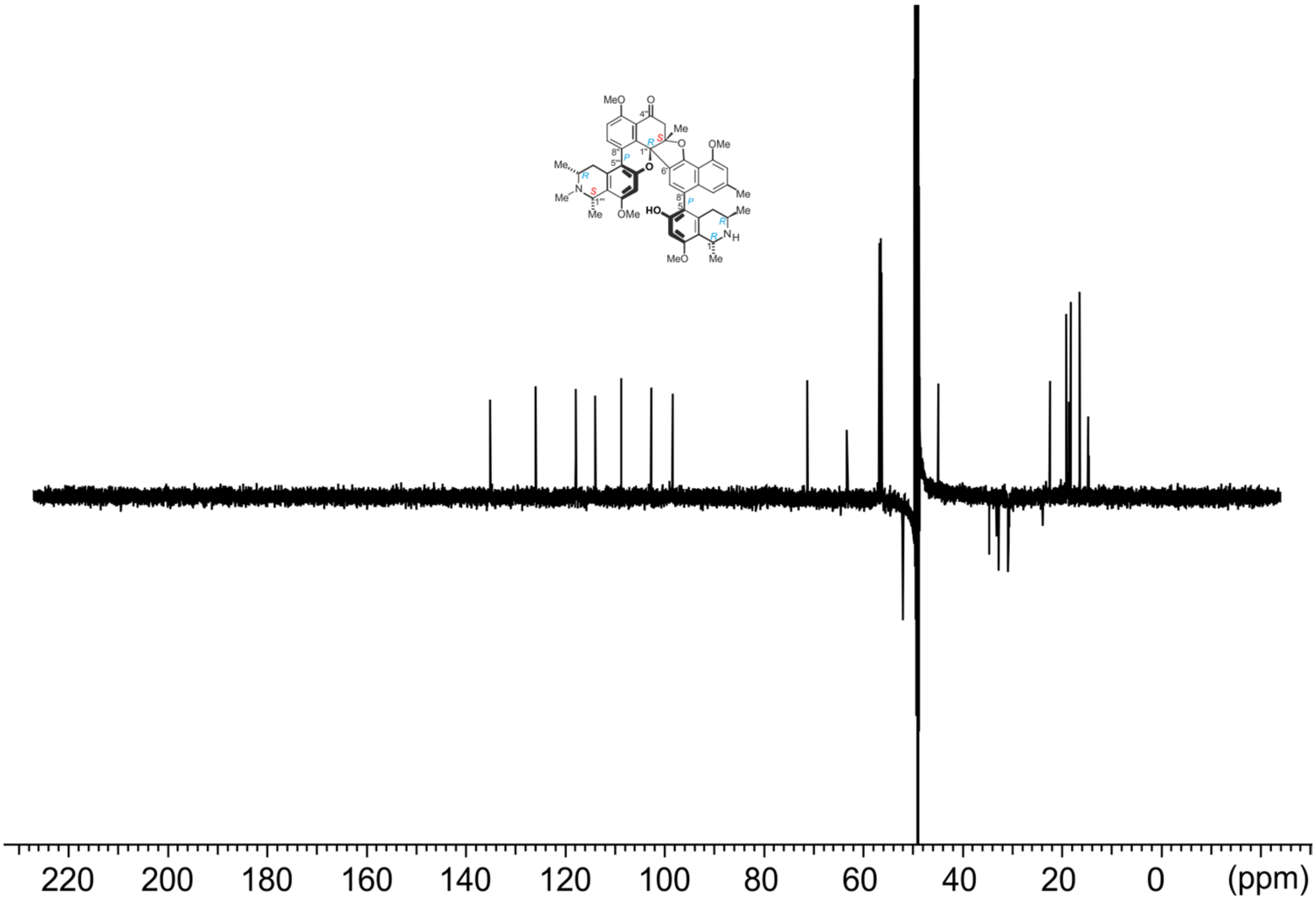

Figure S45. DEPT-135 NMR spectrum of cyclombandakamine A_4 **(4)** in methanol-d_4.

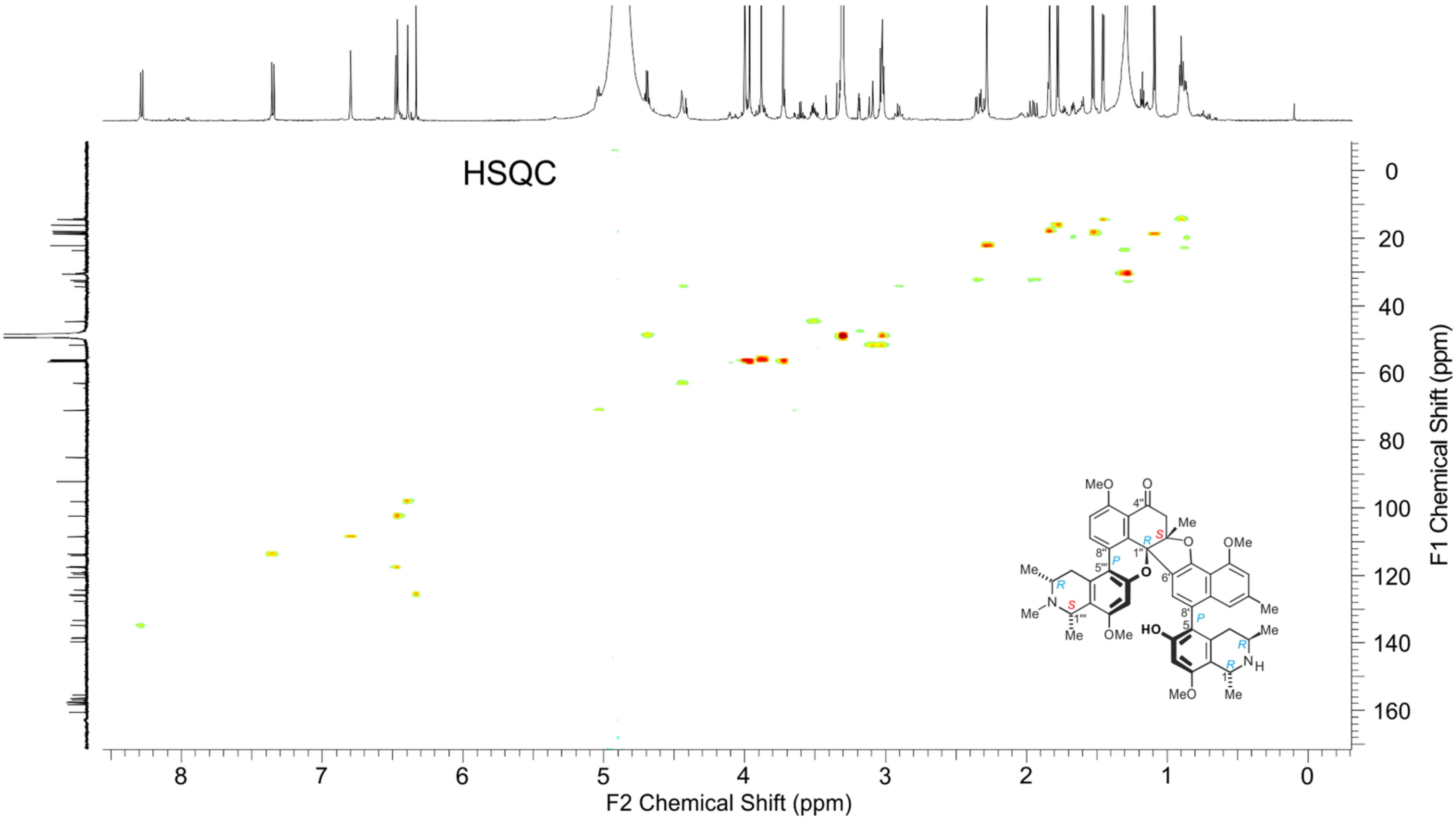

Figure S46. Overall HSQC spectrum of cyclombandakamine A_4 **(4)** in methanol-d_4.

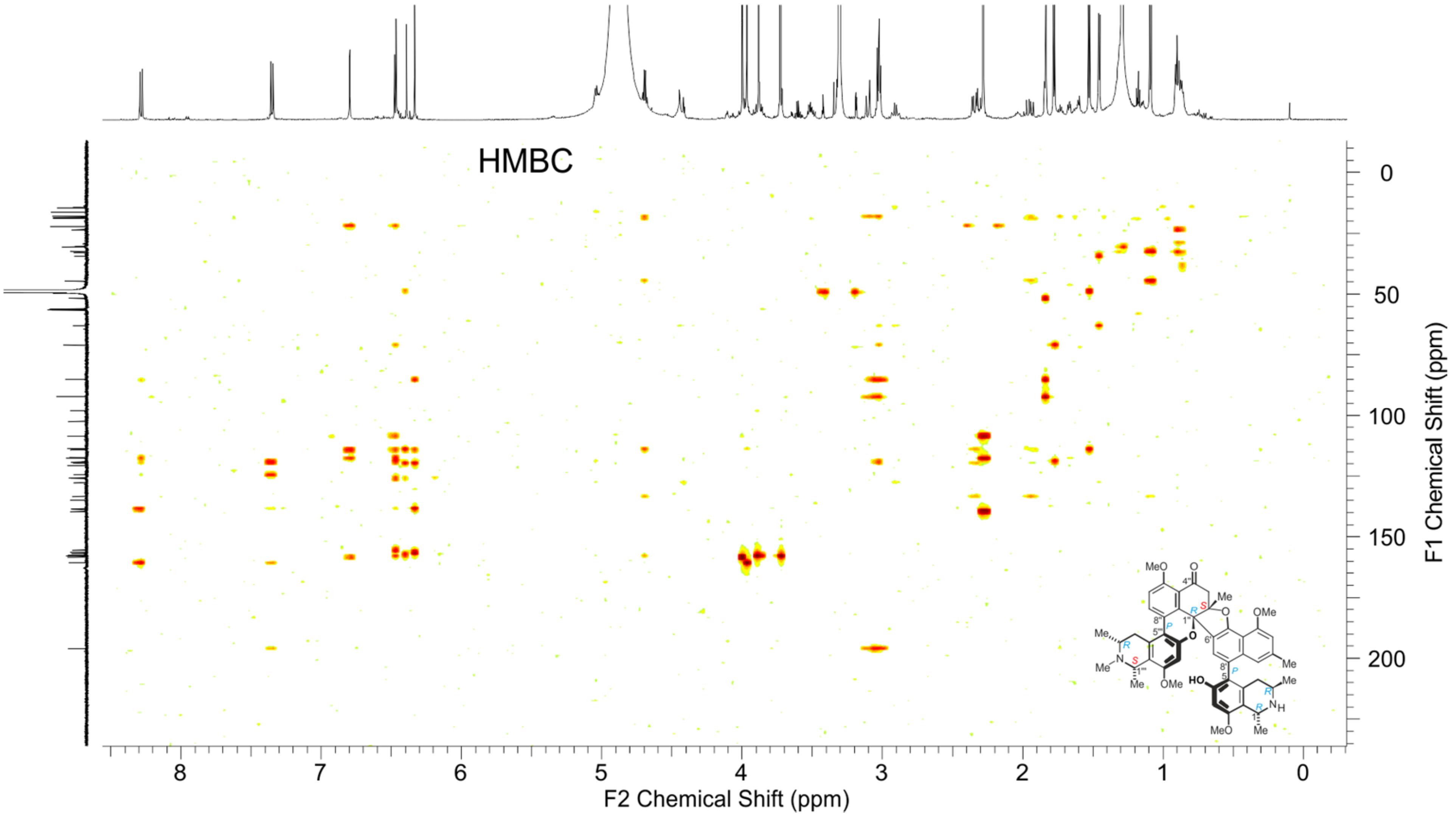

Figure S47. HMBC spectrum of cyclombandakamine A_4 **(4)** in methanol-d_4.

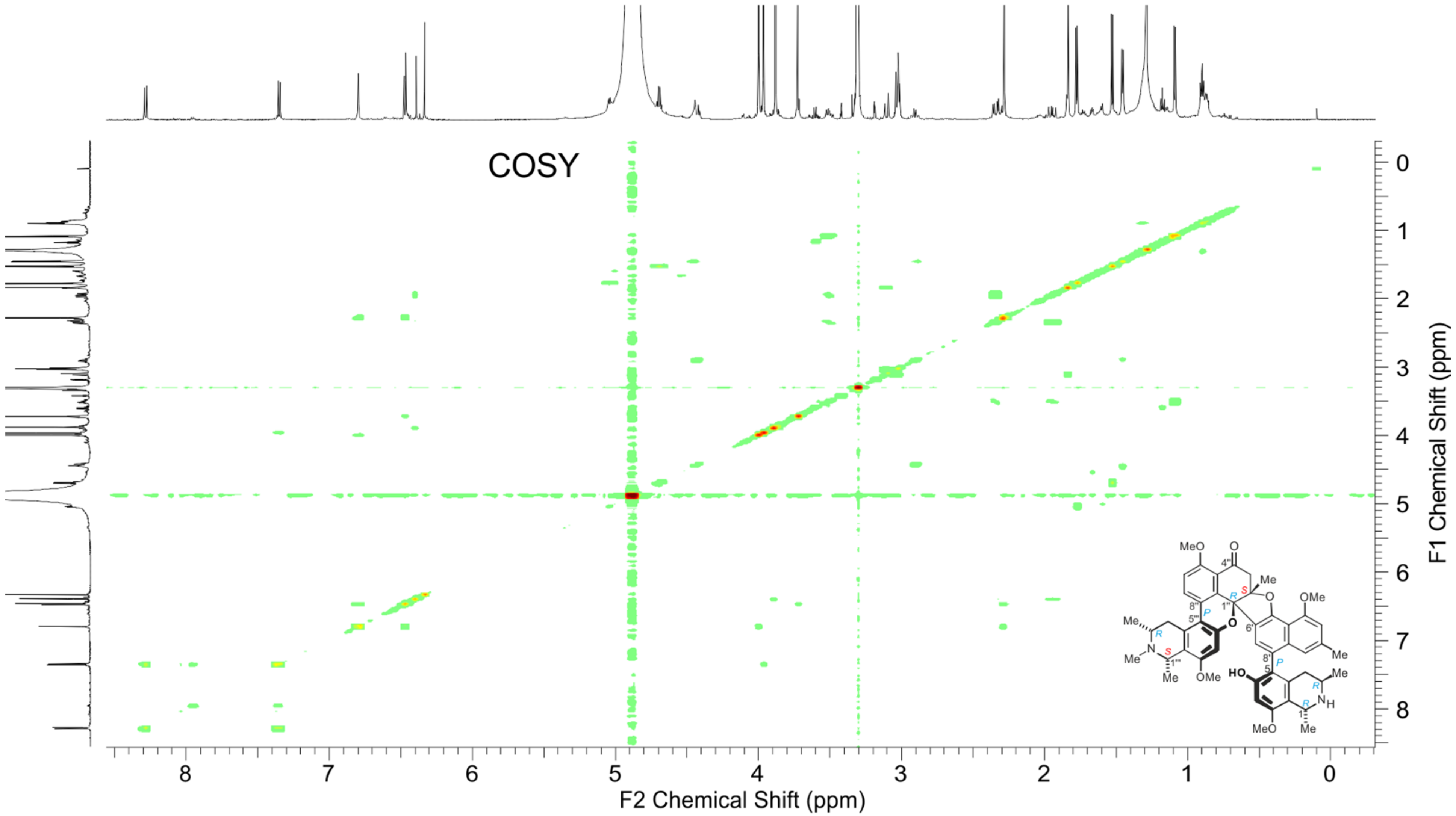

Figure S48a. Overall COSY spectrum of cyclombandakamine A_4 **(4)** in methanol-d_4.

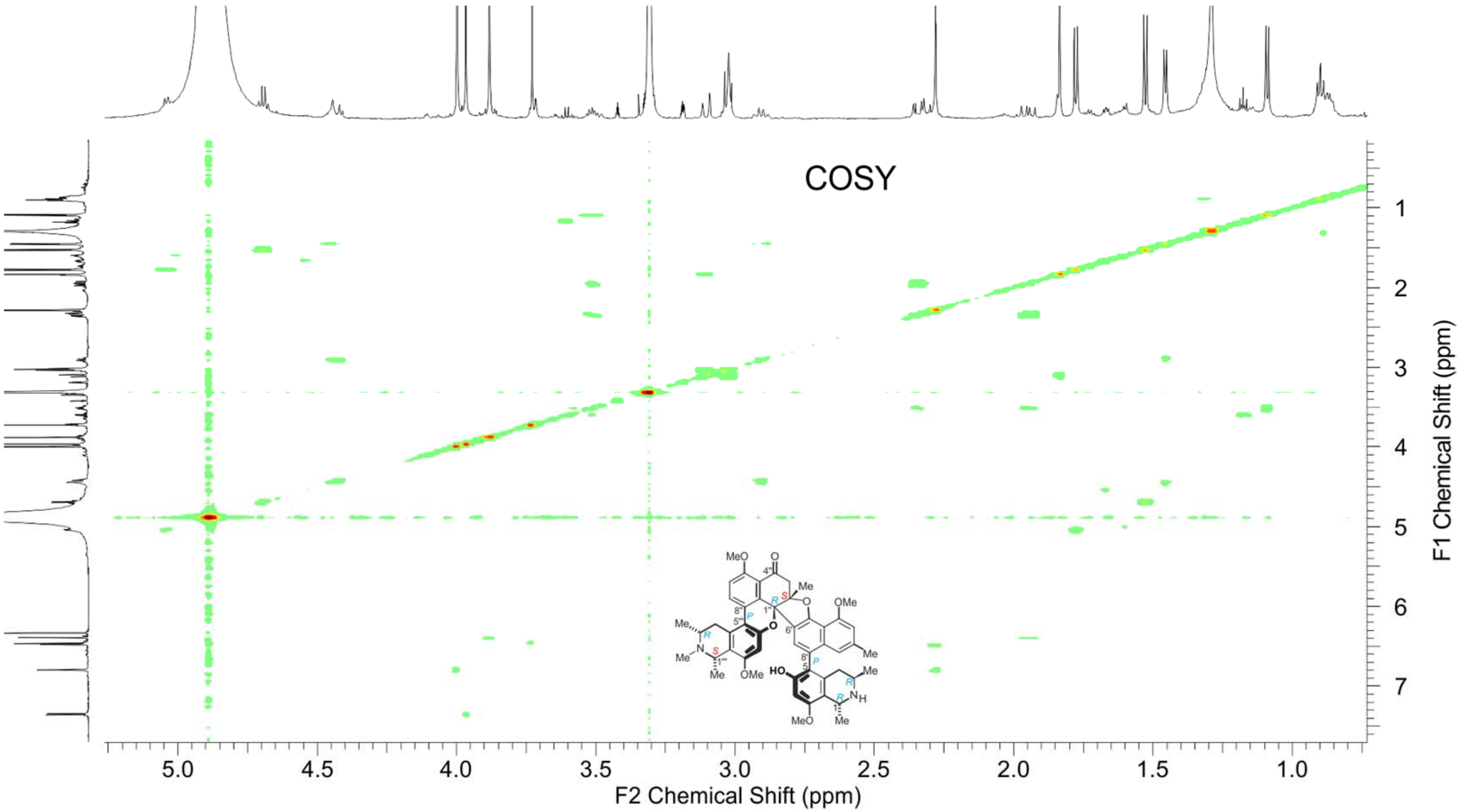

Figure S48b. Part of the COSY spectrum of cyclombandakamine A_4 **(4)** in methanol-d_4.

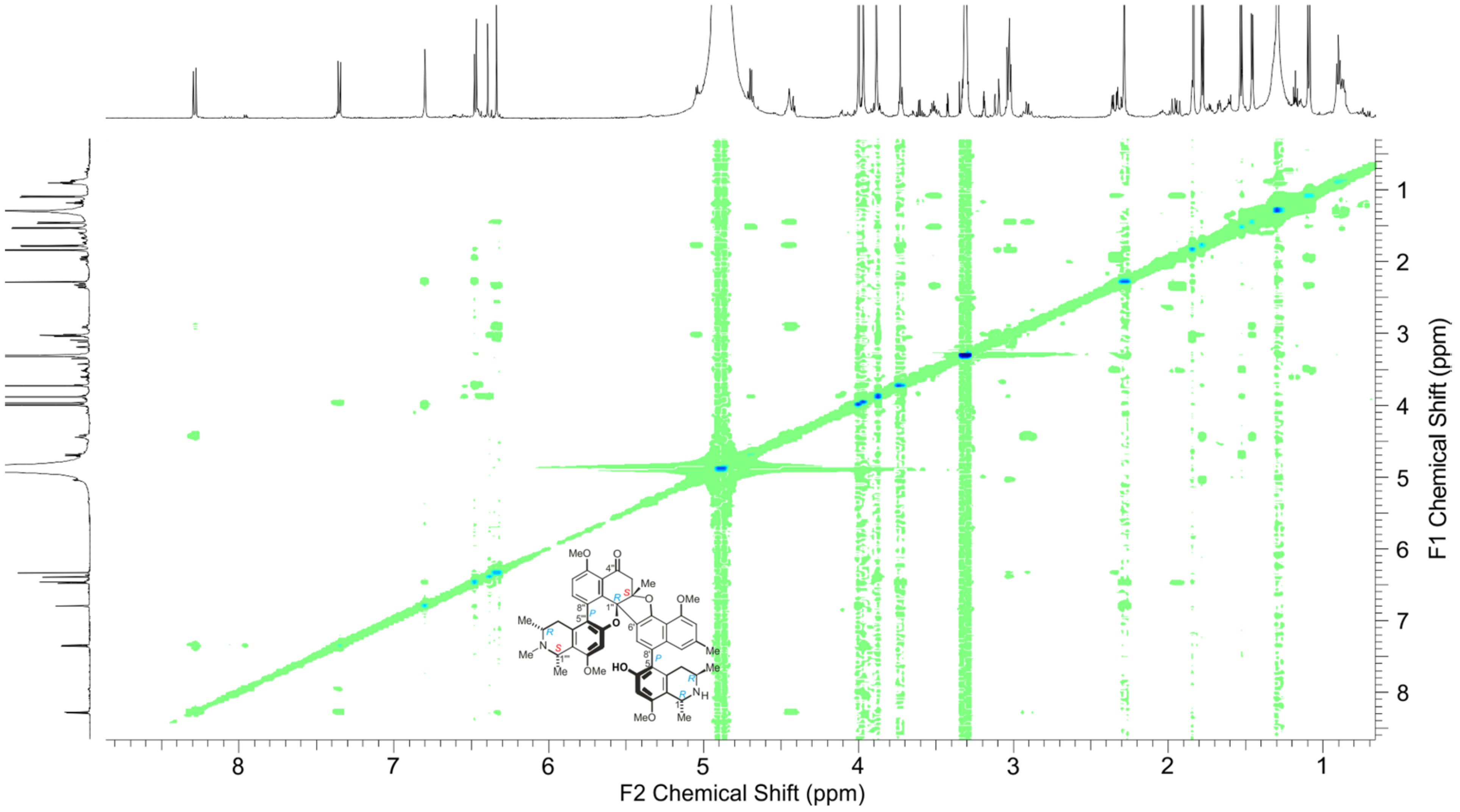

Figure S49a. ROESY spectrum of cyclombandakamine A_4 **(4)** in methanol-d_4.

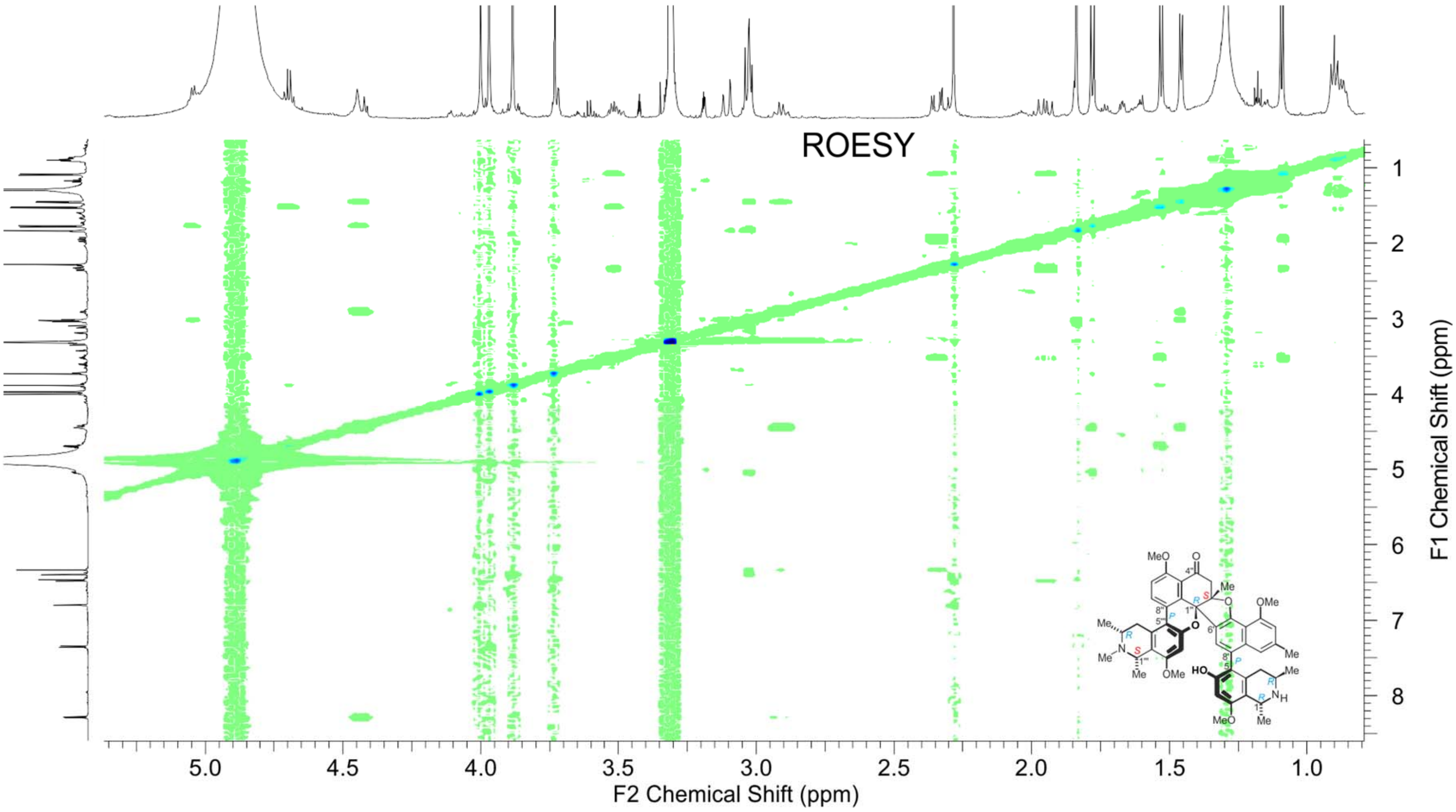

Figure S49b. Part of ROESY spectrum of cyclombandakamine A_4 **(4)** in methanol-d_4.

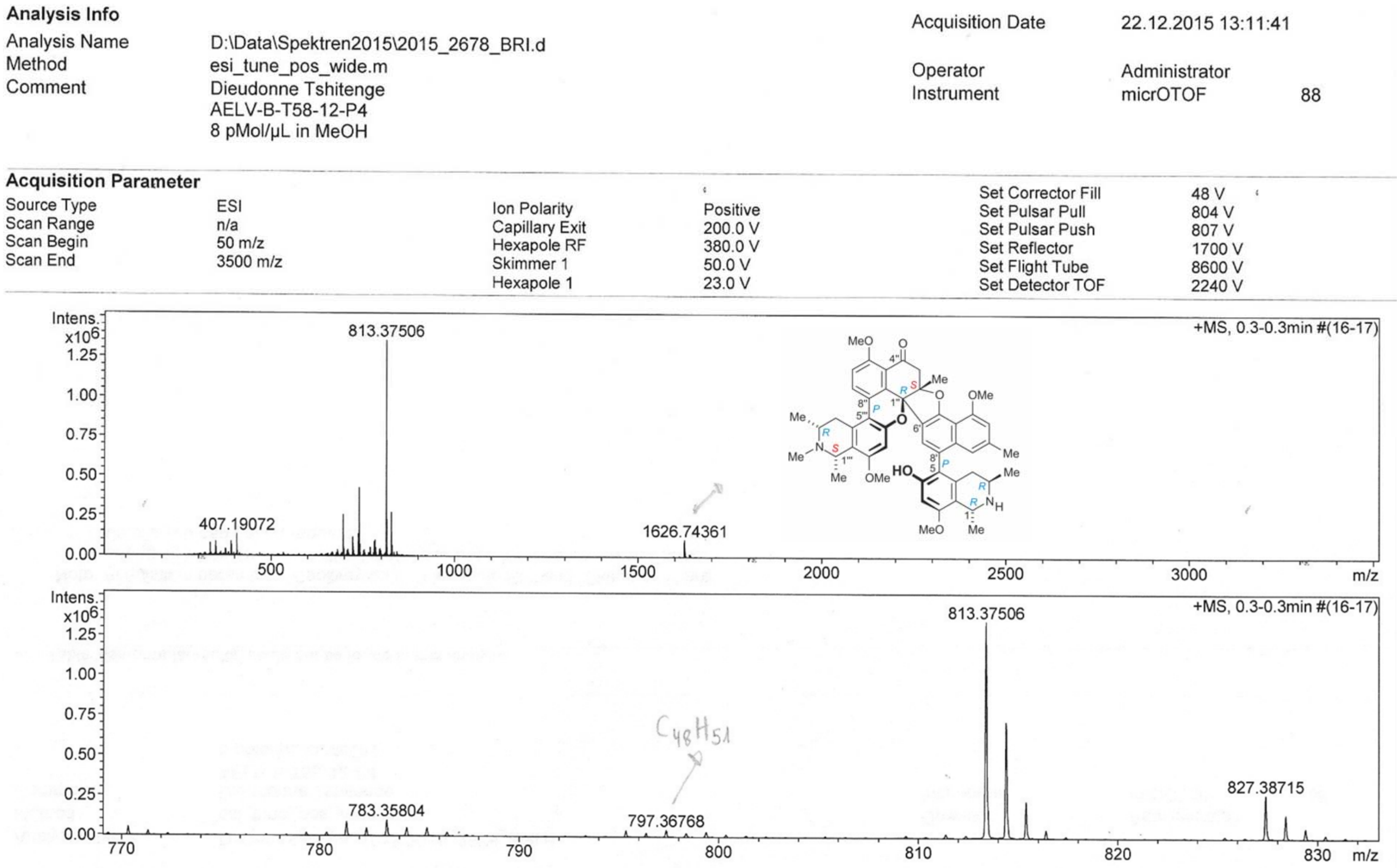

Figure S50. HRESIMS spectrum of cyclombandakamine A_4 **(4)** methanol.

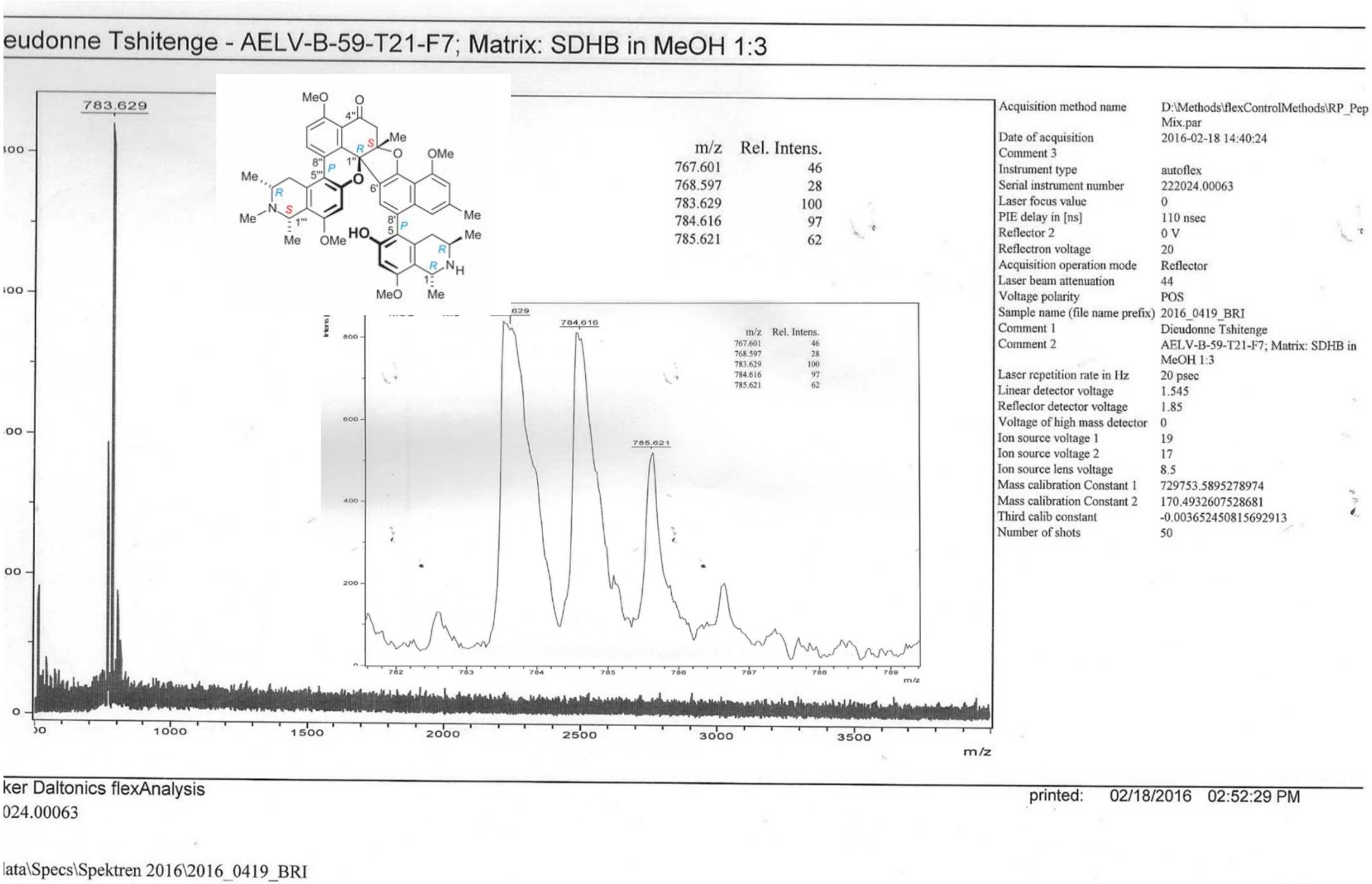

Figure S51. MALDI analysis: profile of cyclombandakamine A_4 (**4**).

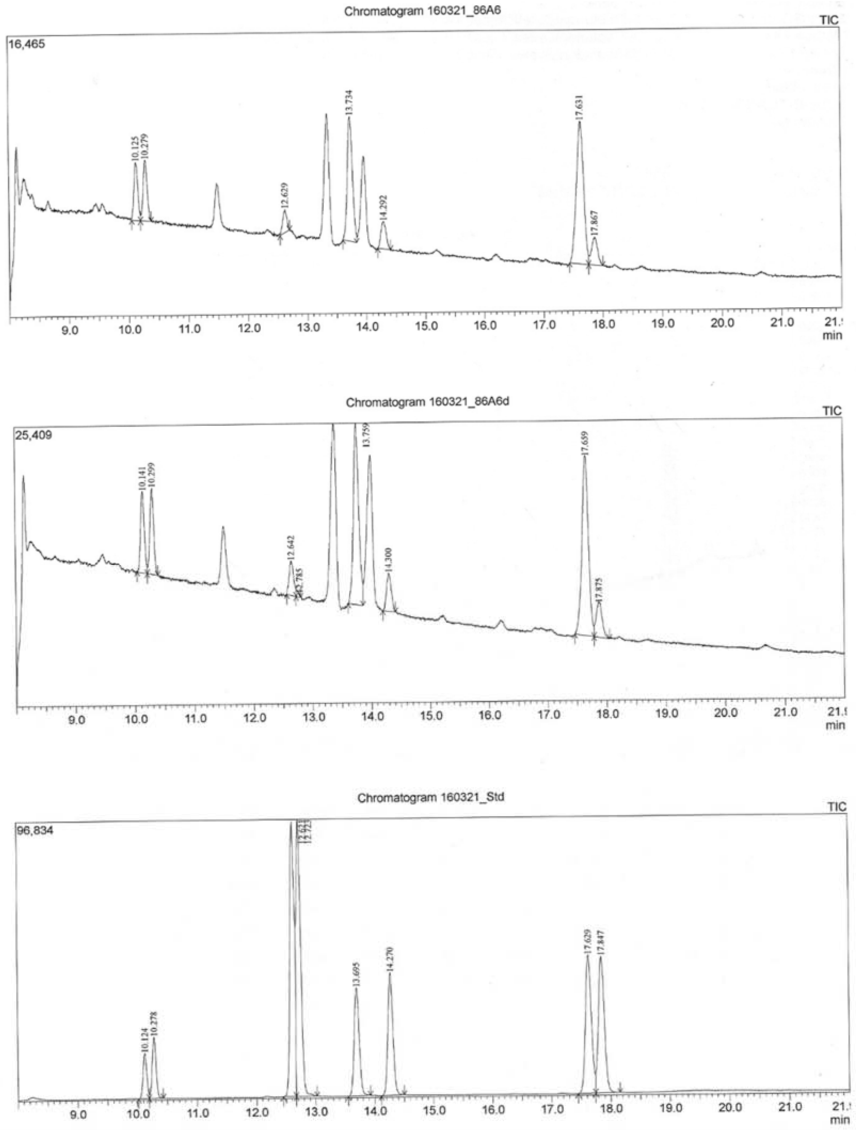

Modified by : Admin
Modified : 21.03.2016 10:51:48

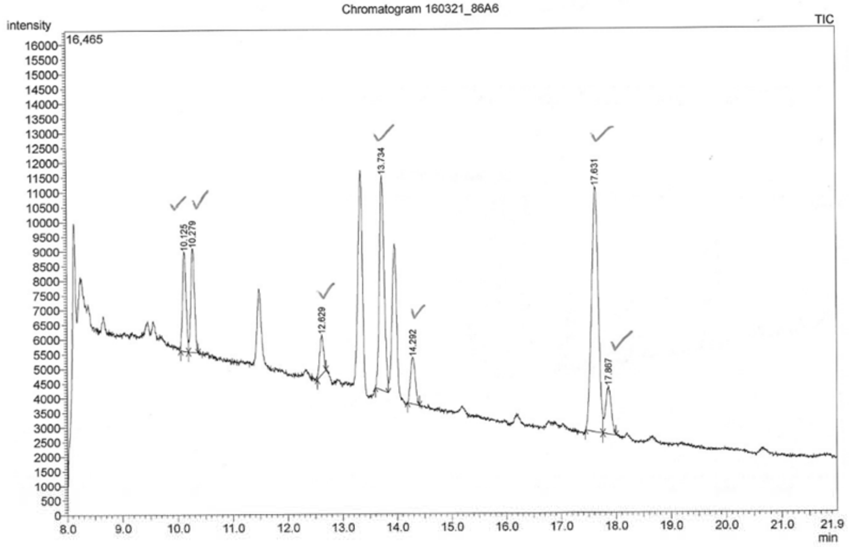

Peak#	Name	R.Time	I.Time	F.Time	Area	Area%	Height	Height%	
1		10.125	10.055	10.200	15143	8.67	3401	12.50	
2		10.279	10.200	10.375	16428	9.41	3547	13.04	
3		12.629	12.545	12.700	6471	3.71	1359	4.99	
4		13.734	13.605	13.840	44531	25.51	7311	26.87	R:S 4,6:1
5		14.292	14.195	14.410	9661	5.53	1601	5.88	
6		17.631	17.445	17.765	70765	40.54	8350	30.69	R:S 6:1
7		17.867	17.765	18.000	11571	6.63	1642	6.03	
					174570	100.00	27211	100.00	

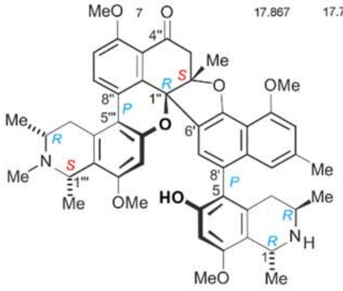

Figure S52. Oxidative degradation results of cyclombandakamine A_4 (**4**) (very diluted sample).

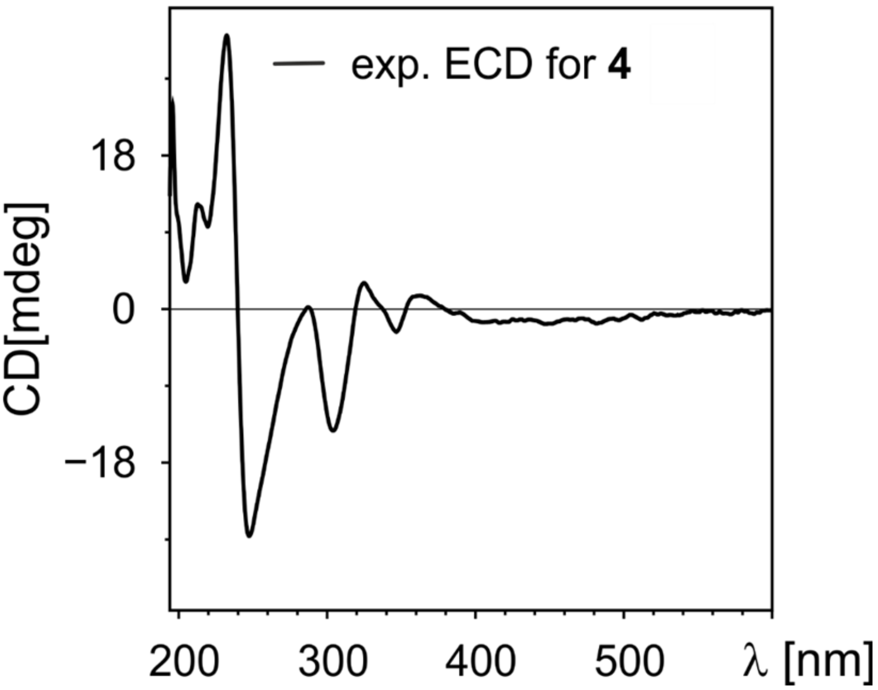

Figure S53. ECD spectrum of cyclombandakamine A_4 (**4**) in methanol.

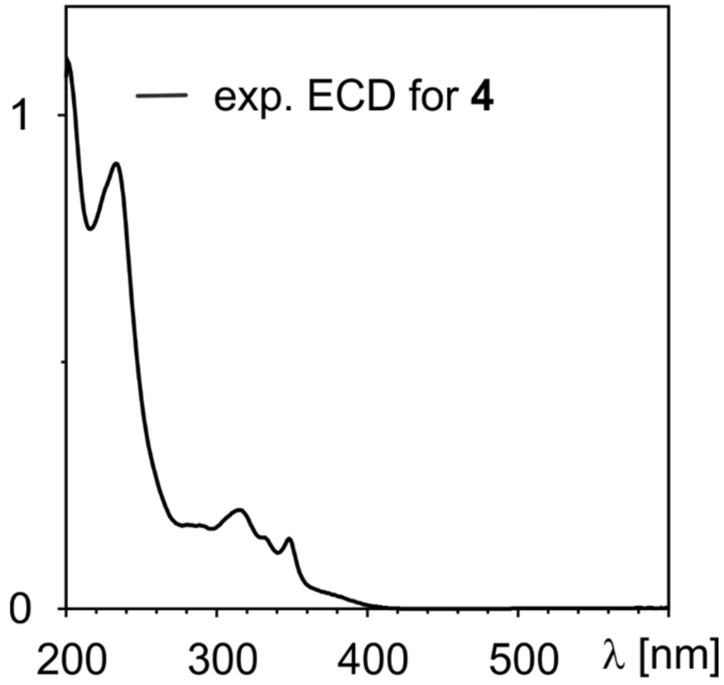

Figure S54. Offline UV spectrum of cyclombandakamine A_4 (**4**) in methanol.

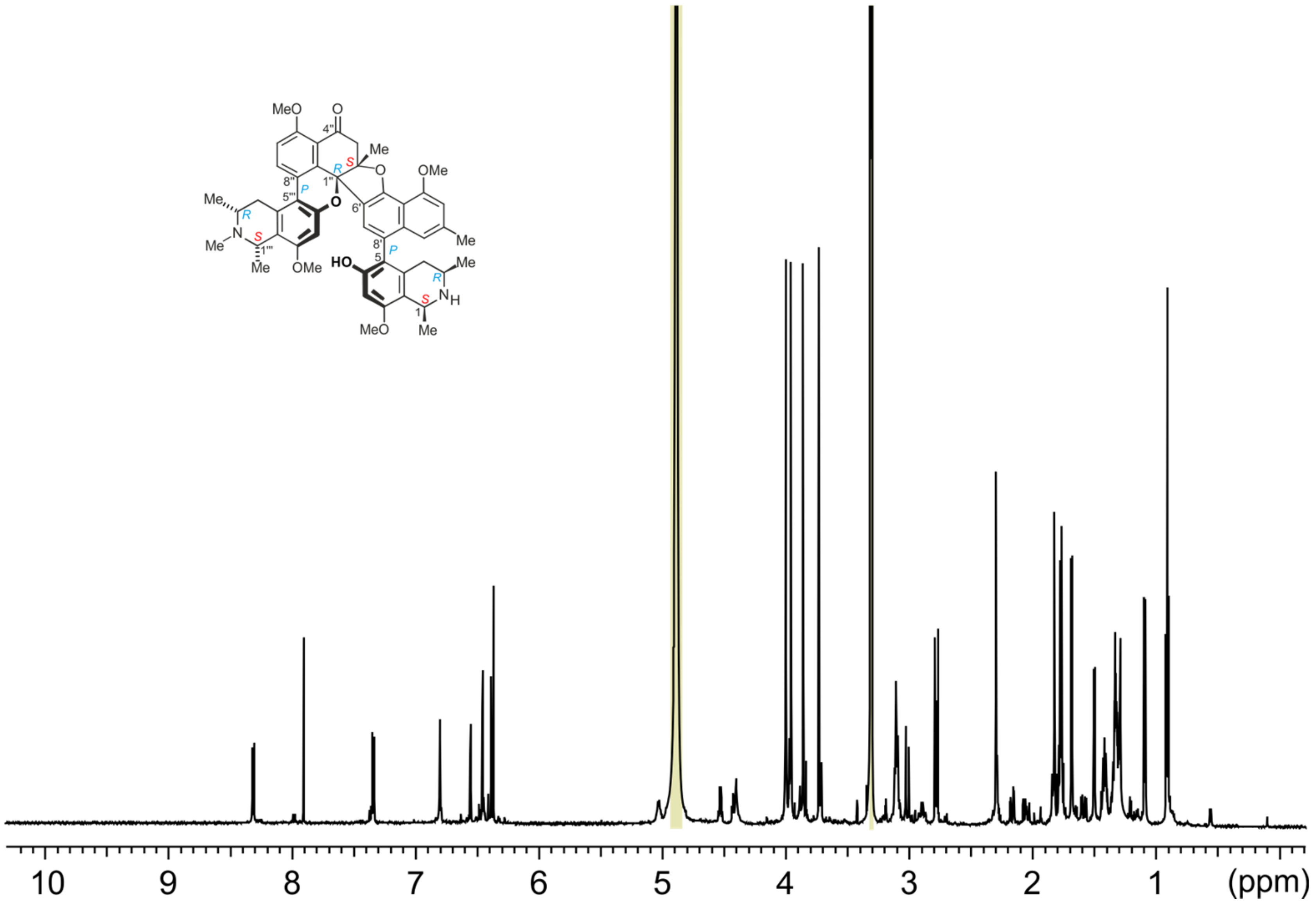

Figure S55a. Overall ^{1}H NMR spectrum of cyclombandakamine A_5 (**5**) in methanol-d_4

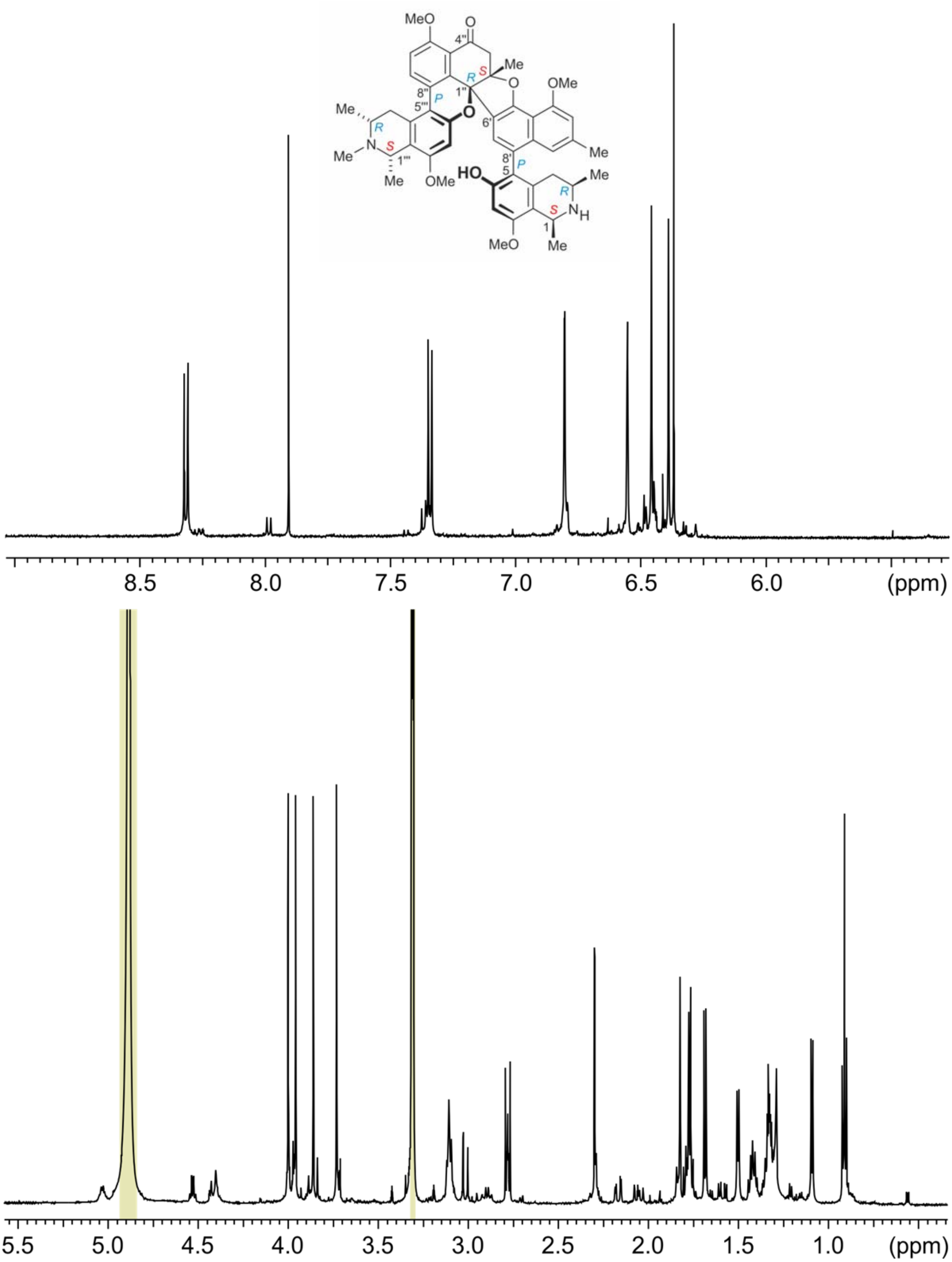

Figure S55b,c. Parts of the ^{1}H NMR spectrum of cyclombandakamine A_5 (**5**) in methanol-d_4.

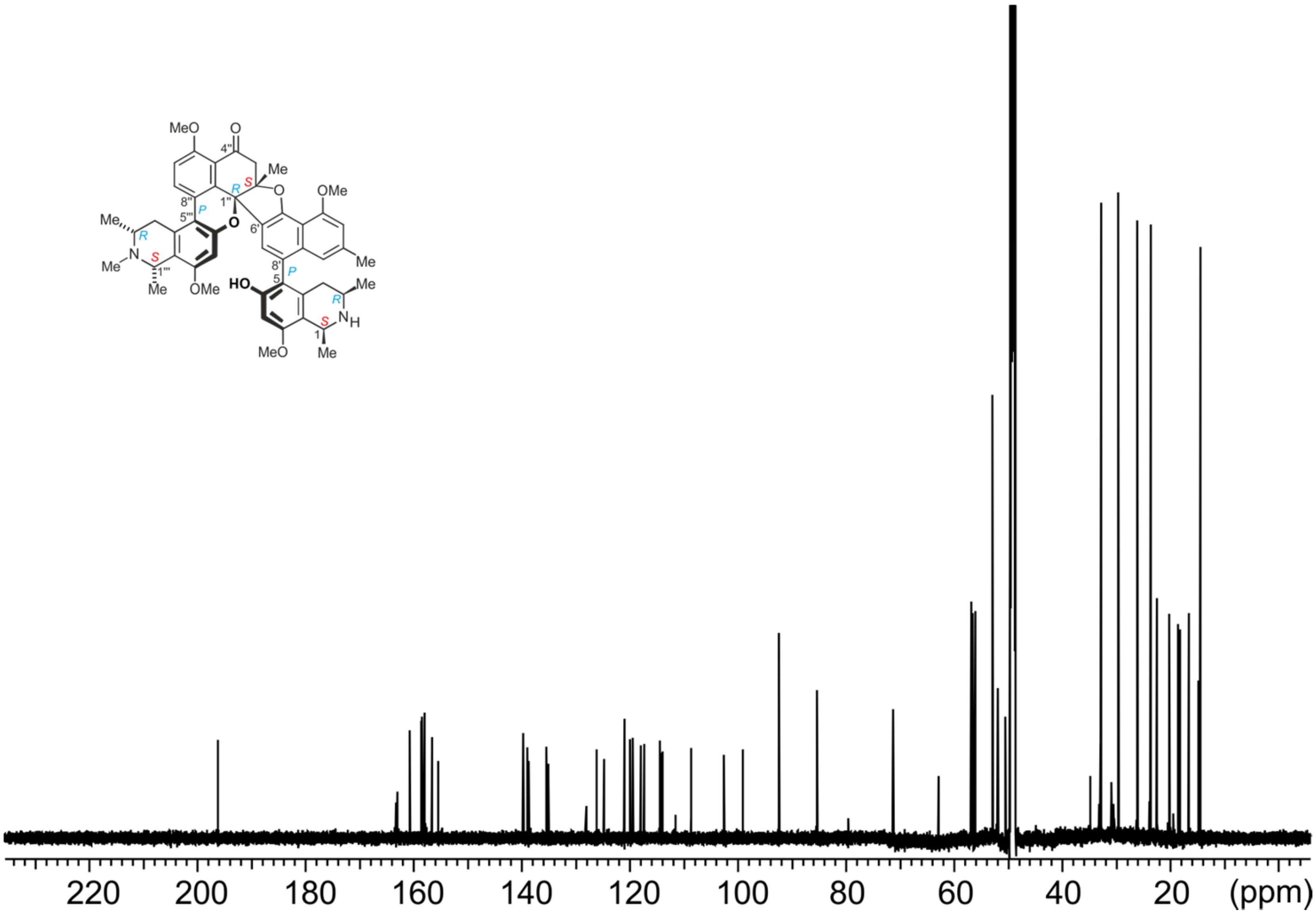

Figure S56. ^{13}C NMR spectrum of cyclombandakamine A$_5$ **(5)** in methanol-d_4.

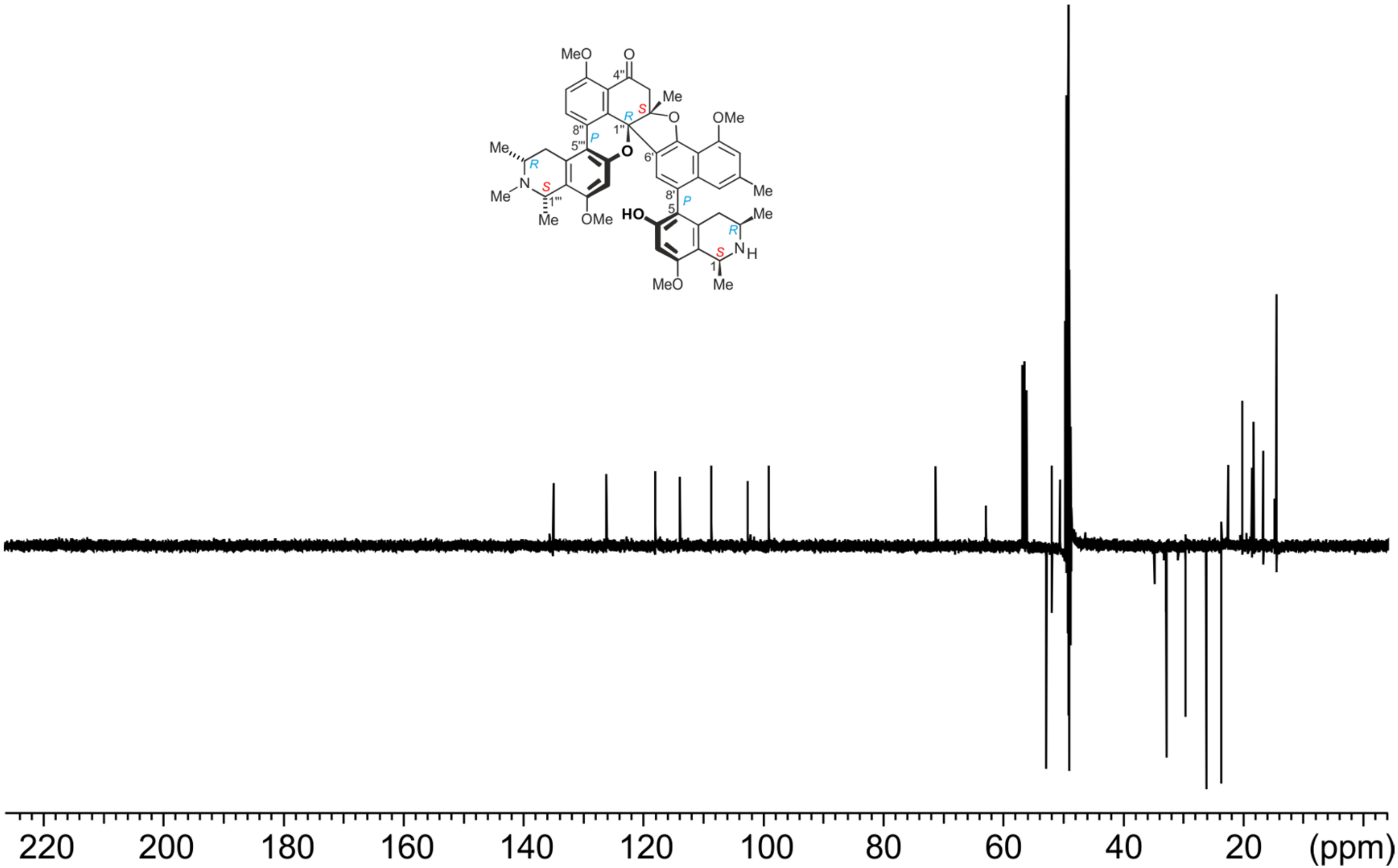

Figure S57. DEPT-135 NMR spectrum of cyclombandakamine A_5 **(5)** in methanol-d_4.

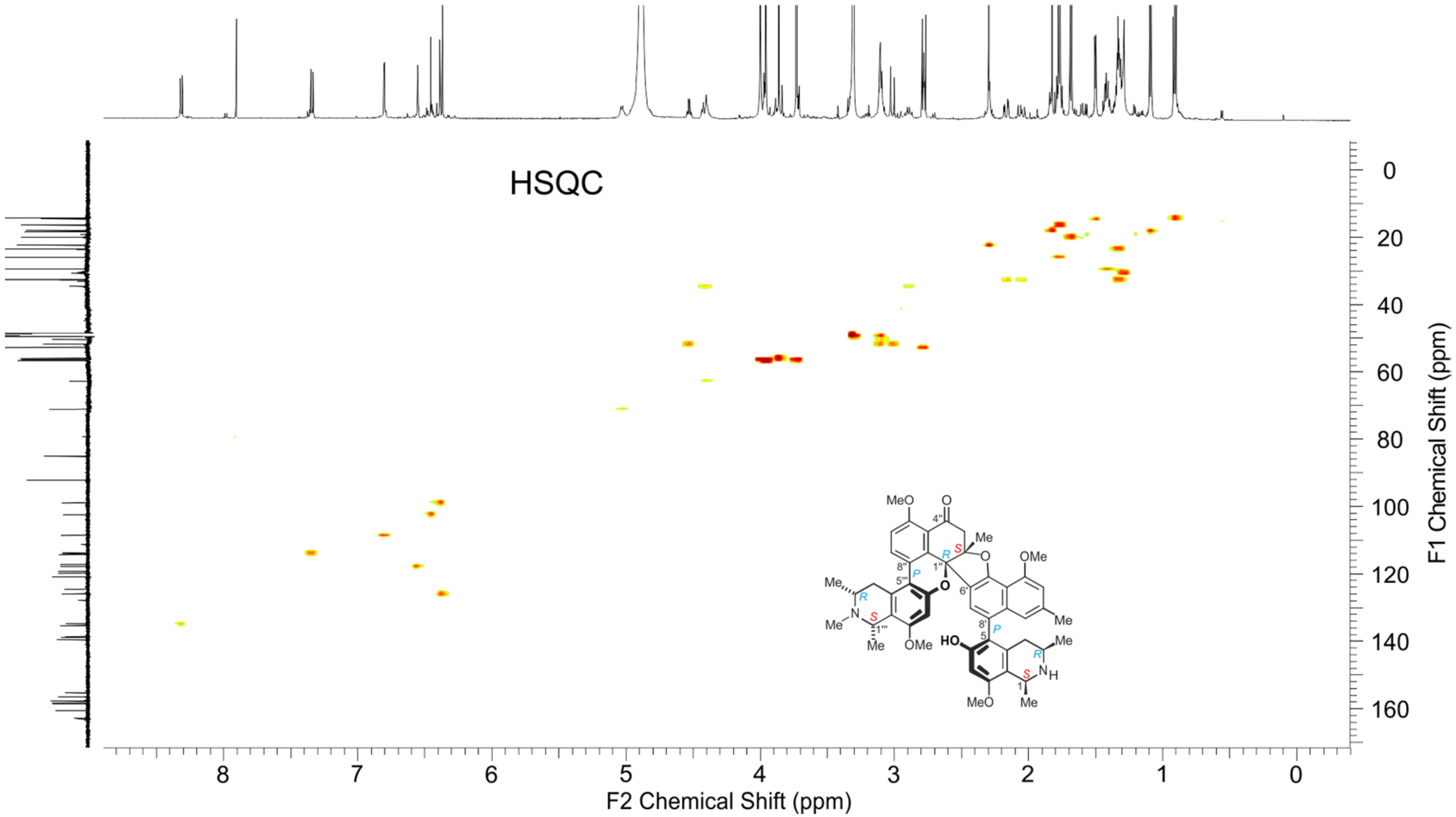

Figure S58a. Overall HSQC spectrum of cyclombandakamine A_5 **(5)** in methanol-d_4.

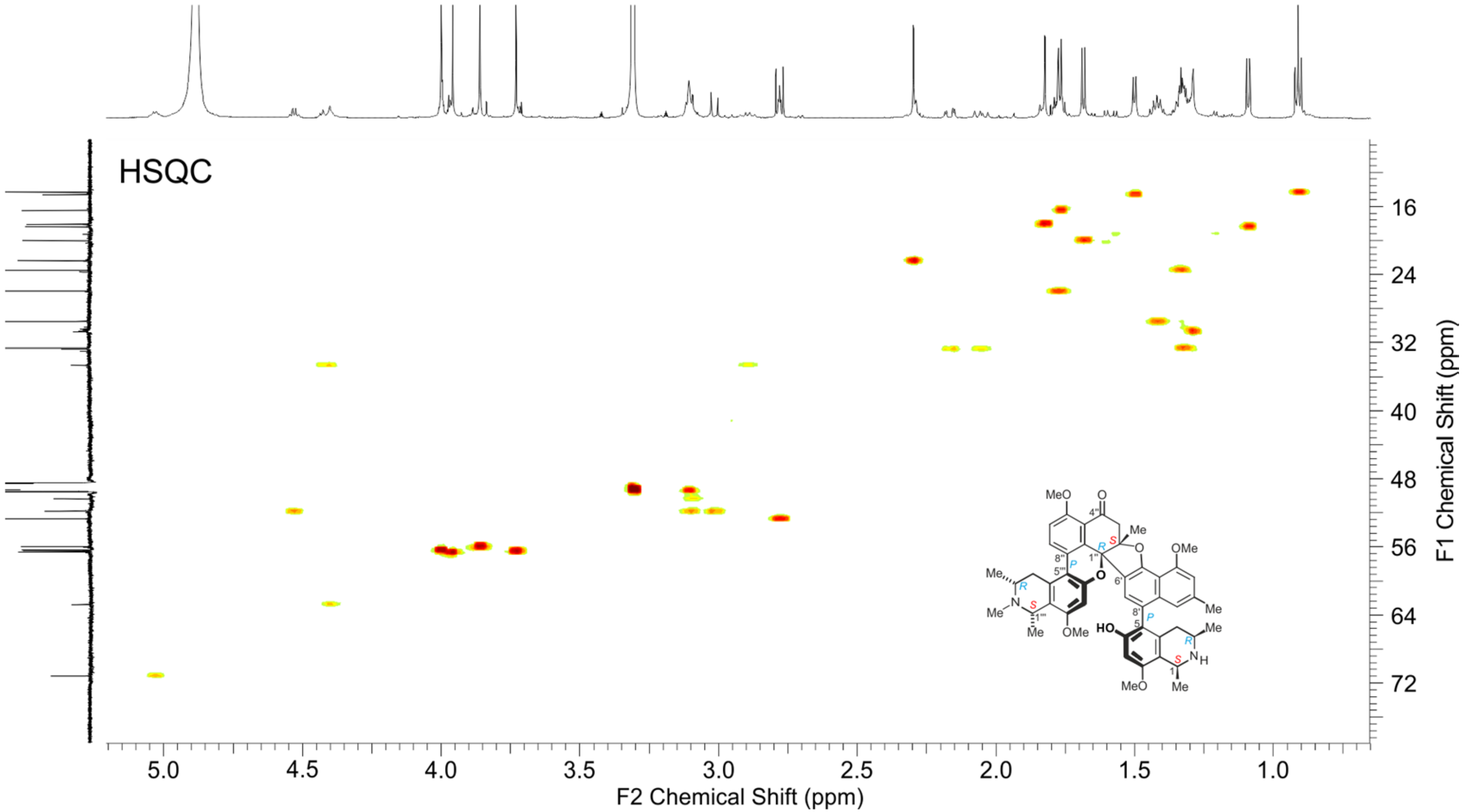

Figure S58b. Part of the HSQC spectrum of cyclombandakamine A_5 **(5)** in methanol-d_4.

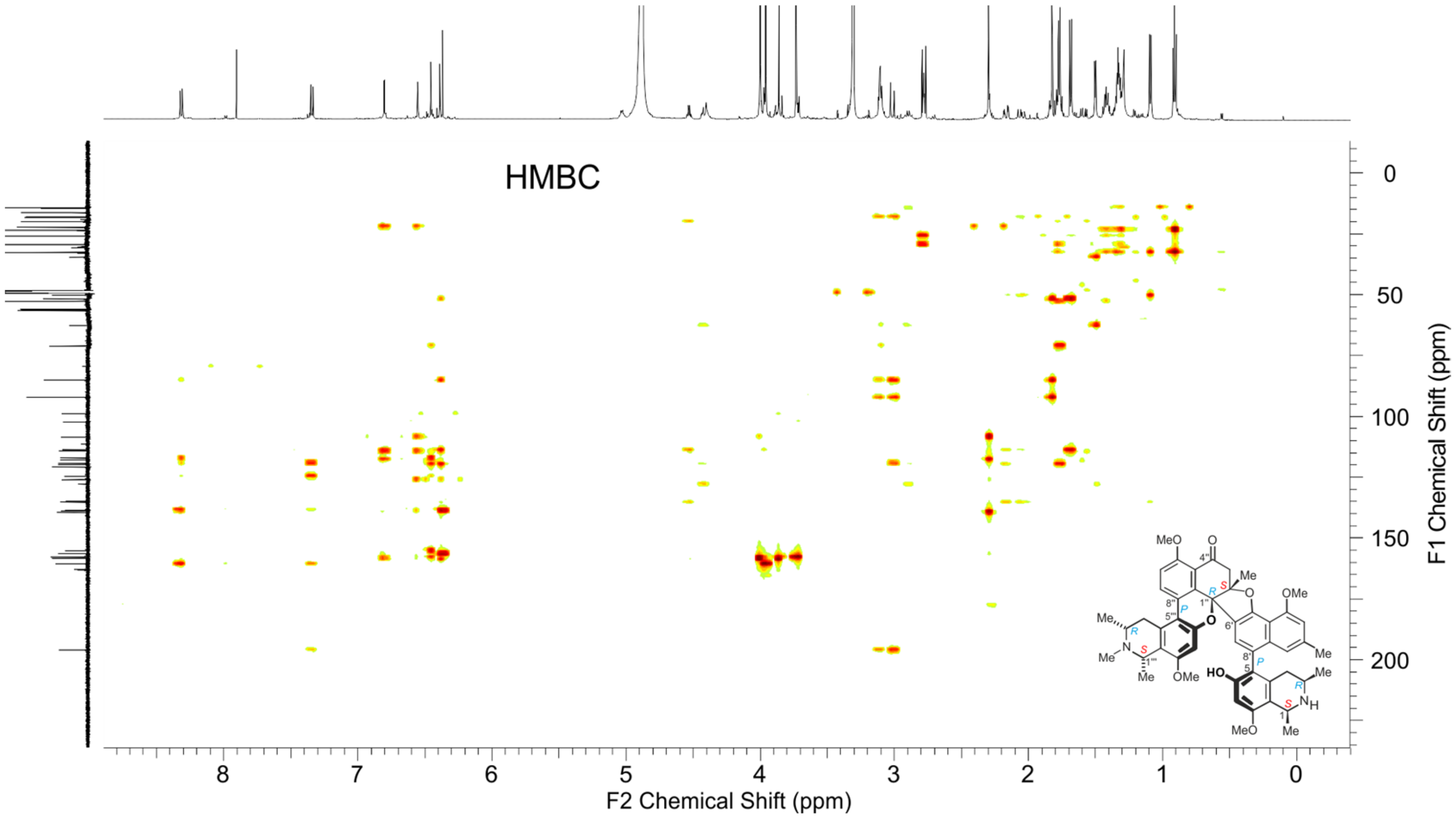

Figure S59a. HMBC spectrum of cyclombandakamine A_5 **(5)** in methanol-d_4.

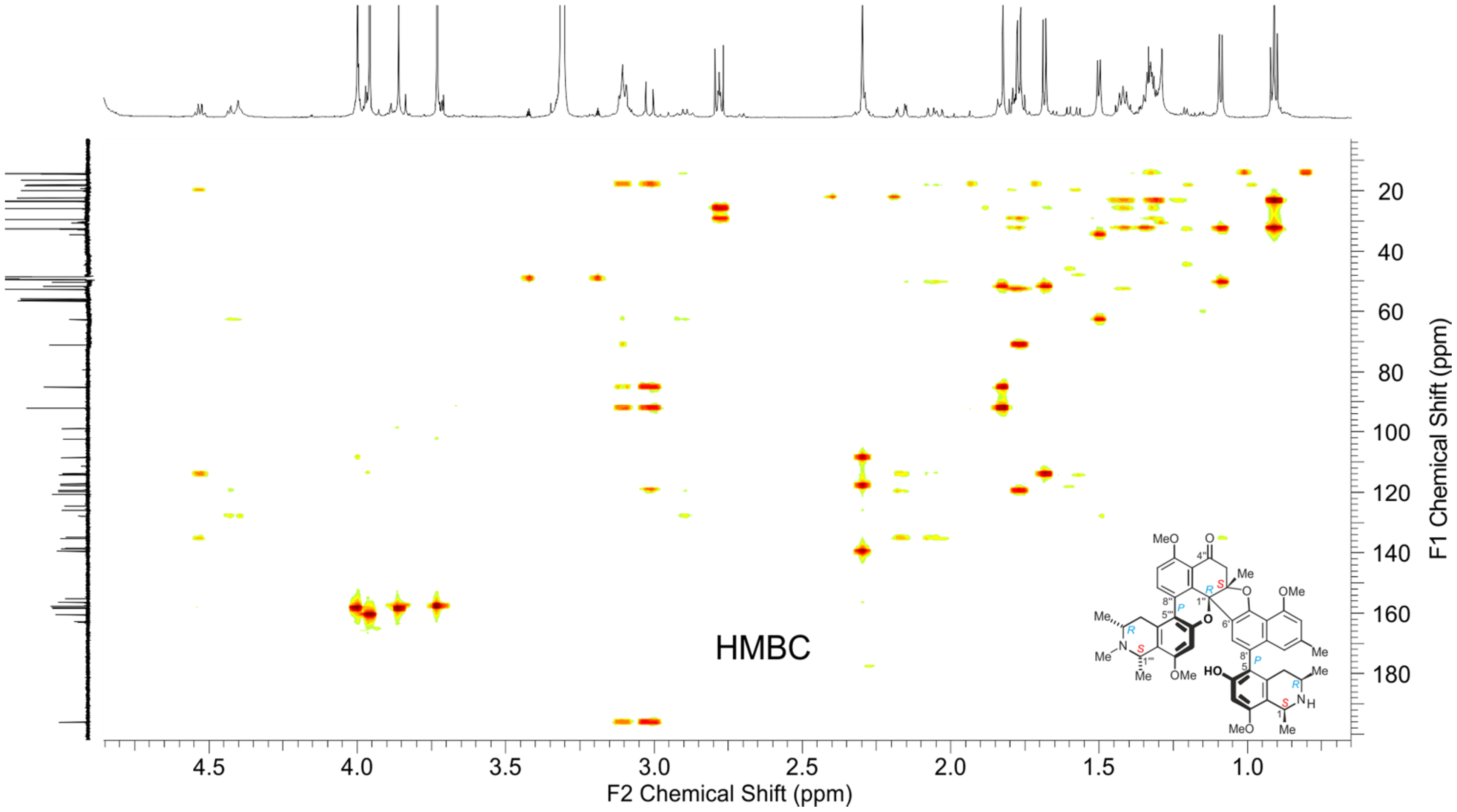

Figure S59b. HMBC spectrum of cyclombandakamine A_5 **(5)** in methanol-d_4.

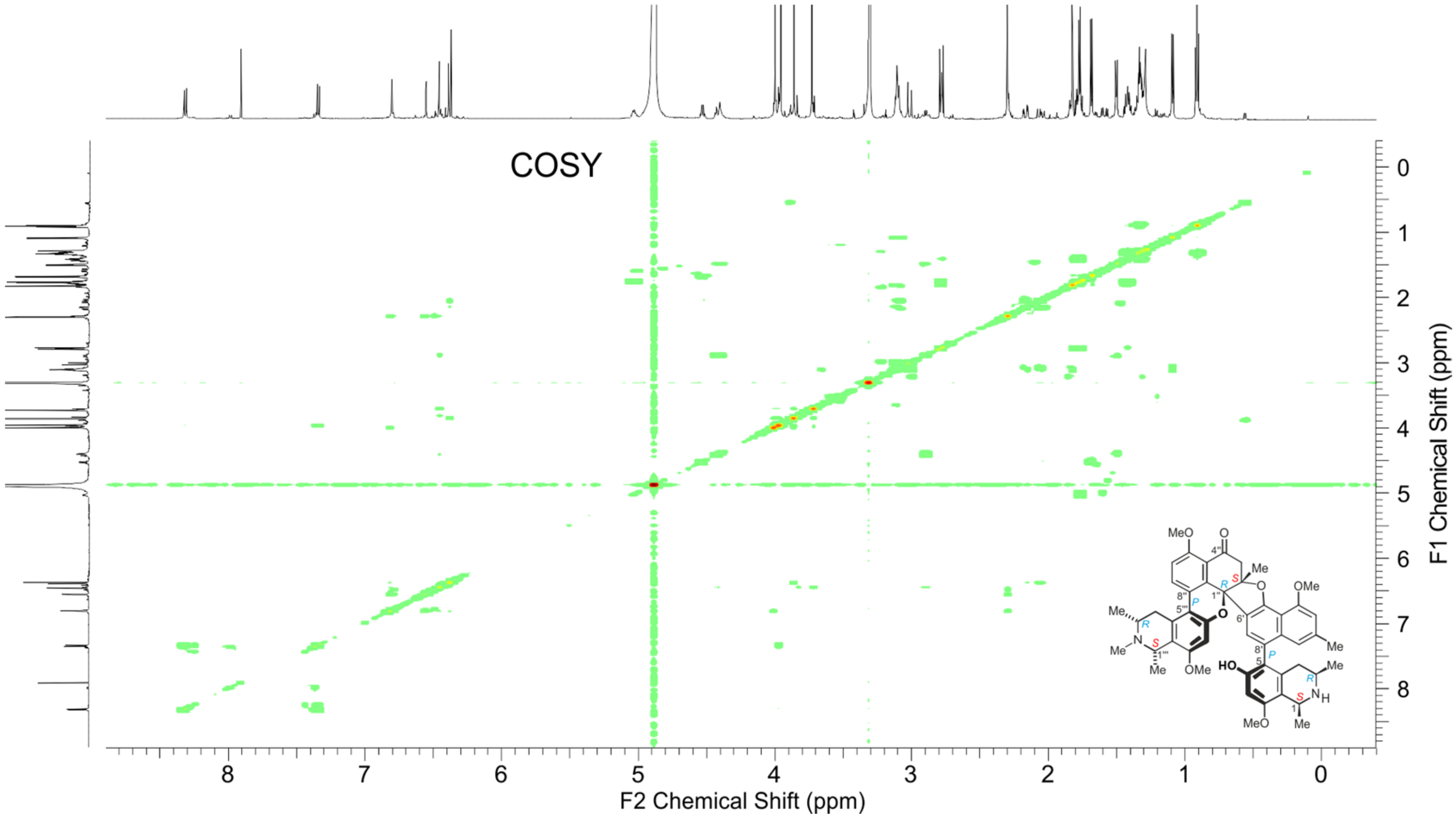

Figure S60. Overall COSY spectrum of cyclombandakamine A$_5$ **(5)** in methanol-d_4.

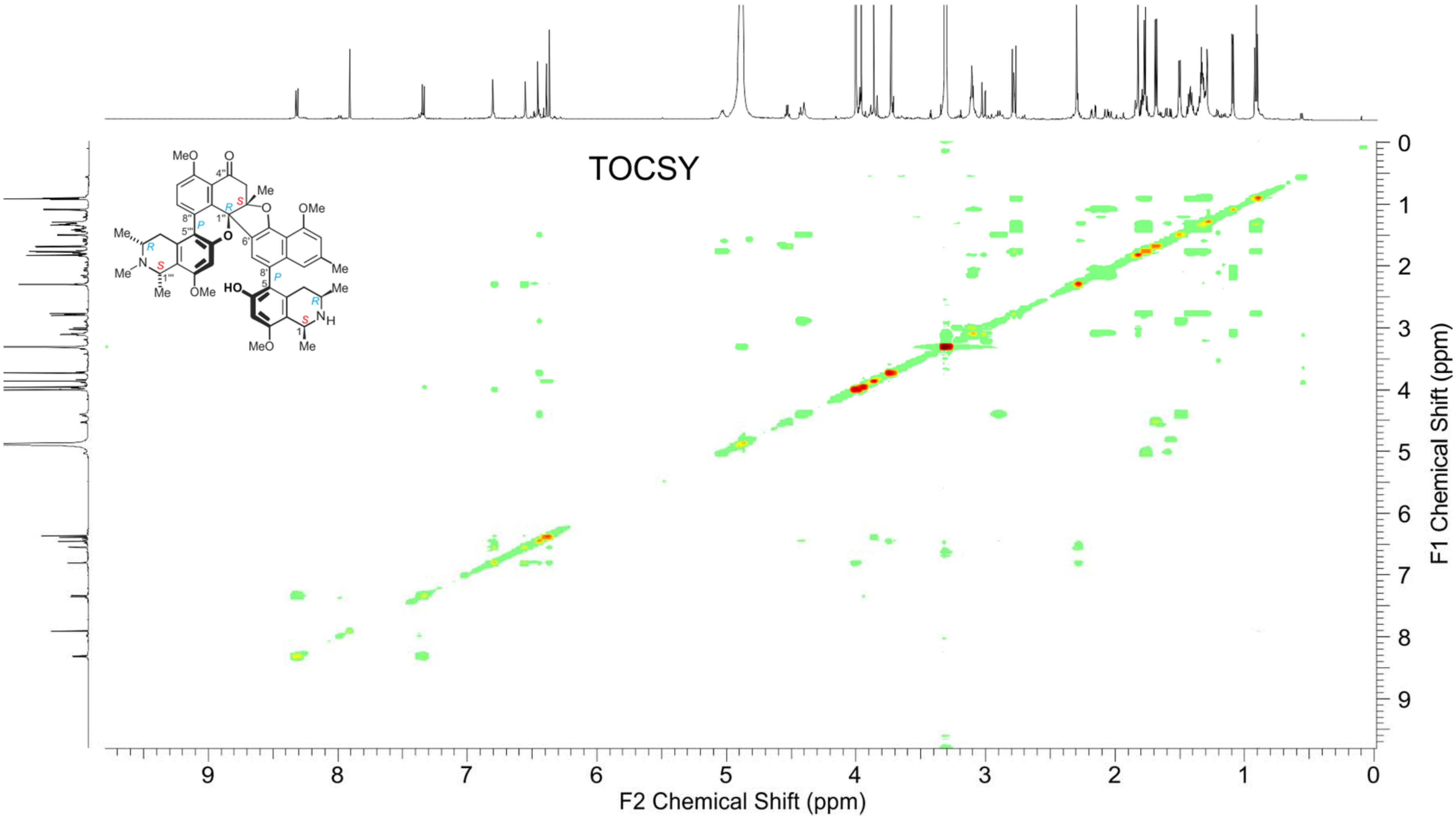

Figure S61. TOCSY spectrum of cyclombandakamine A_5 **(5)** in methanol-d_4.

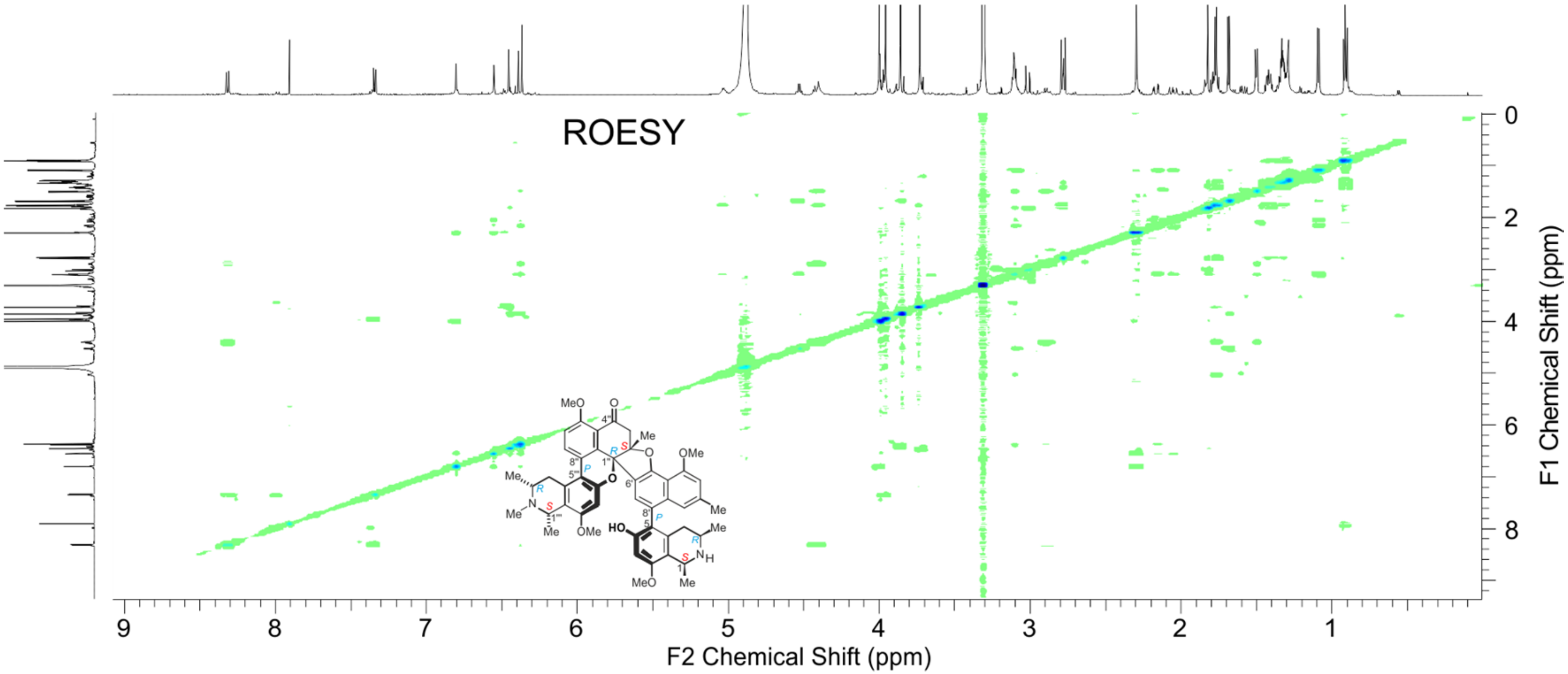

Figure S62a. ROESY spectrum of cyclombandakamine A_5 **(5)** in methanol-d_4.

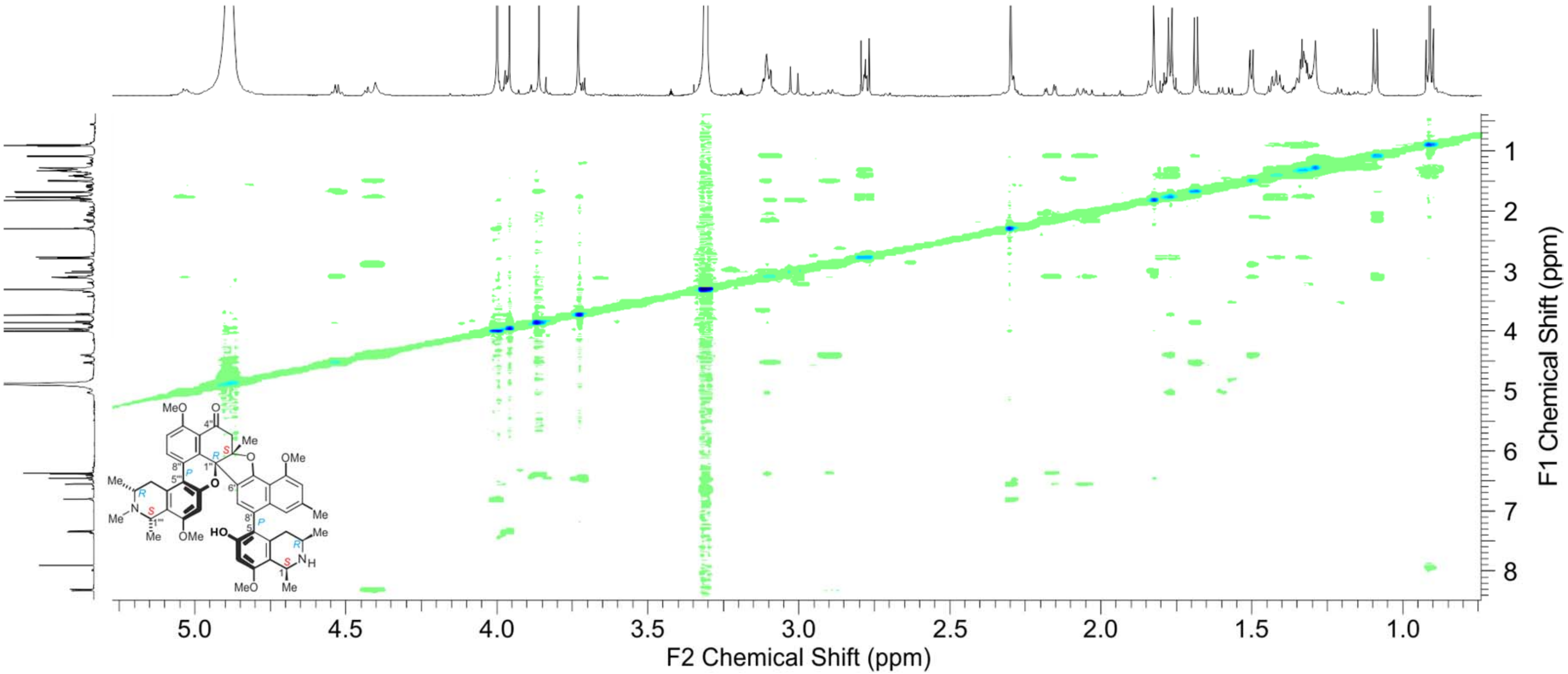

Figure S62b. Aliphatic part of the ROESY spectrum of cyclombandakamine A_5 **(5)** in methanol-d_4.

Bruker Daltonics DataAnalysis 3.3

Mass Spectrum Molecular Formula Report

Analysis Info

Analysis Name	D:\Data\Spektren2016\2016_0416_BRI.d
Method	esi_tune_pos_wide.m
Comment	Dieudonne Tshitenge<br>AELV-B-T58-13-PZ3<br>8 pMol/µL in MeOH

Acquisition Date	18.02.2016 13:50:09	
Operator	Administrator	
Instrument	micrOTOF	88

Acquisition Parameter

Source Type	ESI	Ion Polarity	Positive	Set Corrector Fill	48 V
Scan Range	n/a	Capillary Exit	280.0 V	Set Pulsar Pull	804 V
Scan Begin	50 m/z	Hexapole RF	380.0 V	Set Pulsar Push	807 V
Scan End	3500 m/z	Skimmer 1	100.0 V	Set Reflector	1700 V
		Hexapole 1	23.0 V	Set Flight Tube	8600 V
				Set Detector TOF	2240 V

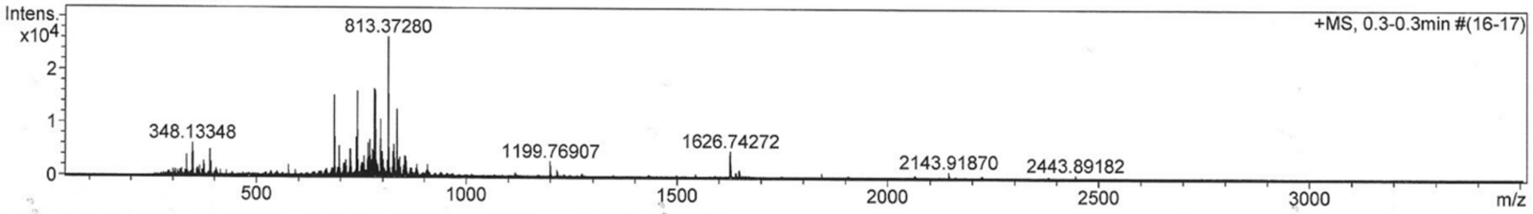

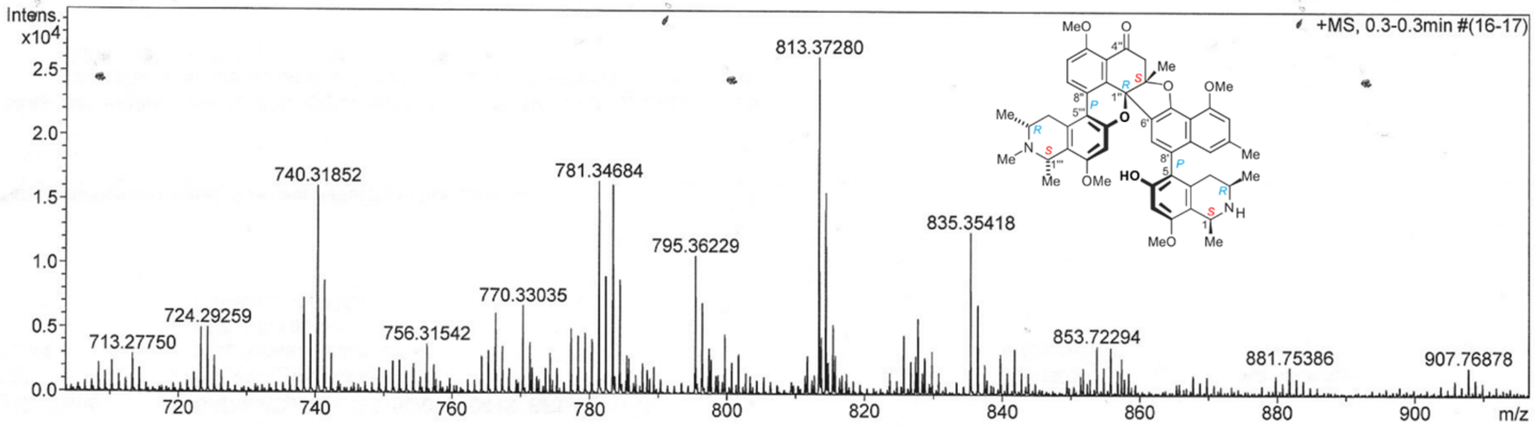

Figure S63. HRESIMS spectrum of cyclombandakamine A_5 **(5)** methanol.

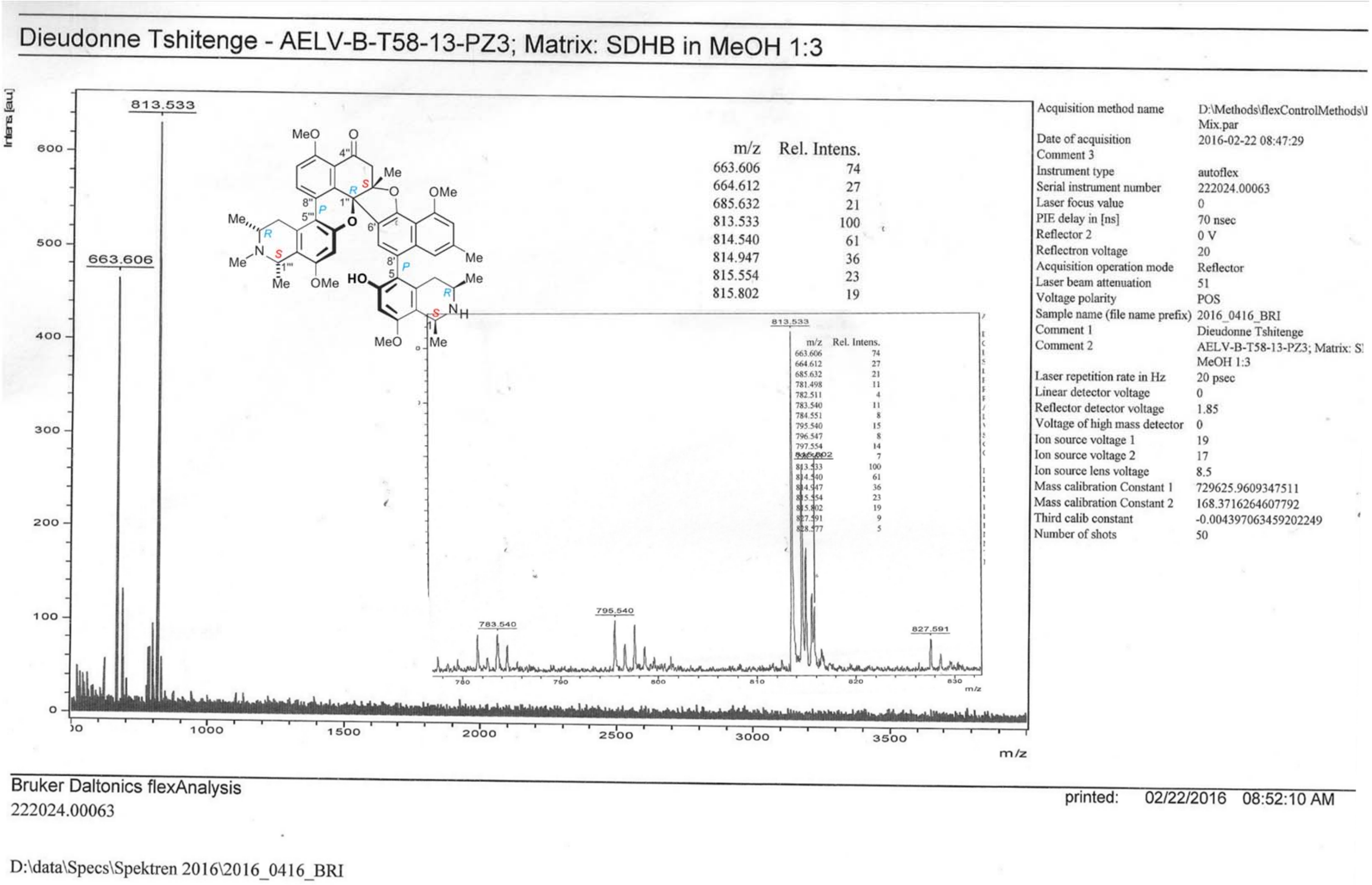

Figure S64. MALDI analysis: profile of cyclombandakamine A_5 (**5**).

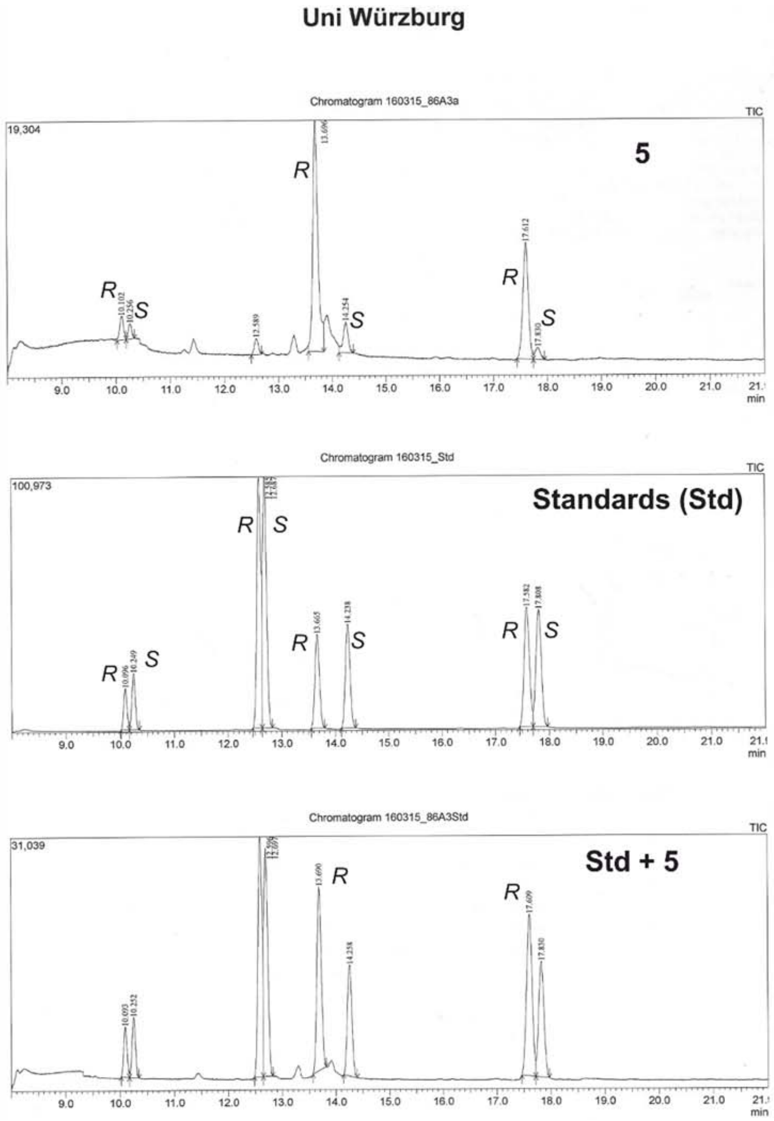

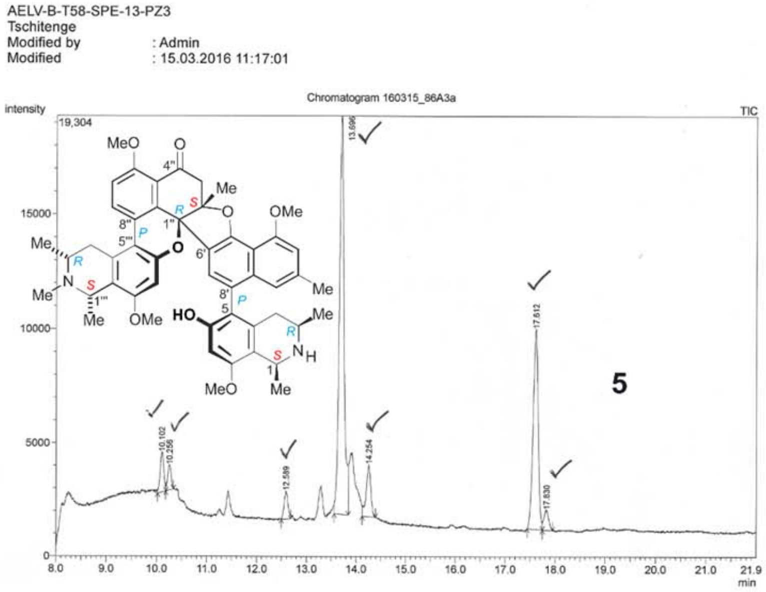

Peak#	Name	R.Time	I.Time	F.Time	Area	Area%	Height	Height%	
1		10.102	10.020	10.185	8809	4.02	1794	5.33	
2		10.256	10.185	10.335	5252	2.39	1137	3.38	
3		12.589	12.495	12.685	6617	3.02	1250	3.71	
4		13.696	13.560	13.845	116126	52.95	17447	51.82	R:S 7,88:1
5		14.254	14.125	14.390	14739	6.72	2328	6.91	
6		17.612	17.440	17.745	61813	28.19	8812	26.17	R:S 10,4:1
7		17.830	17.745	17.950	5954	2.71	903	2.68	
					219310	100.00	33671	100.00	

Figure S65. Oxidative degradation results of cyclombandakamine A_5 (**5**) (very diluted sample).

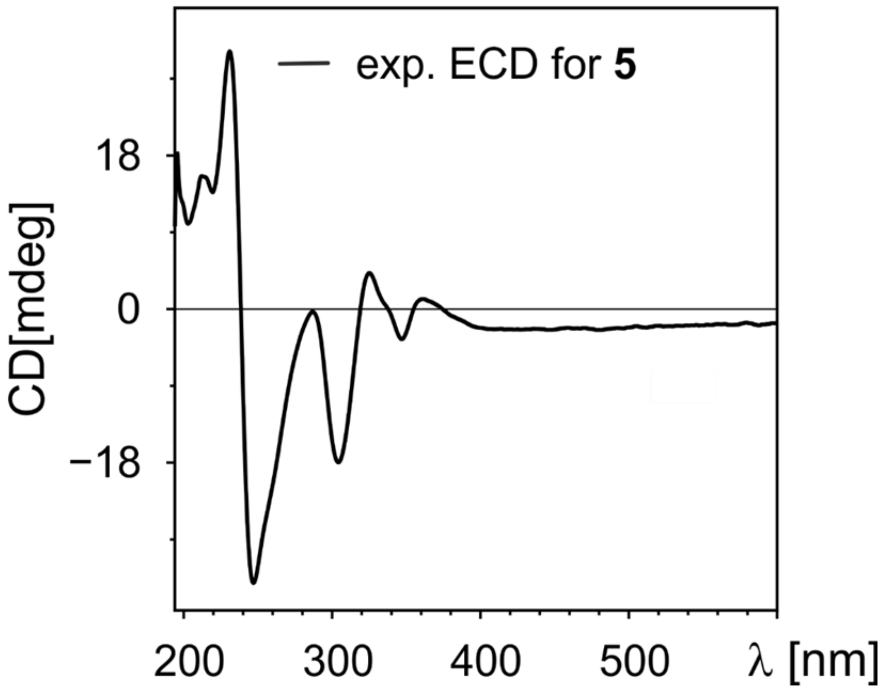

Figure S66. ECD spectrum of cyclombandakamine A_5 (**5**) in methanol.

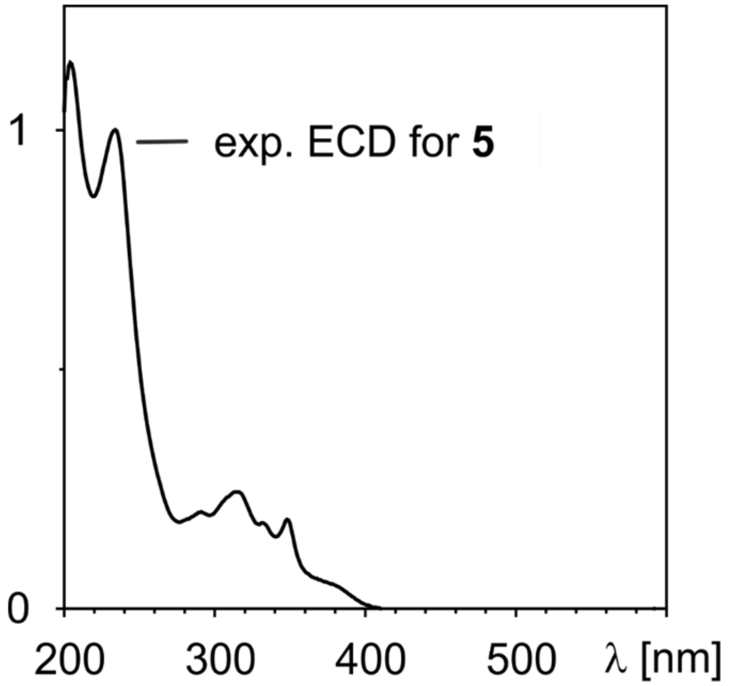

Figure S67. Offline UV spectrum of cyclombandakamine A_5 (**5**) in methanol.

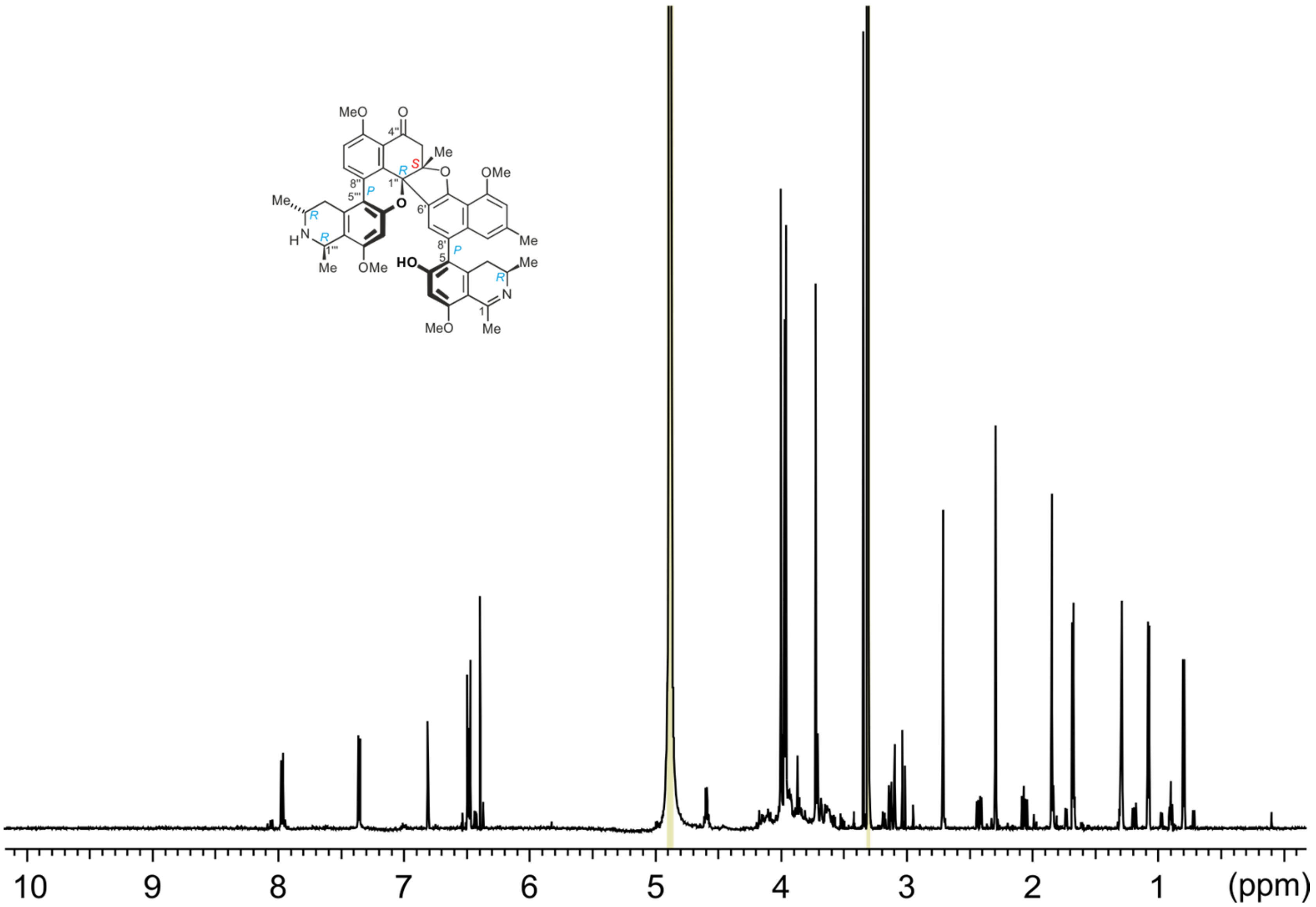

Figure S68a. Overall ^{1}H NMR spectrum of cyclombandakamine A$_6$ (**6**) in methanol-d_4.

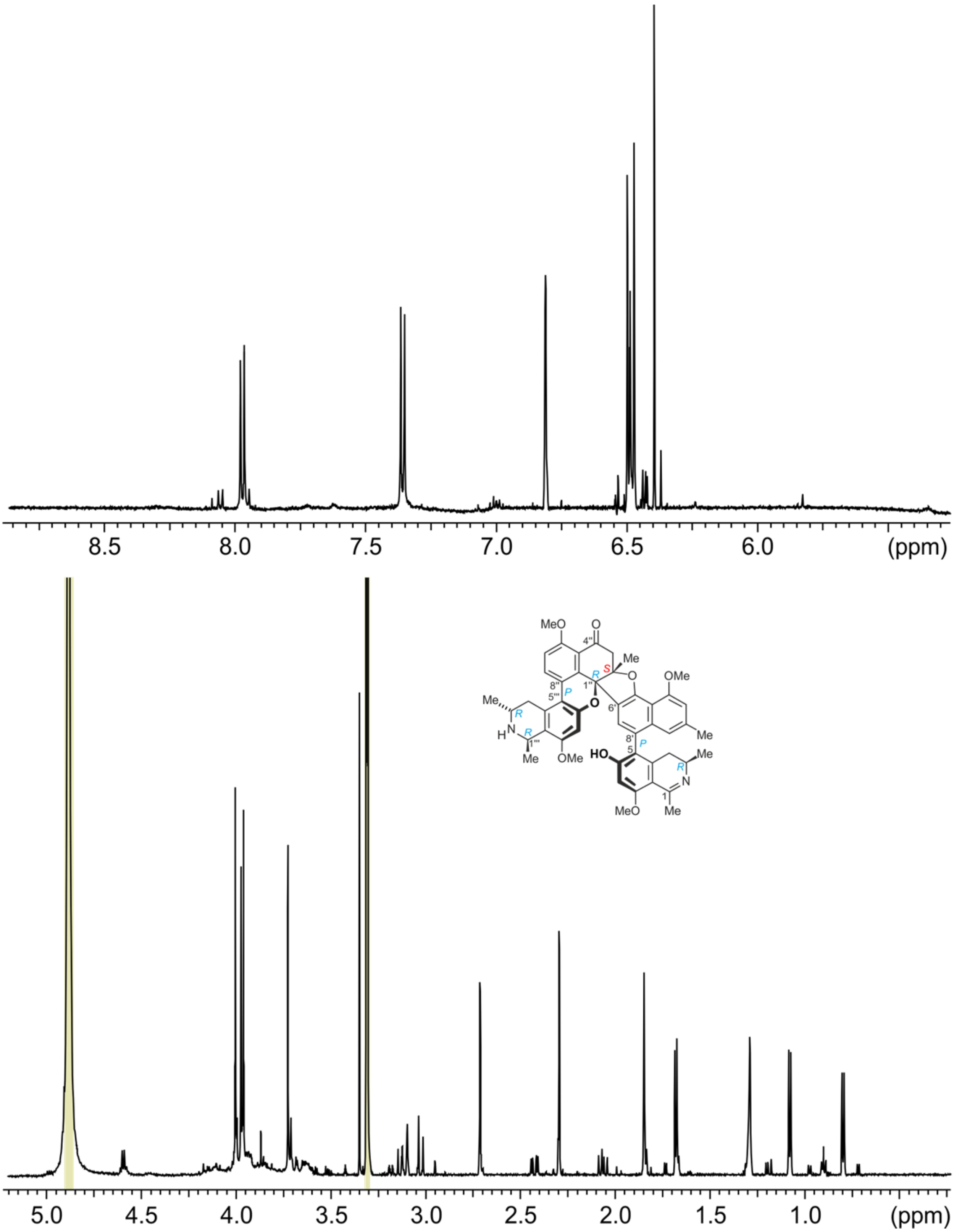

Figure S68a,c. Parts of the ^{1}H NMR spectrum of cyclombandakamine A_6 (**6**) in methanol-d_4.

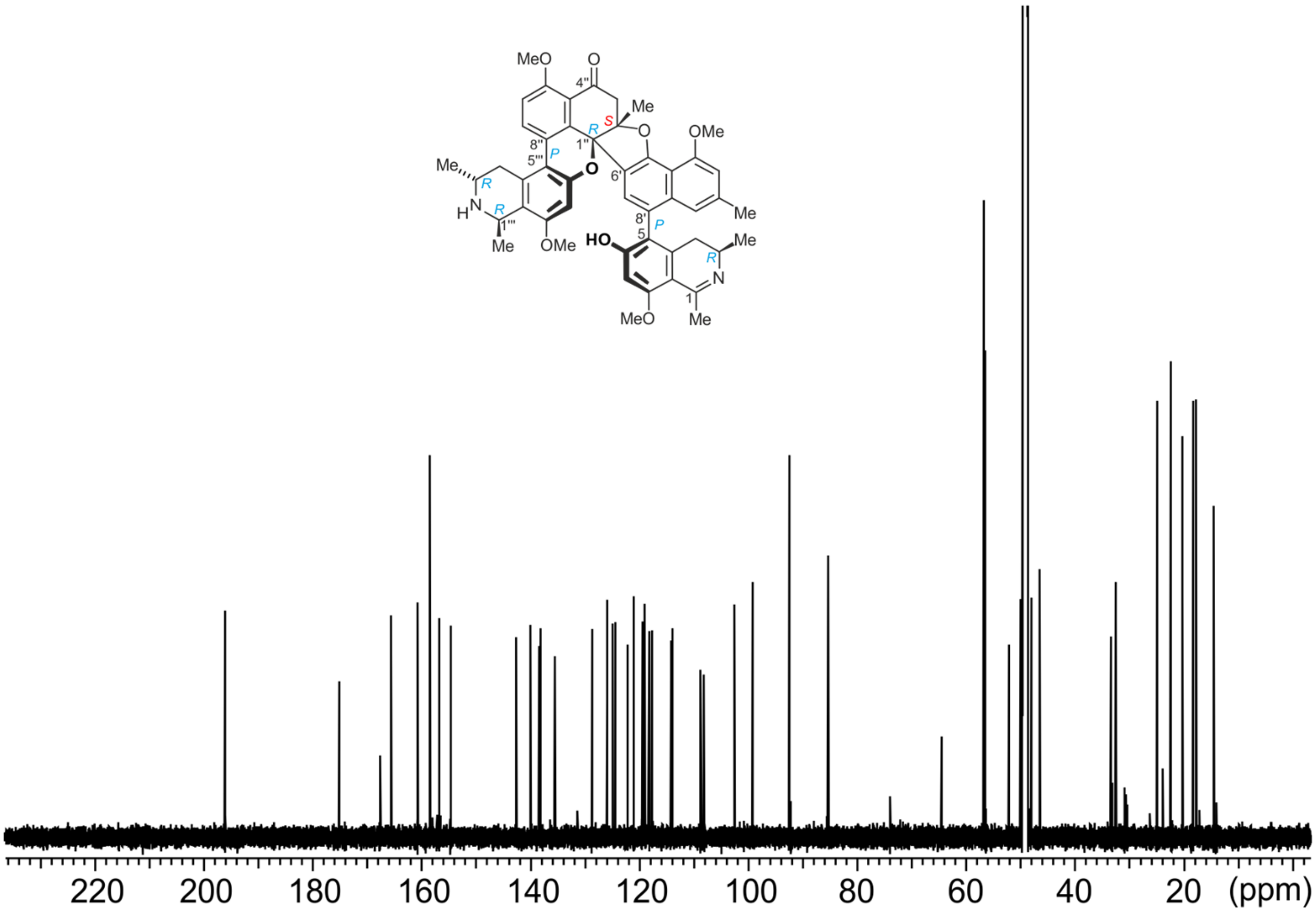

Figure S69. ^{13}C NMR spectrum of cyclombandakamine A_6 (**6**) in methanol-d_4.

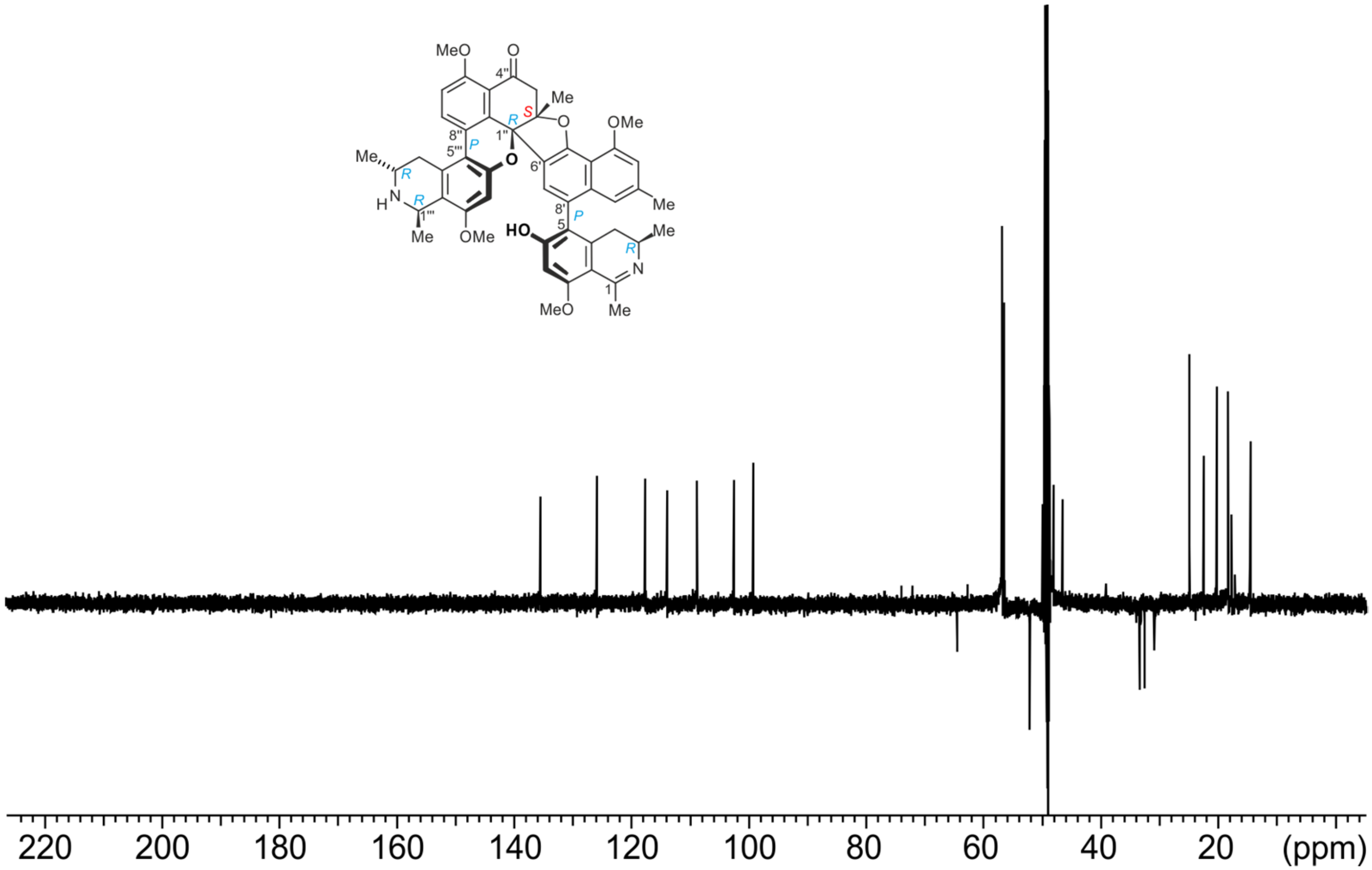

Figure S70. DEPT-135 NMR spectrum of cyclombandakamine A_6 (**6**) in methanol-d_4.

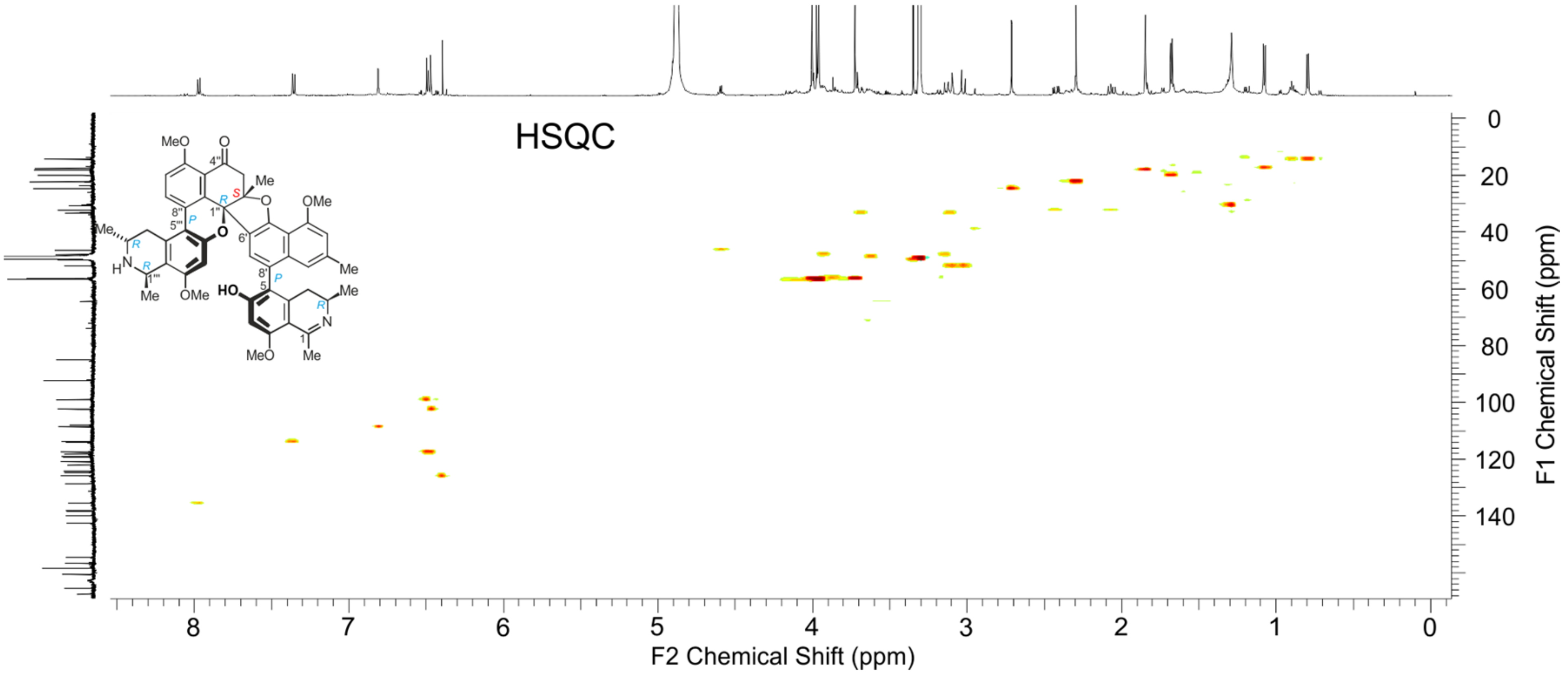

Figure S71. Overall HSQC spectrum of cyclombandakamine A_6 (**6**) in methanol-d_4.

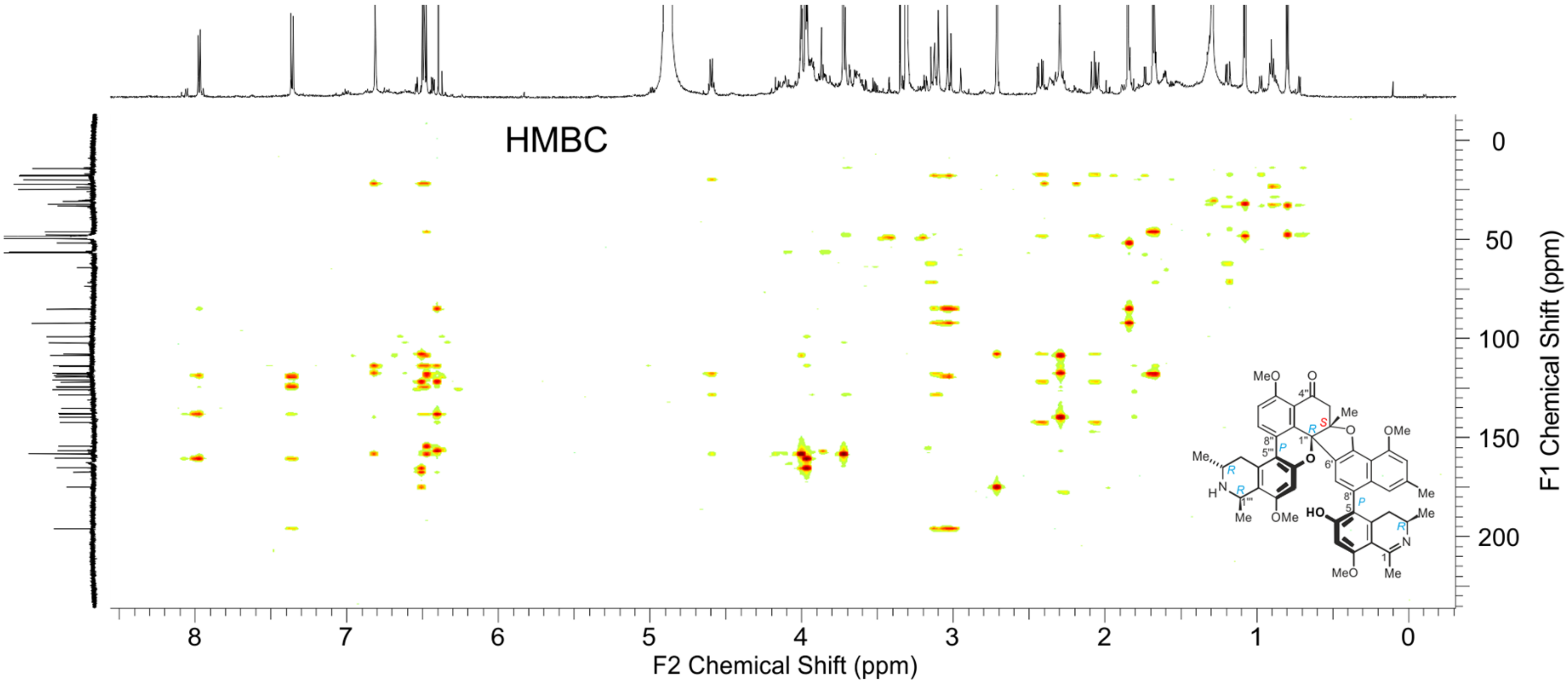

Figure S72. HMBC spectrum of cyclombandakamine A_6 (**6**) in methanol-d_4.

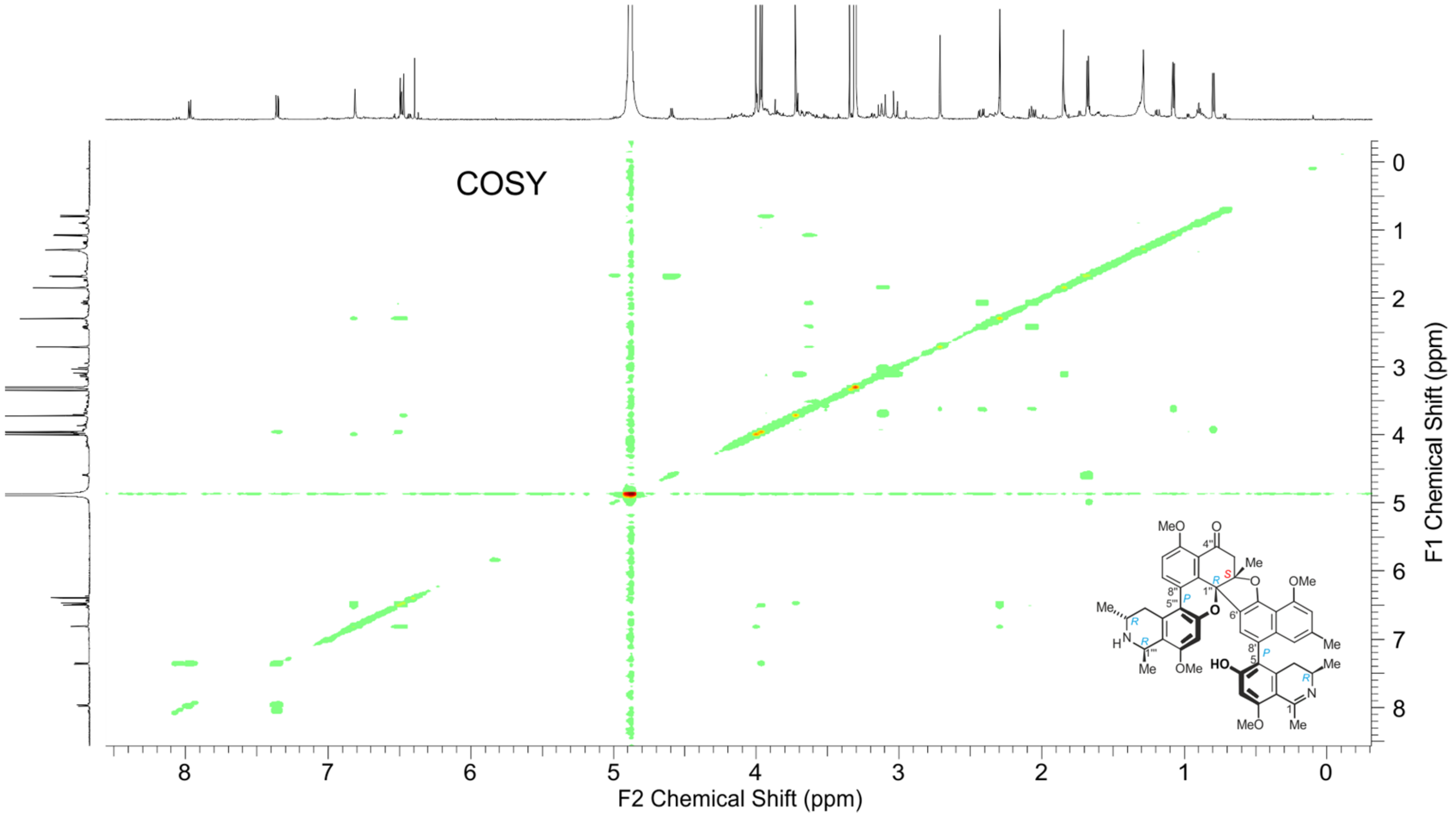

Figure S73. Overall COSY spectrum of cyclombandakamine A_6 (**6**) in methanol-d_4.

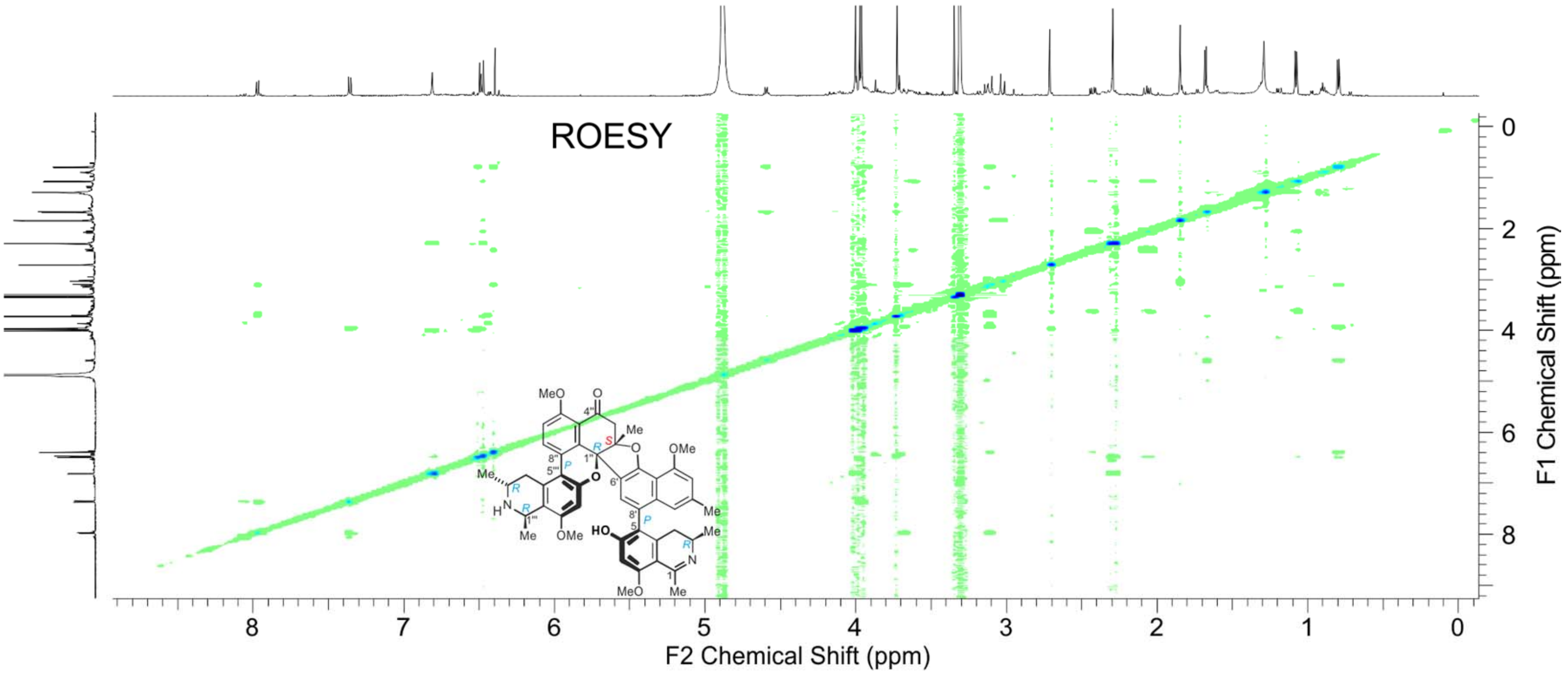

Figure S74. ROESY spectrum of cyclombandakamine A_6 (**6**) in methanol-d_4.

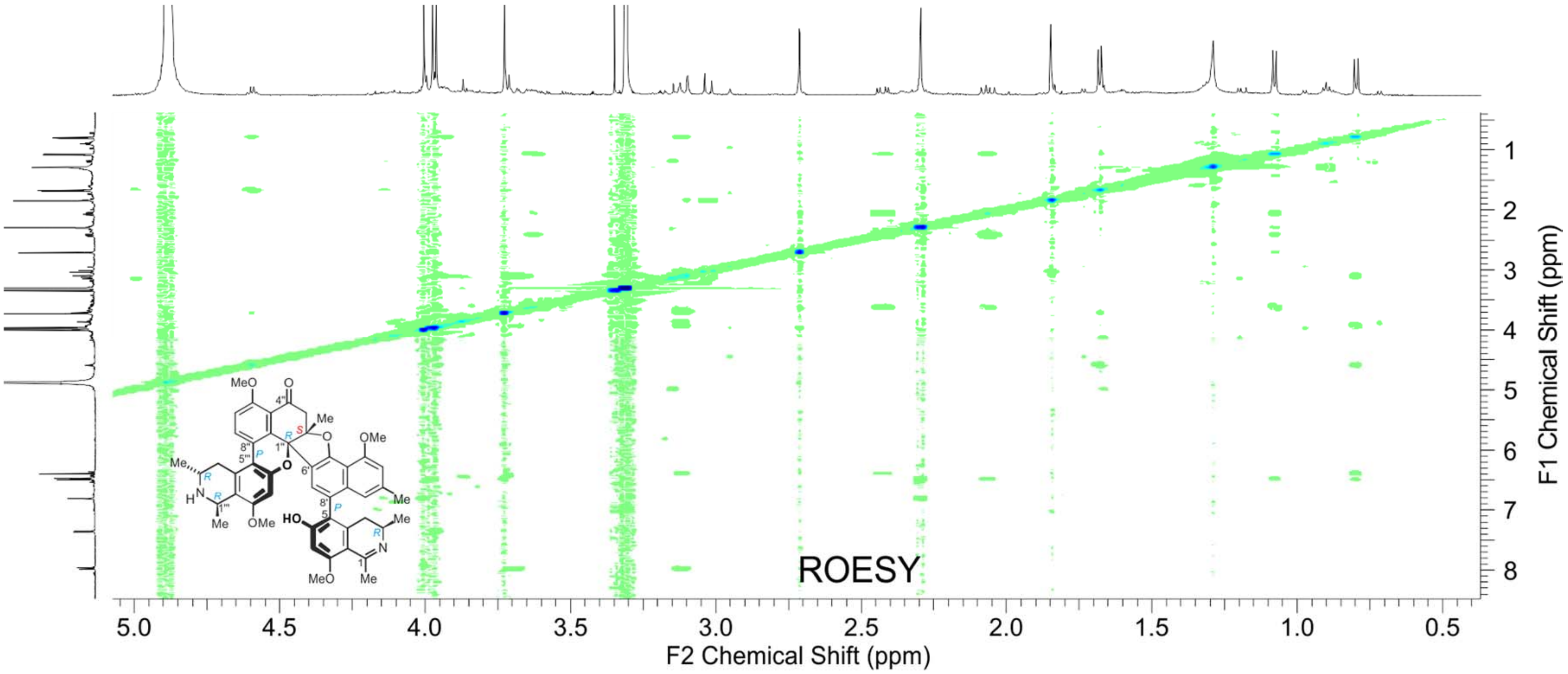

Figure S75. Aliphatic part of the ROESY spectrum of cyclombandakamine A_6 (**6**) in methanol-d_4.

Bruker Daltonics DataAnalysis 3.3

Mass Spectrum Molecular Formula Report

Analysis Info

Analysis Name	D:\Data\Spektren2015\2015_2680_BRI.d	Acquisition Date	22.12.2015 13:47:49
Method	esi_tune_pos_wide.m	Operator	Administrator
Comment	Dieudonne Tshitenge<br>AELV-B-T58-P15<br>8 pMol/µL in MeOH	Instrument	micrOTOF 88

Acquisition Parameter

Source Type	ESI	Ion Polarity	Positive	Set Corrector Fill	48 V
Scan Range	n/a	Capillary Exit	200.0 V	Set Pulsar Pull	804 V
Scan Begin	50 m/z	Hexapole RF	380.0 V	Set Pulsar Push	807 V
Scan End	3500 m/z	Skimmer 1	50.0 V	Set Reflector	1700 V
		Hexapole 1	23.0 V	Set Flight Tube	8600 V
				Set Detector TOF	2240 V

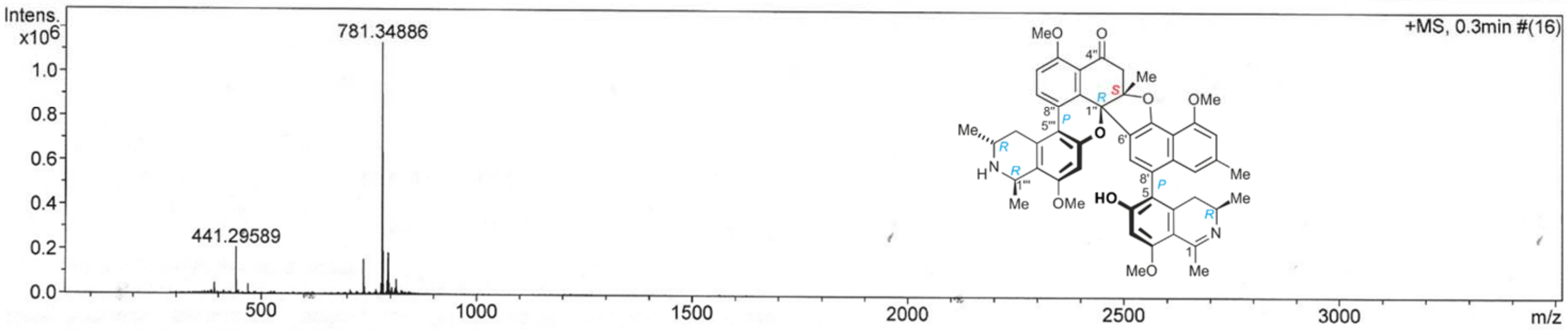

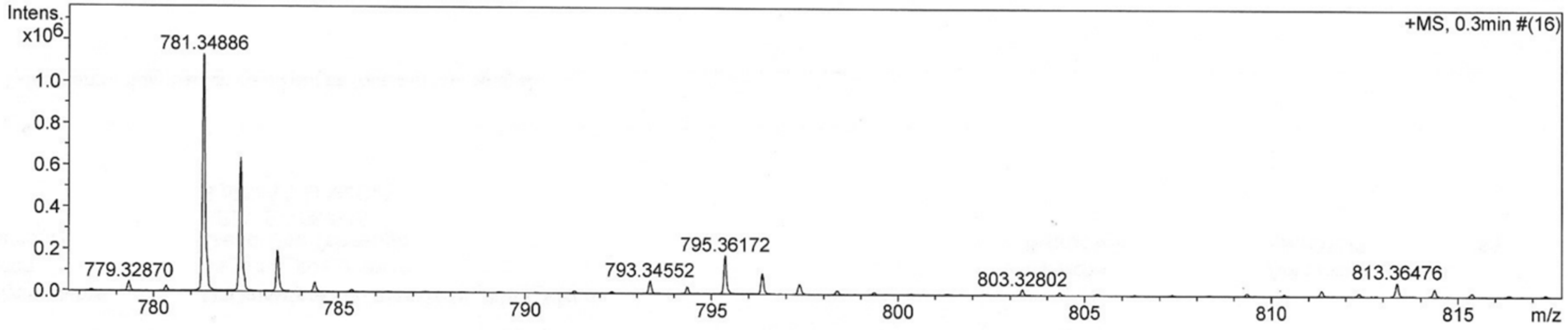

Figure S76. HRESIMS spectrum of cyclombandakamine A_6 (**6**) methanol.

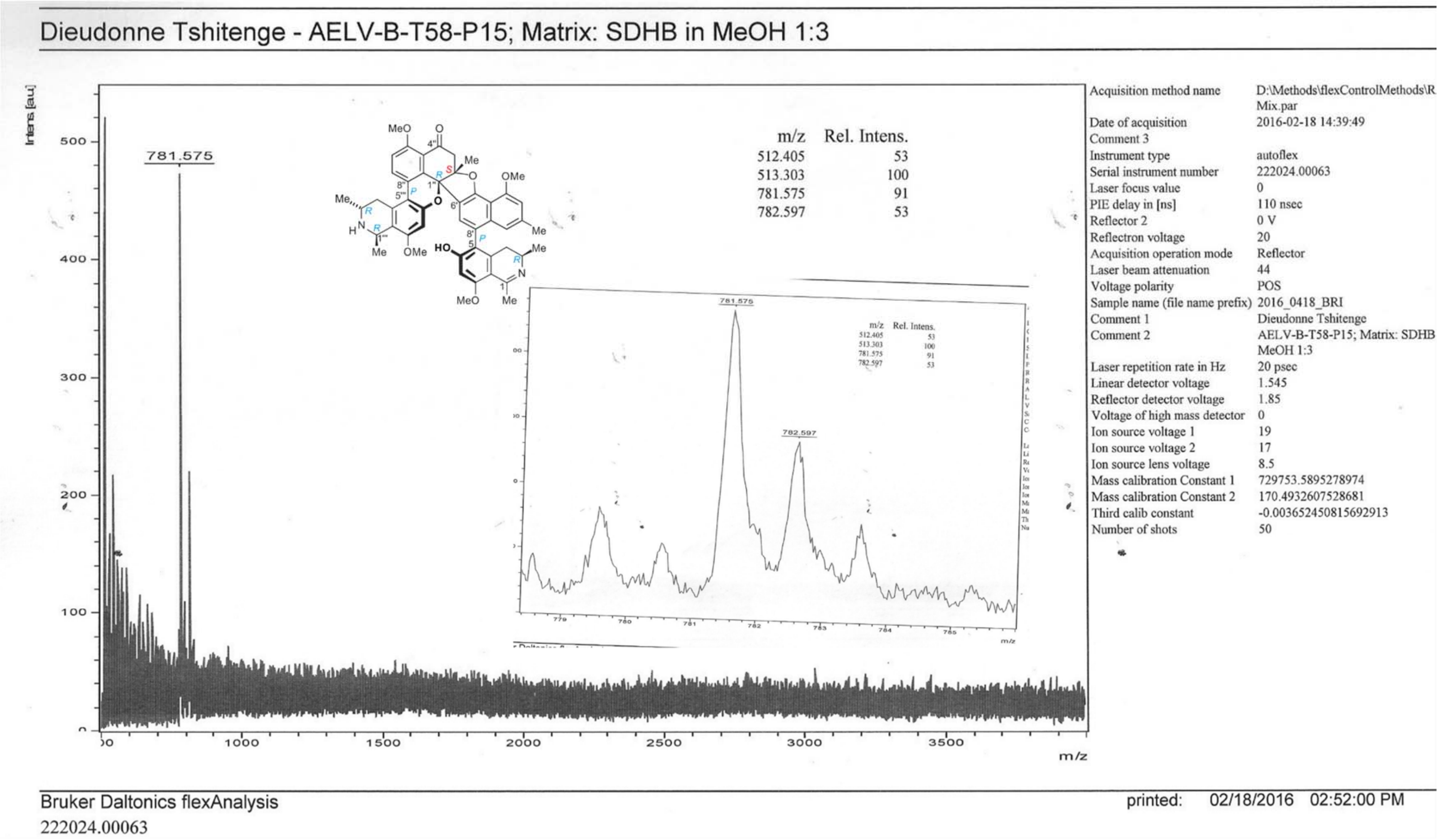

Figure S77. MALDI analysis: profile of cyclombandakamine A_6 (**6**).

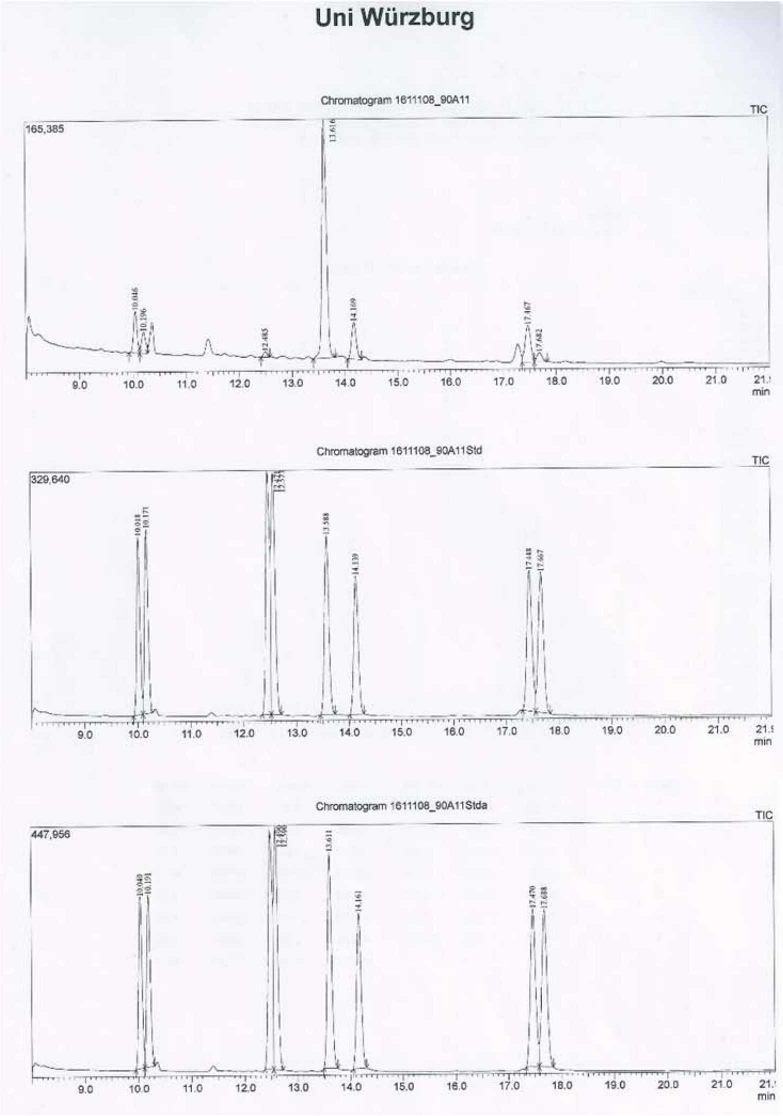

Tschitenge
Modified by : Admin
Modified : 09.11.2016 13:04:49

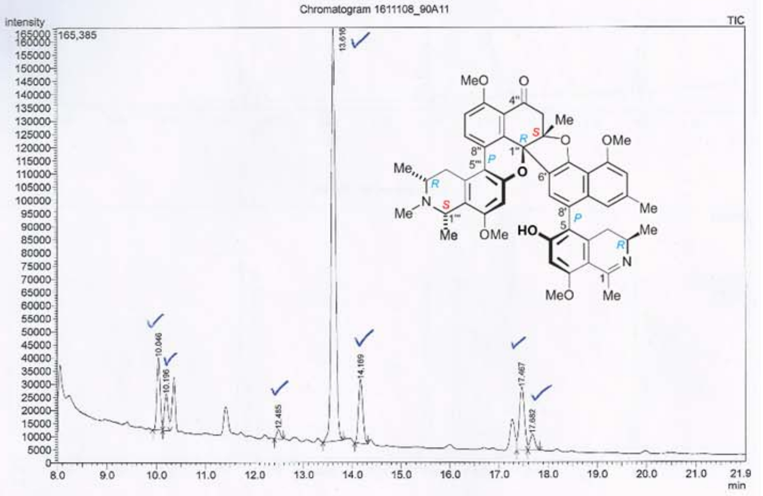

Peak#	Name	R.Time	I.Time	F.Time	Area	Area%	Height	Height%	
1		10.046	9.925	10.125	126312	8.30	27630	10.74	
2		10.196	10.125	10.275	73234	4.81	13951	5.42	
3		12.485	12.405	12.580	18916	1.24	3605	1.40	R-N-Me-ALA
4		13.616	13.395	13.810	934983	61.42	157236	61.12	N-H-ABA
5		14.169	14.045	14.315	150521	9.89	24496	9.52	R:S 6,2:1
6		17.467	17.360	17.590	175074	11.50	24081	9.36	N-Me-ABA
7		17.682	17.590	17.830	43323	2.85	6248	2.43	R:S 4:1
					1522363	100.00	257247	100.00	

Figure S78. Oxidative degradation results of cyclombandakamine A_6 (**6**) (very diluted sample).

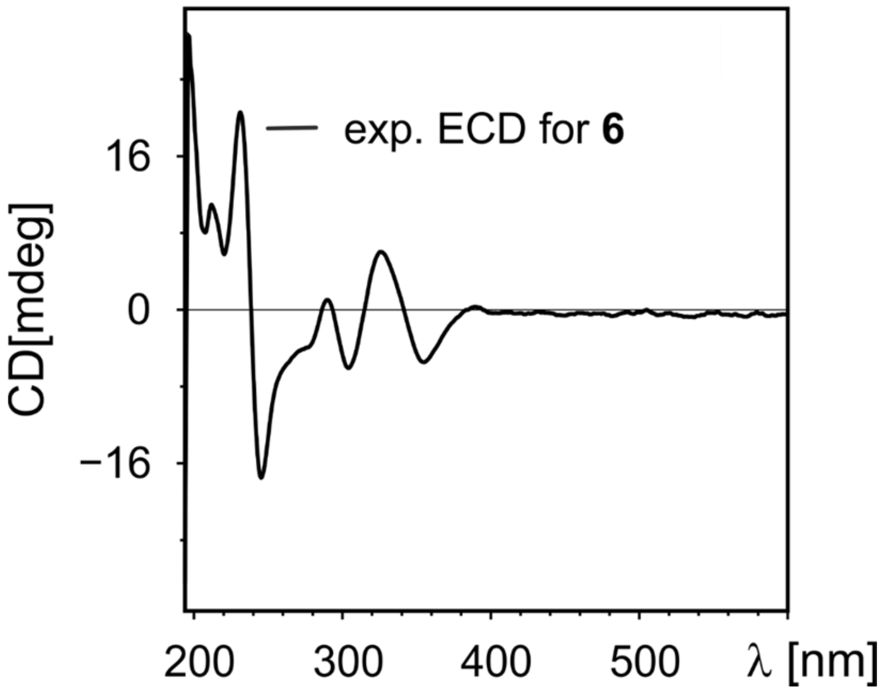

Figure S79. ECD spectrum of cyclombandakamine A_6 (**6**) in methanol.

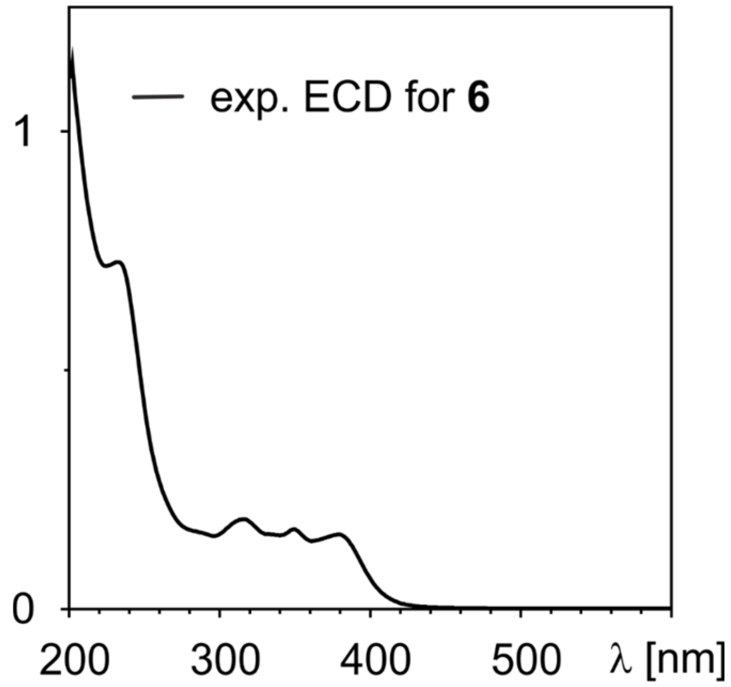

Figure S80. Offline UV spectrum of cyclombandakamine A_6 (**6**) in methanol.

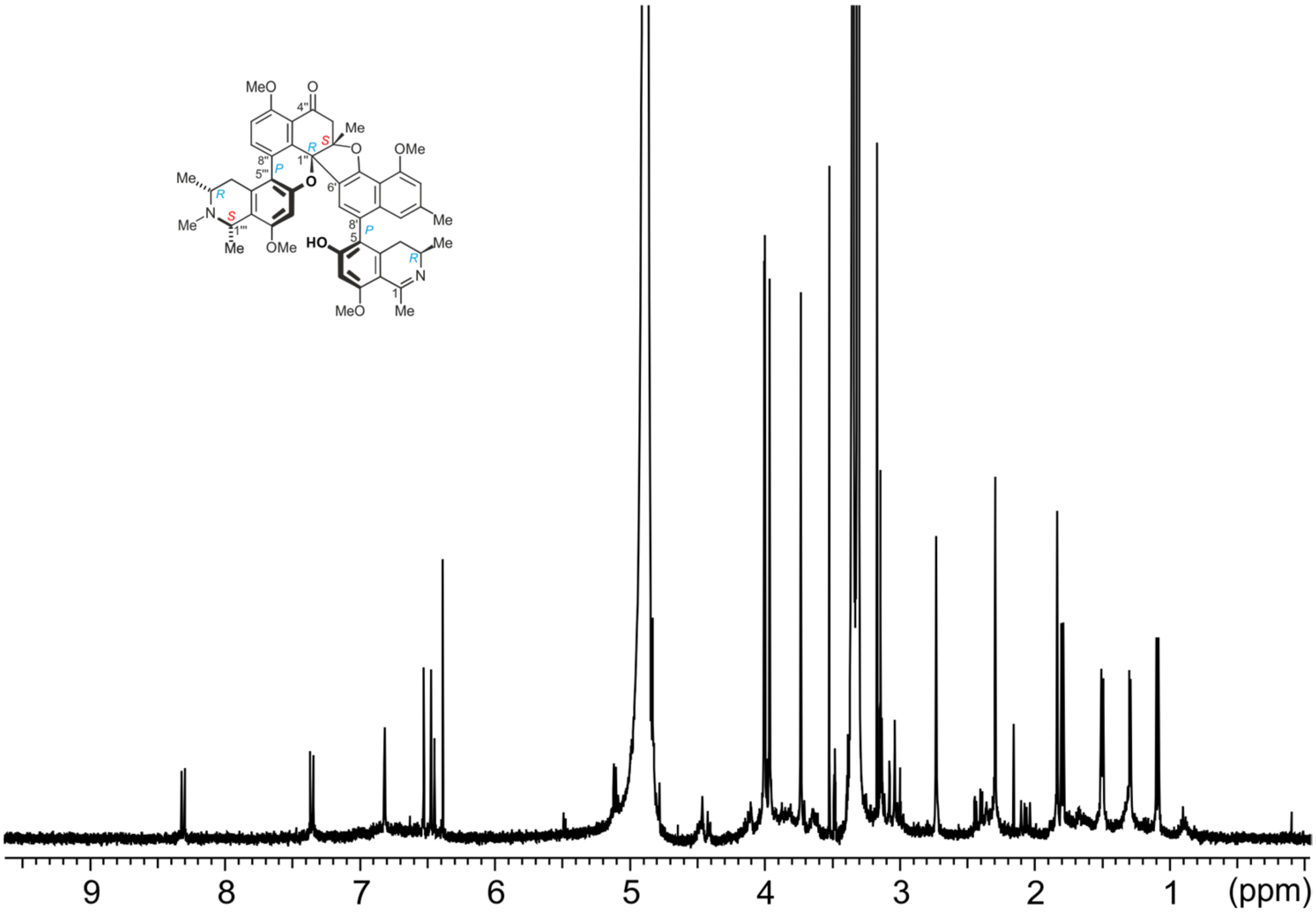

Figure S81a. Overall ^{1}H NMR spectrum of cyclombandakamine A_7 (**7**) in methanol-d_4.

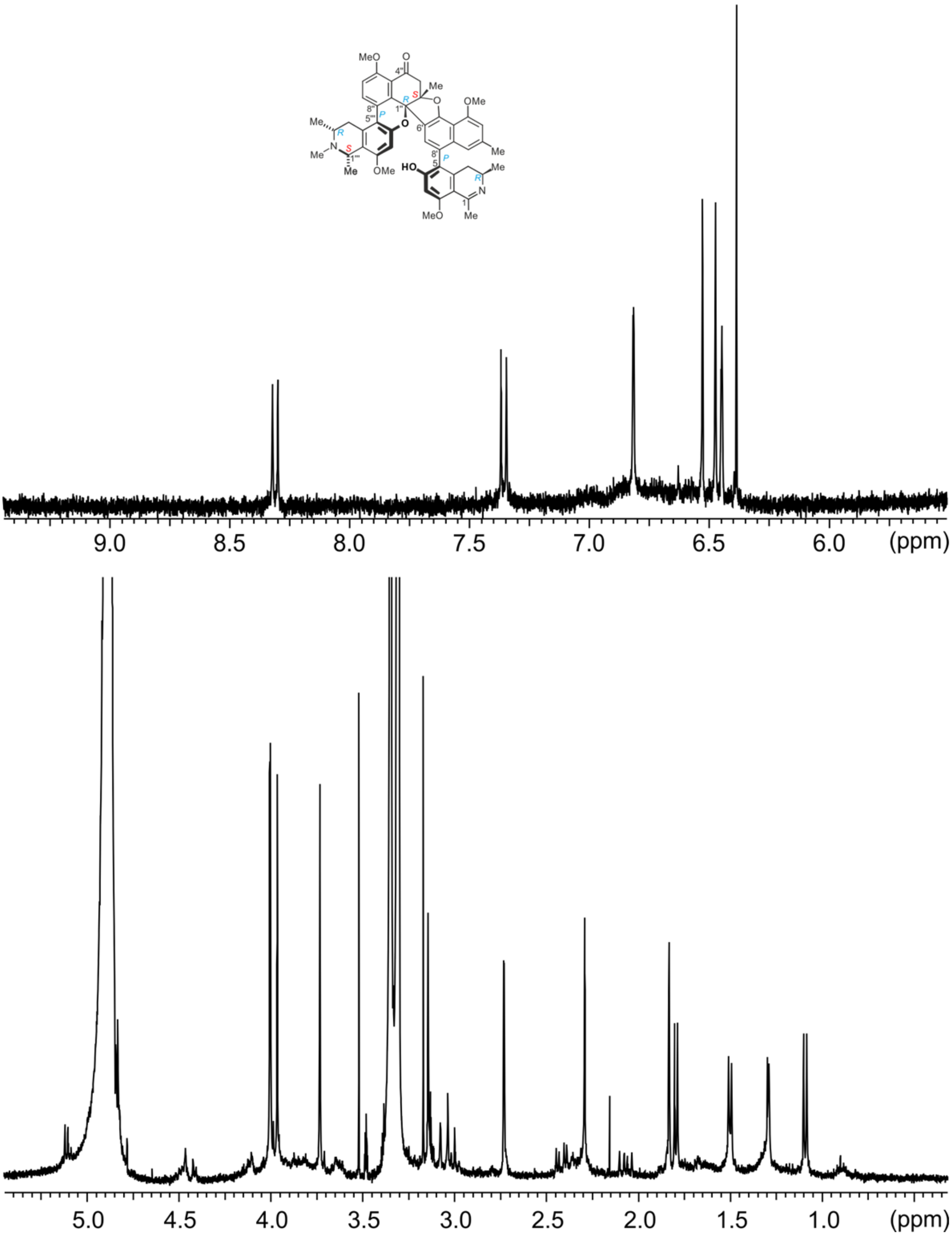

Figure S81b,c. Parts of the ^{1}H NMR spectrum of cyclombandakamine A_7 (**7**) in methanol-d_4.

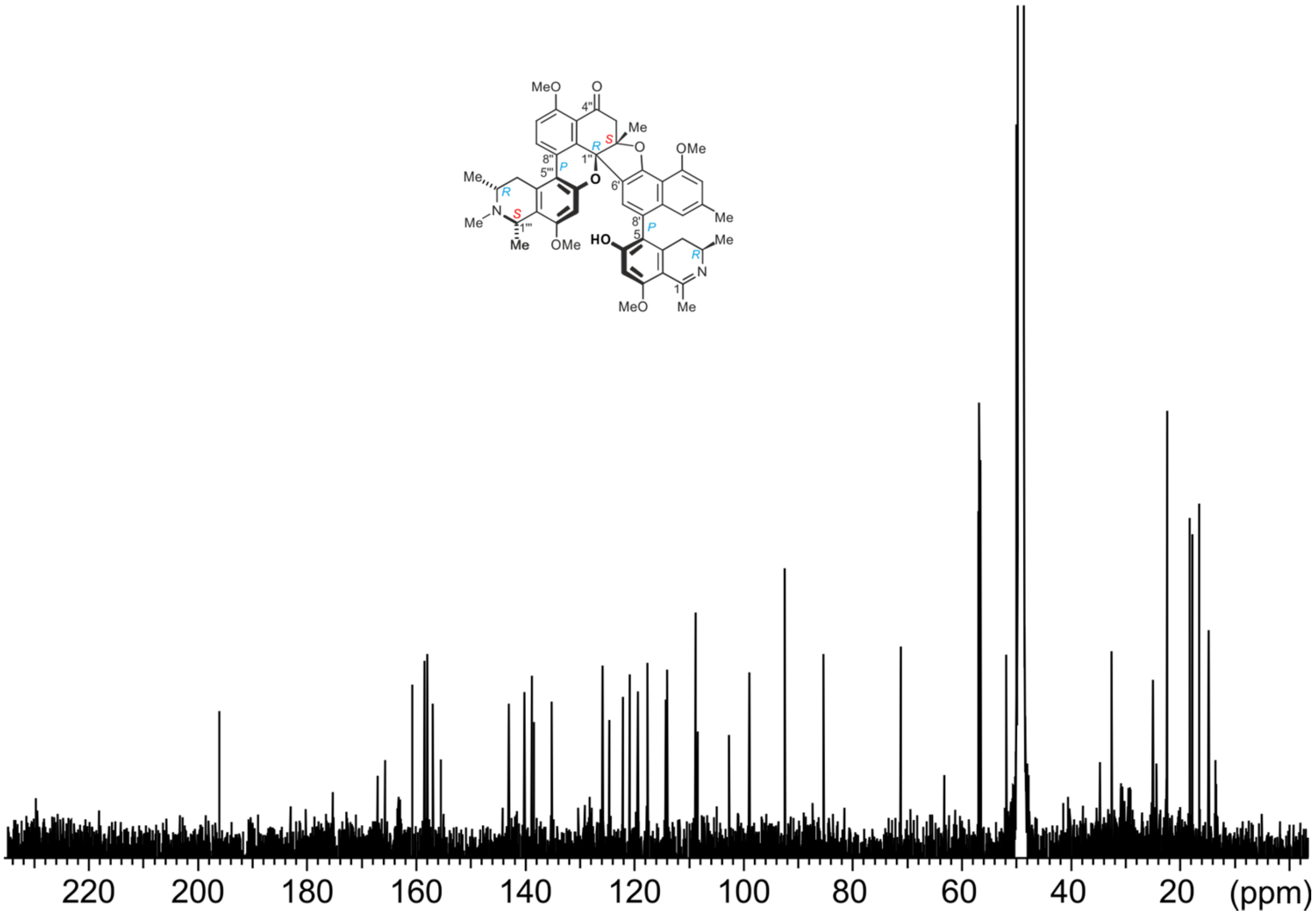

Figure S82. ^{13}C NMR spectrum of cyclombandakamine A$_7$ (**7**) in methanol-d_4.

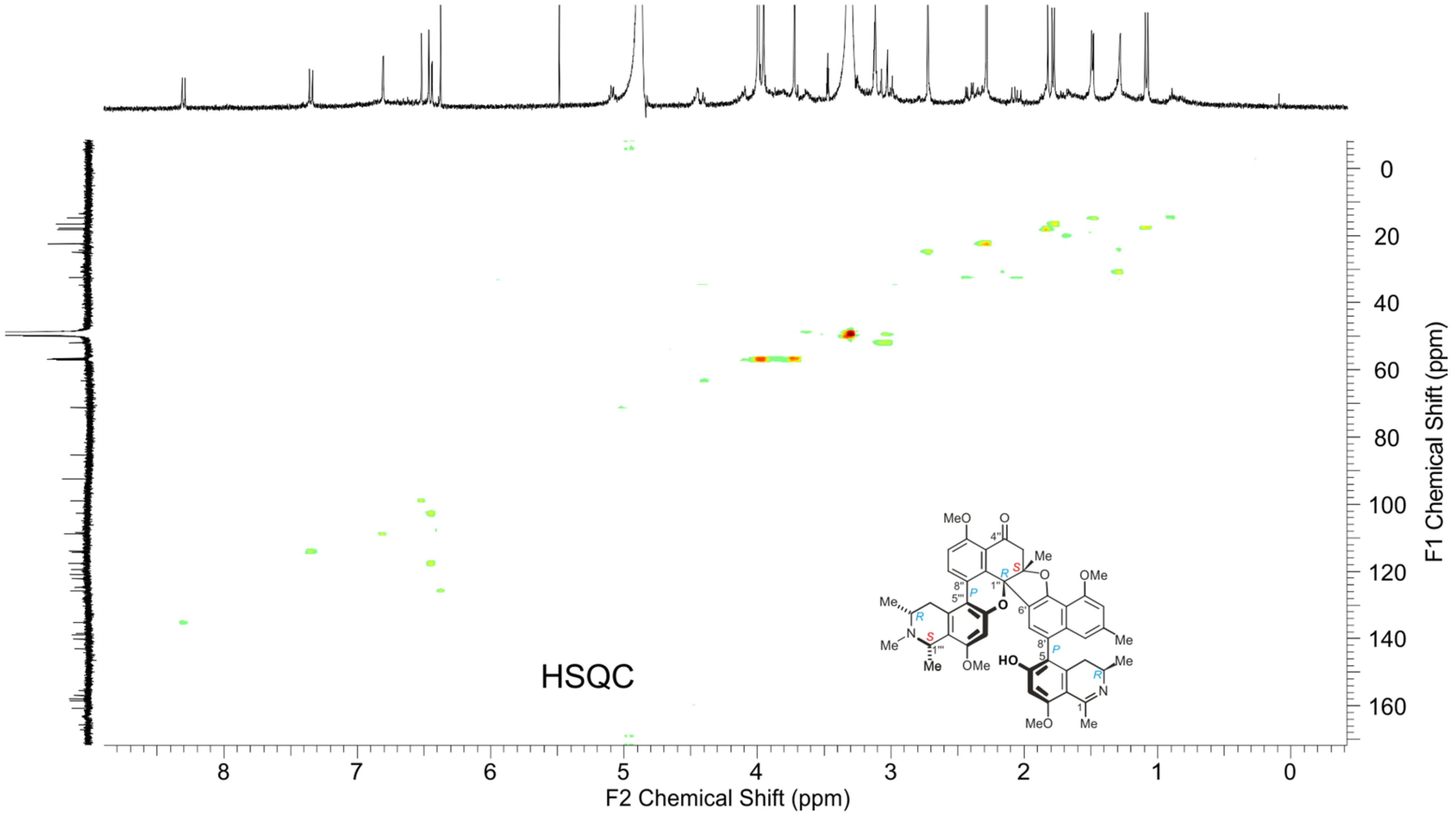

Figure S83a. Overall HSQC spectrum of cyclombandakamine A_7 (**7**) in methanol-d_4.

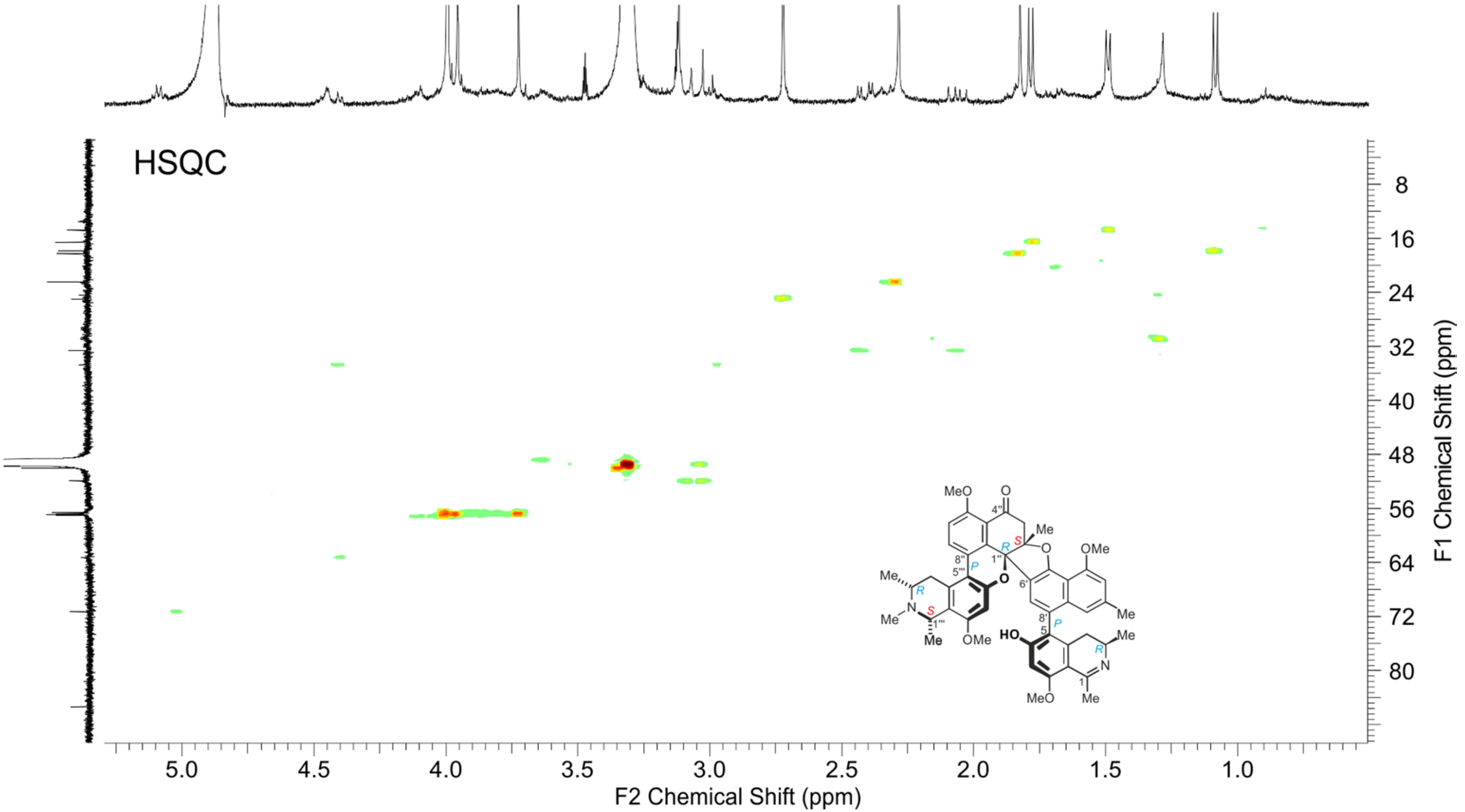

Figure S83b. Part of the HSQC spectrum of cyclombandakamine A_7 (**7**) in methanol-d_4.

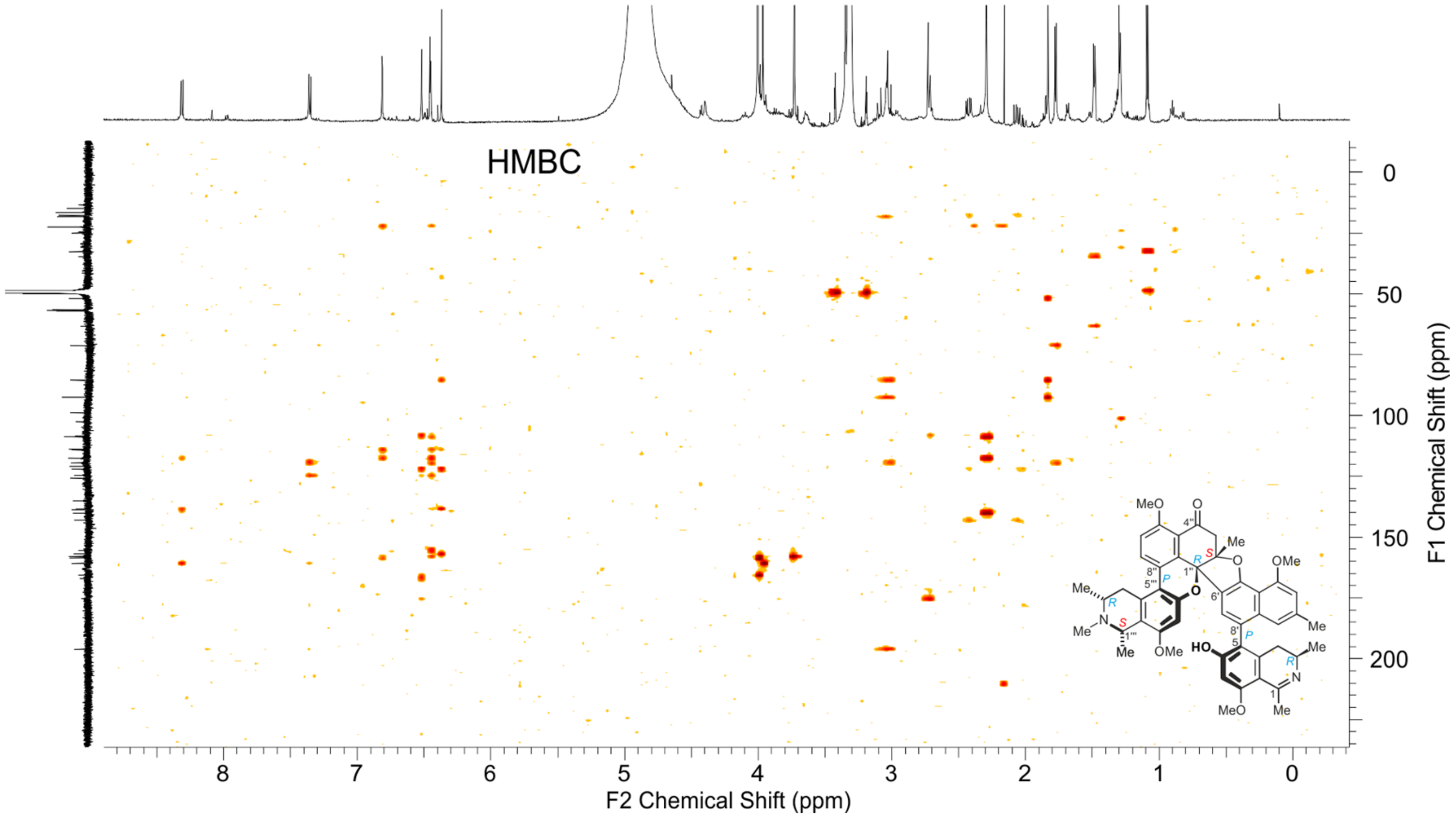

Figure S84. HMBC spectrum of cyclombandakamine A_7 (**7**) in methanol-d_4.

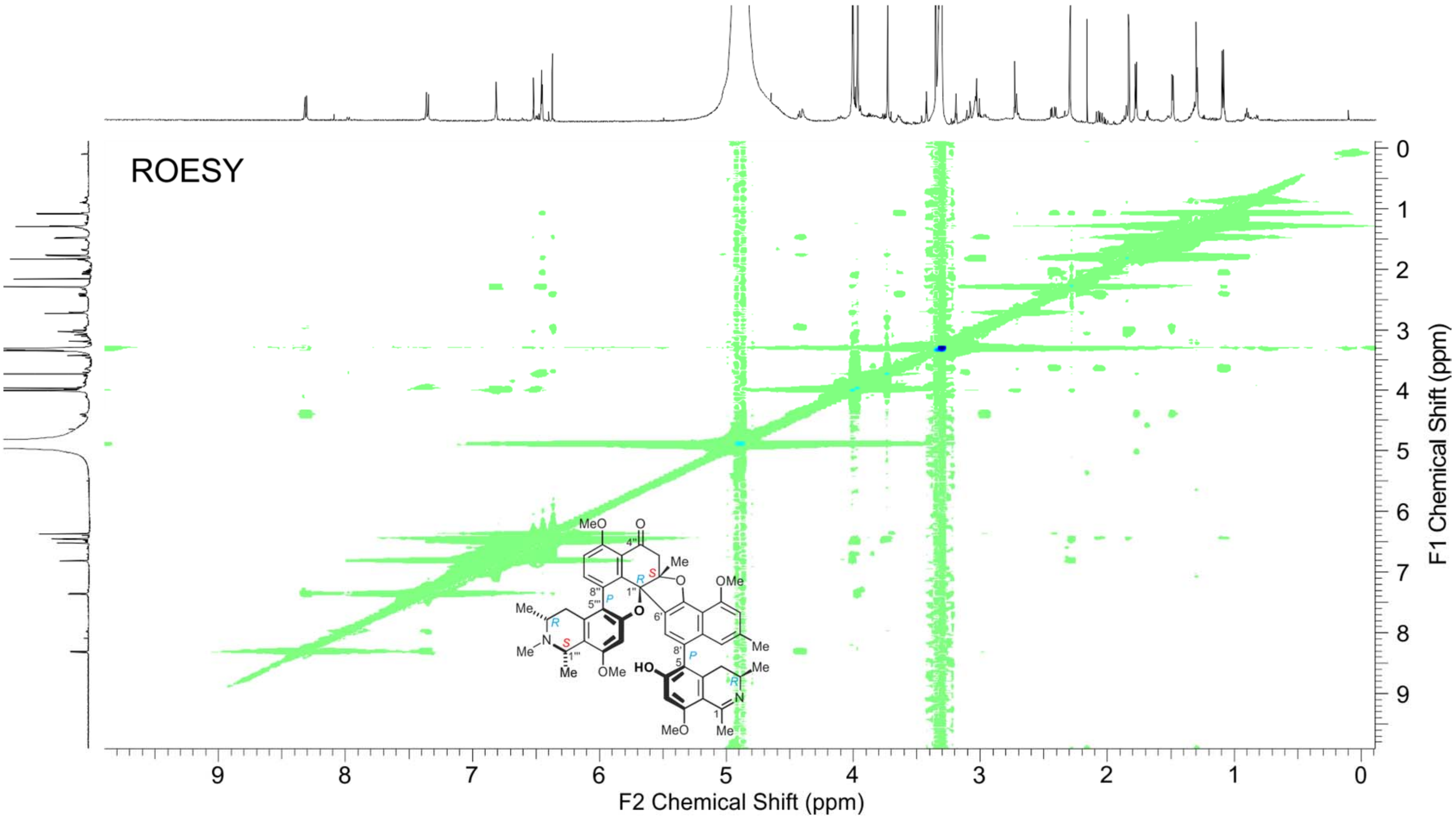

Figure S85a. ROESY spectrum of cyclombandakamine A$_7$ (**7**) in methanol-d_4.

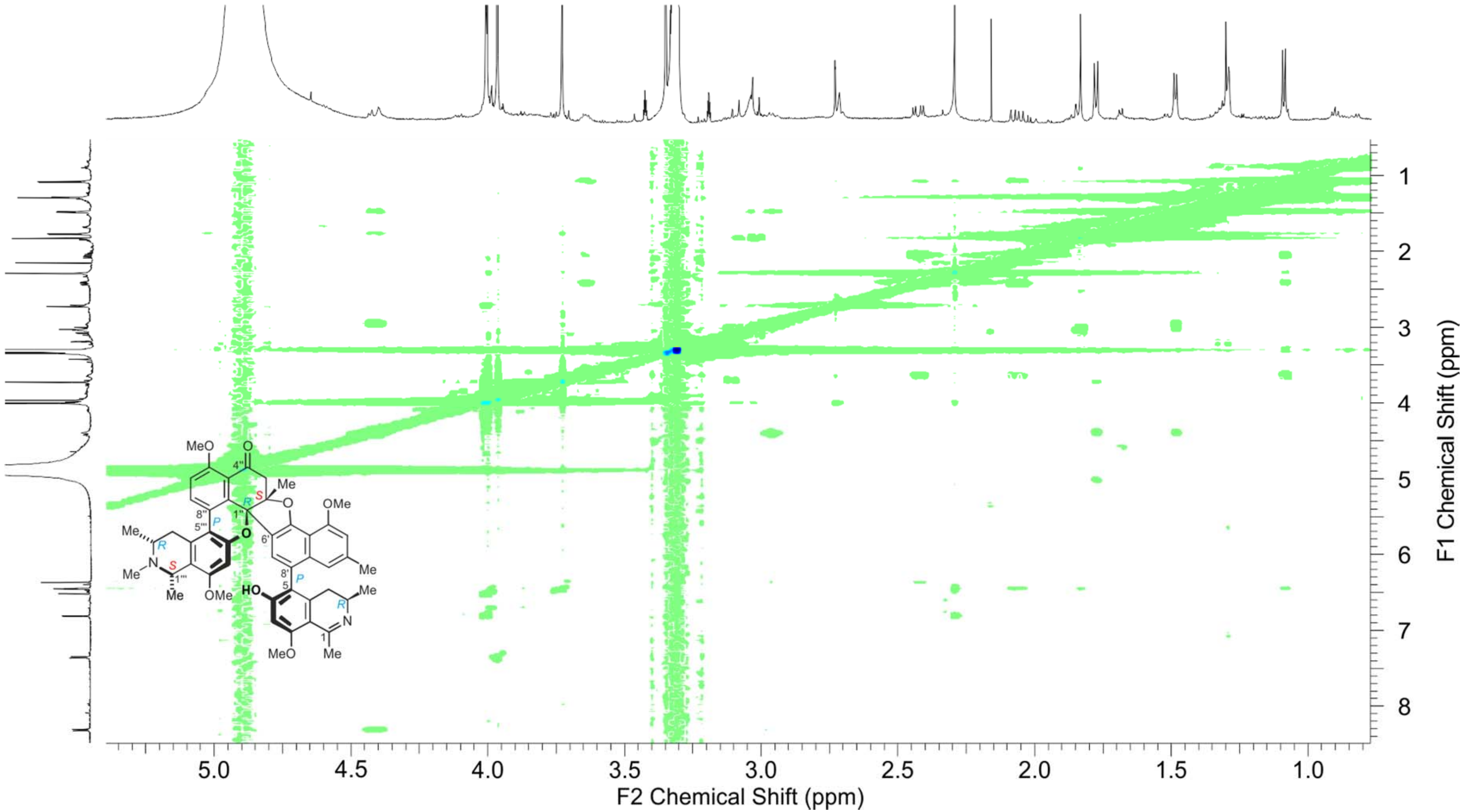

Figure S85b. Aliphatic part of ROESY spectrum of cyclombandakamine A_7 (**7**) in methanol-d_4.

Bruker Daltonics DataAnalysis 3.3

Mass Spectrum Molecular Formula Report

Analysis Info

Analysis Name	M:\microtof\data\Spektren2016\2016_1327_BRI.d	Acquisition Date	01.06.2016 13:29:39
Method	esi_tune_pos_wide.m	Operator	Administrator
Comment	Dieudonne Tshitenge<br>AELV-B5935-P13*<br>undiluted	Instrument	micrOTOF 88

Acquisition Parameter

Source Type	ESI	Ion Polarity	Positive	Set Corrector Fill	48 V
Scan Range	n/a	Capillary Exit	220.0 V	Set Pulsar Pull	804 V
Scan Begin	50 m/z	Hexapole RF	580.0 V	Set Pulsar Push	807 V
Scan End	3000 m/z	Skimmer 1	50.0 V	Set Reflector	1700 V
		Hexapole 1	23.0 V	Set Flight Tube	8600 V
				Set Detector TOF	2340 V

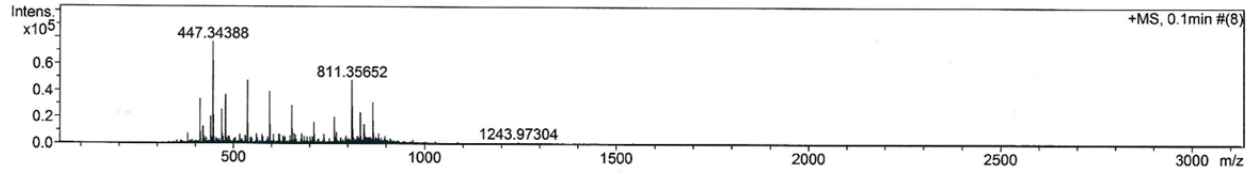

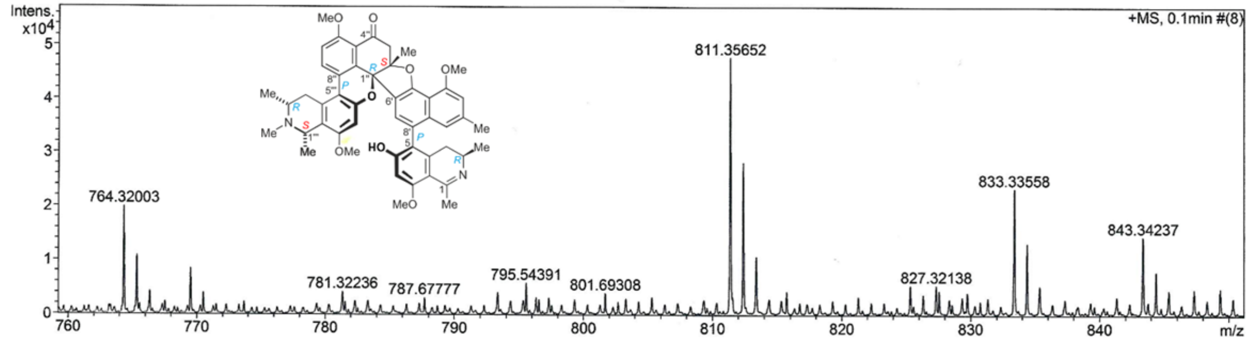

Analysis Name	M:\microtof\data\Spektren2016\2016_1327_BRI.d		
Method	esi_tune_pos_wide.m	Operator	Administrator
Comment	Dieudonne Tshitenge<br>AELV-B5935-P13*<br>undiluted	Instrument	micrOTOF 88

Sum Formula	Sigma	m/z	Err [ppm]	Mean Err [ppm]	rdb	N Rule	e⁻
C 50 H 50 N 3 Na 1 O 6	0.04	811.35918	3.28	5.14	27.00	-	odd
C 49 H 51 N 2 O 9	0.04	811.35891	2.94	4.75	25.50	ok	even

Figure S86. HRESIMS spectrum of cyclombandakamine A_7 (**7**) methanol.

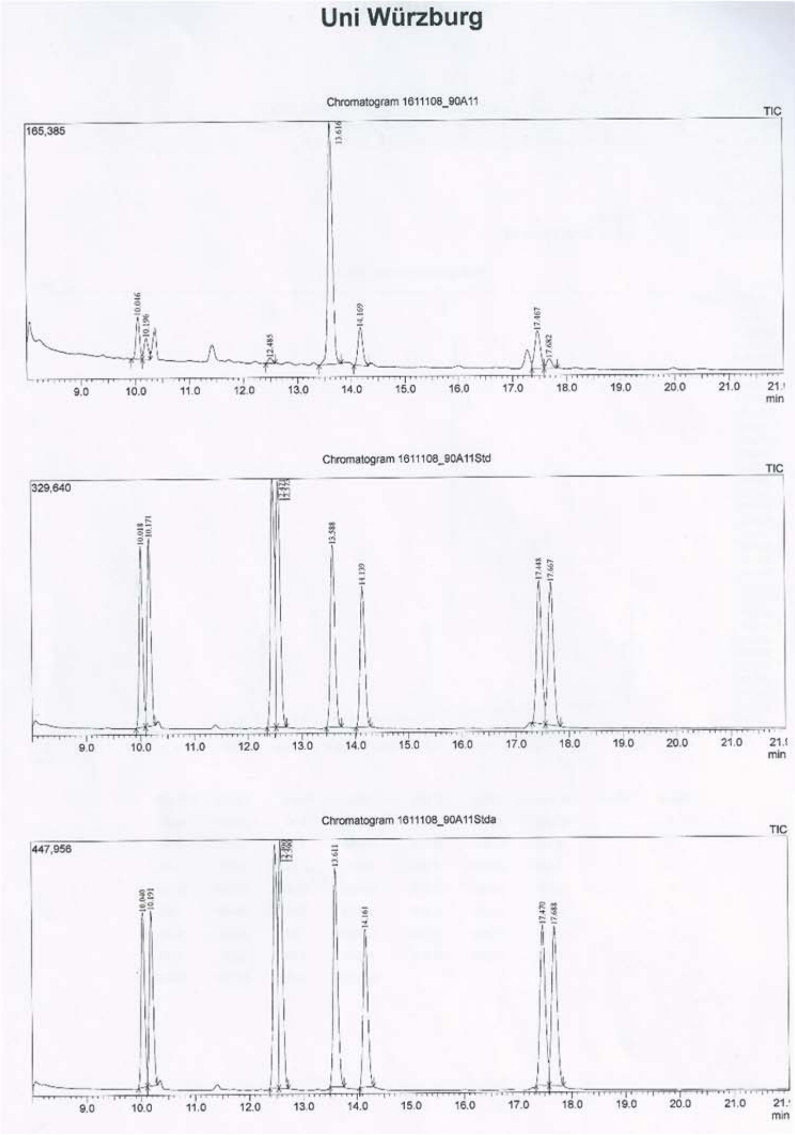

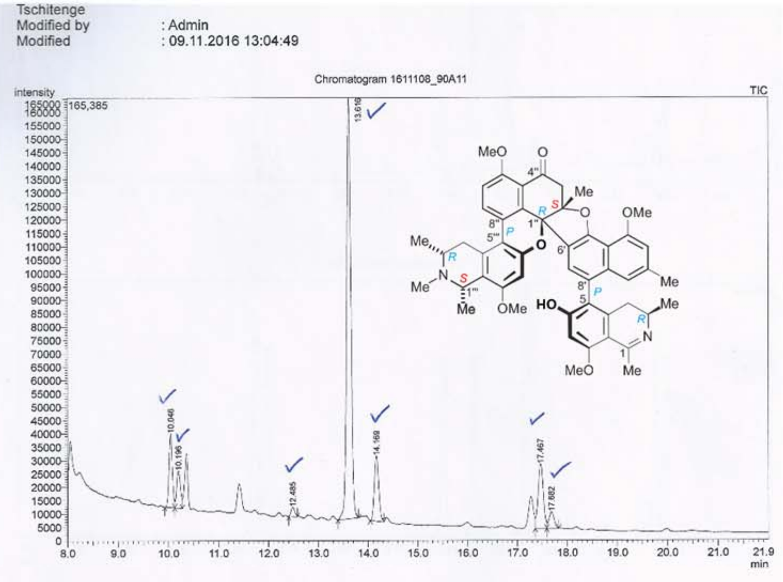

Peak#	Name	R.Time	I.Time	F.Time	Area	Area%	Height	Height%	
1		10.046	9.925	10.125	126312	8.30	27630	10.74	
2		10.196	10.125	10.275	73234	4.81	13951	5.42	
3		12.485	12.405	12.580	18916	1.24	3605	1.40	R-N-Me-ALA
4		13.616	13.395	13.810	934983	61.42	157236	61.12	N-H-ABA R:S 6,2:1
5		14.169	14.045	14.315	150521	9.89	24496	9.52	
6		17.467	17.360	17.590	175074	11.50	24081	9.36	N-Me-ABA R:S 4:1
7		17.682	17.590	17.830	43323	2.85	6248	2.43	
					1522363	100.00	257247	100.00	

Figure S87. Oxidative degradation results of cyclombandakamine A_7 (**7**) (very diluted sample).

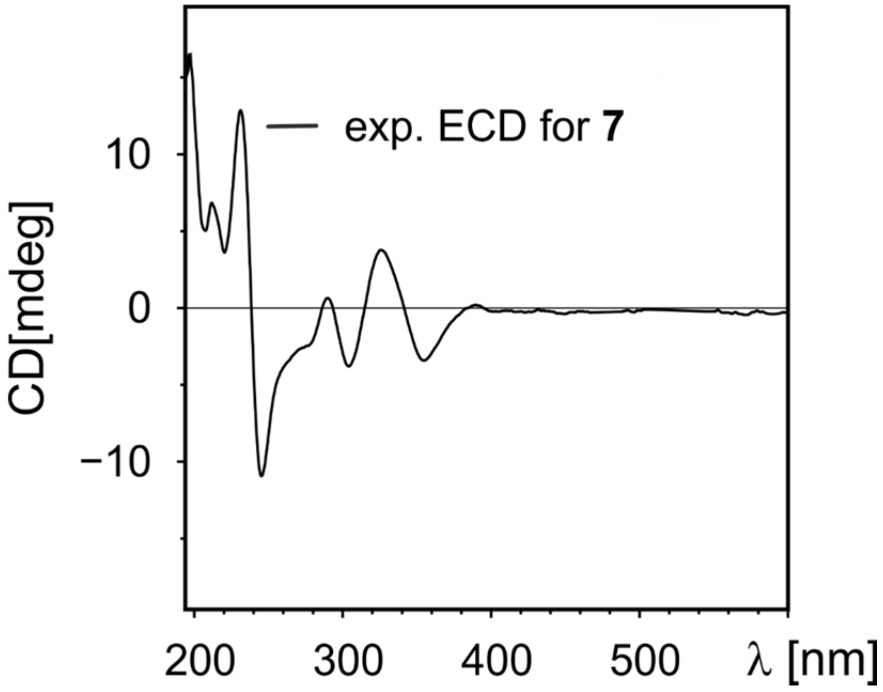

Figure S89. ECD spectrum of cyclombandakamine A_7 (**7**) in methanol.

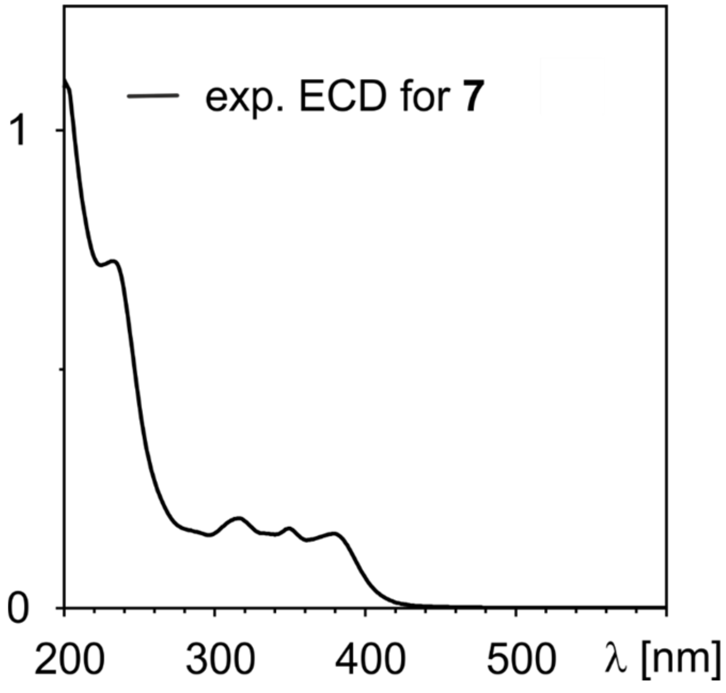

Figure S90. Offline UV spectrum of cyclombandakamine A_7 (**7**) in methanol.
